# Gains and losses of metabolic function inferred from a phylotranscriptomic analysis of algae

**DOI:** 10.1038/s41598-019-46869-3

**Published:** 2019-07-19

**Authors:** Falicia Qi Yun Goh, Justin Jeyakani, Phornpimon Tipthara, Amaury Cazenave-Gassiot, Rajoshi Ghosh, Nicholas Bogard, Zhenxuan Yeo, Gane Ka-Shu Wong, Michael Melkonian, Markus R. Wenk, Neil D. Clarke

**Affiliations:** 1 Yale-NUS College Singapore, 138527 Singapore, Singapore; 2 Computational and Systems Biology, Genome Institute of Singapore, Singapore, 138672 Singapore; 3 Department of Biochemistry, Yong Loo Lin School of Medicine National University of Singapore, Singapore, 117596 Singapore; 4 Department of Biological Sciences, National University of Singapore, Singapore, 117543 Singapore; 5 Department of Biological Sciences, University of Alberta, Edmonton, T6G 2E9 Canada; 6 Department of Medicine, University of Alberta, Edmonton, T6G 2E1 Canada; 7 BGI-Shenzhen, Shenzhen, 518083 China; 8 Botanical Institute, Cologne Biocenter, University of Cologne, 50674 Cologne, Germany

**Keywords:** Enzymes, Computational biology and bioinformatics, Molecular evolution

## Abstract

Hidden Markov models representing 167 protein sequence families were used to infer the presence or absence of homologs within the transcriptomes of 183 algal species/strains. Statistical analyses of the distribution of HMM hits across major clades of algae, or at branch points on the phylogenetic tree of 98 chlorophytes, confirmed and extended known cases of metabolic loss and gain, most notably the loss of the mevalonate pathway for terpenoid synthesis in green algae but not, as we show here, in the streptophyte algae. Evidence for novel events was found as well, most remarkably in the recurrent and coordinated gain or loss of enzymes for the glyoxylate shunt. We find, as well, a curious pattern of retention (or re-gain) of HMG-CoA synthase in chlorophytes that have otherwise lost the mevalonate pathway, suggesting a novel, co-opted function for this enzyme in select lineages. Finally, we find striking, phylogenetically linked distributions of coding sequences for three pathways that synthesize the major membrane lipid phosphatidylcholine, and a complementary phylogenetic distribution pattern for the non-phospholipid DGTS (diacyl-glyceryl-trimethylhomoserine). Mass spectrometric analysis of lipids from 25 species was used to validate the inference of DGTS synthesis from sequence data.

## Introduction

Photosynthetic eukaryotes arose from the endosymbiosis of a photosynthetic bacterium by a eukaryotic cell^1^. The descendants of this endosymbiotic event, with the exception of the land plants (Embryophyta), are all referred to as ‘algae’. The algae include the Glaucophyta (a small group of freshwater species that have features thought to resemble those of the common ancestor), the Rhodophyta (‘red algae’, some of which are unicellular and others, complex seaweeds), and the ‘green algae’. The ‘green algae’ are essentially the Viridiplantae (“green plants”) that are not embryophytes (land plants). They are extraordinarily diverse organisms, and by definition consist of lineages that are as old or older than the land plants. They consist of multicellular seaweeds (Ulvophyceae) as well as numerous classes of mostly unicellular organisms, Chlorophyceae and Trebouxiophyceae being the two largest groups. The green algae also include prasinophytes, a paraphyletic group that includes several classes of mostly planktonic marine species. Together, all of these groups are considered to be members of the Chlorophyta. The green algae also include the streptophyte algae, a paraphyletic group, that, together with embryophytes, form the sister group to the Chlorophyta.

Most algae are unicellular, but many are not; indeed, multicellularity evolved several times in this group^2^. Given this diversity of algal morphology, and the presence of species in nearly every imaginable ecological niche, it is reasonable to suppose that there have been interesting adaptations in metabolism. The potential for metabolic novelty among the algae is of particular interest because these organisms are poised to be of increasing importance to biotechnology^3^.

The transcripts that are encoded by an organism can be sampled by the sequencing of cDNA fragments. The assembled transcriptome that results is necessarily incomplete, for two reasons. First, the abundance of different transcripts is highly skewed: a relatively small number of transcripts are highly abundant, but many more are expressed at low levels, vanishingly low even. Deeper sequencing can mitigate this problem, but a complete transcriptome can only be approached asymptotically, with diminishing returns of new transcripts. A second problem is that many transcripts might not be expressed at all except under particular conditions or (in multicellular organisms) in only one kind of cell. The completeness of the transcriptome can be improved by sequencing RNA from more than one cell type, or from more than one environmental condition, but biologically relevant transcripts will almost certainly remain undetected.

The flip side of this problem of having skewed transcript abundances is that it is relatively easy and inexpensive to get a large number of moderately well-expressed transcripts. In addition, nearly all of the sequence obtained is protein coding. The high density of functional sequences, and the ease with which many thousands of transcripts can be detected means that a relatively large amount of information can be obtained inexpensively. The inference of function for those sequences can often be done rather reliably based on sequence similarity. Especially powerful in this regard is the use of sequence profiles for protein sequence families, for which some family members have known functions. Such profiles can be obtained from curated libraries (such as the PFAM library of hidden Markov models (HMMs)) or created from protein sequence alignments, such as those found in the KEGG database of metabolic pathways and their associated enzymes^4,5^.

Although the highly skewed nature of transcript abundances precludes the possibility of completeness, it also contributes to the cost-effectiveness of sequencing by ensuring that a great many sequences can be determined through quite shallow sequencing. This also means that many strains and species can be sampled, which brings greater power to statistical analyses. Even though an individual transcriptome may lack sequences due to incompleteness, the existence of sufficiently large sample sizes can allow us to reach conclusions about the likely presence or absence of transcripts. In particular, we can ask whether there are clades in which a transcript is absent more often than expected by chance, relative to the frequency with which it is found in other clades. In addition, by considering the multiple enzyme activities that are typically associated with a particular metabolic pathway, probabilistic inferences can be drawn about the presence of a pathway that do not require every constituent of the pathway to be detected.

Here we describe a survey of selected metabolic functions from 183 diverse algal species/strains. The presence or absence of pathways was inferred by scoring each transcriptome for the existence of a homolog to 167 protein sequence profiles, selected for their relevance to pathways of interest, and then analyzing the distribution of hits among clusters of related species. This allowed us to identify pathways that appear to have been gained or lost along different evolutionary branches.

The algae that were examined include members of each of the major groups described above, all of which are direct descendants of the founding endosymbiotic event, In addition, there are other, more complex lineages of algae that acquired their plastids through secondary endosymbiosis, such that the endosymbiont in these cases was a photosynthetic eukaryote, not a bacterium^6^. A set of these organisms, which we refer to in the aggregate as Chromista, was also included in our analysis.

## Results

### Transcriptome sequences

For the analysis described here, we obtained *de novo* assembled transcriptomes from the 1000 Plant Transcriptome Project (OneKP). OneKP was created several years ago in order to obtain protein coding information from a phylogenetically broad collection of plants and algae^7^. RNA from more than a thousand different species, contributed by dozens of laboratories, has been sequenced as part of this project; sequencing and transcriptome assembly has all been done by BGI-Shenzhen.

### Phylogenetic relationships

As part of the analysis performed by the OneKP consortium, more than 1,000 plant and algal samples were assembled into a phylogenetic tree using Astral^8^, which is under review as part of the OneKP consortium’s capstone paper. Our analysis relies strictly on this phylogeny, determined independently by the OneKP consortium. However, using functions in the *ape* and *dendextend* packages of R, we did select from the OneKP tree the subset of transcriptomes relevant to our analysis, re-rooted the tree on the Chromista, rotated around nodes for presentation purposes, and cut the tree at different levels as part of our statistical analyses.

**Figure 1:**
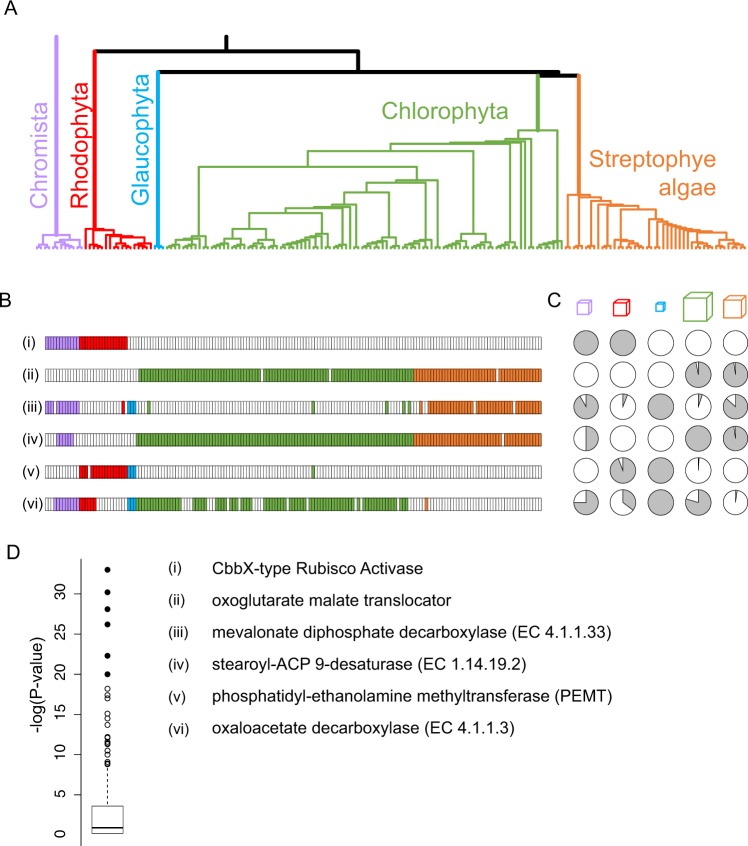
Skewed distributions of transcripts among major algal clades. (**A**) Phylogenetic tree for 169 algal species or strains. This is a sub-tree of one produced by the OneKP project, restricted to those transcriptomes for which we conducted HMM searches (Methods). Cutting the tree at a high level results in five clades; the identification of these as Chromista, Rhodophyta, Glaucophyta, Chlorophyta, and Charophyta is based on the taxonomic information that the OneKP project has associated with each sample. (**B**) Presence (filled boxes) or absence (empty) of HMM hits for six HMMs (numbered i to vi). Each box corresponds to a transcriptome, ordered from left to right as in the tree. Colors of the boxes correspond to the color-coding of the clades in panel A. (**C**) Fraction of transcriptomes with a hit to the HMM in of each of the five clades. Cubes above the pie-charts have volumes that are to scale with respect to the number of samples in each clade. (**D**) P-values (Fisher's exact test) for non-random distribution of HMM hits among the five major clades. The top six are shown as filled circles, and correspond to the six HMMs whose distributions are shown in panels B and C. The names of the enzymes are shown.

As is clear from the tree, the algal samples we analyzed fall into five principal groups, albeit ones of very different sizes. These groups correspond closely to conventional taxonomic groups, as inferred from the names given to the samples by those who provided the samples. Twelve of the samples (labeled Chromista in Fig. 1A) constitute the outgroup. Unlike all the other samples analyzed, they are not direct descendants of the primary endosymbiotic event. Instead they are descendants of subsequent endosymbiotic events involving either a red algal cell (forming a secondary endosymbiont) or a previously created Chromista lineage (tertiary or even quaternary endosymbiosis). The gene complement of these organisms have complex histories, but chloroplast genes, and some nuclear genes transferred to the host, bear the mark of their common ancestry with the rhodophytes. We have left this clade unattached to the rest of the tree as a reminder of the complex history. The rhodophytes themselves are represented by 17 samples, and a total of six different classes; their divergence from the green lineages is the earliest split among the direct descendants of the primary endosymbiont. The next major lineage on the tree, the Glaucophyta, is represented here by only three samples. Although small in number, the lineage is an important group for understanding the evolutionary history of the Viridiplantae as it is sister to them. The largest groups in the tree share a common ancestor more recently than the rest, uniting them as ‘green algae’. One clade corresponds to the Chlorophyta, while the second group corresponds to the streptophyte algae, the group from within which the Embryophyta arose.

We were interested in understanding how these five major groups of algae, and indeed smaller sub-groups as well, differ in their physiology and metabolism. To that end, we assembled a set of hidden Markov models representing 167 protein sequence alignments and used these to infer functions for some of the 8.5 million transcripts in our dataset. Although our primary motivation is an investigation of evolutionary gains and losses in metabolic function, we chose to focus on functions that might be relevant to the practical applications of algae and to the selection of particular organisms for experimental study. Therefore, our largest group of HMMs relate to lipids, and, more broadly, to carbon metabolism (Supplementary Table S2). Eleven of the HMMs we classify as being related to carbon assimilation and a further 25 in central carbon metabolism. A larger group of 56 HMMs are related directly to membrane lipids or triacylglycerides. Subgroups of these relate more specifically to fatty acid synthesis, glycerol backbone metabolism, or the synthesis of membrane lipid headgroups. In addition to these fatty-acid based lipids, there are 11 HMMs related to terpenoids (isoprene synthesis or the utilization of isoprene units).

The second largest group of functions we targeted are those associated with vitamin synthesis and utilization. Vitamin synthesis and utilization is of interest from an evolutionary perspective because B12-independence, at least, appears to have has arisen multiple times in algal lineages. It may be of practical value, as well, in considering growth requirements for different species. In total, we looked for matches to 40 HMMs that are relevant to vitamins B12, B6, biotin, and thiamine.

Rounding out the functions we examined, 17 of the HMMs (~10% of the total) were for miscellaneous sequences that may be of relevance in guiding the selection of species for experimental work. These include, principally, functions that are related to RNA silencing or meiosis, the latter demonstrating the existence of sexual reproduction in the species. While the absence of transcripts for these functions is likely, in most cases, to cells being in the wrong developmental state, where transcripts are present they could be helpful in deciding which strains to avoid (in the case of active silencing mechanisms) or favor (in the case of sexual reproduction, in the event classical genetics is desired). These data are not discussed further but are provided as a resource as part of Supplementary Table S2 and Supplementary Table S3.

### Skewed distributions of transcript sequences, and metabolic function, across the major clades

Figure 1B shows, for each of six HMMs, the samples that have transcripts matching those HMMs (filled boxes) and those for which no hit was found (empty boxes). Among all of the HMMs tested, these are six with the most biased distribution among the five major groups in our analysis. The bias is obvious from visual inspection, as is the diversity of ways in which sequences are skewed across the dataset (Fig. 1C). An assessment of statistical significance was made using Fisher's exact test, under the null hypothesis that HMM hits are distributed randomly (Fig. 1D; Supplementary Table S2). Unsurprisingly, the distribution of p-values shows that many sequences are evolutionarily skewed; the six that are highlighted here have p-values less than 10^−20^. We review each of these briefly to illustrate the kinds of evolutionary history that might be inferred from these distributions.

At the top of the list is the CbbX-type Rubisco activase (P < 10^−34^). Rubisco activase has been found in every organism that uses Rubisco (Ribulose 1,5-bisphosphate carboxylase) because the latter enzyme is prone to forming an inactive complex with its substrates and related compounds. Rubisco activase dissociates these inactive complexes, re-creating an active Rubisco. All known Rubisco activases are members of the AAA + superfamily of ATP-driven molecular machines^9^. The activases found in red algae are derived from the cbbX gene of phototrophic proteobacteria (‘purple bacteria’), and hence are known as CbbX-type activases, while the activases in green algae and plants are derived from cyanobacteria^10^. The two activase families are different enough that it is possible they actually converged on this function independently from other AAA + activities, rather than diverging from a shared Rubisco activase ancestor^10^. In our analysis, all 17 rhodophyte transcriptomes were positive for CbbX-type Rubisco activase, as were all 12 of the Chromista transcriptomes. The Chromista lineages have CbbX-type activase because they acquired their photosynthetic machinery through endosymbiosis of rhodophytes. None of the other 146 samples have CbbX-type activase.

This result is a demonstration, not a discovery, as it was already known that the red and green lineages differ in the evolutionary history of their Rubisco activases. This search also differed from the rest of the searches conducted in this paper in that we used the ratio of two HMM scores to define a hit (Methods). In all other search reported here, we used a 10-bit cutoff on a single HMM. We defer to the Discussion section an explanation for this, and a more general discussion about the for sensitivity and specificity of sequence profile searches.

Our second example is that of an oxoglutarate-malate translocator, which, in spinach, is localized to a chloroplast membrane^11^. Sequences for this enzyme were completely absent in the Chromista, glaucophyte, and rhodophyte samples, but were found in nearly every one of the samples from the green lineages (95/98 of the chlorophytes and 43/45 of the streptophyte algae). A PSI-BLAST search of the NCBI nr database found no hits outside of the Viridiplantae, confirming that the enzyme is unique to this group of organisms.

The third most biased distribution is that of EC:4.1.1.33, mevalonate diphosphate decarboxylase (p = 10^−28^). This enzyme catalyzes the final step in the mevalonate pathway for terpenoid synthesis. Homologous transcripts are nearly absent from the rhodophytes (1/16), and from the chlorophytes (5/98) indicating that, with possible rare exceptions (or perhaps contaminants of the transcriptome samples), the metabolic pathway is absent in these clades. In contrast, homologous transcripts were found in nearly all of the streptophyte algae (40/44) and in all three glaucophytes. These results extend earlier observations regarding the evolutionary history of the mevalonate pathway^12–14^. The most parsimonious explanation for the distribution of sequences is that it was lost independently in the rhodophytes and in the Chlorophyta.

Next is a fatty acid desaturase (stearoyl-ACP 9-desaurase; EC:1.14.19.2). Like the oxoglutarate-malate translocator (second on the list), this fatty acid desaturase is localized to the chloroplast, absent from the rhodophyte and glaucophyte transcriptomes, and present in nearly all of the green lineage transcriptomes. Among the Chromista, the six samples with the sequence form a monophyletic clade dominated by strains that are considered haptophytes. All of the strains outside of this clade are cryptophytes or ochrophytes (Supplementary Figure S2).

The fifth most biased distribution is that of phosphatidyl-ethanolamine methyltransferase (PEMT), a step in one of several pathways that lead to the synthesis of the membrane lipid phosphatidylcholine (PC). PEMT It is found in all of the rhodophytes and glaucophytes (with one exception, likely to be a sampling issue) suggesting that it was present in the common ancestor of the Archaeplastida. In contrast, it appears to be entirely absent from the green lineages, suggesting that it was missing in the common ancestor of the Chlorophyta, the streptophyte algae, and the embryophytes. (The one hit that was found to a chlorophyte transcriptome, IJMT, is suspect because the transcriptome has a number of incongruous transcripts, and indeed the culture was subsequently found to be contaminated with an amoeba).

Our final example is that of oxaloacetate decarboxylase (EC 4.1.1.3) The energetically favorable decarboxylation of oxaloacetate to pyruvate is coupled to the establishment of a Na^+^ ion gradient, at least in the bacterial species in which it has been most carefully studied^15^. What makes the distribution of homologous sequences so different in this case is that it appears there has been a loss of the gene in particular lineages. The sequence is found in all three glaucophytes and in most chlorophytes, but is absent among the streptophyte algae except for in *Mesostigma viride* (KYIO). As perhaps the earliest diverging species in the group, it might have retained the sequence. More work will be required to confirm the origin of this sequence Among rhodophytes and Chromista, the distribution of oxaloacetate decarboxylase sequences is more complex, but inspection of the phylogenetic tree shows that the pattern of hits in these groups reflects their evolutionary history. Within the Chromista, the haptophytes and ochrophytes all have the sequence, while the species missing the sequence are all cryptophytes (Supplementary Figure S2). Similarly, among the rhodophytes, the six species that have a homologous transcript represent five different classes, while all eleven members of the class Florideophyceae are missing the sequence, suggesting loss of the gene in the ancestor of the Florideophyceae. This striking concordance of taxonomy with the presence or absence of homologs lends credence to both the phylogeny, and to the sequence coverage of the transcriptomes. Even the absence of the sequence in some chlorophytes looks like much of it is due to the evolutionary loss of the gene or its expression, rather than sampling issues: of the twenty chlorophytes lacking a homologous sequence, eleven occur in just three clusters, of size four, four, and three (discussed further below).

### Gains and losses on evolutionary branches within the chlorophyta

The loss of a gene in an ancestral species almost invariably results in its absence in the species that are descended from it; only when a related sequence is regained, through horizontal transfer or, less likely, convergence, will a lost gene be detected in a descendant species. Similarly, gain of function in an ancestral species means the function will be present in its descendants unless there was a subsequent loss. The fact that sequences can have evolutionary histories of this kind is precisely why the distribution of sequences among extant groups can be expected to be non-random. Conversely, the existence of a biased distribution among extant species is itself evidence for the loss or gain of function in an ancestral species.

The skewed distributions we have examined thus far are at a coarse level, with all species falling into just five, high-level groups. In addition, the groups contain dramatically different numbers of samples and one them, the Chromista, does not have a simple binary-branching relationship with any of the other groups, its members having arisen from secondary endosymbiotic events. For the remainder of our analysis, therefore, we turned our focus to the largest of the five groups, the 98 chlorophytes, and looked for gains and losses of sequences along branches of that tree. To simplify the analysis and its presentation, we first eliminated from the chlorophyte tree any nodes for which further branching failed to increase the confidence with which we could say that any of the sequences under study was skewed in its distribution. (see Methods). In practice this meant eliminating branches with a single sequence (i.e. terminal nodes) and truncating the tree in a way that left it with 13 terminal groups, with sizes ranging from two samples to thirteen.

**Figure 2:**
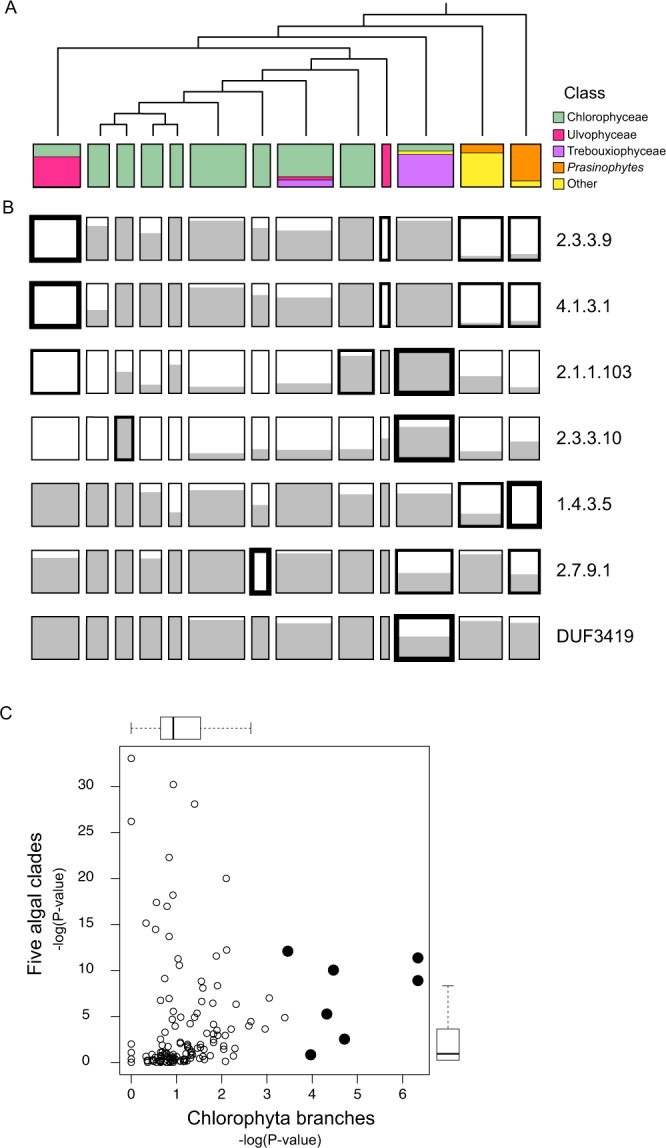
Skewed distributions of transcripts within the Chlorophyta. (**A**) Simplified phylogenetic tree for 98 Chlorophyta samples (Methods). The width of the boxes corresponds to the number of clustered transcriptomes, which range from 2 to 13. Samples were assigned to a taxonomic class based on the classification system used by AlgaeBase and the lower level taxonomic names provided by OneKP. A majority of the samples belong to the class *Chlorophyceae*. Two other classes singled out for explicit representation are *Ulvophyceae* and *Trebouxiophycea*. The term prasinophytes here represents the classes *Nephroselmidophyceae*, *Mamiellophyceae*, and *Pyramimonadophyceae*. (**B**) Distribution of homologous transcripts for the seven HMMs whose distributions within the chlorophytes is most significantly different than random. The gray fill in each box indicates the fraction of transcriptomes in that cluster that have hits to the indicated HMM. At each branch point, descending from the top of the tree, the skew in the distribution of samples with HMM hits was assessed with Fisher's exact test. Boxes with thick lines indicate clusters with a p-value less than 0.001. The lowest p-value for each HMM is indicated by the single thickest line in that row. 2.3.3.9: malate synthase; 4.1.3.1: isocitrate lyase; 2.1.1.103: phosphoethanolamine methyltransferase; 2.3.3.10: HMG-CoA synthase; 1.4.3.5: pyridoxamine phosphate oxidase; 2.7.9.1: pyruvate-phosphate dikinase; DUF3419: Unknown Function according to Pfam function, but associated here with DGTS synthase. (**C**) Distribution of p-values for HMM-hit distributions. Each point is an HMM. The y-axis shows the p-value for the distribution across the five major clades; this is the same as shown in Fig. 1. The x-axis shows, for each HMM, the lowest of the p-values for splits along the Chlorophyta tree. Filled circles represent the HMMs shown in panel B. From right to left, these correspond to the schematics in panel B, from top to bottom.

The resulting tree is shown at the top of Fig. 2A. The membership of the clusters by class is indicated by color. This assignment of samples to classes was done according to AlgaeBase, starting from the Order and/or Family names associated with the samples at the OneKP web site (current as of December 17, 2018). The OneKP taxonomic labels themselves are based on the expertise of those providing samples or are derived from the names in the strain collections from which samples were obtained. Except in rare cases, the taxonomic names used to assign samples to classes are independent of the OneKP data itself.

Figure 2 also shows the seven sequences whose distribution among the chlorophytes is most highly skewed by this criterion. These are different from the six HMMs discussed above, which are the most highly skewed across the five major clades (Fig. 1). However, most of these new HMMs are also highly skewed across the major clades, with five having p-values better than 10^−5^. (Fig. 2C). Thus, the gain or loss of these sequences is, in most cases, associated with the evolutionary history of the algae more broadly, and not just within the chlorophyte sub-trees in which their skewed distribution is most significant.

### Repeated loss of the glyoxylate shunt

**Figure 3:**
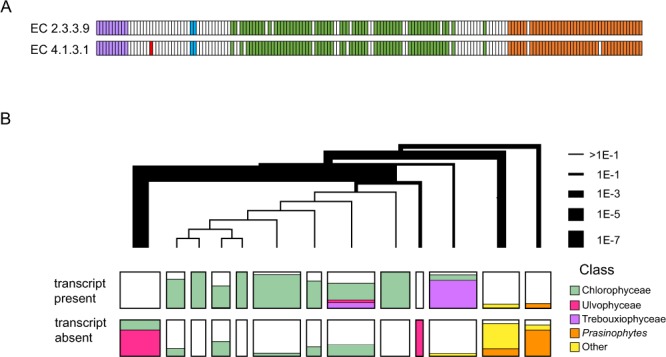
Distribution of glyoxylate shunt enzymes shows a preferential absence of both sequences in certain clades. (**A**) Hits to glyoxylate shunt HMMs across the full set of transcriptomes. EC:2.3.3.9: malate synthase. EC:4.1.3.1: isocitrate lyase. (**B**) Chlorophyta tree with branchpoint thicknesses related to the p-value associated with the segregation of HMM-positive and HMM-negative transcriptomes. Within each cluster, the fraction of transcriptomes with and without hits to the HMM are indicated, as well as the taxonomic Class (or group of Classes) to which the samples belong.

A good example to start with is the first pair of pair of enzymes shown in Fig. 2B, EC:2.3.3.9 (malate synthase) and EC:4.1.3.1 (isocitrate lyase). These two enzymes together constitute a pathway, known as the glyoxylate shunt, that allows organisms to use certain two carbon compounds as a carbon source, building up molecules through the synthesis of malate (4-carbons) from two acetyl-CoAs. It is clear from Fig. 2B that the distribution of transcripts for these enzymes is very similar across the chlorophytes, which makes sense: both enzymes are required for a functional glyoxylate shunt and there is no other known function that would maintain selection for one or the other. As shown in Fig. 3, there is in fact an almost perfect correspondence of hits at the resolution of individual samples across the entire dataset, not just the chlorophytes. Of the 175 transcriptomes, 110 have both malate synthase and isocitrate lyase, 56 have neither, and only 9 have one but not the other. The degree of concordance is highly unlikely to be due to chance (P < 10^−15^).

The most striking feature of the distributions across the full dataset is the near absence of homologs among the rhodophytes. Of the 17 rhodophyte samples, none has a malate synthase homolog and just one (ZUIJ, *Porphyra yezoensis*) has isocitrate lyase. In contrast, homologous transcripts for both enzymes are found in two of the three glaucophytes, most of the chlorophytes, and nearly all the streptophyte algae. This pattern could be due to a gain of function in the green lineage, or to a loss in rhodophytes, but losses of the pathway certainly seem to have been common within the chlorophytes. (Fig. 3) In particular, transcriptomes that lack homologs are disproportionately found among the ulvophytes and prasinophytes, and in a small group of other classes. In contrast, most of the Chlorophyceae and the Trebouxiophyceae retain these sequences. The groups that have preferentially lost the glyoxylate shunt transcripts diverged early from the rest of the chlorophytes, but did so at different times because these classes of algae do not, together, constitute a monophyletic group. The implication is that these genes were lost on multiple occasions in the evolution of the chlorophytes, and perhaps as well on the rhodophyte lineage.

The absences of transcripts could reflect gene regulatory differences rather than gene loss. Perhaps those species that seem to lack the glyoxylate shunt simply do not express the genes under the conditions used to prepare the RNA. To check whether genes for these enzymes have actually been lost in species like those for which we see this pattern, we searched representative genomes for which annotations exist in KEGG (Methods). As a kind of control, we first surveyed genomes from a small number of randomly selected animals, plants and fungi, using enzyme names and EC numbers as search words: in all cases, the plants and fungi had both isocitrate lyase and malate synthase, and in all cases the animals had neither, as expected. Doing the same for the fourteen algal genomes represented yielded both enzymes in four of the fourteen genomes, plus, in one more, a hit to malate synthase, only. All five of these genomes are from species belonging to the classes Chlorophyceae or Trebouxiophyceae. These are the same classes from which, in our analysis, we found most samples to have the sequences. In contrast, none of the three rhodophyte genomes and none of the five prasinophytes genome represented in KEGG appear to have either of the two genes. This again is consistent with the transcriptome analysis. We conclude that the distribution of glyoxylate shunt transcripts in the OneKP data set is a good proxy for the existence or absence of their genes.

The reason for the frequent loss of the glyoxylate shunt enzymes is unclear, but it may be relevant that the groups for which absence of the sequences is most striking – the rhodophytes, ulvophytes, and prasinophytes – are generally marine species. Most other species prefer fresh or brackish water or are subaerial/terrestrial. Perhaps the marine environment has a paucity of two-carbon metabolites that would provide a selective advantage for retention of the glyoxylate shunt. A similar loss of selection in marine environments might also explain the near-total absence of sequences for pyridoxal-5'-phosphate synthase (EC 1.4.3.5) in prasinophytes. There is a single exception to the rule that prasinophyte samples lack this sequence (MMKU), and, perhaps not coincidentally, the organism from which the RNA came (*Nephroselmis olivacea*) is one of only two prasinophytes in our dataset that is a freshwater species, rather than marine, according to Algaebase.

### Isoprene synthesis and the possible co-option of HMG-CoA synthase

Isoprenyl-diphosphate is a five-carbon, branched molecule that is the building block for a host of isoprenoids, including cholesterol and related molecules, photo-absorbing pigments such as carotenoids, and a broad array of secondary metabolites. There are two distinct pathways for synthesizing isoprene units: the mevalonic acid pathway (MVA) and the non-mevalonate or MEP/DOXP pathway, named for a pair of intermediates^16^. The MVA pathway is found in animals, fungi, and archaea, while the MEP/DOXP pathway is found in nearly all bacteria. Both pathways are found in most plants: MVA functions in the cytosol while the MEP/DOXP pathway is localized to the chloroplast. As its localization suggests, the MEP/DOXP pathway was inherited from the cyanobacterial endosymbiont that founded the algal/plant lineages.

**Figure 4:**
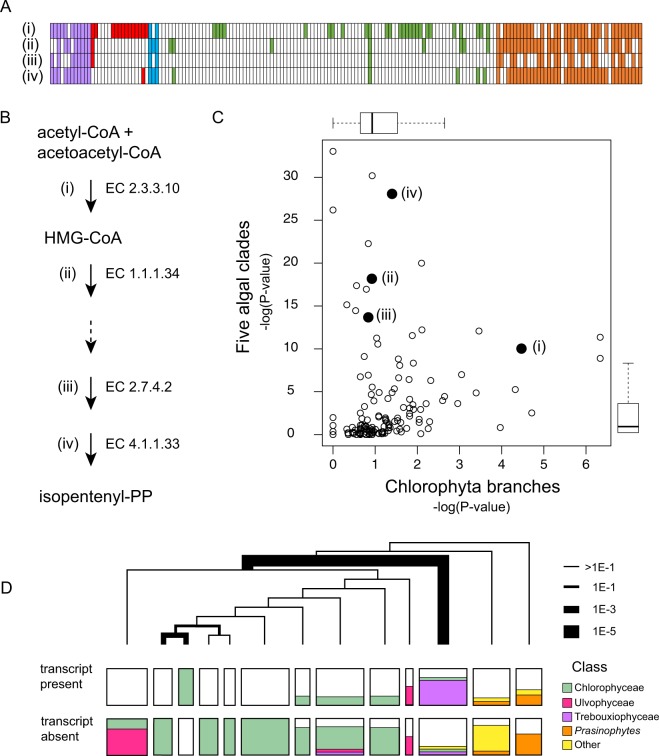
Distribution of sequences in the mevalonate (MVA) pathway for isoprenoid synthesis, and the possible co-option of HMG-CoA synthase. (**A**) Hits to four enzymes in the mevalonate pathway. Numbers correspond to the EC codes in panel B. (**B**) Schematic of the pathway indicating the production of HMG-CoA by hydroxymethylglutaryl-CoA synthase (EC:2.3.3.10) and the ultimate production of an isoprenoid monomer. EC 1.1.134: HmG-CoA reductase; EC 2.7.4.2: phosphomevalonate kinase; EC 4.1.1.33: diphosphomevalonate decarboxylase. The dashed arrow denotes an enzyme for which we were unable to characterize the presence of transcripts because we lacked a sufficiently specific HMM. (**C**) Distribution of p-values as in Fig. 2C. Transcripts in the MVA pathway are labeled and indicated by large filled circles. (**D**) Distribution of hits to the HMM for HMG-CoA synthase (EC:2.3.3.10). The tree and the boxes denoting HMM hits in each cluster are as described in Fig. 3.

While both pathways persist in plants, having the MEP/DOXP pathway as a kind of back-up system has evidently allowed the loss of the MVA pathway in many algae. (12–14) Fig. 4 shows the distribution of transcripts for four enzymes in the pathway. (A fifth enzyme in the pathway, mevalonate kinase, EC:2.7.1.36, is not shown, and is indicated by a dashed line (Fig. 4B) because its HMM is non-specific, picking up galactokinase homologs as well as mevalonate kinases). Taking into account the incomplete nature of transcriptomes, it would appear that the Chromista, the glaucophytes, and the streptophyte algae have retained the MVA pathway, while the chlorophytes and rhodophytes have lost it. There may be one exception to this rule among the rhodophytes. Sample LLXJ (*Chroodactylon ornatum*) has three of the four transcripts we looked for, seemingly missing only the last step in the pathway. It has recently been suggested that species in the rhodophyte class Stylonematophyceae may have retained a functional MVA pathway, and LLXJ is the only sample we have from the class Stylonematophyceae. If this species does retain the MVA pathway, perhaps the last sequence is missing due to insufficient depth of sequencing.

Many of the rhodophyte and chlorophyte samples, although otherwise lacking evidence for an MVA pathway, have retained sequences that appear to encode the first enzyme in the pathway, HMG-CoA synthase. The presence of an HMG-CoA synthase sequence in the unicellular rhodophyte *Cyanidioschyzon merolae* has been noted before^17^, but our analysis shows this to be a very common feature of rhodophytes. It is not quite as common in chlorophytes, but Fig. 4C (and Fig. 2B as well) shows that it does occur in this group as well, and that its distribution is highly non-random. The nature of the distribution strongly implies that it is a consequence of evolutionary history rather than random sampling noise. Figure 4D shows that much of the bias in the distribution is due to samples of the class Trebouxiophyceae having the sequence. A second, smaller component to the skewed distribution is a cluster of four Chlorophyceae, all of whose members have a homolog, something that is not true for any of their 26 closest relatives.

It is not clear what evolutionary explanation might exist for so many Trebouxiophyceae and Rhodophyta to have HMG-CoA synthase, or for that matter, any of the other species that seem to have it, including the curious cluster of four Chlorophyceae. The widespread absence of other enzymes in the pathway, and the biochemical evidence consistent with the loss of this pathway in red and green algae, suggests that HMG-CoA synthase activity has been co-opted for some other pathway, or the enzyme has diverged in specificity for some other purpose.

### Membrane lipid synthesis: pathways to phosphatidylcholine and the non-phosphate containing lipid DGTS

**Figure 5:**
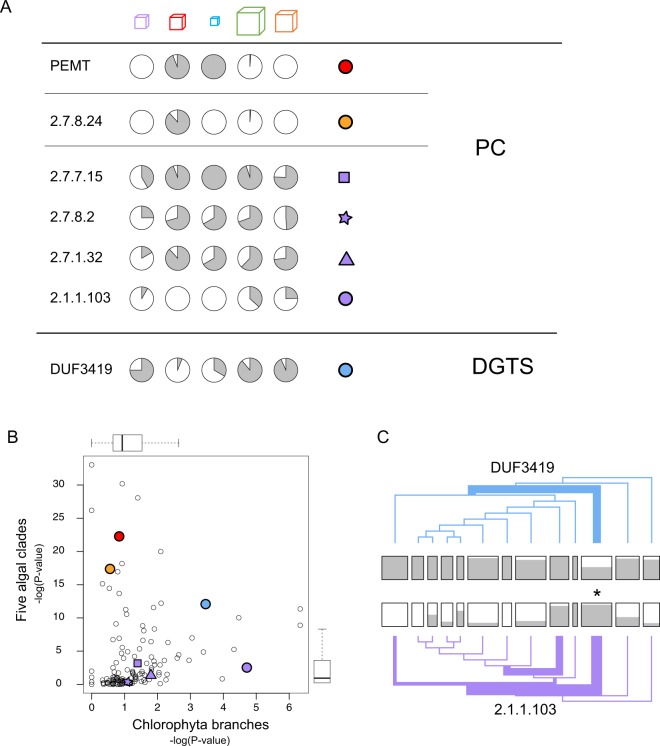
Distribution of enzyme sequences for the synthesis of PC and DGTS. (**A**) Distribution of HMM hits for seven enzymes discussed in the text. The first two are involved in two separate pathways for PC. The third and fourth complete a third pathway, while the fifth and sixth are two different ways of creating the starting point for that third pathway. The last HMM is for an enzyme involved in DGTS synthesis. The colored symbols are a key to panel B. (**B**) P-values for the distribution of membrane lipid HMM hits. Discussion focuses on those indicated by the colored circles. (**C**) Chlorophyta trees for DUF3419 (blue) and 2.1.1.103 (purple). Differences in line thickness at different branchpoints are related to the p-value for skewed distributions of HMM hits. Note that both HMMs are most biased at the same branchpoint: DUF3419 has unusually few hits in one of the clusters and 2.1.1.103 has unusually many, both indicated by the asterisk between the two.

Our final example involves the synthesis of membrane lipids, specifically the phospholipid phosphatidylcholine (PC) and the betaine lipid diacylglycerol-trimethyl-homoserine (DGTS). PC is a widespread membrane lipid, with at least three distinct pathways for its synthesis. Choline is the common name for tri-methyl ethanolamine, and the simplest way to make PC is to methylate PE (phosphatidyl-ethanolamine), another common membrane lipid. This reaction is catalyzed by phosphatidyl ethanolamine methyltransferase (PEMT), which was discussed earlier in the context of Fig. 1 because it is among the most highly skewed sequences across our five major clades. The distribution of homologs to PEMT is also shown in Fig. 5, as are hits to the other HMMs related to membrane lipid synthesis, described below.

A second pathway transfers choline directly onto an activated form of diacylglycerol; this reaction is catalyzed by an enzyme with the EC number 2.7.8.24. The final pathway starts with phosphorylated choline, proceeds to further activate the choline by hydrolysis of CTP (resulting in choline-CDP), and then transfers the choline to diacylglycerol. These two reactions are catalyzed by EC:2.7.7.15 and EC:2.7.8.2. respectively. This pathway is actually more like a pair of pathways because there are two routes to phospho-choline: choline can be phosphorylated (EC 2.7.1.32) or phosphor-ethanolamine can be methylated (EC 2.1.1.103). All six of the enzymes we have mentioned as being involved in a PC-synthesis pathway are represented in Fig. 5.

In addition to PC we also consider the lipid DGTS. DGTS is similar to PC in that both are zwitterionic, with a positively charged tri-methylated amine at the end of the headgroup and a negatively charged moiety between the amine and glycerol backbone. However, the negatively charged group in DGTS is not a phosphate, as it is in phospholipids like PC. DGTS can therefore substitute for PC in environments that are phosphorus-limited, and probably represents an adaptation for just that purpose.

As shown in Fig. 5, the first two pathways for PC that we discussed have highly skewed distributions. PEMT is essentially found only in the rhodophytes and glaucophytes, whereas 2.7.8.24 is essentially found only in the rhodophytes. If we skip to DGTS, we see that the pattern is almost the reverse. Nearly all the chlorophytes and algal streptophytes have sequences that imply DGTS synthesis, but almost none of the Rhodophyta do. Thus, ignoring the third pathway to PC for the moment, it seems that there is a divide between the red and green algae, with the green adopting DGTS and the red PC.

It is more complicated than that, though, because enzymes for the third PC pathway are, for the most part, found at high frequency in all of the clades. Thus, although most green algae lack both of the first two pathways for PC synthesis, and generally do have a sequence that implies DGTS synthesis, they also have transcripts that suggest they can produce PC through the third pathway. The fact that many green algae produce both DGTS and PC is something we confirmed experimentally in the course of assessing the DUF3419 HMM as a marker for DGTS synthesis. (Methods). Interestingly, though, there is one exception to this wide-spread coverage of the enzymes involved in the third pathway, and that is for EC:2.1.1.103. This enzyme, as discussed above, catalyzes one of two alternative routes to the synthesis of phospho-choline, the substrate for the remaining portion of this pathway to PC. As an alternative route to the starting material, EC:2.1.1.103 is not absolutely required, and indeed it seems less widely used than EC:2.7.1.32. What is most notable about the transcripts for this enzyme, though, is their highly biased distribution among the Chlorophyta. It is, in fact, the third most highly biased of the 167 HMMs we examined, behind only the two glyoxylate shunt enzymes. As shown in Fig. 5C, there are a couple of clusters of chlorophytes that tend to have this sequence, while other, less closely related species, tend not to have it. Fig. 5C also shows the transcript distribution for DUF3419, which itself has the seventh most highly biased distribution among the 167 HMMS. Nearly all chlorophytes have a DUF3419 sequence, so what makes the distribution of transcripts so unusual for this HMM is that there is a single cluster of 13 species in which about half of the members are missing the sequence. Intriguingly, this cluster is one that is enriched for EC:2.1.1.103 sequences. It is tempting to think that for these algae, which are mostly Trebouxiophyceae, the alternative route to PC synthesis has obviated the need for DGTS synthesis.

## Discussion

We have employed here the power of *de novo* assembled transcriptomes to reveal gains and losses of function along evolutionary trajectories. Similar kinds of analyses can, of course, be done with genome sequences^18–20^., which have the considerable advantage of near-completeness. Why bother, then, with transcriptomes? There are three reasons. First, transcriptomes have a much higher density of protein coding sequence than do eukaryotic genomes, which typically contain lots of introns and long intergenic sequence. Transcriptome sequencing is thus a cheaper route to obtaining a large fraction of an organism's protein coding sequence. Second, the fact that transcriptomes lack introns, and are less likely to include pseudogenes, makes it easier to infer function from sequence. Third, the fact that transcriptomes are cheaper and easier to analyze means we can obtain more of them, bringing to bear greater statistical power in the downstream analysis. Even with the cost of sequencing as low as it is, it would be expensive to obtain (and difficult to analyze in sufficient detail) genome sequences for all 175 species we have used here.

Notwithstanding the advantages of transcriptomes, there are two issues with their incompleteness that we need to address, one fairly trivial, the other important. The first is the sampling effect that results in some transcriptomes lacking a sequence purely by chance. This is the most likely explanation, for example, for the one charophyte and three chlorophytes for which we did not find a hit to the HMM for an oxoglutarate-malate translocator (Fig. 1) The absence of the sequence in the three chlorophytes is not due to a shared evolutionary event because the species are not closely related. The missing sequences are also not due to these samples being sequenced less deeply than the others; in fact, the number of scaffolds for these samples is entirely typical, two being above average and two below. While it is possible that the gene has been lost independently in four species, it is more likely that it is present in all 135 chlorophytes and charophytes but that its expression level is such that it just happens to be have been missed in four of the samples.

The incompleteness of transcriptomes limits our ability to detect authentically skewed distributions. Deeper sequencing can improve sensitivity to detect evolutionary gains and losses, but it is a very inefficient means of doing so because of the highly skewed nature of transcript abundances and because statistical power is, in any case, limited by sample size. A more efficient approach is to simply increase the number of organisms sequenced, Large sample sizes allow conclusions to be reached with statistical confidence that could not be reached with smaller numbers of sequences, even if those sequences were complete genomes^21^.

The fact that some transcripts go undetected due to sampling effects will affect the sensitivity of the analysis (failing to find evidence for gain or loss of sequences that have) but it is not expected to increase the number of false positives (sequences that appear to have been lost or gained on an evolutionary branch, but really have not). The null hypothesis for statistical tests, after all, is that the observed distribution has arisen precisely because of sampling effects, and these tests implicitly take into account the observed fraction of hits. The more incomplete the transcriptomes, the more likely it is that a truly uniform distribution can appear skewed by chance, but the threshold for deeming the skewed distribution to be statistically significant becomes higher as well. The real risk that false positives will arise from sampling errors comes from the possibility that transcript coverage itself could be non-randomly distributed across different taxa. However, this seems not to be the case based on the number of transcripts assembled from each of the samples (Supplementary Figure S1). More importantly, if false positives due to skewed abundances were a substantial problem we would expect to see, for most of the HMMs that show skewed distributions at all, similar patterns of under- and over-representation, but instead we see different patterns for different sequences.

A more important issue with the use of transcriptomes, as compared to genomes, is one of interpretation. The fact that there is a real difference in the distribution of HMM hits across a phylogenetic tree does not mean that genes themselves have been lost or gained. The same kind of skewed distributions in HMM hits could arise from differences in gene expression levels, and the larger those differences the more plausible this is. For some of the pathways for which we have found skewed distributions there is direct and independent evidence for gene loss or gain. In other cases, though, there is no such evidence, and in these cases we need to remember that biased distributions in HMM hits could be due to clade-specific differences in gene expression levels. Of course, evolutionary increases or decreases in expression that are, by definition, large enough and consistent enough to lead to the biased HMM hit distributions that we observe may well be as interesting and biologically important as the gain or losses of the genes themselves.

It is also important to keep in mind that a statistically significant match to an HMM does not mean that the sequence necessarily has the activity that is ascribed to that HMM, in part because the HMM may not be specific for the activity that is implied by its name. A case in point, discussed above in the context of terpenoid synthesis, is the HMM we constructed for mevalonate kinase (EC 2.7.1.36). We found that this HMM also recognizes galactokinase (EC 2.7.1.6), and so we chose not to use it in our analysis. Every HMM, though, lies somewhere on a spectrum of specificity; specificity is not an all-or-nothing trait. Partly, this is due to real differences in biochemical specificity; some proteins are simply less specific for their substrates or binding partners than others. Partly, it is due to differences in the ‘sequence resolution’ that distinguishes biological activities; some proteins occupy distinct, well-resolved regions of ‘sequence space’ that are unlikely to lead to mis-annotation, while others are so similar in sequence to proteins of a distinct function that they cannot be perfectly resolved by a single HMM. Finally, some HMMs may lack specificity because they were built from sequences with the incorrect specificity, part of a chain of annotation errors propagating the misattribution of specificity.

In future, rather than using a single HMM to infer function, annotation might benefit from comparisons of HMM bitscores, using HMMs of overlapping specificity. It might also be instructive to take into account the phylogenetic patterns of HMM hits, under the assumption that better annotation criteria are likely to imply more evolutionary histories that are more parsimonious. In fact, the first example we have used here, that of CbbX-type Rubisco Activase, is a good illustration of this concept. We first build an HMM for this activity from a set of sequences found by PSI-BLAST, using as the seed a proteobacterial cbbX coding sequence (Methods). Applying to this HMM the same 10-bit cutoff used for all other HMMs in this paper, sequences were found in all of the rhodophytes (12/12) and Chromista (17/17). However, we also found hits in 30 of the 146 green alga (Supplementary Figure S3). The skewed distribution towards algae of the red lineage is significant (P < 10^−15^), but imperfect. It seems likely that the widely scattered green alga sequences that match the HMM for CbbX-type Rubisco activase are really artifacts, a consequence of the fact that this enzyme is a member of a very large and diverse sequence superfamily, the AAA+ family, represented by the Pfam AAA (PF00004) HMM. By comparing bitscores for the AAA HMM and the CbbX-type Rubisco activase, and making the assumption that a more accurate annotation of CbbX-type Rubisco activase would produce a more parsimonious phylogenetic pattern, we were easily able to define a criterion that produces a perfect phylogenetic resolution for this activity. (Methods; Supplementary Figure S3).

The analysis reported here, though focused on algae, supports the more general notion that transcriptomic surveys can provide a powerful complement to genome sequencing. In principle, transcriptome sequencing allows for a much deeper sampling of species, with the attendant statistical power that comes from that sampling. Our analysis suggests that an even deeper sampling of ‘phylogenetic space’ and a more comprehensive survey of protein domains, would likely yield new insights into the evolution of metabolic pathways. By sequencing greater numbers of species in closely related groups it should be possible to access more recent evolutionary changes than those we have been able to explore here. In addition, in focusing on a limited set of 167 HMMs we have examined only a small fraction of the metabolic potential of algae. It will be of interest, and perhaps even of practical value to the development of algal-based technologies, to explore more deeply and broadly the evolution of metabolism in these organisms.

Just how reliably we might infer differences and novelties in algal metabolism from genomic data is an open question. However, as a foundation for hypothesis generation there are unquestioned advantages. First, sequence information can be used to assess the likelihood that a metabolic pathway exists almost without regard to the nature of the pathway. The more direct alternative is to perform biochemical assays for each function of interest, but that is enormously more difficult. Second, the procedures for nucleic acid extraction and purification are nearly standard regardless of the organism, making it relatively easy to conduct a broad phylogenetic survey; this is much less true for enzymological studies.

## Methods

### RNA preparation, sequencing and assembly

The transcriptomes used in this analysis were sequenced as part of the OneKP project. RNA was prepared as described.(21). Most of the algal transcriptomes used in the present analysis were from RNA samples provided by M.M; nine were provided by F.Q.Y.G and N.D.C; other labs provided some as well. Information on the samples included in these analyses is provided in Supplementary Table S1. Paired end sequencing was done on the Illumina platform and assembled by BGI. Details are provided in the OneKP ‘capstone’ paper currently under review.

### Protein sequence hidden markov models

We used hidden Markov models representing 167 protein sequence alignments to infer functions for some of the 8.5 million transcripts in our dataset. The functions for which HMMs were selected correspond largely to photosynthesis, central carbon metabolism, lipid synthesis, and terpenoid synthesis. We also targeted enzymatic activities associated with vitamin synthesis and utilization. Rounding out the set of functions we sought to infer were some that have putative roles in sexual reproduction and/or homologous recombination as these might help guide the selection of tractable species for future experimental work.

Thirty-seven of the HMMs came from the Pfam database^4^ and are referred to here by their Pfam names, prefixed with “Pfam:” An additional 126 HMMs were built from sequence alignments obtained from the KEGG database^5^ using hmmbuild (HMMER^22^;). They are referred to here using the prefix “EC:” followed by the Enzyme Commission number for the enzyme. The remaining four HMMs were built using hmmbuild from sequence alignments obtained from PSI-BLAST searches using as query sequences (i) *Chlamydomonas* oxoglutarate-malate translocator (NCBI: XP_001696590);(ii) *Chlamydomonas* SPO11-like topoisomerase (NCBI: XP_001701722), *Chlamydomonas* acyl-carrier protein (NCBI: XP_001699275), and (iv) the CbbX Rubisco activase from *Rhodobacter sphaeroides*^9^. Supplementary Table S2 provides the full list of HMMs.

HMM searches of the transcriptomes were conducted using genewisedb, part of the Wise2 package (Wise 2.4.1)^23^. Default parameters were used for all searches; matches with a bit-score above 10 were considered positive hits. In the case of CbbX-like Rubisco activase, a member of the AAA+ family, sequences were scored as Rubisco activase only if the bitscores for the CbbX HMM were at least five-fold greater than for the Pfam:AAA HMM. The number of matching transcripts found for each of the 167 HMMs and in each of the 183 transcriptomes is provided in Supplementary Table S3.

### Phylogenetic tree

The OneKP consortium constructed a phylogenetic tree that included more than 1000 algal and plant transcriptomes. The tree was constructed using Astral, which constructs species trees based on a set of gene trees, and was kindly provided by J. Leebens-Mack in advance of publication. The tree includes 175 of the 183 samples we analyzed; thus, for analyses that rely on the Astral tree, we used this subset of samples. The R packages *ape*^24^ and *dendextend*^25^ were used to remove other species, recalculate branch lengths, and reorder branches. For visualization of sequence distributions within the Chlorophyta, a simplified version of the tree was produced, removing uninformative nodes and producing a set of 13 terminal clusters, ranging from two samples to thirteen. In brief, we began with the tree of Chlorophyta samples and successively assessed the effect of cutting the tree at each node, starting at the highest level (splitting the chlorophytes into two groups), then the next (three groups). and so on. For each successive cut, we determined, for each of the 167 HMMs, the Fisher's exact test p-value for non-random distribution of hits across the full set of chlorophytes. Nodes deemed uninformative were the ones for which daughter branches failed to improve substantially the significance of any one of the HMMs. In practice, these were branches with a single sample or at most two. For purposes of visualizing the distributions, samples in these branches were merged with those of the smallest branch in the next descendant node that met the threshold for significance. This process was stopped when we reached 13 terminal clusters because no further splitting of these clusters had a substantial effect on statistical significance.

### Glyoxylate shunt genes in KEGG complete genomes

The KEGG GENOME database (https://www.genome.jp/kegg/genome.html) contains fourteen algal genomes, grouped into two ‘organism groups', green algae (11) and red algae (3). Each of the groups was searched using the keywords “isocitrate lyase” and “isocitrate lyase”, as well as their corresponding EC numbers. Species with matches to isocitrate lyase and/or EC 4.1.3.1 consisted of *Chlamydomonas reinhardtii*, *Monoraphidium neglectum*, *Coccomyxa subellipsoidea*, and *Auxenochlorella protothecoides*. All of these species, plus *Volvox carteri*, had matches to malate synthase and/or EC 2.3.3.9. None of the other algal genomes in KEGG had matches to any of the search terms. These other species are *Ostreococcus lucimarinus*, *Ostreococcus tauri*, *Bathycoccus prasinos*, *Micromonas commode*, *Micromonas pusilla*, *Chlorella variabilis*, *Cyanidioschyzon merolae*, *Galdieria sulphuraria*, and *Chondrus crispus*.

### Identification of Pfam:DUF3419 as a predictor of DGTS synthesis

*Rhodobacter sphaeroides* genes BtaA and BtaB are the canonical genes for DGTS synthesis^26^. To obtain an HMM suitable for predicting DGTS synthesis, we searched Pfam with these two sequences. BtaB matched an HMM called Methyltransf_23, but sequences detected by this HMM turn out to be very widespread. BtaA, on the other hand, was very strongly matched by Pfam:DUF3419 (P = 2 × 10^−106^) and had a distribution that seemed plausibly specific. To test whether DUF3419 expression correlates with DGTS synthesis, we analyzed lipid extracts from 25 species for which we had transcriptome data, measuring DGTS and PC by mass spectrometry. Of the 25 strains from which we analyzed lipids, 21 had transcriptomes that were DUF3419-positive. (Supplementary Table S4). Of those 21, 20 were confirmed to be DGTS positive. All of them, in fact, had more DGTS than PC. Conversely, of the four strains that lacked a DUF3419-positive transcript, three had undetectable levels of DGTS. The one exception – having DGTS despite an apparent absence of the relevant transcript - is almost certainly due to incomplete coverage of the transcriptome. Less easily explained is the absence of detectable DGTS in one of the 21 strains with a DUF3419-positive transcript. However, it is also the case that this strain (*Picocystis salinarum*) was the only one of the DUF3419-positive strains whose transcriptome also included hits to all of the Kennedy pathway enzymes for PC synthesis (2.7.1.32, 2.7.7.15, 2.7.8.2) plus a hit to phosphatidylethanolamine methyltransferase (2.1.1.103), which feeds phosphocholine to the Kennedy pathway through an alternative source. Thus, the failure to detect DGTS in this single DUF3419 positive strain sample may be due to a particularly high transcriptional activation of the PC pathway. In any case, the association between DGTS in lipid extracts and a DUF3419-postive transcript is significant (P < 0.01), and so we have proceeded to use sequence similarity to the DUF3419 HMM to infer the ability to synthesize DGTS.

### Characterization of PC and DGTS using mass spectrometry

Lipid extraction was done according to Bligh and Dyer^27^. Briefly, cell pellets were thawed on ice for 10 min. 900 µL of freshly prepared, chilled extraction solvents (chloroform (CHCl3)/methanol (MeOH) 1:2 (v/v)) were added to the cell pellets and re-suspension achieved by pipetting the solvent several times. Cell suspensions were transferred into 2 mL screw-cap tube containing ~300 mg of glass bead (acid washed, Sigma). The tubes were beaten for 5 min in cold room (4 °C) using a homogenizer, followed by addition of 300 µL of chilled deionized water and shaking in cold room for 1-2 h. 300 µL of chilled CHCl3 were added, followed by brief vortexing and centrifugation at 9,000 rpm for 2 min at 4 °C. The lower phase was collected into a new tube. Algal cells were re-extracted with 500 µL of chilled CHCl3 with 1 h shaking. The lower phases were pooled together and dried in a speed vac. Dried lipid extracts were kept in −80 °C for mass spectrometric (MS) analysis. Total lipid extracts were dissolved in 200 µL of CHCl3/MeOH 1:1 (v/v) containing phosphatidylcholine (14:0/14:0, DMPC) as an internal standard prior to MS analysis.

The LC-MS analysis of algal extracts was undertaken using liquid chromatography coupled with electrospray ionization mass spectrometry (LC-ESI-MS) on an LTQ-Orbitrap XL (Thermo scientific, USA). A Zorbax Eclipse XDB-C18 column (3.0 × 150 mm, 1.8 µm particle size, Agilent Technologies, USA) and a mobile phase consisting of chloroform-methanol (1:1, vol/vol) containing 2 mM ammonium acetate were used. Chromatographic separation was achieved isocratically at a flow rate of 130 µl/min. The total runtime was 20 min. The LTQ-Orbitrap XL was operated with positive ionization with an electrospray voltage of 3.8 kV, capillary temperature 300 °C, Sheat gas flow 40, Auxiliary gas flow, 10, sweep gas flow 10. The m/z range was 300–1000. Under these conditions, PC and DGTS ionize as protonated ions ([M+ H]+) and elute between 8 and 19 min depending on chain length and unsaturation. Raw MS data were manually curated using Xcalibur software (Thermo Scientific, USA). PC and DGTS species were identified with an MS tolerance of 8 ppm. The identification was based on both accurate mass and retention times. In the absence of adequate commercial standard for quantification of DGTS, the DGTS/PC ratios were calculated as the ratio of the total abundance of DGTS over the total abundance of PC. Total abundances for both classes were calculated by adding up the area under curve (AUC) of individual PC and DGTS extracted ion chromatograms

## Supplementary information

Supplementary Information

## Data Availability

Transcripts are available through the OneKP consortium. (5) (https://sites.google.com/a/ualberta.ca/onekp/) HMMs, other than those that are part of the Pfam database, are available from the corresponding author. Derived data, including the matrix of hits for 167 HMMs vs 183 transcriptomes and the Fisher exact p-values for the non-randomness of HMM hit distributions are provided in Supplementary Information.
