# Supplementary material for: Gains and losses of metabolic function inferred from a phylotranscriptomic analysis of algae
Source: Sci Rep. 2019 Jul 19;9:10482. doi: 10.1038/s41598-019-46869-3 (PMC6642084; doi:10.1038/s41598-019-46869-3)
Supplement: Supplementary file 1 — Supplementary Information [file 41598_2019_46869_MOESM1_ESM.pdf]

**Supplementary Information**

# Gains and losses of metabolic function inferred from a phylotranscriptomic analysis of algae

Falicia Qi Yun Goh[a,b], Justin Jeyakani[b], Phornpimon Tipthara[b], Amaury Cazenave-Gassiot[c], Rajoshi Ghosh[b], Nicholas Bogard[b], Zhenxuan Yeo[a,b], Gane Ka-Shu Wong[e,f,g] , Michael Melkonian[h], Markus R. Wenk[c], and Neil D. Clarke[a,b,d,*]

[a] Yale-NUS College
Singapore 138527, Singapore

[b] Computational and Systems Biology
Genome Institute of Singapore
Singapore 138672

[c] Department of Biochemistry
Yong Loo Lin School of Medicine
National University of Singapore
Singapore 117596

[d] Department of Biological Sciences
National University of Singapore
Singapore 117543

[e] Department of Biological Sciences
University of Alberta
Edmonton, AB, T6G 2E9, Canada

[f] Department of Medicine
University of Alberta
Edmonton, AB, T6G 2E1, Canada

[g] BGI-Shenzhen
Beishan Industrial Zone, Yantian District
Shenzhen 518083, China

[h] Botanical Institute, Cologne Biocenter,
University of Cologne
50674 Cologne, Germany

Corresponding author:
Neil D. Clarke
Yale-NUS College
6 College Avenue West
Singapore 138527
+65 8123 1497
neil.clarke@yale-nus.edu.sg

Supplementary Figure S1

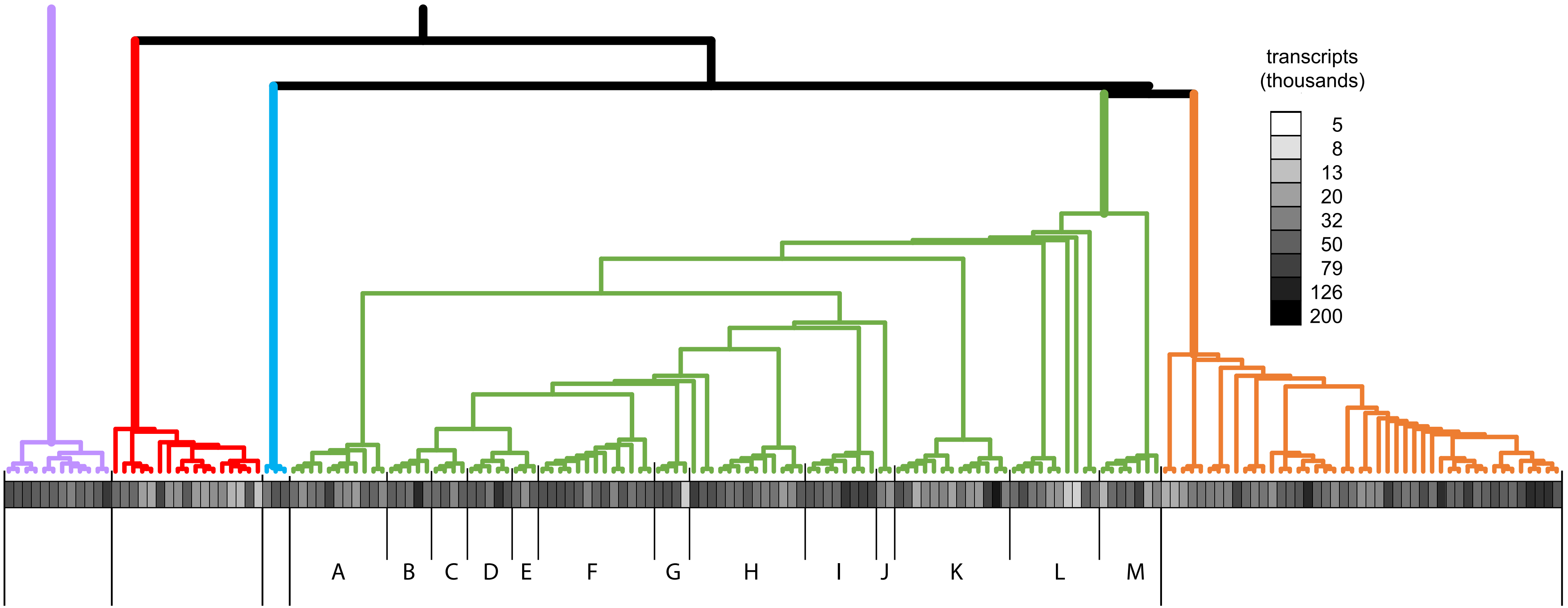


**Supp Figure 1**: Phylogenetic tree with transcript numbers and chlorophyte cluster labels. The number of transcript scaffolds assembled for each of the samples in the tree is indicated in the heatmap. Long vertical lines below the heatmap demarcate the five major clades. Shorter lines demarcate the 13 clusters of Chlorphyta defined as described in Methods. Clusters are labeled A-M.

Supplementary Figure S2

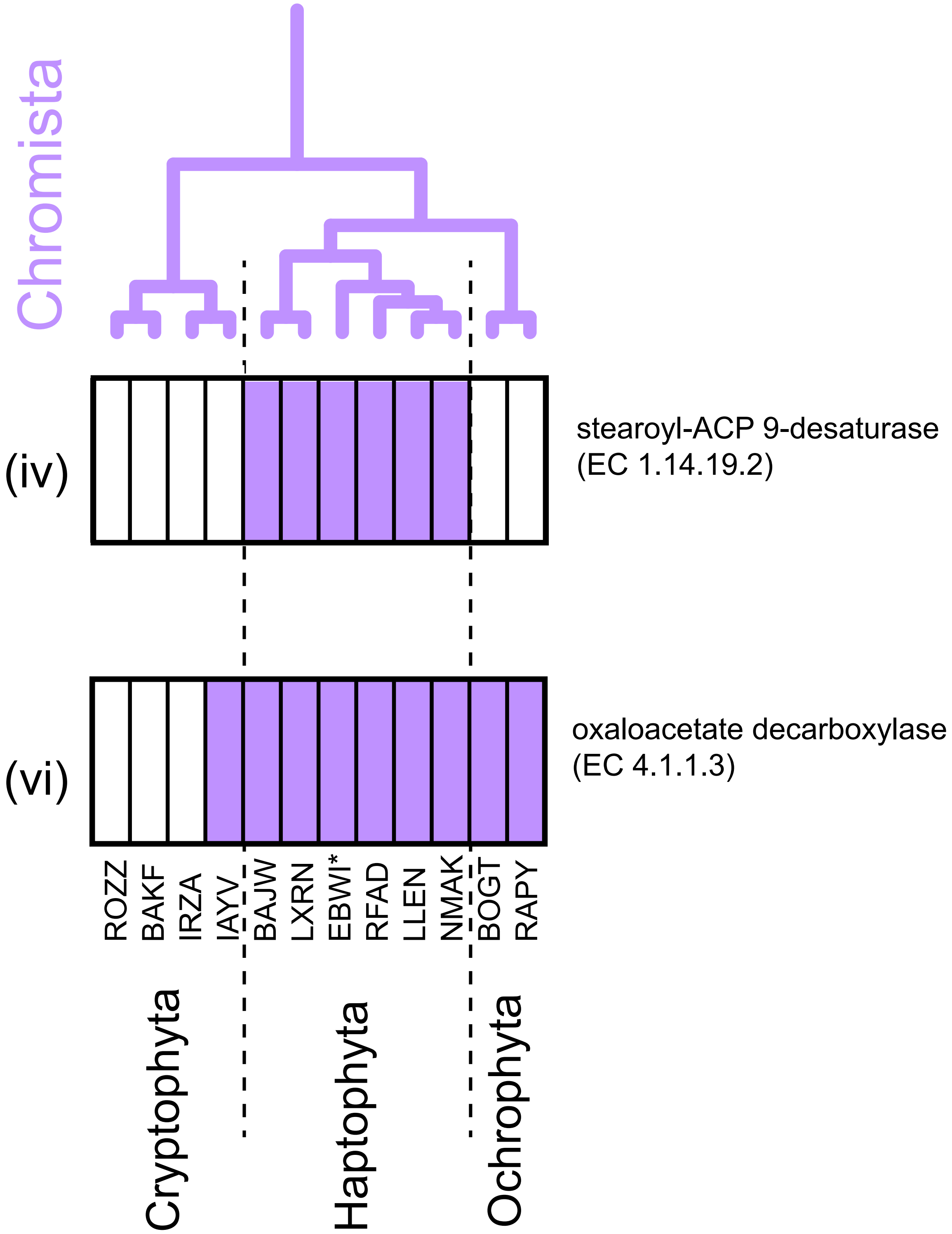


**Supp Figure S2**: This is essentially a close-up of the Chromista region from Fig 1A in the paper, and Fig 1B, HMMs iv and vi. This closeup provides the OneKP codes for the 12 chromista samples. Dashed lines help distinguish the three main clades as defined by the tree. These clades correspond very closely to the traditional taxonomic classification, given by the labels at the bottom. (There is only one sample - EBWI, indicated by an asterisk - whose placement is suspect). Note that all six samples that have hits to EC 1.14.19.2 (a fatty acid desaturase) are in the clade labeled as Haptophyta; conversely all of the samples in the clade have the sequence. Oxaloacetate decarboxylase is absent in only three of the twelve Chromista, but all three of the sampes in which it is missing are cryptophytes.

Supplementary Figure S3

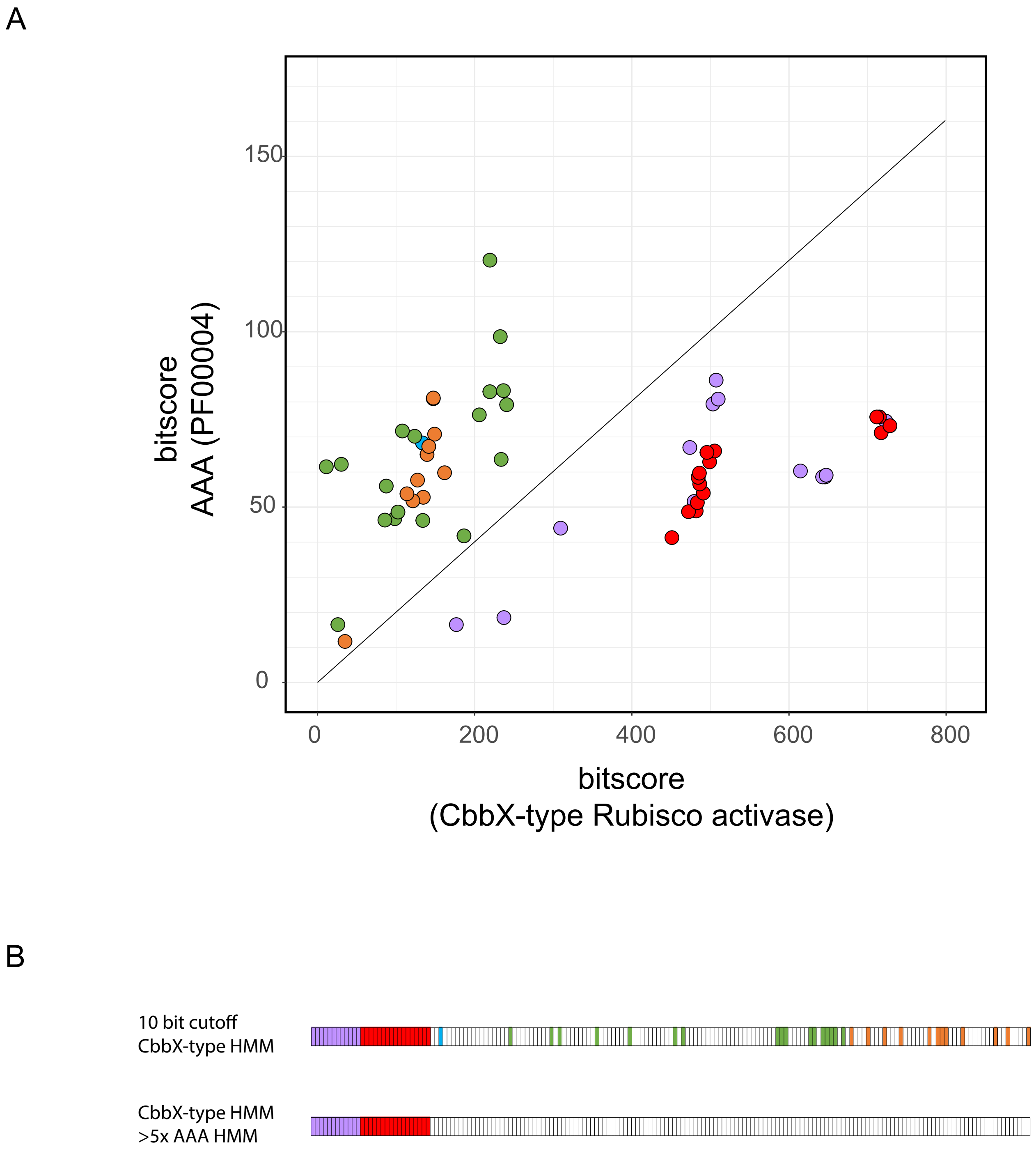


**Supp Figure S3**: Alternative criteria for defining the presence of CbbX-type Rubisco activase sequences. (A) Plot of all samples for which a hit was found to an HMM for CbbX-type Rubisco activase at a threshold of 10 bits. The y-axis value is the bit-score for the same scaffold run against the Pfam HMM PF00004 (AAA). Samples are color coded in the same way as figures in the manuscript: purple are Chromista; red are rhodophytes; green are chlorophytes; and orange are streptophyte algae. The diagnoal line has an intercept of 0 and a slope of 0.2; points to the right of this line have bitscores for CbbX-type Rubisco activase that are at least 5x higher than for the AAA HMM. (B) Representation of CbbX-type positive samples based on only the 10 bit threshold criterion (top) and for the criterion used int he paper, which is the same 10 bit threshold, plus a bitscore for CbbX-type HMM at least 5-fold higher than for the AAA HMM.

Supplementary Table S1

| Data in columns A:I were downloaded from http://www.onekp.com/samples/list.php. | | | | | | | | | additional annotations | | | | |
|---|---|---|---|---|---|---|---|---|---|---|---|---|---|
| 1kP_Sample *[sample codes]* | Clade | Order | Family | Species | Tissue Type | Voucher Data *[culture collection strain names]* | Sample Provider | Notes *[appended to bottom]* | order (left to right) in tree figs | #scaffolds in assembly | Chlorophyte class (based on Algaebase) | chlorophyte cluster (see Methods, Fig S1) | prasinophytes |
| ROZZ | Chromista (Algae) | Pyrenomonadales | Chroomonadacea | Chroomonas sp. | algal cells | M1312 | M. Melkonian | | 1 | 62685 | | | |
| BAKF | Chromista (Algae) | Cryptomonadales | Cryptomonadace | Cryptomonas curvata | algal cells | CCAC 0006 (M0420) | M. Melkonian | | 2 | 56233 | | | |
| IRZA | Chromista (Algae) | Pyrenomonadales | Geminigeraceae | Proteomonas sulcata | algal cells | CCMP 704 | M. Melkonian | | 3 | 62109 | | | |
| IAYV | Chromista (Algae) | Pyrenomonadales | Pyrenomonadace | Rhodomonas sp. | algal cells | M1630 | M. Melkonian | | 4 | 51627 | | | |
| BAJW | Chromista (Algae) | Isochrysidales | Isochrysidaceae | Isochrysis sp. | algal cells | CCAP 927/14 | M. Melkonian | | 5 | 45228 | | | |
| LXRN | Chromista (Algae) | Prymnesiales | Prymnesiaceae | Prymnesium parvum | algal cells | SAG 127.79 | M. Melkonian | | 6 | 48769 | | | |
| EBWI | Chromista (Algae) | Chromulinales | Chromulinaceae | Ochromonas sp. | algal cells | M1920 | M. Melkonian | | 7 | 37616 | | | |
| RFAD | Chromista (Algae) | Pavlovales | Pavlovaceae | Pavlova lutheri CCAC 0028 | algal cells | CCAC 0028 (M1025) | M. Melkonian | | 8 | 30689 | | | |
| LLEN | Chromista (Algae) | Pavlovales | Pavlovaceae | Pavlova lutheri SAG 926-1 | algal cells | SAG 926-1 | M. Melkonian | | 9 | 45335 | | | |
| NMAK | Chromista (Algae) | Pavlovales | Pavlovaceae | Pavlova lutheri | cells grown on F/2 | UTEX LB 1293 | N. Clarke | | 10 | 38916 | | | |
| BOGT | Chromista (Algae) | Synurales | Mallomonadacea | Mallomonas sp. | algal cells | CCAC 0094 | M. Melkonian | | 11 | 51201 | | | |
| RAPY | Chromista (Algae) | Laminariales | Laminariaceae | Saccharina sculpera | blades | Tao Liu 201107019 | Jun Yu | | 12 | 88085 | | | |
| LLXJ | Red Algae | Stylonematales | Stylonematacea | Chroodactylon ornatum | algal cells | CCAP 1364/1 | M. Melkonian | | 13 | 43085 | | | |
| JJZR | Red Algae | Rhodochaetales | Rhodochaetacea | Rhodochaete parvula | algal cells | SAG 8.99 | M. Melkonian | | 14 | 37895 | | | |
| RSOF | Red Algae | Glaucosphaerales | Glaucosphaerace | Glaucosphaera vacuolata | algal cells | SAG 13.82 | M. Melkonian | | 15 | 43879 | | | |
| PVGP | Red Algae | Porphyridiales | Porphyridiaceae | Porphyridium purpureum | algal cells | SAG 1380-1c | M. Melkonian | | 16 | 20562 | | | |
| OBUY | Red Algae | Porphyridiales | Porphyridiaceae | Porphyridium cruentum | cells grown on F/2 | UTEX 161 | N. Clarke | | 17 | 18242 | | | |
| ZULJ | Red Algae | Bangiales | Bangiaceae | Pyropia yezoensis | blades | Tao Liu 201104053 | Jun Yu | | 18 | 72975 | | | |
| XAXW | Red Algae | Ceramiales | Rhodomelaceae | Neosiphonia japonica | branches and blad | Tao Liu 201104042 | Jun Yu | | 19 | 24378 | | | |
| JEBK | Red Algae | Gigartinales | Solieriaceae | Eucheuma denticulatum | branches | Tao Liu 201010021 | Jun Yu | | 20 | 25026 | | | |
| IHJY | Red Algae | Gigartinales | Solieriaceae | Kappaphycus alvarezii | branches | Tao Liu 201103110 | Jun Yu | | 21 | 54853 | | | |
| CKXF | Red Algae | Gigartinales | Phyllophoraceae | Ahnfeltiopsis flabelliformis | branches and blad | Tao Liu 201108707 | Jun Yu | | 22 | 21987 | | | |
| SBLT | Red Algae | Gigartinales | Endocladiaceae | Gloiopeltis furcata | branches and blad | Tao Liu 201104135 | Jun Yu | | 23 | 20185 | | | |
| UGPM | Red Algae | Gigartinales | Gigartinaceae | Chondrus crispus | blades | Tao Liu 201104049 | Jun Yu | | 24 | 25527 | | | |
| PYDB | Red Algae | Halymeniales | Halymeniaceae | Grateloupia catenata | branches and blad | Tao Liu 201108705 | Jun Yu | | 25 | 28270 | | | |
| URSB | Red Algae | Halymeniales | Halymeniaceae | Grateloupia turuturu | blades | Tao Liu 201104125 | Jun Yu | | 26 | 15114 | | | |
| IKIZ | Red Algae | Halymeniales | Halymeniaceae | Grateloupia livida | blades | Tao Liu 201104057 | Jun Yu | | 27 | 14255 | | | |
| ZJOJ | Red Algae | Halymeniales | Halymeniaceae | Grateloupia filicina | branches and blad | Tao Liu 201104047 | Jun Yu | | 28 | 56235 | | | |
| FTRP | Red Algae | Gracilariales | Gracilariaceae | Gracilaria chouae | blades | Tao Liu 201104180 | Jun Yu | | 29 | 12073 | | | |
| QFND | Glaucophyta (Alga | Glaucocystales | Glaucocystaceae | Cyanophora paradoxa CCAC 0074 (M | algal cells | CCAC 0074 (M0763) | M. Melkonian | | 30 | 36658 | | | |
| POOW | Glaucophyta (Alga | Glaucocystales | Glaucocystaceae | Glaucocystis cf. nostochinearum | algal cells | CCAC 0088 (M1106/1) | M. Melkonian | | 31 | 58140 | | | |
| PQED | Glaucophyta (Alga | Glaucocystales | Glaucocystaceae | Gloeochaete wittrockiana | algal cells | SAG 46.84 | M. Melkonian | | 32 | 54785 | | | |
| CBNG | Green Algae | Ulotrichales | Borodinellaceae | Planophila laetevirens | algal cells | SAG 2008 | M. Melkonian | | 33 | 41649 | Ulvophycea | A | |
| LETF | Green Algae | Ulotrichales | Borodinellaceae | Planophila terrestris | algal cells | SAG 32.98 | M. Melkonian | | 34 | 25194 | Ulvophycea | A | |
| AJAU | Green Algae | Chaetophorales | Chaetophoracea | Helicodictyon planctonicum | algal cells | UTEX LB 1570 | M. Melkonian | | 35 | 37626 | Chlorophyceae | A | |
| JIWJ | Green Algae | Ulotrichales | Ulotrichaceae | Acrosiphonia sp. | algal cells | SAG 127.80 | M. Melkonian | | 36 | 41151 | Ulvophycea | A | |
| CQQP | Green Algae | Ulvales | Ulvaceae | Ochlochaete sp. | algal cells | CCMP 2390 | M. Melkonian | 1 | 37 | 77098 | Ulvophycea | A | |
| OAEZ | Green Algae | Ulvales | Ulvaceae | Percursaria percursa | algal cells | M1835 | M. Melkonian | | 38 | 30043 | Ulvophycea | A | |
| NQYP | Green Algae | Chaetophorales | Chaetophoracea | Pirula salina | algal cells | SAG 1.95 | M. Melkonian | | 39 | 28302 | Chlorophyceae | A | |
| GJIY | Green Algae | Sphaeropleales | Neochloridaceae | Neochloris sp. | algal cells | UTEX LB 1445 | M. Melkonian | | 40 | 21703 | Chlorophyceae | A | |
| KADG | Green Algae | Ulotrichales | Ulotrichaceae | Ignatius tetrasporus | algal cells | UTEX 2012 | M. Melkonian | | 41 | 47187 | Ulvophycea | A | |
| VBLH | Green Algae | Cladophorales | Cladophoraceae | Cladophora glomerata | algal cells | CPCC 13 | M. Melkonian | | 42 | 47161 | Ulvophycea | A | |
| YDCQ | Green Algae | Trentepohliales | Trentepohliacea | Cephaleuros virescens | algal cells | SAG 28.93 | M. Melkonian | | 43 | 27954 | Ulvophycea | A | |
| ZDIZ | Green Algae | Volvocales | Dunaliellaceae | Dunaliella tertiolecta | algal cells | CCMP 364 | H. Sederoff | | 44 | 51322 | Chlorophyceae | B | |
| WDWX | Green Algae | Volvocales | Dunaliellaceae | Dunaliella tertiolecta | algal cells | UTEX LB 1000 | H. Sederoff | 2 | 45 | 45761 | Chlorophyceae | B | |
| RHVC | Green Algae | Volvocales | Dunaliellaceae | Dunaliella salina | algal cells | SAG 42.88 | M. Melkonian | | 46 | 38926 | Chlorophyceae | B | |
| UKUC | Green Algae | Volvocales | Dunaliellaceae | Dunaliella salina | algal cells | UTEX LB 200 | H. Sederoff | | 47 | 109228 | Chlorophyceae | B | |
| SYJM | Green Algae | Volvocales | Dunaliellaceae | Dunaliella viridis dumsii | algal cells | UTEX LB 1644 | H. Sederoff | 3 | 48 | 41020 | Chlorophyceae | B | |
| QRTH | Green Algae | Volvocales | Chlamydomonad | Chlorococcum microstigmaticum | algal cells | SAG 11.43 | M. Melkonian | 4 | 49 | 43057 | Chlorophyceae | C | |
| PRIQ | Green Algae | Pleurastrales | Pleurastraceae | Chlorococcum oleofaciens | algal cells | SAG 30.93 | M. Melkonian | 5 | 50 | 52089 | Chlorophyceae | C | |
| ZLQE | Green Algae | Volvocales | Haematococcace | Stephanosphaera pluvialis | algal cells | SAG 78-1a | M. Melkonian | | 51 | 28849 | Chlorophyceae | C | |
| GUBD | Green Algae | Volvocales | Chlamydomonad | Brachiomonas submarina | algal cells | CCAC 0069 (M1823) | M. Melkonian | | 52 | 51744 | Chlorophyceae | C | |
| MULF | Green Algae | Volvocales | Chlamydomonad | Chlamydomonas bilatus | algal cells | SAG 7.72 | M. Melkonian | | 53 | 53577 | Chlorophyceae | D | |
| VALZ | Green Algae | Volvocales | Chlamydomonad | Chlamydomonas noctigama | algal cells | SAG 36.72 | M. Melkonian | | 54 | 54524 | Chlorophyceae | D | |
| JRGZ | Green Algae | Volvocales | Chlamydomonad | Chlamydomonas moewusii | algal cells | SAG 1.98 | M. Melkonian | | 55 | 36079 | Chlorophyceae | D | |
| GFUR | Green Algae | Volvocales | Chlamydomonad | Chloromonas subdivisa | algal cells | SAG 67.72 | M. Melkonian | | 56 | 95867 | Chlorophyceae | D | |
| TSBQ | Green Algae | Volvocales | Chlamydomonad | Chlamydomonas sp. | algal cells | M2762 | M. Melkonian | | 57 | 61904 | Chlorophyceae | D | |
| ACRY | Green Algae | Volvocales | Phacotaceae | Pteromonas sp. | algal cells | M2761 | M. Melkonian | | 58 | 43687 | Chlorophyceae | E | |
| LNIL | Green Algae | Volvocales | Phacotaceae | Pteromonas angulosa | algal cells | M2612 | M. Melkonian | | 59 | 25461 | Chlorophyceae | E | |
| ZIVZ | Green Algae | Volvocales | Phacotaceae | Phacotus lenticularis | algal cells | SAG 61-1 | M. Melkonian | | 60 | 52103 | Chlorophyceae | E | |
| WRSL | Green Algae | Volvocales | Volvocaceae | Volvox aureus | algal cells | M2242 | M. Melkonian | | 61 | 62468 | Chlorophyceae | F | |
| JWGT | Green Algae | Volvocales | Volvocaceae | Volvox aureus | algal cells | M1028 | M. Melkonian | | 62 | 63713 | Chlorophyceae | F | |
| RYJX | Green Algae | Volvocales | Volvocaceae | Pandorina morum | algal cells | CCAC 0063 | M. Melkonian | | 63 | 64557 | Chlorophyceae | F | |
| RNAT | Green Algae | Volvocales | Volvocaceae | Eudorina elegans | algal cells | CCAC 0011 | M. Melkonian | | 64 | 59712 | Chlorophyceae | F | |
| ISPU | Green Algae | Volvocales | Volvocaceae | Volvox globator | algal cells | ASW 05075 | M. Melkonian | | 65 | 52127 | Chlorophyceae | F | |
| IHOI | Green Algae | Volvocales | Chlamydomonad | Chloromonas oogama | algal cells | SAG 9.79 | M. Melkonian | | 66 | 30344 | Chlorophyceae | F | |
| BCYF | Green Algae | Volvocales | Chlamydomonad | Chlamydomonas cribrum | algal cells | SAG 13.72 | M. Melkonian | | 67 | 47025 | Chlorophyceae | F | |
| JKKI | Green Algae | Volvocales | Chlamydomonad | Lobomonas rostrata | algal cells | SAG 45-1 | E. Kazamia | | 68 | 58095 | Chlorophyceae | F | |
| USIX | Green Algae | Chlorosarcinales | Chlorosarcinacea | Neochlorosarcina sp. | clonal culture, veg | M 2315 | M. Melkonian | | 69 | 30224 | Chlorophyceae | F | |
| QWRA | Green Algae | Volvocales | Chlamydomonad | Vitreochlamys sp. | algal cells | M2074 | M. Melkonian | | 70 | 59097 | Chlorophyceae | F | |
| IRYH | Green Algae | Volvocales | Chlamydomonad | Heterochlamydomonas inaequalis | algal cells | SAG 4.75 | M. Melkonian | | 71 | 37353 | Chlorophyceae | F | |
| OFUE | Green Algae | Volvocales | Chlamydomonad | Lobochlamys segnis | algal cells | SAG 50.84 | M. Melkonian | | 72 | 47755 | Chlorophyceae | F | |
| XDLL | Green Algae | Volvocales | Chlamydomonad | Oogamochlamys gigantea | algal cells | SAG 44.91 | M. Melkonian | | 73 | 50148 | Chlorophyceae | F | |
| AJUW | Green Algae | Volvocales | Chlamydomonad | Chloromonas rosae | algal cells | UTEX SNO 47 | M. Melkonian | | 74 | 58510 | Chlorophyceae | G | |
| LBRP | Green Algae | Volvocales | Chlamydomonad | Chloromonas reticulata | algal cells | SAG 26.86 | M. Melkonian | | 75 | 63012 | Chlorophyceae | G | |
| AOUJ | Green Algae | Volvocales | Chlamydomonad | Chlamydomonas sp. | algal cells | M2779 | M. Melkonian | | 76 | 57048 | Chlorophyceae | G | |
| UTRE | Green Algae | Volvocales | Chlamydomonad | Chloromonas tughillensis | algal cells | UTEX SNO 88 | M. Melkonian | | 77 | 10300 | Chlorophyceae | G | |
| FXHG | Green Algae | Volvocales | Dunaliellaceae | Hafniomonas reticulata | algal cells | M0530/1 | M. Melkonian | | 78 | 82903 | Chlorophyceae | H | |
| RUIF | Green Algae | Volvocales | Carteriaceae | Carteria obtusa | algal cells | SAG 39.84 | M. Melkonian | | 79 | 54689 | Chlorophyceae | H | |
| VIAU | Green Algae | Volvocales | Carteriaceae | Carteria crucifera | algal cells | SAG 8-7a | M. Melkonian | | 80 | 64474 | Chlorophyceae | H | |
| XTON | Green Algae | Chlorococcales | Hydrodictyaceae | Pediastrum duplex CCAC 0147 | algal cells | CCAC 0147 | M. Melkonian | | 81 | 50064 | Chlorophyceae | H | |
| XKWQ | Green Algae | Chlorococcales | Hydrodictyaceae | Pediastrum duplex CCAC 0012 | algal cells | CCAC 0012 (M0549) | M. Melkonian | | 82 | 38322 | Chlorophyceae | H | |
| VJDZ | Green Algae | Trebouxiales | Botryococcaceae | Botryococcus sudeticus | algal cells | UTEX 2629 | N. Clarke | | 83 | 41539 | Trebouxiophyceae | H | |
| PZIF | Green Algae | Chlorococcales | Scenedesmacea | Scenedesmus dimorphus | cells grown on 3N | UTEX 746 | N. Clarke | | 84 | 56356 | Chlorophyceae | H | |
| MWAN | Green Algae | Chlorellales | Chlorellaceae | Chlorella minutissima | cells grown on F/2 | UTEX 2219 | N. Clarke | | 85 | 36163 | Trebouxiophyceae | H | |
| OTQG | Green Algae | Sphaeropleales | Ankistrodesmace | Ankistrodesmus sp. | algal cells | M3332 | M. Melkonian | | 86 | 31799 | Chlorophyceae | H | |
| BZSH | Green Algae | Chlorococcales | Golenkiniaceae | Golenkinia longispicula | algal cells | SAG 73.80 | M. Melkonian | | 87 | 37735 | Chlorophyceae | H | |
| ENAU | Green Algae | Volvocales | Chlamydomonad | Spermatozopsis similis | algal cells | CCAC 0013 (M0563/1) | M. Melkonian | | 88 | 21774 | Chlorophyceae | H | |
| MXDS | Green Algae | Volvocales | Chlamydomonad | Spermatozopsis exsultans | algal cells | M0562 | M. Melkonian | | 89 | 27853 | Chlorophyceae | H | |
| DZPJ | Green Algae | Ulotrichales | Cylindrocapsacea | Cylindrocapsa geminella | algal cells | SAG 3.87 | M. Melkonian | | 90 | 54221 | Ulvophycea | H | |
| JMUI | Green Algae | Chaetophorales | Chaetophoracea | Stigeoclonium helveticum | algal cells | SAG 477-1 | M. Melkonian | | 91 | 56140 | Chlorophyceae | I | |
| VFIV | Green Algae | Chaetophorales | Chaetophoracea | Fritschiella tuberosa | algal cells | SAG 112.8 | M. Melkonian | | 92 | 48720 | Chlorophyceae | I | |
| ISGT | Green Algae | Chaetophorales | Chaetophoracea | Uronema sp. | algal cells | CCAP 334/1 | M. Melkonian | 6 | 93 | 41559 | Chlorophyceae | I | |

| | | | | | | | | | | | | | |
|---|---|---|---|---|---|---|---|---|---|---|---|---|---|
| RAWF | Green Algae | Chaetophorales | Chaetophoraceae | Uronema belkae | algal cells | SAG 34.86 | M. Melkonian | | 94 | 43871 | Chlorophyceae | I | |
| IJMT | Green Algae | Chaetophorales | Aphanochaetace | Aphanochaete repens | algal cells | M2226 | M. Melkonian | | 95 | 92039 | Chlorophyceae | I | |
| SDPC | Green Algae | Oedogoniales | Oedogoniaceae | Oedogonium foveolatum | algal cells | UTEX LB 933 | M. Melkonian | | 96 | 71612 | Chlorophyceae | I | |
| DVYE | Green Algae | Oedogoniales | Oedogoniaceae | Oedogonium cardiacum | algal cells | SAG 575-1a | M. Melkonian | | 97 | 80265 | Chlorophyceae | I | |
| BAZF | Green Algae | Chaetopeltidales | Chaetopeltidacea | Chaetopeltis orbicularis | algal cells | SAG 21.98 | M. Melkonian | | 98 | 72685 | Chlorophyceae | I | |
| JTIG | Green Algae | Bryopsidales | Bryopsidaceae | Bryopsis plumosa | algal cells | KU 272 | M. Melkonian | 7 | 99 | 33234 | Ulvophycea | J | |
| GYBH | Green Algae | Bryopsidales | Codiaceae | Codium fragile | algal cells | KU 654 | M. Melkonian | | 100 | 23572 | Ulvophycea | J | |
| ETGN | Green Algae | Trebouxiales | Botryococcaceae | Botryococcus braunii | cells grown on 3N | UTEX 2441 | N. Clarke | | 101 | 62493 | Trebouxiophyceae | K | |
| QYXY | Green Algae | Trebouxiales | Botryococcaceae | Botryococcus terribilis | algal cells | ASW 05049 | M. Melkonian | | 102 | 49754 | Trebouxiophyceae | K | |
| GXBM | Green Algae | Chlorococcales | Coccomyxaceae | Coccomyxa pringsheimii | algal cells | SAG 216-7 | M. Melkonian | | 103 | 17042 | Chlorophyceae | K | |
| NKXU | Green Algae | Trebouxiales | Trebouxiaceae | Trebouxia arboricola | algal cells | SAG 219-1a | M. Melkonian | | 104 | 28139 | Trebouxiophyceae | K | |
| ZNUM | Green Algae | Chlorellales | Leptosiraceae | Leptosira obovata | algal cells | SAG 445-1 | M. Melkonian | | 105 | 26849 | Trebouxiophyceae | K | |
| WXRI | Green Algae | Prasiolales | Prasiolaceae | Stichococcus bacillaris | algal cells | CCAC 0076 (M1897/1) | M. Melkonian | | 106 | 30268 | Trebouxiophyceae | K | |
| WCLV | Green Algae | Prasiolales | Prasiolaceae | Prasiola crispa | algal cells | SAG 43.96 | M. Melkonian | | 107 | 19130 | Trebouxiophyceae | K | |
| EEJO | Green Algae | Volvocales | incertae sedis | Neochloris oleoabundans | cells grown on 3N | UTEX 1185 | N. Clarke | 8 | 108 | 40853 | Chlorophyceae | K | |
| AKCR | Green Algae | Chlorellales | Chlorellaceae | Parachlorella kessleri | algal cells | SAG 10.80 | M. Melkonian | | 109 | 24207 | Trebouxiophyceae | K | |
| BILC | Green Algae | Chlorellales | Chlorellaceae | Prototheca wickerhamii | algal cells | SAG 263-11 | M. Melkonian | | 110 | 21751 | Trebouxiophyceae | K | |
| JQFK | [Chromista (Algae) | [Eustigmatales] | [Monodopsidace | Picochlorum atomus | cells grown on F/2 | UTEX LB 1998 | N. Clarke | 9 | 111 | 80376 | Trebouxiophyceae | K | |
| MNCB | Green Algae | Chlorellales | Oocystaceae | Eremosphaera viridis | algal cells | M0511b | M. Melkonian | | 112 | 143656 | Trebouxiophyceae | K | |
| MFYC | Green Algae | Chlorellales | Chlorellaceae | Nannochloris atomus | algal cells | SAG 14.87 | M. Melkonian | | 113 | 32696 | Trebouxiophyceae | K | |
| HHXJ | Green Algae | Chlorodendrales | Chlorodendracea | Tetraselmis striata | algal cells | PLY 443 | M. Melkonian | | 114 | 42711 | Chlorodendrophyceae | L | |
| HVNO | Green Algae | Chlorodendrales | Chlorodendracea | Tetraselmis chui | cells grown on F/2 | UTEX LB 232 | N. Clarke | | 115 | 66049 | Chlorodendrophyceae | L | |
| DUMA | Green Algae | Chlorodendrales | Chlorodendracea | Tetraselmis cordiformis | algal cells | CCAC 0051 (M1608/1) | M. Melkonian | | 116 | 36828 | Chlorodendrophyceae | L | |
| FMVB | Green Algae | Chlorodendrales | Chlorodendracea | Scherffelia dubia | algal cells | CCAC 0019 (M0795) | M. Melkonian | | 117 | 38398 | Chlorodendrophyceae | L | |
| PUAN | Green Algae | Pedinomonadales | Pedinomonadace | Pedinomonas tuberculata | algal cells | CCAP 1965/2 | M. Melkonian | | 118 | 22957 | Pedinophyceae | L | |
| RRSV | Green Algae | Pedinomonadales | Pedinomonadace | Pedinomonas minor | algal cells | SAG 1965-3 | M. Melkonian | | 119 | 23185 | Pedinophyceae | L | |
| AYPS | Green Algae | incertae sedis | incertae sedis | unidentified species | algal cells | CCMP 1205 | M. Melkonian | | 120 | 11362 | | L | |
| TGNL | Green Algae | incertae sedis | incertae sedis | Picocystis salinarum | algal cells | CCMP 1897 | M. Melkonian | | 121 | 9806 | | L | |
| MMKU | Green Algae | Nephroselmidales | Nephroselmidac | Nephroselmis olivacea | algal cells | CCAC 0105 | M. Melkonian | | 122 | 51161 | Nephrophyceae | L | + |
| ISIM | Green Algae | Nephroselmidales | Nephroselmidac | Nephroselmis pyriformis | algal cells | CCMP 717 | M. Melkonian | | 123 | 37155 | Nephrophyceae | L | + |
| MCPK | Green Algae | Mamiellales | Mamiellaceae | Bathycoccus prasinos | algal cells | SCCAP K-0417 | M. Melkonian | | 124 | 15089 | Mamiellophyceae | M | + |
| QXSZ | Green Algae | Mamiellales | Mamiellaceae | Mantoniella squamata | algal cells | CCAP 1965/1 | M. Melkonian | | 125 | 43084 | Mamiellophyceae | M | + |
| BTFM | Green Algae | Mamiellales | Monomasticacea | Monomastix opisthostigma | algal cells | CCAC 0206 (M2844/1) | M. Melkonian | | 126 | 51094 | Mamiellophyceae | M | + |
| XOAL | Green Algae | Dolichomastigales | Dolichomastigac | Dolichomastix tenuilepis | algal cells | M1680 | M. Melkonian | | 127 | 43561 | Mamiellophyceae | M | + |
| TNAW | Green Algae | Pyramimonadales | Pyramimonadace | Pyramimonas parkeae | algal cells | CCAC 0082 (M1663/1) | M. Melkonian | | 128 | 78788 | Pyramimonadophyceae | M | + |
| JMTE | Green Algae | Pseudoscourfieldiale | Pycnococcaceae | Pseudoscourfieldia marina | algal cells | SCCAP K-0017 | M. Melkonian | | 129 | 18581 | Pyramimonadophyceae | M | + |
| EGNB | Green Algae | Scourfieldiales | Scourfieldiaceae | Scourfieldia sp. | algal cells | M0560/2 | M. Melkonian | | 130 | 30256 | Pedinophyceae | M | |
| HYHN | Green Algae | Prasinococcales | Prasinococcacea | Prasinoderma coloniale | algal cells | CCMP 1413 | M. Melkonian | | 131 | 16559 | | | |
| XMCL | Green Algae | Prasinococcales | Prasinococcacea | Prasinococcus capsulatus | algal cells | CCMP 1194 | M. Melkonian | | 132 | 16474 | | | |
| AZZW | Green Algae | Klebsormidiales | Klebsormidiacea | Chlorokybus atmophyticus | clonal culture, veg | M 2923 | M. Melkonian | | 133 | 21920 | | | |
| NNHQ | Green Algae | Zygnematales | Mesotaeniaceae | Spirotaenia minuta | clonal culture, veg | M 2661 | M. Melkonian | | 134 | 34193 | | | |
| KYIO | Green Algae | Mesostigmatales | Mesostigmatace | Mesostigma viride | clonal, axenic cult | SAG 50-1 | M. Melkonian | | 135 | 37189 | | | |
| FQLP | Green Algae | Klebsormidiales | Klebsormidiacea | Klebsormidium subtile | clonal, axenic cult | M 1894/1 (CCAC 0119) | M. Melkonian | | 136 | 33647 | | | |
| FPCO | Green Algae | Ulotrichales | Ulotrichaceae | Interfilum paradoxum | algal cells | SAG 338-1 | M. Melkonian | | 137 | 30434 | | | |
| BFIK | Green Algae | Klebsormidiales | Klebsormidiacea | Entransia fimbriata | algal cells | UTEX LB 2353 | M. Melkonian | | 138 | 31044 | | | |
| MWXT | Green Algae | Charales | Characeae | Chara vulgaris | clonal culture, veg | M 3183 | M. Melkonian | | 139 | 76087 | | | |
| VQBJ | Green Algae | Coleochaetales | Coleochaetaceae | Coleochaete scutata | clonal, axenic cult | SAG 110.80M (M 1311) | M. Melkonian | | 140 | 45338 | | | |
| QPDY | Green Algae | Coleochaetales | Coleochaetaceae | Coleochaete irregularis | algal cells | UTEX LB 2790 | M. Melkonian | | 141 | 31619 | | | |
| DRGY | Green Algae | Coleochaetales | Chaetosphaeridia | Chaetosphaeridium globosum | clonal, axenic cult | M 1311 (CCAC 0044) | M. Melkonian | | 142 | 37188 | | | |
| HAOX | Green Algae | Zygnematales | Zygnemataceae | Spirogyra sp. | clonal culture, veg | M 1810 | M. Melkonian | | 143 | 25273 | | | |
| WSJO | Green Algae | Zygnematales | Mesotaeniaceae | Mesotaenium braunii | algal cells | M2214 | M. Melkonian | | 144 | 49049 | | | |
| WDCW | Green Algae | Zygnematales | Mesotaeniaceae | Mesotaenium endlicherianum | algal cells | SAG 12.97 | M. Melkonian | | 145 | 48633 | | | |
| YOXI | Green Algae | Zygnematales | Mesotaeniaceae | Cylindrocystis brebissonii | algal cells | M2213 | M. Melkonian | | 146 | 56995 | | | |
| RPGL | Green Algae | Zygnematales | Mesotaeniaceae | Cylindrocystis brebissonii | algal cells | M2853 | M. Melkonian | | 147 | 114760 | | | |
| HKZW | Green Algae | Zygnematales | Mesotaeniaceae | Mesotaenium caldariorum | algal cells | SAG 648-1 | M. Melkonian | | 148 | 48636 | | | |
| JOJQ | Green Algae | Zygnematales | Zygnemataceae | Cylindrocystis cushleckae | algal cells | M2158 | M. Melkonian | | 149 | 41982 | | | |
| MFZO | Green Algae | Zygnematales | Zygnemataceae | Zygnemopsis sp. | algal cells | CCAP 699/1 | M. Melkonian | | 150 | 27548 | | | |
| FFGR | Green Algae | Zygnematales | Mesotaeniaceae | Netrium digitus | clonal, axenic cult | M 1322/1 (CCAC 0148) | M. Melkonian | | 151 | 47993 | | | |
| KMNX | Green Algae | Zygnematales | Mesotaeniaceae | Nucleotaenium eifelense | algal cells | M3006 | M. Melkonian | | 152 | 38154 | | | |
| SNOX | Green Algae | Zygnematales | Mesotaeniaceae | Planotaenium ohtanii | algal cells | M 2697 | M. Melkonian | | 153 | 24792 | | | |
| XRTZ | Green Algae | Zygnematales | Mesotaeniaceae | Roya obtusa | algal cells | M2403 | M. Melkonian | | 154 | 27653 | | | |
| KEYW | Green Algae | Zygnematales | Gonatozygaceae | Gonatozygon kinahanii | algal cells | M2935 | M. Melkonian | | 155 | 28183 | | | |
| DRFX | Green Algae | Zygnematales | Closteriaceae | Closterium lunula | algal cells | M2156 | M. Melkonian | | 156 | 81203 | | | |
| AEKF | Green Algae | Zygnematales | Peniaceae | Penium margaritaceum | algal cells | SAG 22.82 | M. Melkonian | | 157 | 48819 | | | |
| YSQT | Green Algae | Zygnematales | Peniaceae | Penium exiguum | algal cells | CCAC 0142 (M2159/1) | M. Melkonian | | 158 | 36931 | | | |
| RPQV | Green Algae | Zygnematales | Desmidiaceae | Phymatodocis nordstedtiana | algal cells | SVCK 327 | M. Melkonian | | 159 | 37309 | | | |
| HIDG | Green Algae | Zygnematales | Desmidiaceae | Cosmarium broomei | clonal, axenic cult | M 2075/1 (CCAC 0143) | M. Melkonian | 10 | 160 | 66229 | | | |
| RQFE | Green Algae | Zygnematales | Desmidiaceae | Cosmocladium cf. constrictum | algal cells | ASW 07118 (M1361) | M. Melkonian | | 161 | 38344 | | | |
| GYRP | Green Algae | Zygnematales | Desmidiaceae | Euastrum affine | algal cells | ASW 07012 | M. Melkonian | | 162 | 104323 | | | |
| MNNM | Green Algae | Zygnematales | Desmidiaceae | Cosmarium granatum | algal cells | CCAC 0137 (M2127/1) | M. Melkonian | | 163 | 44947 | | | |
| ISHC | Green Algae | Zygnematales | Desmidiaceae | Staurastrum sebaldi | algal cells | M1129 | M. Melkonian | | 164 | 46452 | | | |
| QWFV | Green Algae | Zygnematales | Desmidiaceae | Bambusina borreri | algal cells | CCAC 0045 (M1320) | M. Melkonian | | 165 | 83393 | | | |
| DFDS | Green Algae | Zygnematales | Desmidiaceae | Desmidium aptogonum | algal cells | ASW 07112 | M. Melkonian | | 166 | 41347 | | | |
| GGWH | Green Algae | Zygnematales | Desmidiaceae | Onychonema laeve | algal cells | CCAC 0151 (M2246/1) | M. Melkonian | | 167 | 49390 | | | |
| WCQU | Green Algae | Zygnematales | Desmidiaceae | Staurodesmus convergens | algal cells | M2558 | M. Melkonian | | 168 | 63206 | | | |
| BHBK | Green Algae | Zygnematales | Desmidiaceae | Cosmarium tinctum | algal cells | M2301 | M. Melkonian | | 169 | 51220 | | | |
| RPRU | Green Algae | Zygnematales | Desmidiaceae | Staurodesmus omearii | algal cells | M0751 | M. Melkonian | | 170 | 43362 | | | |
| MCHJ | Green Algae | Zygnematales | Desmidiaceae | Micrasterias fimbriata | algal cells | ASW 07026 (M1188) | M. Melkonian | | 171 | 64972 | | | |
| GBGT | Green Algae | Zygnematales | Desmidiaceae | Xanthidium antilopaeum | algal cells | M1229 | M. Melkonian | | 172 | 105757 | | | |
| MOYY | Green Algae | Zygnematales | Desmidiaceae | Pleurotaenium trabecula | algal cells | CCAC 0163 | M. Melkonian | | 173 | 99058 | | | |
| STKJ | Green Algae | Zygnematales | Desmidiaceae | Cosmarium ochthodes | algal cells | M1384 | M. Melkonian | | 174 | 99850 | | | |
| WDGV | Green Algae | Zygnematales | Desmidiaceae | Cosmarium subtumidum | algal cells | M3067 | M. Melkonian | | 175 | 56528 | | | |
| MXEZ | Green Algae | Pseudoscourfieldiale | Pycnococcaceae | Pycnococcus provasolii | algal cells | CCMP 1203 | M. Melkonian | | - | 15322 | | | |
| MJMQ | Chromista (Algae) | Pyrenomonadales | Chroomonadacea | Hemiselmis virescens | algal cells | M1635 | M. Melkonian | | - | 42834 | | | |
| YTYU | Glaucophyta (Algae | Glaucocystales | Glaucocystaceae | Cyanophora paradoxa CCAC 0091 (M | algal cells | CCAC 0091 M0507/1 | M. Melkonian | | - | 34058 | | | |
| DXNY | Green Algae | Microthamniales | Microthamniacea | Microthamnion kuetzingianum | algal cells | CCAC 0054 (M0423) | M. Melkonian | | - | 43557 | | | |
| AGIO | Green Algae | Volvocales | Haematococcace | Haematococcus pluvialis | algal cells | CCAC 0055 (M0716/1) | M. Melkonian | | - | 61126 | | | |
| ODXI | Green Algae | Volvocales | Haematococcace | Haematococcus pluvialis | algal cells (red) | CCAC 0055 | M. Melkonian | | - | 82149 | | | |
| NTLE | Green Algae | Volvocales | Asteromonadace | Asteromonas gracilis | algal cells | CCAC 0049 (M1491) | M. Melkonian | | - | 53714 | | | |
| HJVM | Green Algae | Zygnematales | Desmidiaceae | Cosmarium ochthodes | algal cells | ASW 07133 | M. Melkonian | | - | 111788 | | | |

Notes from OneKP site:

| | |
|---|---|
| 1 | weak RNA |
| 2 | originally submitted as D. primolecta, but appears to be identical to ZDIZ/CCMP 364 |
| 3 | Originally submitted as UTEX LB 1644 D. salina, but the sequence was matched to S. viridis |
| 4 | was Chloromonas perforata (see doi:10.1111/jpy.12343) |
| 5 | was Pleurastrum insigne (see doi: 10.1111/jpy.12343) |
| 6 | originally recorded as Hormidiella sp. |
| 7 | weak RNA |
| 8 | was synonym Neochloris oleoabundans |
| 9 | was originally Nannochloropsis oculata |
| 10 | resent; few big contigs |

| Supp. Table 2: | | | | -log(p-val)[3] | |
|---|---|---|---|---|---|
| HMM name[1] | functional group[2] | sub-group | | 5 major clades | most sig. Chlorophyte |
| ec2.7.1.19 | carbon assimilation | | | 0.3 | 1.6 |
| ec4.1.1.39 | carbon assimilation | | | 0.1 | 0.5 |
| ec4.1.2.13 | carbon assimilation | | | 0.0 | 0.8 |
| ec5.1.3.1 | carbon assimilation | | | 0.0 | 0.0 |
| ec5.3.1.6 | carbon assimilation | | | 0.1 | 1.2 |
| PF00016 | carbon assimilation | | | 0.0 | 0.8 |
| PF00101 | carbon assimilation | | | 0.2 | 0.5 |
| PF00194 | carbon assimilation | | | 6.6 | 1.6 |
| PF00484 | carbon assimilation | | | 11.3 | 1.0 |
| PF02788 | carbon assimilation | | | 0.9 | 0.5 |
| Rubisco_activase | carbon assimilation | | | 33.0 | 0.0 |
| ec1.1.1.157 | central carbon | | | 0.6 | 0.3 |
| ec1.1.1.30 | central carbon | | | 0.0 | 0.0 |
| ec1.1.1.37 | central carbon | | | 0.0 | 0.0 |
| ec1.1.1.38 | central carbon | | | 0.0 | 0.8 |
| ec1.1.1.40 | central carbon | | | 0.2 | 0.9 |
| ec1.1.5.3 | central carbon | | | 0.3 | 0.9 |
| ec1.2.1.12 | central carbon | | | 0.0 | 0.0 |
| ec1.2.1.13 | central carbon | | | 0.4 | 0.0 |
| ec1.2.1.9 | central carbon | | | 0.4 | 0.9 |
| ec2.3.1.9 | central carbon | | | 0.0 | 0.9 |
| ec2.3.3.1 | central carbon | | | 0.5 | 0.9 |
| ec2.3.3.9 | central carbon | | | 11.4 | 6.3 |
| ec2.7.2.3 | central carbon | | | 0.1 | 0.6 |
| ec2.7.9.1 | central carbon | | | 0.8 | 4.0 |
| ec3.1.3.11 | central carbon | | | 0.0 | 0.9 |
| ec3.1.3.37 | central carbon | | | 0.6 | 0.9 |
| ec4.1.1.3 | central carbon | | | 20.0 | 2.1 |
| ec4.1.1.32 | central carbon | | | 6.9 | 0.8 |
| ec4.1.1.49 | central carbon | | | 8.3 | 1.9 |
| ec4.1.3.1 | central carbon | | | 8.9 | 6.3 |
| ec5.3.1.1 | central carbon | | | 0.4 | 0.9 |
| ec6.2.1.1 | central carbon | | | 0.0 | 0.6 |
| ec6.4.1.3 | central carbon | | | 0.3 | 1.2 |
| ec6.4.1.4 | central carbon | | | 0.0 | 0.9 |
| oxoglutarate-malate transporter | central carbon | | | 30.2 | 0.9 |
| ec1.1.1.101 | mebrane lipid | backbone | | 0.9 | 0.8 |
| ec1.1.1.94 | mebrane lipid | backbone | | 3.9 | 1.0 |
| ec2.3.1.23 | mebrane lipid | backbone | | 1.5 | 1.3 |
| ec2.3.1.51 | mebrane lipid | backbone | | 0.0 | 0.9 |
| ec2.7.1.107 | mebrane lipid | backbone | | 6.4 | 1.8 |
| ec2.7.1.30 | mebrane lipid | backbone | | 0.1 | 0.7 |
| ec2.7.7.41 | mebrane lipid | backbone | | 0.2 | 0.9 |
| ec2.7.8.5 | membrane lipid | backbone | | 1.1 | 0.7 |
| ec3.1.1.5 | membrane lipid | backbone | | 0.8 | 0.7 |
| ec3.1.3.27 | membrane lipid | backbone | | 0.0 | 0.9 |
| ec3.1.3.4 | membrane lipid | backbone | | 3.5 | 1.9 |
| ec3.1.4.3 | membrane lipid | backbone | | 9.1 | 0.7 |
| PF03062 | membrane lipid | backbone | | 0.9 | 0.9 |
| acyl carrier protein | membrane lipid | fatty acid | | 0.0 | 0.8 |
| ec1.1.1.100 | membrane lipid | fatty acid | | 0.0 | 0.0 |
| ec1.14.19.1 | membrane lipid | fatty acid | | 2.5 | 0.6 |
| ec1.14.19.2 | membrane lipid | fatty acid | | 26.2 | 0.0 |
| ec1.14.19.3 | membrane lipid | fatty acid | | 3.2 | 1.7 |
| ec1.14.19.4 | membrane lipid | fatty acid | | 11.6 | 1.9 |
| ec1.14.99.33 | membrane lipid | fatty acid | | 0.6 | 1.0 |

| | | | | | |
|---|---|---|---|---|---|
| ec1.3.1.38 | membrane lipid | fatty acid | | 1.6 | 1.5 |
| ec1.3.1.9 | membrane lipid | fatty acid | | 1.1 | 0.6 |
| ec1.3.3.6 | membrane lipid | fatty acid | | 5.5 | 0.9 |
| ec1.3.99.3 | membrane lipid | fatty acid | | 0.3 | 0.8 |
| ec2.3.1.16 | membrane lipid | fatty acid | | 0.0 | 0.9 |
| ec2.3.1.179 | membrane lipid | fatty acid | | 0.0 | 0.0 |
| ec2.3.1.180 | membrane lipid | fatty acid | | 6.7 | 0.6 |
| ec2.3.1.181 | membrane lipid | fatty acid | | 0.7 | 1.7 |
| ec2.3.1.39 | membrane lipid | fatty acid | | 0.4 | 0.6 |
| ec3.1.2.14 | membrane lipid | fatty acid | | 12.2 | 2.1 |
| ec3.1.2.20 | membrane lipid | fatty acid | | 2.5 | 1.8 |
| ec3.1.2.22 | membrane lipid | fatty acid | | 15.1 | 0.3 |
| ec4.2.1.17 | membrane lipid | fatty acid | | 0.0 | 0.4 |
| ec4.2.1.60 | membrane lipid | fatty acid | | 0.5 | 0.5 |
| ec6.2.1.3 | membrane lipid | fatty acid | | 0.0 | 0.0 |
| ec6.4.1.2 | membrane lipid | fatty acid | | 0.0 | 0.9 |
| ec2.1.1.103 | membrane lipid | headgroup | | 2.5 | 4.7 |
| ec2.3.1.50 | membrane lipid | headgroup | | 0.5 | 0.9 |
| ec2.7.1.32 | membrane lipid | headgroup | | 3.1 | 1.8 |
| ec2.7.1.82 | membrane lipid | headgroup | | 4.1 | 1.8 |
| ec2.7.7.14 | membrane lipid | headgroup | | 1.4 | 1.6 |
| ec2.7.7.15 | membrane lipid | headgroup | | 4.9 | 1.4 |
| ec2.7.8.1 | membrane lipid | headgroup | | 2.2 | 1.1 |
| ec2.7.8.11 | membrane lipid | headgroup | | 0.3 | 0.5 |
| ec2.7.8.2 | membrane lipid | headgroup | | 2.1 | 1.1 |
| ec2.7.8.24 | membrane lipid | headgroup | | 17.4 | 0.6 |
| ec3.1.3.75 | membrane lipid | headgroup | | 4.0 | 2.6 |
| ec3.13.1.1 | membrane lipid | headgroup | | 0.0 | 0.9 |
| PF04191 | membrane lipid | headgroup | | 22.3 | 0.8 |
| PF11899 | membrane lipid | headgroup | | 12.1 | 3.5 |
| ec2.4.1.241 | membrane lipid | headgroup | | 2.9 | 1.9 |
| ec2.4.1.46 | membrane lipid | headgroup | | 0.1 | 2.1 |
| ec2.3.1.158 | triacylglcyeride | | | 1.9 | 1.5 |
| ec2.3.1.20 | triacylglcyeride | | | 2.9 | 2.1 |
| ec3.1.1.3 | triacylglcyeride | | | 0.1 | 1.2 |
| PF03982 | triacylglcyeride | | | 8.1 | 1.6 |
| *ec2.5.1.1* | terpenoid | downstream from IPP | | 0.4 | 0.0 |
| ec2.5.1.10 | terpenoid | downstream from IPP | | 0.4 | 0.0 |
| ec2.5.1.29 | terpenoid | downstream from IPP | | 0.2 | 0.4 |
| ec2.5.1.32 | terpenoid | downstream from IPP | | 0.0 | 0.6 |
| ec5.3.3.2 | terpenoid | downstream from IPP | | 0.6 | 1.6 |
| ec1.1.1.267 | terpenoid | MEP/DOXP | | 0.6 | 0.8 |
| ec1.17.1.2 | terpenoid | MEP/DOXP | | 0.2 | 0.9 |
| ec1.17.7.1 | terpenoid | MEP/DOXP | | 0.3 | 1.2 |
| ec2.2.1.7 | terpenoid | MEP/DOXP | | 0.2 | 0.6 |
| ec2.7.1.148 | terpenoid | MEP/DOXP | | 1.9 | 0.7 |
| ec2.7.7.60 | terpenoid | MEP/DOXP | | 14.5 | 0.5 |
| ec4.6.1.12 | terpenoid | MEP/DOXP | | 2.0 | 0.0 |
| ec1.1.1.34 | terpenoid | MVA | | 18.2 | 0.9 |
| ec1.1.1.88 | terpenoid | MVA | | 1.1 | 0.0 |
| ec2.3.3.10 | terpenoid | MVA | | 10.0 | 4.5 |
| ec2.7.1.36 | terpenoid | MVA | | 1.0 | 0.8 |
| ec2.7.4.2 | terpenoid | MVA | | 13.7 | 0.8 |
| ec4.1.1.33 | terpenoid | MVA | | 28.1 | 1.4 |
| ec2.1.1.14 | vitamin | B12 | | 1.4 | 2.0 |
| PF00809 | vitamin | B12 | | 0.2 | 1.1 |
| PF01717 | vitamin | B12 | | 1.5 | 2.3 |
| PF02310 | vitamin | B12 | | 0.4 | 1.5 |
| PF02574 | vitamin | B12 | | 0.2 | 0.4 |
| PF02607 | vitamin | B12 | | 0.1 | 0.9 |
| PF02965 | vitamin | B12 | | 0.1 | 1.2 |

| | | | | | |
|---|---|---|---|---|---|
| PF08267 | vitamin | B12 | | 1.5 | 1.5 |
| ec1.1.1.65 | vitamin | B6 | | 0.2 | 0.4 |
| ec1.2.1.72 | vitamin | B6 | | 0.2 | 0.9 |
| ec1.4.3.5 | vitamin | B6 | | 5.3 | 4.3 |
| ec2.3.1.47 | vitamin | B6 | | 0.0 | 0.9 |
| ec2.6.1.52 | vitamin | B6 | | 0.0 | 0.9 |
| ec2.7.1.35 | vitamin | B6 | | 0.9 | 1.2 |
| ec3.1.3.74 | vitamin | B6 | | 1.7 | 0.8 |
| ec4.2.3.1 | vitamin | B6 | | 0.0 | 0.6 |
| PF01174 | vitamin | B6 | | 0.4 | 1.0 |
| ec2.6.1.62 | vitamin | biotin | | 1.0 | 0.7 |
| ec2.8.1.6 | vitamin | biotin | | 0.6 | 0.6 |
| ec6.2.1.14 | vitamin | biotin | | 5.3 | 1.5 |
| ec6.3.3.3 | vitamin | biotin | | 2.0 | 1.2 |
| ec6.3.4.10 | vitamin | biotin | | 3.6 | 3.0 |
| ec6.3.4.11 | vitamin | biotin | | 3.6 | 3.0 |
| ec6.3.4.15 | vitamin | biotin | | 0.8 | 1.4 |
| ec6.3.4.9 | vitamin | biotin | | 3.6 | 3.0 |
| ec1.4.3.19 | vitamin | thiamine | | 0.7 | 2.3 |
| ec2.5.1.3 | vitamin | thiamine | | 0.5 | 0.8 |
| ec2.7.1.49 | vitamin | thiamine | | 1.6 | 1.2 |
| ec2.7.1.50 | vitamin | thiamine | | 6.3 | 2.3 |
| ec2.7.4.16 | vitamin | thiamine | | 0.3 | 1.2 |
| ec2.7.4.7 | vitamin | thiamine | | 1.8 | 1.2 |
| ec2.7.6.2 | vitamin | thiamine | | 0.7 | 0.9 |
| ec2.8.1.7 | vitamin | thiamine | | 0.0 | 0.9 |
| ec3.5.99.2 | vitamin | thiamine | | 3.6 | 2.2 |
| ec3.6.1.15 | vitamin | thiamine | | 0.2 | 1.2 |
| ec3.6.1.28 | vitamin | thiamine | | 7.0 | 3.0 |
| PF00899 | vitamin | thiamine | | 0.0 | 0.0 |
| PF01946 | vitamin | thiamine | | 10.5 | 1.1 |
| PF01964 | vitamin | thiamine | | 4.4 | 2.6 |
| PF02110 | vitamin | thiamine | | 4.9 | 3.4 |
| PF00636 | misc | RNA silencing | | 2.2 | 1.8 |
| PF02170 | misc | RNA silencing | | 4.9 | 1.1 |
| PF02171 | misc | RNA silencing | | 4.7 | 0.9 |
| PF03368 | misc | RNA silencing | | 1.0 | 1.3 |
| PF05183 | misc | RNA silencing | | 0.2 | 0.6 |
| PF02301 | misc | meiosis | | 1.2 | 1.3 |
| PF03962 | misc | meiosis | | 1.8 | 2.0 |
| PF07106 | misc | meiosis | | 0.8 | 1.3 |
| SPO11 | misc | meiosis | | 8.8 | 1.5 |
| PF00078 | misc | misc | | 3.3 | 0.7 |
| PF00270 | misc | misc | | 0.0 | 0.0 |
| PF00271 | misc | misc | | 0.0 | 0.0 |
| PF00493 | misc | misc | | 0.1 | 1.1 |
| PF02889 | misc | misc | | 17.0 | 0.8 |
| PF08241 | misc | misc | | 1.1 | 0.0 |
| PF08423 | misc | misc | | 4.2 | 1.3 |
| PF13489 | misc | misc | | 1.1 | 0.0 |

1: Pfam code:name or EC number from which HMM was generated; for the four HMM names that are not prefaced by PF (Pfam) or ec (constructed by us from KEGG equence alignments for that EC number) the HMM was constructed from sequence alignments obtained using PSI-BLAST, from sequences described in the text

2: Thes are subjective and in some cases fairly arbitrary categories

3: These are the Fisher exact test p-values for the distribution of HMM hits across the five major clades or the best of the branches along the Chlorophyte lineage. Coloring is from white (p-val = 1) 0 to darkest green (p-val is the lowest in that column. Colors are interpolated by Excel based on the values shown in the cells.

# Supplementary Table S3 (pg 1/4, upper left quadrant)

| sample code | ec2.7.1.19 | ec4.1.1.39 | ec4.1.2.13 | ec5.1.3.1 | ec5.3.1.6 | PF00016 | PF00101 | PF00194 | PF00484 | PF02788 | Rubisco_activase | ec1.1.1.157 | ec1.1.1.30 | ec1.1.1.37 | ec1.1.1.38 | ec1.1.1.40 | ec1.1.5.3 | ec1.2.1.12 | ec1.2.1.13 | ec1.2.1.9 | ec2.3.1.9 | ec2.3.3.1 | ec2.3.3.9 | ec2.7.2.3 | ec2.7.9.1 | ec3.1.3.11 | ec3.1.3.37 | ec4.1.1.3 | ec4.1.1.32 | ec4.1.1.49 | ec4.1.3.1 | ec5.3.1.1 | ec6.2.1.1 | ec6.4.1.3 | ec6.4.1.4 | oxoglutarate-malate transpo | ec1.1.1.101 | ec1.1.1.94 | ec2.3.1.23 | ec2.3.1.51 | ec2.7.1.107 | ec2.7.1.30 | ec2.7.7.41 | ec2.7.8.5 | ec3.1.1.5 | ec3.1.3.27 | ec3.1.3.4 | ec3.1.4.3 | PF03062 | acyl carrier protein | ec1.1.1.100 | ec1.14.19.1 | ec1.14.19.2 | ec1.14.19.3 | ec1.14.19.4 | ec1.14.99.33 | ec1.3.1.38 | ec1.3.1.9 | ec1.3.3.6 | ec1.3.99.3 | ec2.3.1.16 | ec2.3.1.179 | ec2.3.1.180 | ec2.3.1.181 | ec2.3.1.39 | ec3.1.2.14 | ec3.1.2.20 | ec3.1.2.22 | ec4.2.1.17 | ec4.2.1.60 | ec6.2.1.3 | ec6.4.1.2 | ec2.1.1.103 | ec2.3.1.50 | ec2.7.1.32 | ec2.7.1.82 | ec2.7.7.14 | ec2.7.7.15 | ec2.7.8.1 | ec2.7.8.11 | ec2.7.8.2 | ec2.7.8.24 | ec3.1.3.75 | ec3.13.1.1 |
|---|---|---|---|---|---|---|---|---|---|---|---|---|---|---|---|---|---|---|---|---|---|---|---|---|---|---|---|---|---|---|---|---|---|---|---|---|---|---|---|---|---|---|---|---|---|---|---|---|---|---|---|---|---|---|---|---|---|---|---|---|---|---|---|---|---|---|---|---|---|---|---|---|---|---|---|---|---|---|---|---|---|---|---|---|
| ROZZ | 1 | 1 | 4 | 2 | 2 | 0 | 0 | 6 | 10 | 1 | 1 | 2 | 8 | 3 | 5 | 3 | 2 | 6 | 2 | 2 | 3 | 1 | 1 | 4 | 1 | 4 | 3 | 0 | 0 | 4 | 1 | 4 | 2 | 2 | 1 | 0 | 0 | 3 | 1 | 4 | 0 | 1 | 1 | 2 | 1 | 0 | 0 | 0 | 1 | 1 | 18 | 0 | 0 | 1 | 1 | 2 | 1 | 2 | 0 | 2 | 3 | 4 | 0 | 0 | 1 | 0 | 1 | 0 | 7 | 0 | 7 | 1 | 0 | 2 | 1 | 1 | 1 | 0 | 0 | 0 | 0 | 0 | 0 | 1 |
| BAKF | 1 | 1 | 4 | 2 | 2 | 1 | 1 | 0 | 2 | 1 | 2 | 2 | 9 | 3 | 2 | 1 | 1 | 2 | 1 | 3 | 2 | 1 | 1 | 4 | 1 | 5 | 3 | 0 | 1 | 0 | 1 | 5 | 3 | 1 | 1 | 0 | 0 | 1 | 1 | 7 | 0 | 1 | 0 | 0 | 0 | 0 | 1 | 0 | 1 | 3 | 21 | 0 | 0 | 0 | 0 | 2 | 1 | 0 | 0 | 5 | 2 | 1 | 1 | 0 | 1 | 0 | 0 | 0 | 6 | 0 | 3 | 1 | 0 | 2 | 0 | 0 | 0 | 0 | 0 | 1 | 0 | 0 | 0 | 1 |
| IRZA | 1 | 0 | 4 | 3 | 1 | 0 | 0 | 3 | 9 | 0 | 1 | 0 | 7 | 4 | 3 | 3 | 2 | 3 | 3 | 5 | 2 | 0 | 1 | 4 | 1 | 4 | 2 | 0 | 0 | 2 | 1 | 3 | 2 | 2 | 3 | 0 | 1 | 0 | 1 | 10 | 1 | 0 | 1 | 1 | 2 | 0 | 2 | 0 | 2 | 2 | 26 | 0 | 0 | 0 | 1 | 1 | 1 | 2 | 0 | 4 | 2 | 5 | 1 | 0 | 1 | 0 | 0 | 1 | 2 | 0 | 11 | 3 | 0 | 3 | 0 | 0 | 1 | 1 | 0 | 1 | 0 | 0 | 0 | 1 |
| IAYV | 1 | 4 | 4 | 3 | 1 | 3 | 3 | 7 | 11 | 2 | 1 | 2 | 8 | 4 | 3 | 1 | 1 | 8 | 3 | 2 | 4 | 1 | 1 | 2 | 2 | 5 | 2 | 1 | 0 | 2 | 1 | 4 | 1 | 2 | 3 | 0 | 0 | 1 | 1 | 5 | 0 | 1 | 1 | 1 | 4 | 0 | 0 | 0 | 2 | 1 | 16 | 0 | 0 | 0 | 1 | 2 | 1 | 1 | 0 | 3 | 4 | 1 | 1 | 1 | 0 | 0 | 0 | 1 | 4 | 0 | 6 | 1 | 0 | 2 | 0 | 0 | 0 | 0 | 1 | 0 | 1 | 0 | 0 | 1 |
| BAJW | 2 | 2 | 5 | 5 | 2 | 1 | 3 | 1 | 11 | 1 | 1 | 3 | 16 | 3 | 3 | 3 | 0 | 9 | 7 | 9 | 5 | 2 | 1 | 5 | 0 | 4 | 4 | 2 | 0 | 1 | 1 | 2 | 6 | 7 | 6 | 0 | 0 | 5 | 0 | 6 | 1 | 2 | 2 | 2 | 2 | 0 | 1 | 1 | 2 | 2 | 38 | 1 | 1 | 0 | 1 | 2 | 0 | 1 | 1 | 12 | 5 | 4 | 1 | 2 | 3 | 0 | 1 | 1 | 11 | 0 | 16 | 6 | 0 | 3 | 0 | 0 | 1 | 0 | 0 | 1 | 0 | 0 | 0 | 1 |
| LXRN | 2 | 3 | 5 | 3 | 1 | 2 | 3 | 0 | 9 | 2 | 2 | 3 | 13 | 4 | 1 | 1 | 1 | 7 | 5 | 3 | 4 | 2 | 1 | 4 | 0 | 4 | 2 | 3 | 0 | 1 | 1 | 3 | 3 | 6 | 5 | 0 | 0 | 4 | 0 | 5 | 0 | 1 | 1 | 0 | 0 | 0 | 0 | 1 | 1 | 2 | 29 | 1 | 1 | 0 | 1 | 1 | 1 | 1 | 1 | 11 | 4 | 5 | 2 | 1 | 7 | 0 | 0 | 2 | 9 | 1 | 8 | 4 | 0 | 0 | 0 | 0 | 1 | 0 | 0 | 1 | 0 | 0 | 0 | 1 |
| EBWI | 1 | 1 | 3 | 2 | 1 | 0 | 2 | 4 | 7 | 0 | 1 | 1 | 19 | 5 | 3 | 3 | 3 | 4 | 2 | 6 | 5 | 1 | 1 | 3 | 0 | 5 | 3 | 1 | 0 | 2 | 1 | 4 | 3 | 3 | 1 | 0 | 2 | 3 | 1 | 2 | 1 | 1 | 1 | 0 | 6 | 0 | 0 | 0 | 1 | 2 | 40 | 1 | 1 | 0 | 1 | 1 | 0 | 0 | 0 | 4 | 5 | 2 | 1 | 0 | 1 | 0 | 0 | 0 | 8 | 0 | 6 | 2 | 0 | 2 | 0 | 0 | 1 | 1 | 0 | 1 | 0 | 0 | 0 | 1 |
| RFAD | 1 | 2 | 4 | 3 | 1 | 2 | 2 | 0 | 17 | 2 | 2 | 1 | 12 | 4 | 2 | 1 | 2 | 5 | 3 | 5 | 6 | 1 | 2 | 2 | 0 | 4 | 4 | 2 | 0 | 4 | 2 | 5 | 1 | 5 | 4 | 0 | 0 | 4 | 0 | 3 | 0 | 1 | 2 | 0 | 3 | 0 | 0 | 1 | 0 | 2 | 31 | 1 | 1 | 1 | 0 | 2 | 0 | 1 | 0 | 3 | 6 | 2 | 2 | 1 | 1 | 0 | 0 | 1 | 7 | 0 | 4 | 5 | 0 | 1 | 0 | 0 | 0 | 0 | 0 | 1 | 0 | 0 | 0 | 1 |
| LLEN | 2 | 2 | 6 | 4 | 2 | 2 | 3 | 0 | 12 | 2 | 2 | 2 | 16 | 8 | 2 | 2 | 2 | 11 | 6 | 5 | 5 | 1 | 1 | 3 | 0 | 6 | 5 | 1 | 0 | 1 | 2 | 8 | 2 | 4 | 3 | 0 | 1 | 4 | 1 | 6 | 0 | 1 | 2 | 1 | 2 | 0 | 0 | 1 | 1 | 2 | 35 | 1 | 1 | 1 | 0 | 2 | 0 | 1 | 1 | 3 | 5 | 1 | 1 | 2 | 1 | 0 | 0 | 1 | 8 | 0 | 5 | 3 | 1 | 1 | 0 | 0 | 1 | 0 | 0 | 1 | 0 | 0 | 0 | 1 |
| NMAK | 1 | 3 | 4 | 3 | 1 | 1 | 3 | 0 | 5 | 1 | 2 | 2 | 20 | 5 | 2 | 2 | 2 | 7 | 4 | 6 | 5 | 1 | 1 | 2 | 0 | 4 | 4 | 2 | 0 | 1 | 1 | 4 | 2 | 5 | 4 | 0 | 2 | 4 | 2 | 8 | 1 | 1 | 2 | 2 | 4 | 0 | 0 | 1 | 1 | 2 | 42 | 1 | 1 | 1 | 1 | 2 | 0 | 1 | 1 | 6 | 5 | 1 | 2 | 2 | 1 | 1 | 0 | 2 | 9 | 0 | 7 | 5 | 0 | 2 | 0 | 0 | 1 | 1 | 0 | 1 | 0 | 0 | 0 | 1 |
| BOGT | 1 | 1 | 2 | 3 | 1 | 1 | 1 | 0 | 15 | 1 | 1 | 2 | 9 | 2 | 2 | 1 | 1 | 2 | 2 | 6 | 2 | 1 | 0 | 3 | 5 | 7 | 4 | 2 | 0 | 0 | 0 | 3 | 4 | 1 | 1 | 0 | 2 | 3 | 1 | 8 | 0 | 1 | 2 | 2 | 3 | 0 | 0 | 1 | 2 | 2 | 19 | 1 | 0 | 0 | 0 | 2 | 1 | 1 | 0 | 3 | 2 | 3 | 1 | 2 | 1 | 0 | 1 | 0 | 6 | 0 | 9 | 3 | 0 | 2 | 0 | 0 | 1 | 1 | 1 | 1 | 1 | 0 | 0 | 1 |
| RAPY | 1 | 3 | 6 | 2 | 2 | 1 | 3 | 3 | 5 | 1 | 2 | 3 | 12 | 4 | 2 | 2 | 2 | 11 | 6 | 6 | 4 | 2 | 0 | 4 | 1 | 6 | 3 | 2 | 0 | 4 | 0 | 4 | 1 | 4 | 5 | 0 | 1 | 2 | 2 | 9 | 1 | 1 | 1 | 3 | 0 | 0 | 1 | 0 | 2 | 4 | 27 | 2 | 0 | 1 | 1 | 4 | 1 | 2 | 2 | 6 | 4 | 3 | 1 | 0 | 1 | 1 | 0 | 0 | 10 | 0 | 9 | 4 | 0 | 2 | 3 | 3 | 2 | 2 | 2 | 2 | 2 | 0 | 1 | 1 |
| LLXJ | 1 | 1 | 6 | 3 | 2 | 0 | 2 | 4 | 7 | 1 | 1 | 5 | 17 | 9 | 2 | 1 | 2 | 3 | 3 | 10 | 7 | 5 | 0 | 2 | 1 | 12 | 5 | 2 | 1 | 1 | 0 | 4 | 5 | 7 | 4 | 0 | 1 | 1 | 3 | 8 | 2 | 1 | 3 | 3 | 4 | 0 | 2 | 0 | 3 | 5 | 34 | 3 | 0 | 1 | 1 | 0 | 1 | 1 | 2 | 10 | 5 | 4 | 0 | 2 | 1 | 0 | 1 | 1 | 7 | 0 | 12 | 3 | 0 | 4 | 2 | 2 | 2 | 2 | 3 | 1 | 3 | 0 | 0 | 1 |
| JJZR | 1 | 1 | 4 | 2 | 1 | 1 | 1 | 0 | 4 | 1 | 2 | 2 | 23 | 4 | 1 | 1 | 1 | 4 | 4 | 6 | 2 | 5 | 0 | 2 | 0 | 7 | 3 | 1 | 0 | 2 | 0 | 2 | 4 | 3 | 4 | 0 | 3 | 2 | 4 | 8 | 2 | 1 | 2 | 4 | 6 | 0 | 1 | 0 | 3 | 1 | 32 | 2 | 0 | 0 | 0 | 2 | 0 | 1 | 3 | 5 | 2 | 2 | 0 | 2 | 1 | 0 | 1 | 0 | 5 | 0 | 10 | 4 | 0 | 2 | 1 | 1 | 1 | 1 | 1 | 1 | 1 | 1 | 0 | 1 |
| RSOF | 2 | 2 | 3 | 2 | 1 | 1 | 3 | 4 | 4 | 2 | 3 | 3 | 7 | 3 | 2 | 2 | 0 | 5 | 2 | 2 | 2 | 4 | 0 | 2 | 2 | 5 | 2 | 2 | 1 | 0 | 0 | 2 | 3 | 1 | 2 | 0 | 0 | 1 | 4 | 6 | 1 | 0 | 2 | 1 | 2 | 0 | 1 | 0 | 2 | 2 | 13 | 3 | 0 | 0 | 0 | 1 | 0 | 1 | 4 | 0 | 1 | 2 | 1 | 2 | 1 | 0 | 1 | 0 | 6 | 0 | 8 | 2 | 0 | 3 | 1 | 1 | 2 | 1 | 1 | 1 | 0 | 1 | 1 | 2 |
| PVGP | 1 | 2 | 2 | 2 | 1 | 1 | 2 | 0 | 1 | 0 | 1 | 2 | 2 | 1 | 2 | 3 | 0 | 3 | 1 | 3 | 3 | 2 | 0 | 2 | 0 | 4 | 4 | 1 | 1 | 0 | 0 | 0 | 3 | 5 | 4 | 0 | 0 | 1 | 1 | 6 | 1 | 0 | 2 | 0 | 2 | 0 | 1 | 0 | 2 | 2 | 5 | 2 | 0 | 0 | 0 | 2 | 0 | 0 | 2 | 4 | 3 | 2 | 0 | 1 | 0 | 0 | 1 | 0 | 6 | 0 | 4 | 4 | 0 | 2 | 0 | 0 | 1 | 1 | 0 | 0 | 0 | 0 | 0 | 1 |
| OBUY | 1 | 4 | 3 | 2 | 1 | 1 | 4 | 0 | 4 | 1 | 1 | 2 | 8 | 2 | 2 | 2 | 0 | 5 | 2 | 2 | 2 | 3 | 0 | 2 | 0 | 2 | 3 | 1 | 3 | 0 | 0 | 1 | 3 | 2 | 1 | 0 | 0 | 1 | 1 | 4 | 1 | 0 | 2 | 2 | 1 | 0 | 1 | 0 | 2 | 3 | 12 | 2 | 0 | 0 | 0 | 1 | 0 | 1 | 2 | 4 | 2 | 2 | 0 | 1 | 1 | 0 | 1 | 0 | 4 | 0 | 7 | 1 | 0 | 2 | 0 | 1 | 1 | 0 | 0 | 1 | 0 | 1 | 1 | 1 |
| ZULJ | 2 | 2 | 7 | 2 | 1 | 1 | 2 | 2 | 12 | 1 | 1 | 2 | 10 | 8 | 3 | 1 | 1 | 8 | 6 | 0 | 4 | 5 | 0 | 6 | 0 | 8 | 3 | 1 | 0 | 5 | 2 | 3 | 3 | 4 | 4 | 0 | 0 | 1 | 2 | 7 | 0 | 1 | 3 | 0 | 2 | 0 | 1 | 0 | 1 | 5 | 18 | 1 | 0 | 0 | 0 | 2 | 1 | 2 | 6 | 8 | 4 | 1 | 0 | 0 | 0 | 0 | 1 | 2 | 6 | 0 | 9 | 4 | 0 | 2 | 1 | 1 | 2 | 3 | 2 | 2 | 1 | 1 | 0 | 1 |
| XAXW | 4 | 4 | 5 | 2 | 1 | 2 | 2 | 0 | 1 | 2 | 2 | 1 | 8 | 3 | 2 | 2 | 1 | 9 | 4 | 1 | 2 | 3 | 0 | 3 | 1 | 6 | 4 | 0 | 0 | 0 | 0 | 2 | 2 | 1 | 2 | 0 | 1 | 1 | 1 | 8 | 1 | 2 | 2 | 1 | 8 | 0 | 2 | 0 | 2 | 2 | 19 | 1 | 0 | 0 | 0 | 2 | 1 | 1 | 2 | 2 | 2 | 1 | 1 | 1 | 1 | 0 | 0 | 0 | 6 | 0 | 8 | 4 | 0 | 2 | 1 | 1 | 1 | 1 | 0 | 1 | 0 | 1 | 0 | 1 |
| JEBK | 2 | 2 | 5 | 2 | 1 | 0 | 3 | 0 | 4 | 0 | 1 | 1 | 11 | 6 | 2 | 2 | 1 | 8 | 4 | 4 | 2 | 2 | 0 | 3 | 1 | 7 | 5 | 0 | 0 | 0 | 0 | 2 | 4 | 1 | 3 | 0 | 1 | 2 | 3 | 9 | 1 | 1 | 2 | 3 | 8 | 0 | 2 | 0 | 2 | 1 | 26 | 2 | 0 | 0 | 0 | 2 | 1 | 1 | 3 | 4 | 2 | 2 | 0 | 1 | 1 | 0 | 0 | 0 | 8 | 0 | 8 | 3 | 0 | 2 | 2 | 2 | 1 | 1 | 1 | 1 | 1 | 1 | 1 | 1 |
| IHJY | 2 | 1 | 4 | 2 | 1 | 2 | 0 | 0 | 1 | 1 | 1 | 1 | 7 | 6 | 2 | 2 | 1 | 6 | 2 | 4 | 2 | 2 | 0 | 2 | 2 | 5 | 4 | 0 | 0 | 0 | 0 | 2 | 2 | 1 | 1 | 0 | 1 | 2 | 2 | 9 | 1 | 1 | 2 | 3 | 6 | 0 | 1 | 0 | 2 | 2 | 20 | 1 | 0 | 0 | 0 | 2 | 0 | 1 | 3 | 3 | 2 | 2 | 0 | 1 | 1 | 0 | 0 | 0 | 7 | 0 | 4 | 3 | 0 | 2 | 2 | 2 | 1 | 1 | 1 | 1 | 1 | 1 | 0 | 1 |
| CKXF | 1 | 1 | 3 | 2 | 1 | 1 | 1 | 0 | 2 | 2 | 1 | 1 | 13 | 4 | 2 | 2 | 2 | 8 | 2 | 5 | 2 | 2 | 0 | 2 | 1 | 7 | 4 | 0 | 4 | 0 | 0 | 2 | 4 | 1 | 3 | 0 | 2 | 2 | 3 | 10 | 1 | 1 | 2 | 4 | 5 | 0 | 2 | 0 | 2 | 1 | 29 | 1 | 0 | 1 | 0 | 2 | 1 | 1 | 2 | 3 | 2 | 2 | 1 | 1 | 2 | 0 | 1 | 0 | 9 | 0 | 9 | 3 | 0 | 2 | 1 | 1 | 1 | 1 | 1 | 1 | 1 | 1 | 1 | 1 |
| SBLT | 1 | 2 | 3 | 2 | 1 | 0 | 2 | 0 | 8 | 0 | 1 | 1 | 14 | 4 | 2 | 2 | 2 | 3 | 3 | 4 | 2 | 2 | 0 | 3 | 0 | 5 | 4 | 0 | 0 | 0 | 0 | 2 | 4 | 1 | 3 | 0 | 2 | 2 | 3 | 9 | 1 | 1 | 2 | 4 | 4 | 0 | 2 | 0 | 2 | 1 | 27 | 1 | 0 | 0 | 0 | 2 | 1 | 1 | 3 | 4 | 2 | 1 | 1 | 0 | 1 | 0 | 1 | 0 | 8 | 0 | 9 | 2 | 0 | 2 | 1 | 1 | 1 | 1 | 1 | 1 | 1 | 1 | 1 | 1 |
| UGPM | 3 | 2 | 5 | 4 | 1 | 1 | 2 | 0 | 1 | 1 | 1 | 1 | 11 | 4 | 2 | 2 | 1 | 14 | 4 | 4 | 2 | 2 | 0 | 4 | 0 | 7 | 4 | 0 | 0 | 0 | 0 | 4 | 3 | 1 | 3 | 0 | 1 | 2 | 3 | 9 | 1 | 2 | 2 | 3 | 5 | 0 | 2 | 0 | 2 | 4 | 23 | 3 | 0 | 0 | 0 | 2 | 1 | 1 | 4 | 4 | 2 | 1 | 1 | 1 | 1 | 0 | 0 | 0 | 6 | 0 | 9 | 5 | 0 | 2 | 1 | 1 | 1 | 1 | 1 | 1 | 0 | 1 | 1 | 1 |
| PYDB | 2 | 1 | 6 | 3 | 1 | 1 | 1 | 0 | 1 | 0 | 1 | 1 | 12 | 6 | 1 | 1 | 1 | 19 | 6 | 4 | 2 | 2 | 0 | 2 | 1 | 8 | 5 | 0 | 4 | 0 | 0 | 2 | 4 | 1 | 3 | 0 | 3 | 1 | 3 | 9 | 1 | 1 | 2 | 3 | 7 | 0 | 2 | 0 | 2 | 3 | 26 | 1 | 0 | 0 | 0 | 2 | 1 | 1 | 2 | 3 | 2 | 2 | 0 | 1 | 1 | 0 | 0 | 0 | 9 | 0 | 9 | 3 | 0 | 2 | 1 | 1 | 1 | 1 | 1 | 1 | 1 | 1 | 1 | 1 |
| URSB | 1 | 1 | 4 | 2 | 1 | 0 | 2 | 0 | 1 | 0 | 1 | 1 | 11 | 4 | 2 | 2 | 1 | 4 | 3 | 5 | 2 | 3 | 0 | 2 | 1 | 6 | 4 | 0 | 0 | 0 | 0 | 2 | 3 | 1 | 3 | 0 | 3 | 2 | 3 | 8 | 1 | 1 | 2 | 2 | 6 | 0 | 2 | 0 | 2 | 1 | 26 | 1 | 0 | 1 | 0 | 2 | 1 | 1 | 2 | 3 | 2 | 2 | 0 | 1 | 1 | 0 | 0 | 0 | 9 | 0 | 10 | 3 | 0 | 2 | 1 | 1 | 1 | 1 | 1 | 1 | 1 | 1 | 1 | 1 |
| IKIZ | 1 | 1 | 4 | 2 | 1 | 1 | 1 | 0 | 4 | 1 | 1 | 1 | 12 | 4 | 2 | 2 | 1 | 3 | 2 | 4 | 2 | 2 | 0 | 2 | 2 | 7 | 4 | 0 | 0 | 0 | 0 | 2 | 3 | 2 | 3 | 0 | 3 | 2 | 3 | 9 | 1 | 1 | 2 | 2 | 6 | 0 | 2 | 0 | 2 | 1 | 27 | 1 | 0 | 0 | 0 | 2 | 1 | 1 | 2 | 3 | 2 | 2 | 1 | 1 | 1 | 0 | 0 | 0 | 7 | 0 | 9 | 4 | 0 | 2 | 1 | 1 | 1 | 1 | 1 | 1 | 1 | 1 | 1 | 1 |
| ZJOJ | 4 | 2 | 12 | 6 | 3 | 1 | 2 | 1 | 5 | 1 | 5 | 1 | 14 | 5 | 2 | 2 | 1 | 19 | 14 | 4 | 3 | 5 | 0 | 9 | 2 | 14 | 8 | 0 | 0 | 4 | 0 | 11 | 3 | 1 | 3 | 0 | 3 | 2 | 3 | 10 | 1 | 1 | 2 | 4 | 6 | 0 | 2 | 0 | 3 | 12 | 31 | 3 | 0 | 0 | 0 | 6 | 1 | 3 | 2 | 3 | 3 | 4 | 3 | 1 | 2 | 0 | 0 | 0 | 8 | 0 | 10 | 5 | 0 | 2 | 1 | 1 | 2 | 2 | 1 | 1 | 1 | 1 | 1 | 3 |
| FTRP | 1 | 1 | 4 | 2 | 1 | 1 | 1 | 0 | 4 | 0 | 1 | 1 | 13 | 5 | 2 | 2 | 1 | 2 | 2 | 5 | 2 | 2 | 0 | 2 | 1 | 7 | 4 | 0 | 0 | 0 | 0 | 2 | 3 | 2 | 3 | 0 | 3 | 2 | 3 | 8 | 1 | 1 | 2 | 5 | 5 | 0 | 2 | 0 | 2 | 1 | 27 | 1 | 0 | 0 | 0 | 1 | 1 | 1 | 2 | 3 | 2 | 1 | 1 | 1 | 1 | 0 | 0 | 0 | 8 | 0 | 9 | 3 | 0 | 2 | 1 | 1 | 1 | 1 | 1 | 1 | 1 | 1 | 1 | 1 |
| QFND | 1 | 3 | 3 | 2 | 2 | 1 | 5 | 0 | 3 | 1 | 0 | 3 | 10 | 2 | 1 | 1 | 1 | 2 | 2 | 3 | 2 | 3 | 0 | 3 | 3 | 6 | 2 | 1 | 1 | 0 | 0 | 2 | 3 | 2 | 1 | 0 | 2 | 1 | 1 | 7 | 1 | 0 | 2 | 2 | 2 | 0 | 1 | 0 | 0 | 1 | 16 | 3 | 0 | 1 | 0 | 2 | 2 | 1 | 2 | 5 | 2 | 3 | 0 | 1 | 1 | 0 | 1 | 0 | 5 | 0 | 5 | 2 | 0 | 2 | 0 | 0 | 1 | 2 | 1 | 1 | 0 | 0 | 1 | 1 |
| POOW | 3 | 2 | 4 | 1 | 2 | 0 | 2 | 0 | 17 | 0 | 0 | 4 | 17 | 3 | 2 | 2 | 1 | 6 | 5 | 11 | 4 | 3 | 3 | 3 | 1 | 5 | 4 | 1 | 1 | 1 | 2 | 2 | 4 | 3 | 3 | 0 | 2 | 2 | 4 | 10 | 3 | 1 | 2 | 3 | 9 | 0 | 0 | 0 | 3 | 2 | 31 | 2 | 0 | 2 | 0 | 3 | 2 | 1 | 4 | 5 | 3 | 1 | 0 | 1 | 3 | 0 | 2 | 1 | 6 | 0 | 12 | 3 | 0 | 3 | 2 | 2 | 1 | 1 | 1 | 1 | 1 | 0 | 0 | 1 |
| PQED | 3 | 4 | 4 | 1 | 4 | 1 | 5 | 0 | 15 | 1 | 0 | 4 | 15 | 4 | 2 | 2 | 1 | 5 | 4 | 7 | 3 | 4 | 2 | 3 | 0 | 8 | 2 | 1 | 6 | 0 | 1 | 3 | 5 | 3 | 3 | 0 | 4 | 1 | 5 | 10 | 1 | 1 | 2 | 4 | 4 | 0 | 1 | 0 | 5 | 4 | 30 | 4 | 0 | 1 | 0 | 1 | 3 | 0 | 3 | 6 | 3 | 3 | 0 | 1 | 2 | 0 | 1 | 1 | 7 | 0 | 10 | 3 | 0 | 2 | 1 | 1 | 1 | 1 | 2 | 1 | 2 | 0 | 1 | 1 |
| CBNG | 1 | 1 | 1 | 1 | 1 | 1 | 0 | 0 | 0 | 1 | 0 | 2 | 11 | 5 | 0 | 0 | 0 | 5 | 2 | 2 | 2 | 2 | 0 | 1 | 0 | 1 | 1 | 1 | 0 | 1 | 0 | 1 | 0 | 2 | 1 | 0 | 0 | 2 | 0 | 4 | 0 | 0 | 2 | 1 | 3 | 0 | 2 | 0 | 1 | 2 | 17 | 1 | 2 | 0 | 0 | 1 | 1 | 1 | 0 | 1 | 2 | 1 | 0 | 1 | 0 | 0 | 0 | 1 | 3 | 0 | 3 | 1 | 0 | 0 | 1 | 1 | 2 | 1 | 0 | 1 | 0 | 0 | 0 | 1 |
| LETF | 1 | 2 | 2 | 1 | 1 | 1 | 1 | 3 | 2 | 1 | 0 | 2 | 19 | 6 | 3 | 2 | 1 | 3 | 3 | 5 | 2 | 2 | 0 | 2 | 1 | 3 | 2 | 1 | 0 | 1 | 0 | 1 | 4 | 6 | 5 | 2 | 2 | 2 | 2 | 7 | 2 | 1 | 2 | 3 | 4 | 0 | 2 | 0 | 4 | 2 | 29 | 1 | 2 | 2 | 0 | 2 | 1 | 1 | 6 | 7 | 2 | 3 | 2 | 2 | 1 | 1 | 1 | 1 | 9 | 0 | 6 | 6 | 0 | 3 | 1 | 1 | 1 | 1 | 1 | 1 | 1 | 0 | 0 | 1 |
| AJAU | 1 | 2 | 3 | 1 | 2 | 0 | 2 | 2 | 0 | 1 | 0 | 2 | 21 | 6 | 5 | 5 | 1 | 2 | 2 | 6 | 2 | 2 | 0 | 1 | 1 | 4 | 2 | 1 | 0 | 1 | 0 | 1 | 3 | 6 | 5 | 2 | 2 | 3 | 4 | 8 | 1 | 1 | 2 | 3 | 5 | 0 | 2 | 2 | 4 | 2 | 37 | 1 | 2 | 2 | 0 | 2 | 1 | 1 | 5 | 8 | 2 | 3 | 1 | 2 | 1 | 1 | 0 | 1 | 8 | 0 | 7 | 6 | 0 | 4 | 1 | 1 | 1 | 1 | 1 | 1 | 1 | 0 | 0 | 1 |
| JIWJ | 1 | 1 | 3 | 3 | 2 | 0 | 1 | 1 | 4 | 1 | 0 | 2 | 17 | 10 | 4 | 4 | 1 | 9 | 5 | 6 | 1 | 3 | 0 | 5 | 4 | 5 | 2 | 2 | 0 | 3 | 0 | 4 | 4 | 4 | 5 | 2 | 1 | 4 | 3 | 8 | 1 | 1 | 2 | 3 | 5 | 0 | 3 | 0 | 2 | 4 | 29 | 2 | 1 | 2 | 1 | 2 | 2 | 2 | 5 | 9 | 1 | 3 | 1 | 2 | 1 | 1 | 0 | 1 | 9 | 0 | 10 | 6 | 0 | 5 | 1 | 1 | 1 | 1 | 1 | 1 | 1 | 0 | 0 | 1 |
| CQQP | 2 | 6 | 4 | 2 | 2 | 2 | 4 | 2 | 0 | 2 | 0 | 3 | 28 | 11 | 4 | 3 | 2 | 10 | 6 | 5 | 4 | 6 | 0 | 4 | 4 | 7 | 5 | 2 | 0 | 3 | 0 | 3 | 5 | 6 | 6 | 3 | 2 | 2 | 0 | 9 | 2 | 2 | 4 | 4 | 7 | 0 | 3 | 1 | 3 | 5 | 51 | 2 | 2 | 0 | 0 | 5 | 3 | 2 | 7 | 16 | 4 | 5 | 1 | 3 | 2 | 2 | 0 | 1 | 15 | 0 | 11 | 8 | 0 | 8 | 1 | 2 | 4 | 2 | 2 | 2 | 2 | 0 | 0 | 1 |
| OAEZ | 1 | 4 | 2 | 1 | 1 | 1 | 3 | 1 | 1 | 1 | 0 | 2 | 17 | 5 | 4 | 4 | 1 | 5 | 3 | 4 | 1 | 2 | 0 | 3 | 2 | 3 | 3 | 1 | 0 | 2 | 0 | 1 | 3 | 5 | 5 | 2 | 1 | 2 | 3 | 5 | 0 | 1 | 2 | 2 | 3 | 0 | 3 | 0 | 3 | 2 | 31 | 1 | 2 | 2 | 0 | 2 | 1 | 1 | 4 | 7 | 1 | 3 | 2 | 2 | 1 | 1 | 0 | 1 | 8 | 0 | 9 | 5 | 0 | 5 | 1 | 1 | 1 | 1 | 1 | 1 | 1 | 0 | 0 | 1 |
| NQYP | 1 | 1 | 2 | 1 | 1 | 0 | 2 | 2 | 1 | 0 | 0 | 1 | 16 | 6 | 2 | 2 | 1 | 3 | 3 | 4 | 2 | 2 | 0 | 3 | 0 | 2 | 1 | 2 | 0 | 1 | 0 | 1 | 1 | 2 | 3 | 2 | 2 | 1 | 3 | 7 | 2 | 1 | 2 | 2 | 5 | 0 | 1 | 0 | 2 | 2 | 30 | 1 | 1 | 0 | 0 | 1 | 2 | 1 | 5 | 3 | 2 | 2 | 1 | 2 | 1 | 1 | 0 | 1 | 7 | 0 | 6 | 4 | 0 | 2 | 0 | 0 | 1 | 1 | 1 | 1 | 1 | 0 | 0 | 1 |
| GJIY | 1 | 2 | 2 | 2 | 2 | 1 | 2 | 3 | 0 | 1 | 0 | 2 | 16 | 6 | 3 | 3 | 2 | 2 | 2 | 4 | 2 | 2 | 0 | 1 | 2 | 4 | 3 | 1 | 0 | 1 | 0 | 1 | 3 | 7 | 7 | 4 | 1 | 1 | 5 | 10 | 1 | 1 | 2 | 2 | 4 | 0 | 2 | 0 | 4 | 2 | 26 | 1 | 2 | 2 | 0 | 3 | 2 | 1 | 4 | 7 | 2 | 3 | 1 | 2 | 1 | 0 | 1 | 1 | 8 | 0 | 8 | 7 | 0 | 4 | 1 | 1 | 1 | 1 | 1 | 1 | 1 | 0 | 0 | 1 |
| KADG | 1 | 2 | 2 | 1 | 2 | 1 | 2 | 2 | 3 | 1 | 0 | 2 | 18 | 6 | 5 | 5 | 2 | 2 | 2 | 3 | 2 | 3 | 0 | 1 | 1 | 6 | 5 | 1 | 0 | 2 | 0 | 2 | 5 | 6 | 6 | 2 | 1 | 1 | 2 | 9 | 1 | 1 | 2 | 4 | 4 | 0 | 2 | 0 | 1 | 2 | 32 | 1 | 3 | 0 | 1 | 2 | 3 | 2 | 5 | 8 | 2 | 3 | 1 | 1 | 1 | 1 | 0 | 1 | 7 | 0 | 12 | 8 | 0 | 3 | 0 | 0 | 1 | 0 | 0 | 1 | 0 | 0 | 1 | 1 |
| VBLH | 1 | 2 | 3 | 2 | 1 | 1 | 2 | 0 | 1 | 1 | 0 | 1 | 2 | 5 | 4 | 2 | 1 | 3 | 3 | 3 | 2 | 2 | 0 | 1 | 4 | 3 | 2 | 1 | 0 | 0 | 0 | 1 | 1 | 0 | 3 | 4 | 0 | 3 | 0 | 2 | 1 | 1 | 1 | 1 | 1 | 0 | 1 | 0 | 2 | 2 | 8 | 1 | 1 | 2 | 0 | 1 | 2 | 0 | 3 | 2 | 3 | 1 | 1 | 2 | 1 | 0 | 0 | 1 | 4 | 0 | 5 | 5 | 0 | 3 | 1 | 1 | 1 | 1 | 1 | 1 | 1 | 0 | 0 | 1 |
| YDCQ | 1 | 1 | 0 | 2 | 2 | 1 | 1 | 0 | 1 | 1 | 0 | 1 | 2 | 4 | 2 | 0 | 0 | 2 | 2 | 5 | 1 | 2 | 0 | 1 | 1 | 2 | 1 | 1 | 0 | 0 | 0 | 1 | 1 | 0 | 4 | 4 | 0 | 1 | 0 | 1 | 0 | 1 | 2 | 1 | 0 | 0 | 1 | 0 | 2 | 0 | 10 | 1 | 1 | 0 | 0 | 0 | 1 | 1 | 3 | 0 | 1 | 2 | 1 | 1 | 1 | 0 | 0 | 1 | 3 | 0 | 3 | 3 | 0 | 2 | 0 | 0 | 2 | 1 | 0 | 1 | 0 | 0 | 0 | 1 |
| ZDIZ | 1 | 2 | 3 | 3 | 2 | 0 | 3 | 3 | 0 | 0 | 0 | 1 | 14 | 5 | 3 | 3 | 2 | 7 | 4 | 4 | 3 | 2 | 1 | 2 | 2 | 4 | 3 | 1 | 0 | 1 | 1 | 1 | 4 | 3 | 4 | 3 | 1 | 4 | 2 | 3 | 2 | 3 | 2 | 4 | 3 | 0 | 1 | 1 | 4 | 2 | 27 | 1 | 2 | 1 | 0 | 3 | 1 | 1 | 4 | 5 | 2 | 3 | 1 | 1 | 1 | 1 | 1 | 1 | 6 | 0 | 11 | 6 | 0 | 3 | 1 | 1 | 2 | 2 | 2 | 1 | 2 | 0 | 1 | 1 |
| WDWX | 2 | 2 | 4 | 3 | 2 | 1 | 1 | 3 | 1 | 1 | 0 | 1 | 14 | 5 | 3 | 3 | 2 | 7 | 5 | 4 | 2 | 2 | 1 | 1 | 2 | 4 | 3 | 1 | 0 | 1 | 0 | 1 | 5 | 3 | 5 | 3 | 1 | 4 | 2 | 4 | 0 | 2 | 2 | 4 | 3 | 0 | 1 | 1 | 4 | 2 | 26 | 1 | 2 | 1 | 0 | 2 | 1 | 2 | 4 | 7 | 2 | 3 | 1 | 1 | 1 | 1 | 1 | 1 | 6 | 0 | 12 | 6 | 0 | 4 | 2 | 2 | 2 | 2 | 2 | 1 | 2 | 0 | 1 | 1 |
| RHVC | 2 | 0 | 3 | 3 | 2 | 0 | 0 | 3 | 0 | 0 | 0 | 1 | 13 | 5 | 3 | 3 | 1 | 4 | 2 | 3 | 2 | 3 | 0 | 2 | 2 | 6 | 4 | 1 | 0 | 1 | 0 | 1 | 4 | 1 | 2 | 3 | 0 | 5 | 2 | 4 | 0 | 2 | 2 | 3 | 4 | 0 | 0 | 0 | 3 | 3 | 25 | 1 | 2 | 1 | 0 | 2 | 1 | 2 | 4 | 6 | 2 | 3 | 1 | 1 | 1 | 1 | 1 | 1 | 6 | 0 | 8 | 4 | 0 | 3 | 2 | 2 | 2 | 2 | 2 | 1 | 2 | 0 | 1 | 1 |
| UKUC | 2 | 2 | 5 | 2 | 2 | 0 | 3 | 3 | 1 | 1 | 0 | 2 | 15 | 5 | 2 | 1 | 1 | 5 | 3 | 4 | 2 | 3 | 1 | 3 | 2 | 4 | 3 | 1 | 0 | 2 | 1 | 1 | 4 | 2 | 2 | 3 | 0 | 2 | 0 | 3 | 1 | 2 | 2 | 2 | 3 | 0 | 1 | 0 | 2 | 4 | 25 | 1 | 2 | 0 | 0 | 2 | 1 | 0 | 3 | 2 | 1 | 2 | 1 | 2 | 1 | 1 | 1 | 1 | 5 | 0 | 8 | 5 | 0 | 2 | 0 | 0 | 2 | 2 | 1 | 1 | 1 | 0 | 0 | 1 |
| SYJM | 1 | 3 | 4 | 2 | 2 | 1 | 1 | 3 | 0 | 1 | 0 | 1 | 18 | 5 | 3 | 3 | 2 | 3 | 3 | 4 | 2 | 3 | 1 | 1 | 2 | 5 | 4 | 1 | 0 | 1 | 0 | 1 | 3 | 3 | 4 | 2 | 0 | 3 | 2 | 5 | 1 | 1 | 2 | 3 | 4 | 0 | 1 | 0 | 2 | 2 | 29 | 1 | 2 | 1 | 0 | 2 | 1 | 1 | 5 | 6 | 2 | 3 | 1 | 1 | 2 | 1 | 2 | 1 | 6 | 0 | 11 | 6 | 0 | 2 | 1 | 1 | 2 | 2 | 2 | 1 | 2 | 0 | 1 | 1 |
| QRTH | 2 | 3 | 4 | 3 | 3 | 2 | 5 | 1 | 1 | 2 | 0 | 2 | 10 | 6 | 3 | 3 | 1 | 7 | 3 | 2 | 2 | 3 | 1 | 2 | 2 | 3 | 3 | 0 | 0 | 1 | 1 | 2 | 3 | 3 | 3 | 2 | 1 | 2 | 1 | 4 | 0 | 1 | 2 | 1 | 5 | 0 | 1 | 0 | 1 | 2 | 24 | 1 | 2 | 1 | 0 | 2 | 1 | 1 | 5 | 7 | 2 | 2 | 1 | 1 | 1 | 0 | 0 | 1 | 6 | 0 | 10 | 4 | 0 | 3 | 1 | 1 | 1 | 1 | 1 | 1 | 1 | 0 | 0 | 1 |
| PRIQ | 1 | 5 | 3 | 3 | 1 | 2 | 2 | 2 | 2 | 2 | 0 | 2 | 17 | 5 | 3 | 3 | 1 | 4 | 3 | 5 | 2 | 3 | 1 | 2 | 2 | 5 | 5 | 0 | 0 | 2 | 1 | 1 | 3 | 3 | 4 | 3 | 2 | 2 | 0 | 6 | 1 | 0 | 2 | 3 | 1 | 0 | 2 | 0 | 4 | 2 | 31 | 1 | 2 | 1 | 0 | 2 | 1 | 1 | 5 | 5 | 2 | 3 | 1 | 1 | 2 | 1 | 1 | 1 | 5 | 0 | 9 | 5 | 0 | 4 | 1 | 1 | 1 | 1 | 2 | 1 | 1 | 0 | 1 | 1 |
| ZLQE | 1 | 1 | 3 | 3 | 1 | 1 | 0 | 3 | 0 | 1 | 0 | 2 | 13 | 5 | 4 | 3 | 1 | 2 | 2 | 4 | 2 | 3 | 1 | 1 | 1 | 4 | 4 | 0 | 0 | 1 | 1 | 1 | 2 | 3 | 3 | 3 | 2 | 3 | 0 | 5 | 1 | 1 | 2 | 4 | 2 | 0 | 1 | 0 | 2 | 2 | 24 | 1 | 2 | 1 | 0 | 2 | 2 | 1 | 5 | 7 | 2 | 2 | 1 | 2 | 1 | 1 | 1 | 1 | 6 | 0 | 7 | 4 | 1 | 3 | 1 | 1 | 1 | 1 | 1 | 1 | 1 | 0 | 1 | 1 |
| GUBD | 1 | 3 | 3 | 3 | 2 | 2 | 1 | 1 | 0 | 1 | 0 | 2 | 16 | 5 | 3 | 3 | 1 | 3 | 3 | 5 | 3 | 3 | 1 | 2 | 1 | 4 | 4 | 0 | 0 | 1 | 1 | 1 | 5 | 1 | 1 | 3 | 4 | 4 | 2 | 6 | 1 | 1 | 2 | 4 | 8 | 0 | 1 | 0 | 4 | 2 | 29 | 2 | 2 | 1 | 0 | 2 | 1 | 1 | 5 | 6 | 2 | 2 | 1 | 2 | 1 | 1 | 1 | 1 | 6 | 0 | 9 | 4 | 1 | 4 | 1 | 1 | 2 | 2 | 1 | 1 | 1 | 0 | 1 | 1 |
| MULF | 1 | 3 | 4 | 3 | 1 | 2 | 3 | 1 | 12 | 2 | 0 | 3 | 9 | 6 | 1 | 1 | 1 | 5 | 1 | 2 | 2 | 2 | 1 | 1 | 1 | 1 | 2 | 1 | 0 | 2 | 1 | 3 | 3 | 1 | 3 | 2 | 0 | 2 | 2 | 3 | 0 | 1 | 2 | 2 | 4 | 0 | 2 | 0 | 1 | 4 | 19 | 1 | 2 | 1 | 0 | 3 | 1 | 1 | 5 | 6 | 2 | 2 | 1 | 1 | 1 | 1 | 0 | 1 | 4 | 1 | 6 | 3 | 0 | 4 | 0 | 0 | 1 | 1 | 1 | 1 | 1 | 0 | 0 | 1 |
| VALZ | 2 | 1 | 4 | 1 | 1 | 1 | 0 | 2 | 2 | 1 | 0 | 2 | 11 | 9 | 3 | 3 | 1 | 4 | 2 | 3 | 5 | 2 | 1 | 1 | 1 | 4 | 2 | 2 | 0 | 3 | 1 | 4 | 5 | 3 | 5 | 2 | 1 | 3 | 0 | 5 | 0 | 1 | 2 | 1 | 6 | 0 | 2 | 0 | 1 | 4 | 17 | 5 | 2 | 1 | 0 | 1 | 1 | 2 | 6 | 9 | 4 | 2 | 2 | 2 | 2 | 1 | 0 | 1 | 6 | 0 | 9 | 6 | 0 | 3 | 1 | 2 | 1 | 1 | 0 | 1 | 0 | 0 | 0 | 1 |
| JRGZ | 1 | 1 | 4 | 3 | 2 | 0 | 1 | 1 | 9 | 1 | 0 | 1 | 9 | 4 | 2 | 2 | 0 | 3 | 2 | 4 | 2 | 2 | 0 | 1 | 0 | 3 | 1 | 1 | 0 | 2 | 1 | 3 | 3 | 4 | 4 | 3 | 2 | 1 | 2 | 4 | 0 | 0 | 2 | 0 | 2 | 0 | 2 | 0 | 2 | 2 | 15 | 2 | 2 | 0 | 0 | 1 | 0 | 2 | 5 | 7 | 2 | 2 | 1 | 1 | 1 | 1 | 0 | 1 | 6 | 0 | 5 | 5 | 0 | 1 | 0 | 0 | 0 | 0 | 0 | 1 | 0 | 0 | 0 | 1 |
| GFUR | 2 | 2 | 4 | 2 | 2 | 1 | 1 | 1 | 0 | 1 | 0 | 2 | 10 | 5 | 2 | 1 | 1 | 4 | 2 | 4 | 2 | 2 | 1 | 1 | 1 | 6 | 3 | 1 | 0 | 2 | 1 | 3 | 6 | 3 | 3 | 2 | 3 | 2 | 3 | 2 | 0 | 1 | 2 | 2 | 2 | 0 | 1 | 0 | 3 | 2 | 20 | 2 | 2 | 0 | 0 | 0 | 2 | 1 | 5 | 7 | 3 | 2 | 1 | 1 | 1 | 0 | 0 | 1 | 4 | 0 | 7 | 5 | 0 | 3 | 1 | 1 | 2 | 1 | 0 | 1 | 0 | 0 | 1 | 1 |
| TSBQ | 2 | 2 | 3 | 2 | 3 | 2 | 1 | 1 | 2 | 1 | 0 | 0 | 8 | 2 | 1 | 1 | 1 | 4 | 1 | 1 | 2 | 2 | 0 | 1 | 1 | 4 | 2 | 1 | 0 | 0 | 1 | 2 | 4 | 2 | 3 | 2 | 1 | 2 | 0 | 5 | 0 | 0 | 2 | 2 | 2 | 0 | 2 | 0 | 1 | 2 | 15 | 2 | 2 | 1 | 0 | 1 | 2 | 1 | 2 | 2 | 2 | 2 | 1 | 1 | 1 | 1 | 0 | 0 | 3 | 0 | 5 | 6 | 1 | 3 | 0 | 0 | 2 | 2 | 2 | 0 | 2 | 0 | 0 | 1 |
| ACRY | 1 | 0 | 2 | 2 | 1 | 0 | 1 | 1 | 0 | 0 | 0 | 2 | 13 | 5 | 2 | 2 | 1 | 4 | 2 | 5 | 3 | 3 | 1 | 1 | 2 | 6 | 4 | 0 | 0 | 1 | 1 | 2 | 1 | 3 | 3 | 3 | 1 | 0 | 2 | 4 | 0 | 1 | 2 | 0 | 1 | 0 | 1 | 0 | 2 | 2 | 18 | 2 | 1 | 1 | 0 | 2 | 0 | 2 | 4 | 5 | 3 | 2 | 1 | 0 | 1 | 1 | 1 | 2 | 5 | 0 | 6 | 4 | 1 | 3 | 0 | 0 | 2 | 2 | 1 | 1 | 1 | 0 | 0 | 1 |
| LNIL | 1 | 2 | 3 | 1 | 2 | 2 | 1 | 1 | 5 | 1 | 0 | 2 | 11 | 6 | 4 | 1 | 2 | 3 | 3 | 4 | 2 | 2 | 1 | 1 | 2 | 4 | 3 | 0 | 0 | 1 | 1 | 3 | 4 | 2 | 5 | 3 | 0 | 0 | 2 | 3 | 0 | 0 | 2 | 3 | 1 | 0 | 0 | 0 | 3 | 2 | 20 | 2 | 2 | 1 | 0 | 2 | 0 | 2 | 4 | 6 | 2 | 3 | 1 | 0 | 1 | 1 | 0 | 2 | 6 | 0 | 9 | 5 | 1 | 3 | 0 | 0 | 2 | 2 | 1 | 1 | 1 | 0 | 1 | 1 |
| ZIVZ | 1 | 2 | 3 | 1 | 1 | 1 | 4 | 4 | 2 | 1 | 0 | 2 | 15 | 5 | 2 | 2 | 0 | 4 | 3 | 5 | 3 | 3 | 1 | 2 | 2 | 5 | 4 | 0 | 0 | 1 | 1 | 3 | 3 | 4 | 2 | 3 | 2 | 1 | 2 | 2 | 1 | 1 | 2 | 3 | 4 | 0 | 3 | 0 | 3 | 2 | 24 | 2 | 1 | 1 | 0 | 1 | 0 | 2 | 5 | 7 | 2 | 2 | 1 | 0 | 1 | 1 | 2 | 1 | 4 | 0 | 8 | 4 | 0 | 3 | 0 | 0 | 2 | 2 | 2 | 1 | 1 | 0 | 0 | 1 |
| WRSL | 1 | 2 | 4 | 2 | 2 | 1 | 2 | 1 | 1 | 1 | 0 | 2 | 18 | 5 | 3 | 3 | 1 | 3 | 3 | 6 | 2 | 2 | 1 | 2 | 2 | 4 | 3 | 1 | 0 | 2 | 1 | 1 | 3 | 4 | 4 | 2 | 3 | 1 | 2 | 8 | 1 | 1 | 2 | 6 | 3 | 0 | 2 | 0 | 2 | 2 | 25 | 2 | 2 | 1 | 0 | 2 | 1 | 2 | 5 | 7 | 1 | 2 | 1 | 1 | 1 | 1 | 0 | 1 | 6 | 0 | 6 | 5 | 0 | 2 | 1 | 1 | 1 | 1 | 1 | 1 | 1 | 0 | 1 | 1 |
| JWGT | 1 | 2 | 4 | 2 | 2 | 1 | 2 | 1 | 2 | 1 | 0 | 2 | 15 | 5 | 3 | 3 | 1 | 3 | 3 | 5 | 1 | 2 | 1 | 2 | 1 | 4 | 3 | 1 | 0 | 2 | 1 | 1 | 3 | 4 | 4 | 2 | 2 | 2 | 2 | 6 | 1 | 1 | 2 | 4 | 5 | 0 | 2 | 0 | 2 | 2 | 23 | 1 | 2 | 0 | 0 | 2 | 2 | 3 | 4 | 6 | 1 | 2 | 1 | 1 | 1 | 1 | 0 | 1 | 6 | 0 | 6 | 5 | 0 | 2 | 1 | 1 | 1 | 1 | 1 | 1 | 1 | 0 | 1 | 1 |
| RYJX | 1 | 1 | 3 | 2 | 2 | 0 | 2 | 2 | 2 | 1 | 0 | 2 | 16 | 5 | 3 | 3 | 1 | 4 | 3 | 6 | 2 | 2 | 1 | 1 | 1 | 3 | 3 | 1 | 0 | 2 | 1 | 1 | 2 | 3 | 4 | 2 | 3 | 2 | 3 | 6 | 0 | 1 | 2 | 3 | 5 | 0 | 2 | 0 | 1 | 2 | 25 | 2 | 2 | 1 | 0 | 1 | 2 | 2 | 5 | 7 | 2 | 3 | 1 | 2 | 1 | 1 | 1 | 1 | 6 | 0 | 6 | 4 | 0 | 2 | 0 | 0 | 1 | 1 | 1 | 1 | 1 | 0 | 0 | 1 |
| RNAT | 1 | 1 | 4 | 3 | 2 | 0 | 1 | 1 | 3 | 0 | 0 | 2 | 16 | 5 | 3 | 3 | 1 | 5 | 5 | 5 | 2 | 2 | 1 | 1 | 2 | 3 | 3 | 1 | 0 | 2 | 1 | 1 | 3 | 4 | 4 | 2 | 2 | 3 | 2 | 6 | 0 | 1 | 2 | 3 | 4 | 0 | 2 | 0 | 1 | 2 | 24 | 2 | 2 | 1 | 0 | 2 | 2 | 3 | 5 | 8 | 2 | 2 | 1 | 1 | 1 | 1 | 3 | 1 | 6 | 0 | 7 | 5 | 0 | 2 | 0 | 0 | 1 | 1 | 1 | 1 | 1 | 0 | 0 | 1 |
| ISPU | 1 | 2 | 3 | 1 | 2 | 1 | 2 | 1 | 3 | 1 | 0 | 2 | 16 | 5 | 3 | 2 | 1 | 3 | 3 | 6 | 4 | 3 | 1 | 1 | 1 | 4 | 4 | 0 | 0 | 1 | 1 | 1 | 4 | 3 | 3 | 2 | 3 | 2 | 2 | 7 | 2 | 1 | 2 | 2 | 3 | 0 | 1 | 0 | 1 | 2 | 25 | 1 | 2 | 1 | 0 | 2 | 1 | 1 | 5 | 7 | 3 | 3 | 1 | 2 | 1 | 1 | 2 | 1 | 6 | 0 | 9 | 4 | 0 | 2 | 0 | 0 | 1 | 1 | 1 | 1 | 1 | 0 | 2 | 1 |
| IHOI | 1 | 3 | 4 | 1 | 1 | 1 | 2 | 2 | 5 | 1 | 0 | 1 | 4 | 3 | 2 | 1 | 0 | 3 | 1 | 1 | 1 | 1 | 1 | 1 | 1 | 3 | 2 | 2 | 0 | 1 | 1 | 2 | 3 | 1 | 2 | 2 | 1 | 0 | 1 | 3 | 0 | 0 | 1 | 0 | 0 | 0 | 1 | 0 | 1 | 2 | 9 | 1 | 2 | 1 | 0 | 2 | 0 | 1 | 2 | 1 | 1 | 2 | 1 | 0 | 1 | 0 | 0 | 0 | 2 | 0 | 2 | 3 | 0 | 2 | 0 | 0 | 1 | 1 | 1 | 1 | 1 | 0 | 0 | 1 |
| BCYF | 2 | 3 | 6 | 2 | 1 | 2 | 3 | 1 | 0 | 2 | 0 | 2 | 10 | 5 | 1 | 1 | 1 | 7 | 4 | 2 | 1 | 2 | 2 | 2 | 1 | 4 | 2 | 2 | 0 | 1 | 1 | 3 | 1 | 2 | 3 | 2 | 2 | 2 | 2 | 3 | 0 | 1 | 2 | 1 | 1 | 0 | 0 | 0 | 2 | 3 | 10 | 1 | 2 | 1 | 0 | 1 | 0 | 1 | 3 | 3 | 1 | 2 | 1 | 1 | 1 | 1 | 0 | 1 | 0 | 0 | 2 | 4 | 0 | 2 | 0 | 0 | 0 | 0 | 0 | 1 | 0 | 0 | 0 | 1 |
| JKKI | 1 | 5 | 3 | 2 | 1 | 1 | 4 | 2 | 2 | 1 | 0 | 2 | 16 | 6 | 3 | 2 | 1 | 5 | 3 | 3 | 2 | 2 | 1 | 2 | 2 | 3 | 2 | 1 | 0 | 1 | 1 | 1 | 1 | 4 | 3 | 2 | 2 | 2 | 1 | 5 | 0 | 1 | 2 | 3 | 3 | 0 | 2 | 0 | 1 | 2 | 25 | 2 | 1 | 1 | 0 | 1 | 2 | 1 | 5 | 5 | 2 | 2 | 1 | 2 | 1 | 1 | 0 | 1 | 5 | 0 | 4 | 5 | 0 | 3 | 0 | 0 | 1 | 1 | 1 | 1 | 0 | 0 | 0 | 1 |
| USIX | 1 | 3 | 1 | 1 | 1 | 1 | 1 | 0 | 0 | 1 | 0 | 1 | 7 | 3 | 3 | 3 | 0 | 1 | 1 | 2 | 2 | 3 | 1 | 1 | 2 | 3 | 3 | 0 | 0 | 1 | 1 | 1 | 3 | 2 | 2 | 1 | 0 | 3 | 1 | 2 | 0 | 0 | 2 | 1 | 2 | 0 | 1 | 0 | 1 | 2 | 11 | 1 | 2 | 0 | 0 | 0 | 2 | 2 | 3 | 5 | 2 | 1 | 0 | 0 | 0 | 0 | 0 | 1 | 4 | 0 | 3 | 3 | 0 | 2 | 0 | 0 | 2 | 1 | 0 | 0 | 0 | 0 | 0 | 1 |
| QWRA | 1 | 1 | 4 | 1 | 2 | 1 | 0 | 1 | 3 | 1 | 0 | 1 | 12 | 4 | 3 | 2 | 1 | 3 | 3 | 4 | 2 | 3 | 0 | 1 | 1 | 3 | 2 | 1 | 0 | 0 | 0 | 2 | 4 | 4 | 4 | 2 | 1 | 1 | 1 | 7 | 0 | 1 | 2 | 2 | 6 | 0 | 1 | 0 | 1 | 2 | 17 | 1 | 2 | 1 | 0 | 1 | 2 | 1 | 4 | 7 | 2 | 2 | 1 | 0 | 1 | 0 | 2 | 1 | 6 | 0 | 9 | 5 | 1 | 3 | 1 | 1 | 3 | 2 | 1 | 1 | 1 | 0 | 0 | 1 |
| IRYH | 1 | 2 | 3 | 2 | 2 | 1 | 3 | 3 | 4 | 1 | 0 | 2 | 19 | 5 | 5 | 3 | 1 | 3 | 3 | 5 | 2 | 3 | 1 | 1 | 1 | 4 | 4 | 1 | 0 | 3 | 1 | 1 | 3 | 4 | 5 | 2 | 4 | 3 | 2 | 5 | 0 | 1 | 2 | 3 | 7 | 0 | 2 | 0 | 2 | 2 | 36 | 1 | 2 | 1 | 1 | 1 | 4 | 2 | 5 | 7 | 2 | 2 | 1 | 2 | 1 | 1 | 2 | 1 | 7 | 0 | 8 | 6 | 1 | 3 | 2 | 2 | 2 | 2 | 1 | 1 | 1 | 0 | 2 | 1 |
| OFUE | 1 | 2 | 2 | 2 | 2 | 1 | 2 | 2 | 3 | 1 | 0 | 2 | 11 | 5 | 3 | 2 | 1 | 3 | 3 | 1 | 2 | 2 | 1 | 1 | 1 | 4 | 4 | 1 | 0 | 2 | 1 | 1 | 5 | 3 | 4 | 1 | 2 | 3 | 2 | 3 | 0 | 1 | 2 | 4 | 4 | 0 | 2 | 0 | 2 | 2 | 18 | 1 | 2 | 0 | 1 | 1 | 1 | 2 | 5 | 8 | 2 | 1 | 1 | 0 | 1 | 0 | 0 | 1 | 5 | 0 | 9 | 5 | 0 | 2 | 0 | 0 | 2 | 1 | 0 | 1 | 0 | 0 | 0 | 1 |
| XDLL | 2 | 2 | 3 | 2 | 2 | 0 | 3 | 1 | 2 | 2 | 0 | 2 | 13 | 5 | 3 | 2 | 1 | 6 | 4 | 2 | 2 | 2 | 2 | 0 | 1 | 5 | 4 | 1 | 0 | 2 | 2 | 1 | 4 | 2 | 4 | 1 | 2 | 4 | 2 | 4 | 0 | 1 | 2 | 4 | 4 | 0 | 1 | 0 | 1 | 4 | 23 | 0 | 1 | 0 | 1 | 0 | 1 | 1 | 5 | 8 | 2 | 1 | 2 | 1 | 1 | 0 | 2 | 1 | 5 | 0 | 8 | 4 | 0 | 2 | 0 | 0 | 2 | 1 | 0 | 1 | 0 | 0 | 0 | 1 |
| AJUW | 1 | 2 | 4 | 4 | 2 | 1 | 3 | 4 | 0 | 2 | 0 | 1 | 20 | 3 | 3 | 3 | 1 | 3 | 2 | 6 | 2 | 3 | 2 | 1 | 0 | 6 | 5 | 0 | 0 | 1 | 1 | 4 | 6 | 4 | 3 | 4 | 2 | 3 | 2 | 6 | 1 | 2 | 2 | 5 | 5 | 0 | 1 | 0 | 2 | 4 | 31 | 2 | 3 | 1 | 0 | 1 | 1 | 2 | 5 | 7 | 2 | 3 | 1 | 1 | 1 | 2 | 1 | 1 | 5 | 0 | 8 | 5 | 0 | 2 | 1 | 1 | 2 | 2 | 2 | 1 | 2 | 0 | 1 | 1 |
| LBRP | 1 | 2 | 3 | 2 | 1 | 1 | 0 | 0 | 5 | 1 | 0 | 1 | 11 | 4 | 1 | 0 | 0 | 3 | 1 | 4 | 2 | 2 | 1 | 1 | 0 | 4 | 3 | 0 | 0 | 1 | 1 | 2 | 4 | 3 | 3 | 4 | 3 | 2 | 0 | 3 | 0 | 1 | 2 | 3 | 2 | 0 | 0 | 0 | 1 | 3 | 19 | 3 | 2 | 0 | 0 | 1 | 1 | 3 | 6 | 4 | 2 | 2 | 1 | 0 | 2 | 1 | 2 | 1 | 5 | 0 | 6 | 4 | 0 | 3 | 0 | 0 | 2 | 2 | 1 | 1 | 1 | 0 | 1 | 1 |
| AOUJ | 1 | 2 | 4 | 2 | 2 | 1 | 3 | 3 | 0 | 1 | 0 | 1 | 18 | 4 | 3 | 3 | 2 | 2 | 1 | 6 | 2 | 3 | 1 | 4 | 0 | 4 | 3 | 0 | 0 | 1 | 1 | 3 | 3 | 3 | 3 | 5 | 4 | 4 | 2 | 7 | 0 | 1 | 2 | 2 | 2 | 0 | 1 | 0 | 2 | 3 | 27 | 2 | 2 | 0 | 0 | 3 | 1 | 2 | 5 | 9 | 2 | 2 | 1 | 0 | 1 | 2 | 0 | 1 | 7 | 0 | 10 | 3 | 0 | 4 | 0 | 1 | 1 | 1 | 1 | 1 | 1 | 0 | 1 | 1 |
| UTRE | 1 | 0 | 1 | 1 | 0 | 0 | 1 | 0 | 0 | 0 | 0 | 1 | 5 | 2 | 1 | 1 | 0 | 3 | 1 | 0 | 0 | 0 | 0 | 0 | 0 | 0 | 0 | 0 | 0 | 1 | 0 | 0 | 4 | 0 | 0 | 0 | 0 | 2 | 0 | 0 | 0 | 1 | 0 | 0 | 2 | 0 | 0 | 0 | 0 | 1 | 9 | 0 | 1 | 1 | 0 | 0 | 0 | 0 | 0 | 2 | 0 | 1 | 0 | 0 | 0 | 0 | 0 | 1 | 2 | 0 | 4 | 0 | 0 | 0 | 0 | 0 | 1 | 1 | 1 | 0 | 1 | 0 | 0 | 0 |
| FXHG | 1 | 2 | 2 | 1 | 2 | 1 | 1 | 1 | 0 | 1 | 0 | 1 | 14 | 5 | 2 | 1 | 1 | 2 | 2 | 5 | 1 | 3 | 0 | 1 | 1 | 5 | 2 | 1 | 0 | 0 | 0 | 3 | 3 | 2 | 2 | 2 | 3 | 2 | 1 | 5 | 1 | 1 | 2 | 3 | 4 | 0 | 1 | 0 | 2 | 3 | 24 | 1 | 4 | 1 | 0 | 1 | 1 | 1 | 5 | 7 | 1 | 3 | 2 | 1 | 1 | 2 | 0 | 1 | 6 | 0 | 7 | 4 | 0 | 2 | 0 | 1 | 1 | 1 | 0 | 1 | 0 | 0 | 0 | 1 |
| RUIF | 1 | 2 | 3 | 2 | 1 | 1 | 1 | 1 | 1 | 0 | 0 | 1 | 13 | 6 | 2 | 2 | 1 | 1 | 1 | 5 | 2 | 1 | 1 | 1 | 1 | 5 | 4 | 1 | 0 | 1 | 1 | 1 | 4 | 5 | 4 | 2 | 3 | 3 | 0 | 3 | 1 | 0 | 2 | 4 | 3 | 0 | 1 | 0 | 1 | 3 | 22 | 1 | 2 | 0 | 0 | 1 | 1 | 1 | 4 | 6 | 2 | 3 | 1 | 1 | 1 | 1 | 0 | 2 | 5 | 0 | 6 | 6 | 0 | 3 | 1 | 1 | 1 | 1 | 1 | 1 | 1 | 0 | 1 | 1 |
| VIAU | 1 | 2 | 3 | 2 | 1 | 1 | 4 | 3 | 2 | 2 | 0 | 2 | 14 | 6 | 5 | 3 | 1 | 2 | 2 | 4 | 2 | 2 | 1 | 1 | 2 | 4 | 2 | 1 | 0 | 2 | 1 | 1 | 5 | 6 | 5 | 2 | 1 | 2 | 2 | 6 | 0 | 1 | 2 | 3 | 2 | 0 | 5 | 0 | 2 | 2 | 27 | 3 | 1 | 0 | 0 | 1 | 2 | 2 | 5 | 7 | 2 | 4 | 2 | 0 | 2 | 0 | 0 | 1 | 7 | 0 | 6 | 6 | 0 | 2 | 0 | 0 | 2 | 2 | 1 | 1 | 1 | 0 | 1 | 1 |
| XTON | 1 | 0 | 4 | 2 | 2 | 0 | 0 | 0 | 0 | 0 | 0 | 2 | 10 | 5 | 2 | 1 | 0 | 3 | 3 | 4 | 0 | 3 | 1 | 1 | 1 | 4 | 4 | 1 | 0 | 2 | 0 | 1 | 3 | 5 | 5 | 1 | 2 | 2 | 1 | 5 | 0 | 0 | 1 | 1 | 2 | 0 | 1 | 0 | 1 | 2 | 18 | 3 | 1 | 1 | 0 | 3 | 1 | 1 | 1 | 3 | 0 | 2 | 1 | 2 | 1 | 1 | 0 | 1 | 6 | 0 | 7 | 6 | 0 | 2 | 1 | 1 | 1 | 2 | 0 | 0 | 0 | 0 | 1 | 1 |
| XKWQ | 1 | 0 | 3 | 1 | 3 | 0 | 0 | 0 | 0 | 1 | 0 | 2 | 8 | 6 | 2 | 2 | 1 | 2 | 2 | 3 | 1 | 3 | 0 | 1 | 1 | 5 | 4 | 1 | 0 | 0 | 1 | 1 | 2 | 4 | 5 | 2 | 1 | 2 | 1 | 3 | 0 | 1 | 2 | 0 | 1 | 0 | 1 | 0 | 2 | 2 | 14 | 2 | 1 | 0 | 0 | 3 | 0 | 2 | 4 | 4 | 1 | 3 | 1 | 2 | 1 | 1 | 1 | 1 | 5 | 0 | 7 | 6 | 0 | 2 | 1 | 1 | 1 | 2 | 0 | 0 | 0 | 0 | 0 | 1 |
| VJDZ | 1 | 3 | 4 | 3 | 2 | 0 | 4 | 2 | 1 | 1 | 0 | 2 | 23 | 5 | 6 | 6 | 1 | 3 | 3 | 6 | 3 | 3 | 1 | 3 | 1 | 4 | 3 | 1 | 0 | 1 | 1 | 1 | 6 | 8 | 7 | 2 | 5 | 3 | 3 | 8 | 1 | 1 | 2 | 2 | 5 | 0 | 5 | 0 | 4 | 2 | 41 | 4 | 1 | 1 | 0 | 1 | 3 | 2 | 5 | 8 | 2 | 3 | 2 | 2 | 1 | 2 | 2 | 1 | 7 | 0 | 10 | 8 | 1 | 4 | 2 | 2 | 2 | 2 | 1 | 1 | 1 | 0 | 2 | 1 |
| PZIF | 1 | 3 | 5 | 3 | 2 | 1 | 2 | 1 | 2 | 1 | 0 | 2 | 18 | 7 | 6 | 5 | 2 | 4 | 4 | 7 | 3 | 3 | 1 | 2 | 1 | 4 | 3 | 1 | 0 | 2 | 1 | 2 | 4 | 6 | 6 | 4 | 3 | 3 | 1 | 5 | 1 | 1 | 2 | 4 | 2 | 0 | 3 | 0 | 2 | 2 | 36 | 3 | 1 | 1 | 0 | 3 | 2 | 2 | 5 | 8 | 2 | 3 | 2 | 1 | 2 | 1 | 1 | 1 | 8 | 0 | 9 | 7 | 0 | 3 | 2 | 2 | 2 | 2 | 1 | 1 | 1 | 0 | 1 | 1 |
| MWAN | 1 | 1 | 1 | 1 | 1 | 1 | 2 | 1 | 1 | 1 | 0 | 2 | 4 | 5 | 3 | 2 | 0 | 2 | 2 | 4 | 1 | 3 | 1 | 1 | 1 | 4 | 3 | 1 | 0 | 0 | 1 | 1 | 1 | 5 | 5 | 2 | 1 | 3 | 0 | 3 | 0 | 0 | 2 | 2 | 0 | 0 | 1 | 0 | 3 | 2 | 12 | 3 | 1 | 1 | 0 | 2 | 1 | 1 | 3 | 6 | 1 | 3 | 2 | 0 | 1 | 1 | 1 | 1 | 1 | 0 | 5 | 6 | 0 | 1 | 0 | 0 | 2 | 2 | 0 | 1 | 0 | 0 | 0 | 1 |
| OTQG | 1 | 2 | 3 | 1 | 1 | 1 | 1 | 0 | 1 | 1 | 0 | 2 | 12 | 5 | 6 | 2 | 0 | 2 | 2 | 3 | 2 | 4 | 1 | 1 | 1 | 4 | 3 | 0 | 0 | 1 | 1 | 1 | 1 | 4 | 4 | 3 | 3 | 3 | 0 | 5 | 0 | 1 | 0 | 1 | 1 | 0 | 1 | 0 | 2 | 2 | 21 | 2 | 1 | 0 | 0 | 3 | 2 | 1 | 5 | 8 | 2 | 2 | 1 | 1 | 1 | 1 | 3 | 1 | 4 | 0 | 7 | 5 | 0 | 1 | 0 | 0 | 3 | 2 | 1 | 1 | 1 | 0 | 0 | 1 |
| BZSH | 1 | 3 | 3 | 2 | 2 | 1 | 3 | 4 | 0 | 1 | 0 | 2 | 30 | 6 | 4 | 4 | 2 | 3 | 3 | 6 | 2 | 3 | 1 | 2 | 2 | 5 | 3 | 2 | 0 | 3 | 1 | 1 | 3 | 7 | 6 | 4 | 5 | 3 | 2 | 5 | 1 | 1 | 2 | 4 | 2 | 0 | 3 | 0 | 3 | 2 | 53 | 1 | 1 | 0 | 0 | 2 | 1 | 3 | 5 | 8 | 2 | 3 | 1 | 2 | 2 | 2 | 2 | 1 | 8 | 0 | 9 | 8 | 1 | 4 | 1 | 1 | 2 | 1 | 1 | 1 | 1 | 1 | 1 | 1 |
| ENAU | 2 | 3 | 3 | 2 | 1 | 2 | 4 | 0 | 0 | 2 | 0 | 2 | 3 | 4 | 1 | 0 | 0 | 6 | 2 | 2 | 0 | 1 | 0 | 3 | 0 | 3 | 1 | 1 | 0 | 0 | 0 | 3 | 0 | 1 | 2 | 2 | 0 | 1 | 0 | 1 | 0 | 0 | 1 | 0 | 0 | 0 | 0 | 0 | 0 | 2 | 11 | 1 | 1 | 1 | 0 | 1 | 0 | 1 | 0 | 1 | 0 | 2 | 1 | 1 | 1 | 0 | 0 | 0 | 2 | 0 | 2 | 4 | 0 | 2 | 0 | 0 | 1 | 1 | 0 | 0 | 0 | 0 | 0 | 0 |
| MXDS | 1 | 2 | 3 | 3 | 2 | 1 | 1 | 3 | 1 | 1 | 0 | 2 | 12 | 5 | 3 | 3 | 1 | 4 | 4 | 4 | 1 | 3 | 0 | 3 | 3 | 6 | 3 | 1 | 0 | 0 | 0 | 2 | 1 | 5 | 2 | 2 | 2 | 2 | 0 | 3 | 0 | 0 | 2 | 2 | 2 | 0 | 1 | 0 | 1 | 3 | 24 | 1 | 2 | 1 | 0 | 0 | 2 | 2 | 5 | 7 | 1 | 4 | 1 | 2 | 2 | 0 | 0 | 1 | 6 | 0 | 4 | 4 | 0 | 3 | 1 | 1 | 2 | 2 | 0 | 1 | 0 | 0 | 1 | 2 |
| DZPJ | 1 | 3 | 3 | 2 | 2 | 1 | 5 | 0 | 0 | 1 | 0 | 2 | 19 | 5 | 4 | 4 | 1 | 4 | 3 | 5 | 2 | 2 | 1 | 1 | 3 | 5 | 4 | 2 | 0 | 1 | 1 | 2 | 4 | 7 | 7 | 4 | 2 | 2 | 1 | 4 | 0 | 1 | 2 | 3 | 4 | 0 | 3 | 0 | 2 | 2 | 32 | 1 | 2 | 1 | 0 | 1 | 2 | 2 | 5 | 8 | 1 | 2 | 1 | 1 | 1 | 1 | 0 | 1 | 6 | 0 | 8 | 8 | 1 | 3 | 1 | 1 | 2 | 2 | 1 | 1 | 1 | 0 | 1 | 1 |
| JMUI | 1 | 1 | 2 | 2 | 2 | 0 | 3 | 1 | 2 | 1 | 0 | 2 | 5 | 5 | 2 | 1 | 2 | 2 | 2 | 5 | 1 | 2 | 1 | 1 | 2 | 5 | 3 | 1 | 0 | 1 | 1 | 1 | 5 | 1 | 2 | 2 | 1 | 3 | 1 | 3 | 0 | 1 | 2 | 1 | 1 | 0 | 0 | 0 | 2 | 2 | 15 | 1 | 2 | 1 | 1 | 2 | 0 | 1 | 3 | 3 | 1 | 2 | 2 | 1 | 1 | 0 | 0 | 1 | 5 | 0 | 5 | 4 | 1 | 2 | 0 | 1 | 2 | 2 | 1 | 1 | 0 | 0 | 1 | 1 |
| VFIV | 1 | 2 | 2 | 2 | 1 | 1 | 2 | 1 | 1 | 1 | 0 | 3 | 14 | 6 | 1 | 1 | 1 | 2 | 2 | 4 | 3 | 3 | 1 | 1 | 1 | 5 | 3 | 1 | 0 | 1 | 1 | 1 | 4 | 6 | 6 | 2 | 3 | 1 | 2 | 4 | 0 | 1 | 1 | 3 | 4 | 0 | 2 | 0 | 2 | 3 | 20 | 0 | 1 | 0 | 1 | 2 | 1 | 1 | 5 | 9 | 2 | 2 | 1 | 2 | 1 | 0 | 0 | 1 | 9 | 0 | 9 | 7 | 1 | 3 | 1 | 1 | 2 | 2 | 1 | 1 | 1 | 0 | 1 | 1 |

# Supplementary Table S3 (pg 2/4, upper right quadrant)

| sample code | PF04191 | PF11899 | ec2.4.1.241 | ec2.4.1.46 | ec2.3.1.158 | ec2.3.1.20 | ec3.1.1.3 | PF03982 | ec2.5.1.1 | ec2.5.1.10 | ec2.5.1.29 | ec2.5.1.32 | ec5.3.3.2 | ec1.1.1.267 | ec1.17.1.2 | ec1.17.7.1 | ec2.2.1.7 | ec2.7.1.148 | ec2.7.7.60 | ec4.6.1.12 | ec1.1.1.34 | ec1.1.1.88 | ec2.3.3.10 | ec2.7.1.36 | ec2.7.4.2 | ec4.1.1.33 | ec2.1.1.14 | PF00809 | PF01717 | PF02310 | PF02574 | PF02607 | PF02965 | PF08267 | ec1.1.1.65 | ec1.2.1.72 | ec1.4.3.5 | ec2.3.1.47 | ec2.6.1.52 | ec2.7.1.35 | ec3.1.3.74 | ec4.2.3.1 | PF01174 | ec2.6.1.62 | ec2.8.1.6 | ec6.2.1.14 | ec6.3.3.3 | ec6.3.4.10 | ec6.3.4.11 | ec6.3.4.15 | ec6.3.4.9 | ec1.4.3.19 | ec2.5.1.3 | ec2.7.1.49 | ec2.7.1.50 | ec2.7.4.16 | ec2.7.4.7 | ec2.7.6.2 | ec2.8.1.7 | ec3.5.99.2 | ec3.6.1.15 | ec3.6.1.28 | PF00899 | PF01946 | PF01964 | PF02110 | PF00636 | PF02170 | PF02171 | PF03368 | PF05183 | PF02301 | PF03962 | PF07106 | SPO11 | PF00078 | PF00270 | PF00271 | PF00493 | PF02889 | PF08241 | PF08423 | PF13489 |
|---|---|---|---|---|---|---|---|---|---|---|---|---|---|---|---|---|---|---|---|---|---|---|---|---|---|---|---|---|---|---|---|---|---|---|---|---|---|---|---|---|---|---|---|---|---|---|---|---|---|---|---|---|---|---|---|---|---|---|---|---|---|---|---|---|---|---|---|---|---|---|---|---|---|---|---|---|---|---|---|---|---|---|---|
| ROZZ | 0 | 1 | 0 | 0 | 0 | 0 | 5 | 2 | 3 | 2 | 5 | 3 | 1 | 1 | 1 | 1 | 1 | 1 | 0 | 1 | 0 | 0 | 1 | 2 | 1 | 1 | 0 | 1 | 0 | 1 | 1 | 1 | 1 | 0 | 3 | 2 | 0 | 2 | 1 | 0 | 2 | 1 | 0 | 1 | 0 | 1 | 0 | 0 | 0 | 0 | 0 | 0 | 0 | 0 | 0 | 0 | 1 | 3 | 1 | 1 | 0 | 5 | 1 | 0 | 0 | 0 | 1 | 0 | 0 | 0 | 0 | 0 | 0 | 0 | 0 | 2 | 23 | 45 | 5 | 1 | 41 | 1 | 25 |
| BAKF | 0 | 1 | 0 | 1 | 0 | 0 | 1 | 1 | 2 | 2 | 1 | 1 | 0 | 1 | 1 | 1 | 1 | 0 | 0 | 0 | 0 | 0 | 1 | 0 | 0 | 1 | 0 | 0 | 0 | 1 | 0 | 1 | 2 | 0 | 2 | 1 | 0 | 3 | 1 | 0 | 1 | 1 | 0 | 1 | 0 | 0 | 0 | 0 | 0 | 0 | 0 | 0 | 0 | 1 | 0 | 0 | 1 | 3 | 0 | 1 | 0 | 6 | 1 | 1 | 0 | 1 | 1 | 0 | 0 | 0 | 0 | 2 | 0 | 0 | 0 | 2 | 23 | 45 | 4 | 2 | 35 | 2 | 12 |
| IRZA | 0 | 1 | 1 | 1 | 0 | 1 | 9 | 1 | 6 | 5 | 5 | 2 | 1 | 1 | 1 | 1 | 1 | 1 | 0 | 1 | 1 | 1 | 1 | 2 | 1 | 1 | 0 | 2 | 0 | 2 | 2 | 1 | 2 | 0 | 4 | 3 | 0 | 2 | 2 | 1 | 3 | 1 | 0 | 0 | 0 | 0 | 0 | 0 | 0 | 0 | 0 | 1 | 1 | 2 | 0 | 1 | 0 | 3 | 2 | 0 | 0 | 6 | 1 | 0 | 0 | 2 | 1 | 0 | 0 | 0 | 0 | 1 | 0 | 0 | 0 | 2 | 43 | 80 | 0 | 0 | 59 | 1 | 37 |
| IAYV | 0 | 0 | 1 | 1 | 0 | 1 | 3 | 0 | 2 | 2 | 2 | 1 | 1 | 0 | 1 | 1 | 0 | 0 | 0 | 0 | 0 | 0 | 1 | 3 | 0 | 0 | 0 | 1 | 0 | 4 | 3 | 1 | 1 | 0 | 3 | 3 | 0 | 1 | 1 | 0 | 1 | 1 | 0 | 1 | 0 | 1 | 0 | 0 | 0 | 0 | 0 | 0 | 0 | 0 | 0 | 0 | 0 | 2 | 1 | 1 | 0 | 6 | 0 | 0 | 0 | 0 | 2 | 0 | 0 | 0 | 0 | 1 | 0 | 0 | 0 | 1 | 17 | 27 | 0 | 0 | 38 | 1 | 16 |
| BAJW | 0 | 1 | 2 | 1 | 0 | 1 | 7 | 0 | 6 | 5 | 6 | 3 | 1 | 1 | 1 | 1 | 1 | 1 | 0 | 1 | 1 | 0 | 3 | 2 | 0 | 1 | 0 | 3 | 0 | 2 | 1 | 1 | 1 | 0 | 1 | 5 | 0 | 3 | 2 | 3 | 2 | 4 | 0 | 1 | 0 | 0 | 0 | 0 | 0 | 0 | 0 | 1 | 1 | 0 | 0 | 1 | 1 | 2 | 2 | 2 | 1 | 10 | 2 | 0 | 0 | 0 | 2 | 1 | 2 | 0 | 1 | 2 | 0 | 1 | 1 | 1 | 49 | 118 | 7 | 5 | 75 | 1 | 43 |
| LXRN | 0 | 1 | 1 | 1 | 0 | 1 | 4 | 0 | 2 | 2 | 2 | 1 | 1 | 1 | 1 | 1 | 1 | 0 | 0 | 1 | 2 | 0 | 0 | 1 | 0 | 2 | 0 | 2 | 0 | 2 | 1 | 1 | 2 | 0 | 3 | 4 | 0 | 3 | 1 | 1 | 1 | 2 | 1 | 0 | 0 | 0 | 0 | 0 | 0 | 0 | 1 | 1 | 1 | 0 | 0 | 1 | 1 | 1 | 2 | 2 | 0 | 10 | 1 | 0 | 0 | 0 | 0 | 0 | 2 | 0 | 0 | 1 | 1 | 1 | 0 | 0 | 34 | 90 | 5 | 3 | 45 | 1 | 23 |
| EBWI | 0 | 1 | 0 | 0 | 1 | 0 | 13 | 0 | 5 | 4 | 4 | 1 | 2 | 1 | 1 | 1 | 1 | 0 | 0 | 1 | 0 | 0 | 1 | 1 | 1 | 1 | 0 | 2 | 0 | 2 | 1 | 1 | 2 | 0 | 5 | 2 | 0 | 2 | 2 | 1 | 3 | 1 | 0 | 2 | 0 | 1 | 0 | 0 | 1 | 0 | 2 | 0 | 0 | 0 | 0 | 0 | 0 | 4 | 1 | 1 | 0 | 9 | 1 | 1 | 0 | 0 | 0 | 0 | 0 | 0 | 0 | 3 | 0 | 0 | 0 | 1 | 16 | 41 | 1 | 0 | 52 | 1 | 33 |
| RFAD | 0 | 0 | 0 | 1 | 0 | 0 | 2 | 0 | 4 | 4 | 3 | 1 | 2 | 1 | 1 | 1 | 1 | 1 | 0 | 1 | 1 | 0 | 1 | 1 | 1 | 1 | 0 | 2 | 0 | 2 | 1 | 1 | 2 | 0 | 3 | 3 | 0 | 1 | 1 | 1 | 1 | 1 | 0 | 1 | 0 | 0 | 0 | 0 | 0 | 0 | 0 | 1 | 0 | 0 | 0 | 0 | 0 | 3 | 1 | 0 | 0 | 7 | 0 | 1 | 0 | 0 | 0 | 0 | 0 | 0 | 0 | 1 | 0 | 0 | 0 | 0 | 20 | 52 | 1 | 2 | 0 | 2 | 0 |
| LLEN | 0 | 1 | 0 | 1 | 0 | 0 | 1 | 2 | 4 | 4 | 3 | 2 | 2 | 1 | 2 | 2 | 1 | 1 | 0 | 1 | 1 | 0 | 1 | 1 | 1 | 1 | 3 | 2 | 1 | 2 | 1 | 1 | 2 | 1 | 3 | 6 | 0 | 1 | 1 | 1 | 2 | 1 | 0 | 1 | 0 | 0 | 0 | 0 | 1 | 0 | 1 | 2 | 0 | 0 | 0 | 0 | 0 | 2 | 1 | 1 | 0 | 8 | 1 | 1 | 0 | 0 | 0 | 0 | 0 | 0 | 0 | 2 | 1 | 1 | 0 | 0 | 23 | 77 | 5 | 3 | 66 | 2 | 32 |
| NMAK | 0 | 1 | 0 | 1 | 1 | 0 | 10 | 2 | 4 | 4 | 3 | 2 | 3 | 1 | 1 | 1 | 1 | 1 | 1 | 1 | 1 | 0 | 1 | 2 | 1 | 1 | 0 | 4 | 0 | 2 | 1 | 1 | 2 | 0 | 5 | 3 | 0 | 2 | 1 | 1 | 2 | 1 | 0 | 1 | 0 | 0 | 0 | 0 | 1 | 0 | 2 | 2 | 1 | 0 | 0 | 1 | 1 | 3 | 2 | 1 | 0 | 12 | 1 | 1 | 0 | 0 | 0 | 0 | 0 | 0 | 0 | 2 | 1 | 1 | 1 | 1 | 50 | 103 | 7 | 0 | 73 | 3 | 39 |
| BOGT | 0 | 1 | 1 | 1 | 0 | 0 | 12 | 2 | 5 | 5 | 4 | 2 | 2 | 1 | 1 | 1 | 1 | 1 | 0 | 1 | 2 | 0 | 2 | 1 | 1 | 1 | 0 | 2 | 0 | 2 | 1 | 1 | 1 | 0 | 3 | 2 | 1 | 3 | 1 | 2 | 1 | 1 | 0 | 1 | 0 | 2 | 0 | 0 | 1 | 0 | 1 | 1 | 1 | 0 | 0 | 1 | 1 | 2 | 1 | 2 | 0 | 11 | 1 | 1 | 0 | 0 | 2 | 2 | 2 | 0 | 1 | 3 | 1 | 1 | 0 | 3 | 39 | 78 | 7 | 1 | 76 | 2 | 49 |
| RAPY | 0 | 0 | 2 | 2 | 0 | 0 | 7 | 3 | 5 | 5 | 4 | 2 | 2 | 1 | 1 | 1 | 1 | 1 | 0 | 1 | 1 | 0 | 1 | 0 | 1 | 1 | 1 | 1 | 1 | 2 | 1 | 1 | 1 | 1 | 3 | 7 | 2 | 3 | 1 | 2 | 2 | 1 | 0 | 1 | 0 | 0 | 0 | 0 | 1 | 0 | 2 | 1 | 1 | 0 | 0 | 1 | 1 | 3 | 1 | 1 | 1 | 7 | 1 | 0 | 1 | 0 | 1 | 1 | 1 | 0 | 0 | 1 | 1 | 1 | 1 | 9 | 42 | 77 | 5 | 3 | 70 | 1 | 45 |
| LLXJ | 1 | 1 | 1 | 4 | 1 | 1 | 7 | 2 | 6 | 5 | 4 | 1 | 2 | 1 | 1 | 1 | 3 | 1 | 0 | 1 | 2 | 0 | 1 | 3 | 1 | 0 | 0 | 2 | 0 | 5 | 3 | 1 | 4 | 0 | 5 | 3 | 1 | 7 | 1 | 2 | 1 | 1 | 1 | 1 | 0 | 1 | 1 | 1 | 1 | 1 | 1 | 2 | 1 | 0 | 0 | 1 | 1 | 4 | 2 | 2 | 1 | 11 | 1 | 0 | 1 | 0 | 2 | 1 | 1 | 0 | 1 | 1 | 2 | 1 | 1 | 5 | 49 | 92 | 5 | 3 | 58 | 2 | 36 |
| JJZR | 2 | 0 | 2 | 3 | 0 | 3 | 6 | 0 | 4 | 3 | 3 | 1 | 1 | 1 | 1 | 1 | 1 | 1 | 1 | 1 | 0 | 0 | 1 | 1 | 0 | 0 | 0 | 2 | 0 | 2 | 1 | 1 | 1 | 0 | 3 | 4 | 1 | 3 | 1 | 2 | 1 | 1 | 0 | 1 | 0 | 1 | 0 | 0 | 0 | 0 | 1 | 1 | 1 | 0 | 0 | 1 | 1 | 4 | 0 | 1 | 0 | 5 | 1 | 0 | 1 | 0 | 4 | 3 | 2 | 0 | 3 | 3 | 1 | 1 | 3 | 9 | 45 | 84 | 8 | 0 | 57 | 2 | 30 |
| RSOF | 1 | 0 | 0 | 1 | 0 | 1 | 4 | 1 | 3 | 2 | 1 | 1 | 1 | 1 | 1 | 1 | 1 | 1 | 0 | 1 | 0 | 0 | 0 | 0 | 0 | 0 | 0 | 1 | 0 | 1 | 1 | 1 | 1 | 0 | 3 | 2 | 1 | 3 | 1 | 1 | 1 | 1 | 0 | 1 | 0 | 0 | 0 | 0 | 0 | 0 | 0 | 1 | 1 | 0 | 0 | 1 | 0 | 3 | 0 | 1 | 0 | 5 | 1 | 0 | 1 | 0 | 1 | 3 | 4 | 0 | 0 | 1 | 1 | 1 | 0 | 6 | 27 | 57 | 0 | 0 | 26 | 1 | 16 |
| PVGP | 0 | 0 | 1 | 0 | 0 | 1 | 3 | 0 | 1 | 1 | 1 | 1 | 0 | 1 | 1 | 1 | 1 | 0 | 0 | 1 | 0 | 0 | 0 | 1 | 0 | 0 | 1 | 2 | 1 | 2 | 1 | 1 | 2 | 1 | 3 | 1 | 1 | 3 | 1 | 0 | 0 | 1 | 0 | 2 | 0 | 0 | 0 | 0 | 0 | 0 | 1 | 1 | 0 | 0 | 0 | 0 | 0 | 2 | 0 | 1 | 0 | 2 | 1 | 0 | 1 | 0 | 2 | 2 | 3 | 0 | 0 | 1 | 1 | 0 | 1 | 3 | 28 | 57 | 0 | 0 | 25 | 2 | 13 |
| OBUY | 1 | 0 | 1 | 0 | 0 | 1 | 2 | 0 | 3 | 2 | 2 | 1 | 0 | 1 | 1 | 1 | 1 | 1 | 0 | 1 | 0 | 0 | 0 | 1 | 0 | 0 | 1 | 3 | 1 | 2 | 2 | 1 | 1 | 1 | 2 | 2 | 1 | 3 | 1 | 0 | 0 | 1 | 0 | 2 | 0 | 0 | 0 | 0 | 0 | 0 | 0 | 1 | 0 | 0 | 0 | 0 | 0 | 2 | 0 | 1 | 0 | 2 | 1 | 0 | 1 | 0 | 3 | 2 | 3 | 0 | 0 | 1 | 0 | 0 | 0 | 10 | 25 | 58 | 3 | 0 | 26 | 2 | 13 |
| ZULJ | 1 | 0 | 0 | 0 | 1 | 0 | 1 | 0 | 2 | 1 | 1 | 1 | 0 | 1 | 1 | 1 | 1 | 1 | 0 | 1 | 0 | 0 | 0 | 1 | 0 | 0 | 1 | 1 | 1 | 2 | 1 | 1 | 1 | 1 | 5 | 6 | 1 | 3 | 2 | 0 | 1 | 1 | 0 | 2 | 0 | 0 | 0 | 0 | 0 | 0 | 0 | 0 | 1 | 0 | 0 | 1 | 0 | 2 | 0 | 2 | 0 | 10 | 0 | 0 | 1 | 0 | 3 | 6 | 8 | 0 | 0 | 1 | 2 | 1 | 1 | 8 | 42 | 75 | 13 | 4 | 33 | 3 | 18 |
| XAXW | 2 | 0 | 1 | 3 | 0 | 1 | 6 | 0 | 2 | 1 | 2 | 1 | 1 | 1 | 1 | 1 | 1 | 1 | 1 | 1 | 0 | 0 | 1 | 0 | 0 | 0 | 0 | 1 | 0 | 1 | 2 | 1 | 1 | 0 | 4 | 4 | 1 | 2 | 1 | 1 | 0 | 1 | 1 | 1 | 0 | 1 | 0 | 0 | 1 | 0 | 0 | 0 | 0 | 0 | 0 | 0 | 1 | 3 | 0 | 0 | 1 | 6 | 1 | 0 | 1 | 0 | 3 | 2 | 2 | 0 | 0 | 1 | 1 | 1 | 1 | 0 | 38 | 62 | 6 | 0 | 40 | 2 | 23 |
| JEBK | 2 | 0 | 1 | 3 | 0 | 2 | 4 | 0 | 3 | 3 | 2 | 3 | 1 | 1 | 1 | 1 | 1 | 1 | 0 | 1 | 0 | 0 | 1 | 2 | 0 | 0 | 0 | 3 | 0 | 1 | 2 | 1 | 2 | 0 | 4 | 4 | 1 | 3 | 2 | 2 | 1 | 1 | 1 | 1 | 0 | 1 | 0 | 0 | 1 | 0 | 2 | 2 | 1 | 0 | 0 | 1 | 0 | 3 | 1 | 1 | 1 | 6 | 1 | 0 | 1 | 0 | 4 | 2 | 2 | 0 | 1 | 2 | 1 | 1 | 2 | 4 | 47 | 76 | 7 | 2 | 49 | 1 | 27 |
| IHJY | 1 | 0 | 1 | 3 | 0 | 1 | 3 | 0 | 3 | 3 | 2 | 1 | 1 | 1 | 1 | 1 | 1 | 1 | 0 | 1 | 0 | 0 | 1 | 0 | 0 | 0 | 0 | 2 | 0 | 1 | 2 | 1 | 1 | 0 | 4 | 2 | 1 | 3 | 1 | 2 | 1 | 1 | 0 | 1 | 0 | 1 | 0 | 0 | 0 | 0 | 1 | 2 | 1 | 0 | 0 | 1 | 1 | 2 | 1 | 1 | 0 | 5 | 1 | 0 | 0 | 0 | 2 | 2 | 2 | 0 | 0 | 1 | 1 | 0 | 2 | 4 | 41 | 71 | 5 | 1 | 42 | 1 | 23 |
| CKXF | 2 | 0 | 2 | 4 | 0 | 1 | 7 | 0 | 3 | 3 | 2 | 2 | 1 | 1 | 1 | 1 | 1 | 1 | 0 | 1 | 0 | 0 | 1 | 2 | 0 | 0 | 1 | 3 | 1 | 1 | 3 | 1 | 2 | 1 | 5 | 2 | 1 | 3 | 2 | 2 | 1 | 1 | 0 | 1 | 0 | 1 | 1 | 1 | 1 | 1 | 3 | 2 | 1 | 0 | 0 | 1 | 1 | 4 | 1 | 1 | 1 | 6 | 1 | 0 | 1 | 0 | 4 | 2 | 2 | 0 | 0 | 2 | 1 | 1 | 2 | 8 | 48 | 80 | 8 | 2 | 61 | 2 | 39 |
| SBLT | 2 | 0 | 1 | 4 | 0 | 2 | 7 | 0 | 3 | 3 | 2 | 1 | 1 | 1 | 1 | 1 | 1 | 1 | 0 | 1 | 0 | 0 | 1 | 2 | 0 | 0 | 1 | 3 | 1 | 0 | 0 | 1 | 2 | 1 | 4 | 3 | 1 | 3 | 2 | 2 | 0 | 1 | 0 | 1 | 0 | 1 | 1 | 1 | 1 | 1 | 2 | 1 | 1 | 0 | 0 | 1 | 1 | 2 | 1 | 1 | 0 | 6 | 1 | 0 | 1 | 0 | 4 | 3 | 3 | 1 | 0 | 2 | 1 | 1 | 2 | 11 | 45 | 76 | 7 | 2 | 50 | 2 | 27 |
| UGPM | 2 | 0 | 1 | 3 | 0 | 2 | 8 | 0 | 5 | 3 | 4 | 1 | 1 | 1 | 1 | 1 | 1 | 1 | 0 | 1 | 0 | 0 | 1 | 2 | 0 | 0 | 1 | 3 | 1 | 1 | 1 | 1 | 2 | 1 | 5 | 4 | 1 | 5 | 2 | 1 | 1 | 1 | 0 | 1 | 0 | 1 | 1 | 1 | 1 | 1 | 1 | 1 | 0 | 0 | 0 | 0 | 1 | 3 | 1 | 1 | 1 | 5 | 1 | 0 | 1 | 0 | 4 | 3 | 3 | 0 | 0 | 2 | 1 | 1 | 2 | 3 | 44 | 81 | 6 | 3 | 46 | 2 | 25 |
| PYDB | 2 | 0 | 2 | 4 | 0 | 2 | 12 | 0 | 3 | 3 | 2 | 2 | 1 | 1 | 1 | 1 | 1 | 1 | 0 | 1 | 0 | 0 | 1 | 2 | 0 | 0 | 1 | 4 | 1 | 1 | 3 | 1 | 2 | 0 | 3 | 6 | 1 | 3 | 2 | 2 | 1 | 1 | 0 | 1 | 0 | 1 | 1 | 1 | 1 | 1 | 2 | 2 | 1 | 0 | 0 | 1 | 1 | 2 | 1 | 1 | 1 | 6 | 1 | 0 | 1 | 0 | 4 | 2 | 2 | 1 | 1 | 2 | 1 | 1 | 1 | 7 | 49 | 82 | 9 | 2 | 57 | 2 | 36 |
| URSB | 2 | 0 | 2 | 3 | 0 | 2 | 12 | 0 | 3 | 3 | 2 | 1 | 1 | 1 | 1 | 1 | 1 | 1 | 0 | 1 | 0 | 0 | 1 | 2 | 0 | 0 | 1 | 2 | 1 | 1 | 2 | 1 | 1 | 1 | 3 | 2 | 1 | 4 | 2 | 1 | 1 | 1 | 0 | 1 | 0 | 0 | 1 | 1 | 1 | 1 | 2 | 2 | 1 | 0 | 0 | 1 | 0 | 2 | 1 | 1 | 1 | 5 | 1 | 0 | 1 | 0 | 3 | 2 | 2 | 1 | 2 | 1 | 1 | 1 | 2 | 2 | 45 | 76 | 8 | 2 | 59 | 2 | 32 |
| IKIZ | 2 | 0 | 2 | 4 | 0 | 2 | 8 | 0 | 4 | 3 | 3 | 1 | 1 | 1 | 1 | 1 | 1 | 1 | 0 | 1 | 0 | 0 | 1 | 2 | 0 | 0 | 1 | 1 | 1 | 1 | 2 | 1 | 1 | 1 | 3 | 2 | 1 | 3 | 2 | 2 | 1 | 1 | 1 | 1 | 0 | 1 | 1 | 1 | 1 | 1 | 1 | 2 | 1 | 0 | 0 | 1 | 1 | 2 | 1 | 1 | 1 | 5 | 1 | 0 | 1 | 0 | 5 | 2 | 2 | 1 | 1 | 1 | 1 | 1 | 1 | 0 | 41 | 76 | 8 | 1 | 48 | 2 | 30 |
| ZJOJ | 2 | 0 | 2 | 3 | 0 | 2 | 11 | 0 | 6 | 7 | 4 | 4 | 1 | 2 | 2 | 2 | 2 | 1 | 0 | 1 | 0 | 0 | 1 | 2 | 0 | 1 | 2 | 3 | 2 | 2 | 3 | 3 | 6 | 2 | 4 | 14 | 3 | 3 | 2 | 2 | 2 | 1 | 0 | 1 | 0 | 1 | 1 | 1 | 1 | 1 | 2 | 2 | 1 | 0 | 0 | 1 | 1 | 6 | 1 | 1 | 1 | 8 | 1 | 0 | 2 | 0 | 4 | 2 | 2 | 1 | 0 | 3 | 1 | 1 | 1 | 3 | 53 | 98 | 9 | 4 | 74 | 3 | 41 |
| FTRP | 2 | 0 | 2 | 4 | 0 | 3 | 9 | 0 | 3 | 3 | 2 | 2 | 1 | 1 | 1 | 1 | 1 | 1 | 0 | 1 | 0 | 0 | 1 | 1 | 0 | 0 | 1 | 4 | 1 | 1 | 2 | 1 | 2 | 1 | 5 | 2 | 1 | 3 | 2 | 1 | 1 | 1 | 0 | 1 | 0 | 1 | 0 | 0 | 1 | 0 | 1 | 2 | 0 | 0 | 0 | 0 | 1 | 2 | 1 | 1 | 0 | 5 | 1 | 0 | 1 | 0 | 4 | 2 | 2 | 1 | 0 | 2 | 1 | 1 | 3 | 11 | 46 | 73 | 7 | 2 | 54 | 2 | 35 |
| QFND | 1 | 0 | 0 | 1 | 0 | 0 | 10 | 0 | 4 | 4 | 3 | 2 | 1 | 1 | 1 | 1 | 1 | 0 | 0 | 1 | 1 | 0 | 1 | 0 | 1 | 1 | 0 | 1 | 0 | 2 | 1 | 1 | 1 | 0 | 3 | 2 | 1 | 2 | 1 | 2 | 2 | 1 | 0 | 2 | 2 | 0 | 0 | 0 | 0 | 0 | 0 | 2 | 1 | 0 | 0 | 1 | 1 | 3 | 0 | 1 | 2 | 7 | 1 | 1 | 1 | 0 | 2 | 2 | 2 | 0 | 0 | 1 | 0 | 0 | 0 | 10 | 26 | 53 | 1 | 0 | 47 | 1 | 31 |
| POOW | 2 | 0 | 0 | 1 | 0 | 0 | 11 | 4 | 3 | 3 | 2 | 2 | 1 | 1 | 1 | 1 | 1 | 1 | 0 | 1 | 1 | 0 | 0 | 1 | 0 | 1 | 0 | 2 | 0 | 2 | 3 | 1 | 1 | 0 | 3 | 5 | 1 | 3 | 2 | 2 | 2 | 1 | 0 | 2 | 2 | 0 | 1 | 1 | 1 | 1 | 1 | 1 | 1 | 0 | 0 | 1 | 1 | 3 | 0 | 1 | 0 | 8 | 1 | 1 | 1 | 0 | 4 | 5 | 4 | 0 | 0 | 2 | 1 | 0 | 1 | 1 | 41 | 84 | 4 | 3 | 71 | 1 | 48 |
| PQED | 1 | 1 | 0 | 1 | 1 | 3 | 11 | 3 | 4 | 4 | 3 | 1 | 2 | 1 | 1 | 1 | 1 | 1 | 0 | 1 | 1 | 0 | 1 | 2 | 2 | 1 | 0 | 2 | 0 | 2 | 3 | 1 | 1 | 0 | 8 | 4 | 0 | 3 | 1 | 2 | 3 | 1 | 0 | 2 | 3 | 1 | 1 | 1 | 1 | 1 | 1 | 3 | 1 | 0 | 0 | 1 | 1 | 5 | 2 | 1 | 0 | 10 | 1 | 1 | 0 | 0 | 11 | 9 | 6 | 0 | 1 | 2 | 1 | 1 | 1 | 1 | 50 | 102 | 8 | 3 | 64 | 2 | 53 |
| CBNG | 0 | 1 | 0 | 0 | 0 | 1 | 2 | 1 | 2 | 1 | 1 | 2 | 1 | 1 | 1 | 1 | 0 | 1 | 1 | 1 | 0 | 0 | 0 | 0 | 0 | 0 | 0 | 1 | 0 | 2 | 1 | 1 | 1 | 0 | 3 | 2 | 1 | 1 | 1 | 1 | 0 | 1 | 0 | 0 | 0 | 0 | 0 | 0 | 0 | 0 | 0 | 0 | 0 | 0 | 0 | 0 | 1 | 0 | 0 | 1 | 0 | 6 | 1 | 2 | 1 | 0 | 3 | 3 | 2 | 1 | 0 | 1 | 1 | 1 | 1 | 6 | 16 | 31 | 1 | 0 | 32 | 2 | 15 |
| LETF | 0 | 1 | 0 | 1 | 1 | 1 | 10 | 1 | 4 | 4 | 4 | 2 | 2 | 1 | 1 | 1 | 1 | 1 | 1 | 1 | 0 | 0 | 0 | 0 | 0 | 0 | 1 | 3 | 1 | 2 | 3 | 1 | 1 | 1 | 5 | 3 | 1 | 3 | 1 | 1 | 1 | 1 | 0 | 1 | 0 | 1 | 1 | 1 | 1 | 1 | 2 | 2 | 0 | 1 | 0 | 0 | 1 | 5 | 1 | 1 | 0 | 9 | 1 | 1 | 2 | 1 | 3 | 2 | 2 | 0 | 2 | 1 | 1 | 1 | 1 | 7 | 43 | 82 | 6 | 0 | 65 | 2 | 43 |
| AJAU | 0 | 1 | 0 | 1 | 1 | 1 | 13 | 1 | 4 | 4 | 3 | 2 | 2 | 1 | 1 | 1 | 1 | 1 | 0 | 1 | 0 | 0 | 0 | 1 | 0 | 0 | 1 | 2 | 1 | 2 | 1 | 1 | 2 | 0 | 7 | 2 | 1 | 4 | 2 | 2 | 2 | 1 | 1 | 1 | 0 | 1 | 1 | 1 | 1 | 1 | 1 | 2 | 1 | 1 | 1 | 1 | 1 | 3 | 1 | 1 | 0 | 9 | 1 | 1 | 1 | 1 | 2 | 2 | 2 | 1 | 2 | 2 | 1 | 0 | 1 | 13 | 50 | 90 | 7 | 0 | 65 | 3 | 45 |
| JIWJ | 0 | 1 | 1 | 1 | 1 | 1 | 8 | 1 | 5 | 5 | 4 | 2 | 1 | 1 | 1 | 1 | 1 | 1 | 1 | 1 | 1 | 0 | 0 | 1 | 0 | 0 | 2 | 4 | 2 | 1 | 2 | 1 | 1 | 2 | 3 | 5 | 1 | 5 | 1 | 2 | 2 | 1 | 0 | 1 | 0 | 1 | 1 | 1 | 1 | 1 | 0 | 3 | 1 | 0 | 0 | 1 | 1 | 4 | 1 | 1 | 0 | 11 | 1 | 1 | 1 | 0 | 2 | 3 | 2 | 1 | 0 | 2 | 2 | 1 | 1 | 5 | 57 | 100 | 7 | 0 | 54 | 3 | 35 |
| CQQP | 0 | 2 | 1 | 2 | 2 | 0 | 9 | 0 | 8 | 7 | 6 | 4 | 2 | 2 | 2 | 2 | 2 | 1 | 1 | 2 | 2 | 0 | 0 | 2 | 0 | 1 | 1 | 2 | 1 | 3 | 3 | 2 | 5 | 0 | 4 | 6 | 1 | 8 | 2 | 3 | 2 | 2 | 2 | 2 | 0 | 2 | 2 | 2 | 2 | 2 | 1 | 2 | 2 | 2 | 0 | 2 | 2 | 5 | 2 | 1 | 0 | 14 | 2 | 2 | 2 | 2 | 4 | 5 | 4 | 0 | 2 | 2 | 2 | 1 | 2 | 17 | 80 | 143 | 12 | 0 | 86 | 2 | 50 |
| OAEZ | 0 | 1 | 1 | 1 | 1 | 1 | 7 | 1 | 4 | 4 | 3 | 2 | 1 | 1 | 1 | 1 | 1 | 1 | 1 | 1 | 0 | 0 | 0 | 1 | 0 | 0 | 0 | 3 | 0 | 1 | 2 | 1 | 2 | 0 | 9 | 3 | 1 | 5 | 2 | 2 | 1 | 1 | 0 | 1 | 0 | 1 | 0 | 0 | 1 | 0 | 2 | 2 | 1 | 1 | 0 | 1 | 1 | 3 | 1 | 1 | 0 | 9 | 1 | 1 | 1 | 1 | 2 | 1 | 2 | 1 | 0 | 0 | 1 | 0 | 1 | 6 | 44 | 90 | 7 | 0 | 60 | 0 | 38 |
| NQYP | 0 | 1 | 0 | 1 | 1 | 0 | 8 | 1 | 4 | 4 | 4 | 1 | 2 | 1 | 1 | 1 | 1 | 1 | 1 | 1 | 0 | 0 | 0 | 0 | 0 | 0 | 2 | 2 | 1 | 1 | 1 | 1 | 2 | 0 | 4 | 3 | 1 | 2 | 2 | 1 | 1 | 1 | 0 | 1 | 0 | 0 | 1 | 1 | 1 | 1 | 0 | 0 | 0 | 1 | 0 | 0 | 1 | 2 | 1 | 1 | 0 | 7 | 1 | 1 | 1 | 1 | 3 | 1 | 1 | 0 | 1 | 1 | 1 | 1 | 1 | 2 | 37 | 76 | 6 | 0 | 55 | 3 | 29 |
| GJIY | 0 | 1 | 1 | 0 | 1 | 1 | 8 | 1 | 4 | 4 | 4 | 2 | 1 | 1 | 1 | 1 | 1 | 1 | 1 | 1 | 0 | 0 | 0 | 1 | 0 | 0 | 1 | 4 | 1 | 2 | 3 | 1 | 1 | 1 | 5 | 2 | 1 | 4 | 2 | 2 | 2 | 1 | 1 | 1 | 0 | 0 | 1 | 1 | 1 | 1 | 2 | 3 | 1 | 1 | 0 | 1 | 1 | 3 | 1 | 1 | 0 | 8 | 1 | 1 | 1 | 1 | 3 | 2 | 2 | 1 | 0 | 0 | 1 | 1 | 1 | 4 | 43 | 86 | 8 | 0 | 58 | 2 | 38 |
| KADG | 0 | 1 | 1 | 1 | 0 | 1 | 18 | 1 | 4 | 3 | 3 | 3 | 1 | 1 | 1 | 1 | 1 | 1 | 1 | 1 | 0 | 0 | 0 | 1 | 0 | 0 | 1 | 2 | 1 | 2 | 2 | 1 | 2 | 0 | 8 | 2 | 1 | 4 | 1 | 2 | 2 | 1 | 1 | 1 | 0 | 1 | 0 | 0 | 0 | 0 | 0 | 1 | 1 | 1 | 1 | 1 | 2 | 2 | 2 | 1 | 0 | 7 | 0 | 1 | 1 | 1 | 3 | 4 | 4 | 1 | 1 | 1 | 0 | 0 | 1 | 7 | 43 | 82 | 6 | 0 | 66 | 2 | 43 |
| VBLH | 0 | 1 | 1 | 1 | 0 | 1 | 0 | 0 | 3 | 2 | 3 | 1 | 0 | 1 | 1 | 1 | 1 | 1 | 1 | 1 | 0 | 0 | 0 | 1 | 0 | 0 | 0 | 3 | 0 | 1 | 1 | 1 | 2 | 0 | 1 | 3 | 1 | 3 | 1 | 0 | 0 | 1 | 0 | 1 | 0 | 0 | 0 | 0 | 0 | 0 | 0 | 1 | 0 | 0 | 0 | 0 | 1 | 3 | 0 | 1 | 0 | 5 | 1 | 0 | 0 | 0 | 0 | 1 | 3 | 0 | 0 | 0 | 1 | 0 | 1 | 6 | 25 | 55 | 8 | 0 | 27 | 1 | 8 |
| YDCQ | 0 | 1 | 0 | 1 | 0 | 1 | 0 | 0 | 2 | 1 | 3 | 1 | 0 | 1 | 1 | 1 | 2 | 1 | 0 | 1 | 0 | 0 | 0 | 0 | 0 | 0 | 1 | 0 | 1 | 0 | 0 | 0 | 0 | 1 | 1 | 2 | 1 | 1 | 1 | 0 | 3 | 1 | 0 | 1 | 0 | 0 | 0 | 0 | 0 | 0 | 0 | 1 | 1 | 1 | 0 | 1 | 0 | 2 | 0 | 0 | 0 | 6 | 0 | 1 | 1 | 1 | 6 | 0 | 3 | 0 | 0 | 0 | 0 | 0 | 1 | 1 | 23 | 51 | 6 | 1 | 19 | 1 | 4 |
| ZDIZ | 0 | 1 | 1 | 1 | 0 | 1 | 11 | 3 | 4 | 4 | 3 | 2 | 1 | 1 | 1 | 1 | 1 | 1 | 1 | 1 | 0 | 0 | 0 | 0 | 0 | 0 | 1 | 2 | 1 | 1 | 2 | 1 | 1 | 0 | 6 | 4 | 1 | 3 | 1 | 1 | 0 | 1 | 0 | 1 | 0 | 0 | 1 | 1 | 1 | 1 | 1 | 1 | 1 | 0 | 0 | 1 | 1 | 3 | 2 | 1 | 0 | 8 | 1 | 2 | 1 | 0 | 2 | 1 | 1 | 1 | 0 | 1 | 1 | 2 | 1 | 12 | 47 | 86 | 5 | 0 | 54 | 1 | 30 |
| WDWX | 0 | 1 | 1 | 1 | 1 | 1 | 11 | 3 | 4 | 4 | 3 | 2 | 1 | 1 | 1 | 1 | 1 | 1 | 1 | 1 | 0 | 0 | 0 | 1 | 0 | 0 | 1 | 2 | 1 | 1 | 2 | 1 | 1 | 0 | 6 | 4 | 1 | 2 | 1 | 2 | 0 | 1 | 1 | 1 | 0 | 1 | 1 | 1 | 1 | 1 | 1 | 1 | 1 | 1 | 0 | 1 | 1 | 3 | 2 | 1 | 0 | 8 | 1 | 1 | 1 | 1 | 2 | 1 | 1 | 2 | 1 | 1 | 1 | 1 | 1 | 15 | 50 | 93 | 7 | 0 | 53 | 2 | 33 |
| RHVC | 0 | 1 | 1 | 1 | 1 | 0 | 8 | 3 | 4 | 4 | 3 | 2 | 1 | 1 | 1 | 1 | 1 | 1 | 1 | 1 | 0 | 0 | 0 | 0 | 0 | 0 | 1 | 2 | 1 | 1 | 2 | 1 | 1 | 1 | 4 | 2 | 1 | 3 | 1 | 2 | 0 | 1 | 0 | 1 | 0 | 0 | 1 | 1 | 1 | 1 | 1 | 0 | 1 | 1 | 0 | 1 | 1 | 3 | 1 | 1 | 0 | 7 | 1 | 1 | 1 | 1 | 1 | 1 | 1 | 0 | 0 | 1 | 1 | 2 | 1 | 8 | 34 | 75 | 7 | 0 | 50 | 1 | 30 |
| UKUC | 0 | 1 | 0 | 0 | 0 | 0 | 7 | 4 | 4 | 4 | 3 | 2 | 1 | 1 | 1 | 1 | 1 | 1 | 1 | 1 | 0 | 0 | 0 | 0 | 0 | 0 | 3 | 1 | 2 | 1 | 2 | 1 | 1 | 2 | 3 | 4 | 1 | 2 | 1 | 2 | 0 | 1 | 0 | 1 | 0 | 0 | 0 | 0 | 0 | 0 | 0 | 1 | 1 | 1 | 0 | 1 | 1 | 3 | 2 | 1 | 0 | 6 | 1 | 1 | 1 | 1 | 3 | 0 | 1 | 2 | 0 | 1 | 1 | 1 | 1 | 16 | 39 | 82 | 6 | 0 | 41 | 1 | 29 |
| SYJM | 0 | 1 | 1 | 1 | 1 | 0 | 8 | 3 | 4 | 4 | 3 | 2 | 1 | 1 | 1 | 1 | 1 | 1 | 1 | 1 | 0 | 0 | 0 | 1 | 0 | 0 | 1 | 2 | 1 | 2 | 1 | 1 | 2 | 1 | 7 | 3 | 1 | 3 | 1 | 2 | 0 | 1 | 1 | 1 | 0 | 1 | 0 | 0 | 1 | 0 | 1 | 1 | 1 | 0 | 0 | 1 | 1 | 3 | 0 | 1 | 0 | 8 | 1 | 1 | 1 | 0 | 3 | 1 | 1 | 1 | 0 | 1 | 1 | 2 | 1 | 14 | 50 | 90 | 4 | 0 | 56 | 1 | 32 |
| QRTH | 0 | 1 | 1 | 1 | 0 | 0 | 8 | 1 | 3 | 3 | 2 | 1 | 2 | 1 | 1 | 1 | 1 | 1 | 1 | 1 | 0 | 0 | 1 | 1 | 0 | 0 | 0 | 3 | 0 | 1 | 1 | 1 | 2 | 0 | 4 | 3 | 1 | 3 | 1 | 0 | 0 | 1 | 0 | 1 | 0 | 0 | 0 | 0 | 0 | 0 | 0 | 0 | 0 | 0 | 0 | 0 | 0 | 3 | 0 | 1 | 0 | 7 | 1 | 1 | 1 | 0 | 0 | 0 | 0 | 0 | 0 | 1 | 1 | 0 | 1 | 4 | 34 | 72 | 7 | 0 | 40 | 0 | 24 |
| PRIQ | 0 | 1 | 1 | 1 | 1 | 2 | 5 | 1 | 4 | 4 | 3 | 1 | 1 | 1 | 1 | 1 | 1 | 1 | 1 | 1 | 0 | 0 | 1 | 1 | 0 | 0 | 1 | 1 | 1 | 1 | 0 | 1 | 1 | 0 | 5 | 3 | 1 | 5 | 1 | 1 | 2 | 1 | 0 | 1 | 0 | 1 | 0 | 0 | 0 | 0 | 0 | 0 | 1 | 1 | 0 | 1 | 1 | 3 | 0 | 1 | 0 | 6 | 1 | 1 | 1 | 0 | 0 | 1 | 2 | 0 | 0 | 1 | 1 | 0 | 1 | 2 | 46 | 88 | 6 | 0 | 53 | 1 | 27 |
| ZLQE | 0 | 1 | 1 | 1 | 0 | 1 | 16 | 1 | 4 | 4 | 3 | 2 | 1 | 1 | 1 | 1 | 1 | 1 | 1 | 1 | 0 | 0 | 1 | 1 | 0 | 0 | 0 | 1 | 0 | 1 | 1 | 1 | 2 | 0 | 3 | 2 | 1 | 3 | 1 | 1 | 0 | 1 | 0 | 1 | 0 | 0 | 0 | 0 | 0 | 0 | 2 | 2 | 0 | 1 | 0 | 0 | 1 | 2 | 0 | 1 | 0 | 7 | 1 | 0 | 1 | 1 | 2 | 1 | 2 | 0 | 0 | 1 | 1 | 0 | 0 | 6 | 34 | 71 | 1 | 0 | 57 | 2 | 31 |
| GUBD | 0 | 1 | 1 | 1 | 1 | 1 | 9 | 2 | 4 | 4 | 3 | 2 | 1 | 1 | 1 | 1 | 1 | 1 | 1 | 1 | 0 | 0 | 1 | 1 | 0 | 0 | 0 | 1 | 0 | 1 | 1 | 1 | 1 | 0 | 5 | 3 | 1 | 4 | 1 | 2 | 2 | 1 | 0 | 1 | 0 | 0 | 0 | 0 | 1 | 0 | 1 | 1 | 1 | 1 | 0 | 1 | 1 | 3 | 1 | 1 | 0 | 7 | 1 | 1 | 1 | 1 | 2 | 2 | 2 | 0 | 1 | 2 | 1 | 1 | 1 | 13 | 47 | 87 | 6 | 0 | 55 | 2 | 31 |
| MULF | 0 | 1 | 1 | 1 | 0 | 0 | 8 | 3 | 4 | 4 | 3 | 1 | 2 | 1 | 1 | 1 | 1 | 1 | 0 | 1 | 0 | 0 | 0 | 0 | 0 | 0 | 0 | 0 | 0 | 1 | 1 | 1 | 1 | 0 | 5 | 1 | 0 | 3 | 2 | 2 | 2 | 1 | 0 | 2 | 0 | 0 | 0 | 0 | 0 | 0 | 0 | 0 | 0 | 0 | 0 | 0 | 1 | 2 | 1 | 1 | 0 | 4 | 1 | 0 | 0 | 0 | 0 | 3 | 3 | 0 | 0 | 1 | 1 | 1 | 1 | 3 | 27 | 53 | 2 | 0 | 32 | 0 | 20 |
| VALZ | 0 | 1 | 1 | 1 | 0 | 1 | 4 | 3 | 3 | 2 | 2 | 1 | 1 | 2 | 1 | 1 | 2 | 0 | 1 | 2 | 0 | 0 | 0 | 1 | 0 | 0 | 0 | 4 | 0 | 1 | 2 | 2 | 1 | 0 | 2 | 2 | 1 | 3 | 2 | 1 | 0 | 0 | 0 | 2 | 0 | 0 | 0 | 0 | 0 | 0 | 0 | 0 | 0 | 1 | 0 | 0 | 0 | 2 | 0 | 1 | 0 | 6 | 1 | 2 | 1 | 1 | 0 | 2 | 4 | 0 | 0 | 1 | 0 | 0 | 0 | 7 | 41 | 56 | 0 | 0 | 39 | 0 | 17 |
| JRGZ | 0 | 1 | 1 | 1 | 0 | 0 | 8 | 2 | 3 | 3 | 3 | 1 | 1 | 1 | 1 | 1 | 0 | 1 | 1 | 2 | 0 | 0 | 0 | 0 | 0 | 0 | 0 | 1 | 0 | 1 | 1 | 1 | 2 | 0 | 1 | 2 | 1 | 1 | 1 | 1 | 1 | 1 | 0 | 2 | 0 | 0 | 0 | 0 | 0 | 0 | 0 | 1 | 1 | 0 | 0 | 1 | 0 | 2 | 0 | 1 | 0 | 6 | 1 | 2 | 0 | 0 | 0 | 3 | 3 | 0 | 0 | 0 | 0 | 0 | 0 | 1 | 29 | 55 | 0 | 0 | 32 | 0 | 17 |
| GFUR | 0 | 1 | 1 | 1 | 0 | 1 | 4 | 2 | 4 | 4 | 3 | 1 | 1 | 1 | 1 | 1 | 1 | 1 | 1 | 1 | 0 | 0 | 0 | 1 | 0 | 0 | 0 | 0 | 0 | 1 | 0 | 1 | 1 | 0 | 3 | 1 | 1 | 3 | 1 | 1 | 1 | 1 | 1 | 2 | 0 | 0 | 0 | 0 | 1 | 0 | 0 | 1 | 1 | 0 | 0 | 1 | 1 | 1 | 0 | 1 | 0 | 5 | 1 | 1 | 0 | 0 | 0 | 3 | 3 | 1 | 0 | 2 | 1 | 1 | 1 | 1 | 33 | 75 | 8 | 0 | 42 | 2 | 21 |
| TSBQ | 0 | 1 | 1 | 1 | 0 | 0 | 5 | 2 | 3 | 3 | 2 | 0 | 1 | 1 | 1 | 1 | 1 | 1 | 1 | 1 | 0 | 0 | 0 | 0 | 0 | 0 | 0 | 0 | 0 | 1 | 1 | 1 | 1 | 0 | 7 | 1 | 1 | 3 | 1 | 1 | 0 | 1 | 0 | 1 | 0 | 0 | 0 | 0 | 0 | 0 | 0 | 0 | 0 | 0 | 0 | 0 | 1 | 3 | 0 | 1 | 0 | 2 | 1 | 1 | 0 | 0 | 2 | 3 | 2 | 0 | 0 | 0 | 0 | 0 | 0 | 5 | 27 | 59 | 0 | 0 | 52 | 0 | 28 |
| ACRY | 0 | 1 | 1 | 1 | 0 | 0 | 5 | 2 | 4 | 4 | 3 | 1 | 1 | 1 | 1 | 1 | 1 | 1 | 1 | 1 | 0 | 0 | 0 | 1 | 0 | 0 | 0 | 1 | 0 | 1 | 1 | 1 | 1 | 0 | 3 | 2 | 0 | 3 | 1 | 0 | 1 | 1 | 0 | 2 | 0 | 0 | 0 | 0 | 0 | 0 | 0 | 0 | 0 | 0 | 0 | 0 | 0 | 2 | 0 | 0 | 0 | 3 | 0 | 1 | 1 | 0 | 0 | 1 | 1 | 0 | 0 | 0 | 1 | 0 | 1 | 2 | 13 | 29 | 6 | 0 | 26 | 2 | 20 |
| LNIL | 0 | 1 | 1 | 1 | 0 | 0 | 4 | 2 | 4 | 4 | 3 | 1 | 1 | 1 | 1 | 1 | 1 | 1 | 1 | 1 | 0 | 0 | 0 | 1 | 0 | 0 | 0 | 1 | 0 | 1 | 1 | 1 | 1 | 0 | 6 | 3 | 0 | 4 | 1 | 0 | 2 | 1 | 0 | 2 | 0 | 0 | 0 | 0 | 0 | 0 | 0 | 0 | 0 | 1 | 0 | 0 | 1 | 2 | 0 | 1 | 0 | 3 | 0 | 0 | 0 | 1 | 0 | 1 | 0 | 0 | 0 | 1 | 0 | 0 | 1 | 10 | 18 | 39 | 1 | 0 | 38 | 0 | 28 |
| ZIVZ | 0 | 1 | 1 | 1 | 0 | 1 | 7 | 1 | 2 | 2 | 2 | 1 | 1 | 1 | 1 | 1 | 1 | 0 | 1 | 1 | 0 | 0 | 0 | 1 | 0 | 0 | 0 | 1 | 0 | 1 | 1 | 1 | 2 | 0 | 3 | 3 | 1 | 3 | 1 | 1 | 0 | 1 | 0 | 2 | 0 | 0 | 0 | 0 | 0 | 0 | 2 | 0 | 0 | 0 | 0 | 0 | 0 | 2 | 0 | 1 | 0 | 5 | 1 | 1 | 0 | 0 | 0 | 2 | 1 | 0 | 1 | 2 | 0 | 1 | 1 | 9 | 24 | 57 | 7 | 0 | 37 | 1 | 22 |
| WRSL | 0 | 1 | 1 | 1 | 0 | 1 | 8 | 2 | 4 | 4 | 3 | 2 | 1 | 1 | 1 | 1 | 1 | 1 | 1 | 1 | 0 | 0 | 0 | 1 | 0 | 0 | 0 | 4 | 0 | 1 | 2 | 1 | 2 | 0 | 6 | 3 | 1 | 2 | 1 | 1 | 0 | 1 | 0 | 2 | 0 | 0 | 0 | 0 | 0 | 0 | 0 | 0 | 1 | 2 | 0 | 1 | 1 | 2 | 0 | 1 | 0 | 8 | 1 | 1 | 1 | 0 | 1 | 1 | 1 | 0 | 0 | 1 | 1 | 2 | 2 | 16 | 44 | 75 | 7 | 0 | 53 | 1 | 31 |
| JWGT | 0 | 1 | 1 | 1 | 0 | 1 | 9 | 0 | 4 | 4 | 3 | 2 | 1 | 1 | 1 | 1 | 1 | 1 | 1 | 1 | 0 | 0 | 0 | 0 | 0 | 0 | 0 | 3 | 0 | 1 | 2 | 1 | 1 | 0 | 5 | 3 | 1 | 2 | 1 | 1 | 2 | 1 | 0 | 2 | 0 | 1 | 0 | 0 | 0 | 0 | 0 | 1 | 0 | 1 | 0 | 0 | 1 | 2 | 0 | 1 | 0 | 7 | 1 | 1 | 1 | 1 | 0 | 2 | 2 | 0 | 0 | 2 | 1 | 1 | 1 | 20 | 36 | 66 | 5 | 0 | 48 | 4 | 25 |
| RYJX | 0 | 1 | 1 | 1 | 0 | 0 | 6 | 1 | 4 | 4 | 3 | 2 | 1 | 1 | 1 | 1 | 1 | 1 | 1 | 1 | 0 | 0 | 0 | 0 | 0 | 0 | 0 | 2 | 0 | 1 | 1 | 1 | 1 | 0 | 3 | 3 | 1 | 3 | 1 | 1 | 0 | 1 | 0 | 2 | 0 | 0 | 0 | 0 | 1 | 0 | 0 | 1 | 0 | 1 | 0 | 0 | 1 | 2 | 0 | 1 | 0 | 6 | 1 | 1 | 1 | 1 | 2 | 1 | 1 | 0 | 0 | 2 | 1 | 1 | 0 | 24 | 33 | 73 | 4 | 0 | 50 | 0 | 25 |
| RNAT | 0 | 1 | 1 | 1 | 0 | 0 | 5 | 3 | 4 | 4 | 3 | 2 | 0 | 1 | 1 | 1 | 1 | 1 | 1 | 1 | 0 | 0 | 0 | 1 | 0 | 0 | 0 | 1 | 0 | 1 | 1 | 1 | 1 | 0 | 5 | 3 | 1 | 2 | 1 | 1 | 0 | 1 | 0 | 1 | 0 | 0 | 1 | 1 | 1 | 1 | 0 | 1 | 1 | 1 | 1 | 1 | 1 | 3 | 0 | 1 | 0 | 7 | 1 | 1 | 1 | 1 | 2 | 1 | 1 | 1 | 0 | 2 | 1 | 0 | 1 | 10 | 36 | 76 | 6 | 0 | 51 | 1 | 23 |
| ISPU | 0 | 1 | 1 | 1 | 0 | 0 | 10 | 1 | 4 | 4 | 3 | 1 | 1 | 1 | 1 | 1 | 1 | 1 | 1 | 1 | 0 | 0 | 0 | 0 | 0 | 0 | 0 | 4 | 0 | 1 | 2 | 1 | 2 | 0 | 5 | 3 | 1 | 3 | 1 | 1 | 3 | 1 | 0 | 1 | 0 | 0 | 0 | 0 | 0 | 0 | 1 | 1 | 1 | 1 | 1 | 1 | 1 | 2 | 0 | 1 | 0 | 6 | 1 | 1 | 1 | 0 | 0 | 1 | 1 | 0 | 0 | 1 | 0 | 1 | 2 | 15 | 30 | 57 | 6 | 0 | 49 | 2 | 26 |
| IHOI | 0 | 1 | 1 | 1 | 0 | 0 | 1 | 1 | 3 | 3 | 2 | 1 | 0 | 1 | 1 | 1 | 1 | 1 | 1 | 1 | 1 | 0 | 0 | 0 | 0 | 0 | 0 | 1 | 0 | 1 | 1 | 1 | 2 | 0 | 3 | 1 | 0 | 2 | 1 | 1 | 2 | 1 | 0 | 1 | 0 | 0 | 0 | 0 | 0 | 0 | 0 | 1 | 0 | 0 | 0 | 0 | 0 | 1 | 0 | 1 | 0 | 2 | 1 | 0 | 0 | 0 | 0 | 0 | 1 | 0 | 0 | 0 | 0 | 0 | 0 | 2 | 17 | 34 | 2 | 0 | 18 | 0 | 8 |
| BCYF | 0 | 1 | 1 | 0 | 0 | 1 | 0 | 0 | 2 | 2 | 2 | 1 | 0 | 1 | 1 | 1 | 1 | 1 | 1 | 1 | 0 | 0 | 0 | 0 | 0 | 0 | 0 | 0 | 0 | 0 | 0 | 0 | 0 | 0 | 3 | 4 | 0 | 2 | 1 | 0 | 2 | 1 | 0 | 1 | 0 | 0 | 0 | 0 | 0 | 0 | 0 | 0 | 0 | 0 | 0 | 0 | 0 | 1 | 0 | 1 | 0 | 2 | 1 | 2 | 1 | 0 | 0 | 2 | 0 | 0 | 0 | 0 | 0 | 0 | 1 | 3 | 11 | 26 | 1 | 0 | 21 | 0 | 11 |
| JKKI | 0 | 1 | 0 | 1 | 0 | 0 | 6 | 1 | 4 | 4 | 3 | 1 | 1 | 1 | 1 | 1 | 1 | 1 | 1 | 1 | 0 | 0 | 0 | 1 | 0 | 0 | 0 | 1 | 0 | 1 | 1 | 1 | 1 | 0 | 5 | 3 | 1 | 3 | 1 | 1 | 2 | 1 | 0 | 2 | 0 | 0 | 0 | 0 | 0 | 0 | 0 | 0 | 0 | 2 | 1 | 0 | 1 | 1 | 0 | 1 | 0 | 7 | 1 | 1 | 1 | 0 | 0 | 1 | 1 | 0 | 0 | 1 | 0 | 0 | 1 | 10 | 31 | 60 | 6 | 0 | 53 | 1 | 19 |
| USIX | 0 | 0 | 1 | 1 | 0 | 1 | 2 | 1 | 3 | 2 | 2 | 1 | 0 | 1 | 1 | 1 | 1 | 0 | 0 | 1 | 0 | 0 | 0 | 1 | 0 | 0 | 0 | 1 | 0 | 1 | 0 | 1 | 1 | 0 | 0 | 1 | 1 | 2 | 1 | 0 | 0 | 1 | 0 | 0 | 0 | 0 | 0 | 0 | 1 | 0 | 1 | 0 | 0 | 0 | 0 | 0 | 0 | 1 | 0 | 0 | 0 | 2 | 0 | 1 | 0 | 0 | 1 | 2 | 2 | 0 | 0 | 0 | 0 | 1 | 1 | 0 | 12 | 40 | 3 | 0 | 19 | 0 | 10 |
| QWRA | 0 | 1 | 1 | 1 | 0 | 1 | 7 | 2 | 4 | 4 | 3 | 2 | 1 | 1 | 1 | 1 | 1 | 1 | 1 | 1 | 0 | 0 | 0 | 1 | 0 | 0 | 0 | 1 | 0 | 1 | 1 | 1 | 2 | 0 | 2 | 3 | 1 | 3 | 1 | 0 | 0 | 1 | 0 | 1 | 0 | 0 | 0 | 0 | 0 | 0 | 1 | 1 | 0 | 0 | 0 | 0 | 0 | 2 | 0 | 1 | 0 | 8 | 0 | 0 | 0 | 0 | 1 | 5 | 5 | 1 | 1 | 1 | 0 | 0 | 1 | 0 | 27 | 57 | 2 | 0 | 34 | 0 | 18 |
| IRYH | 0 | 1 | 1 | 1 | 1 | 1 | 14 | 0 | 4 | 4 | 3 | 2 | 1 | 1 | 1 | 1 | 1 | 1 | 1 | 1 | 0 | 0 | 0 | 0 | 0 | 0 | 2 | 2 | 1 | 1 | 2 | 1 | 2 | 1 | 7 | 3 | 1 | 3 | 1 | 2 | 3 | 1 | 0 | 1 | 0 | 0 | 1 | 1 | 1 | 1 | 0 | 2 | 1 | 0 | 0 | 1 | 1 | 3 | 1 | 1 | 0 | 6 | 1 | 1 | 0 | 0 | 1 | 3 | 3 | 1 | 0 | 1 | 0 | 0 | 2 | 2 | 48 | 84 | 6 | 0 | 60 | 2 | 39 |
| OFUE | 0 | 1 | 0 | 1 | 0 | 1 | 7 | 1 | 2 | 2 | 1 | 1 | 2 | 1 | 1 | 1 | 1 | 0 | 0 | 1 | 0 | 0 | 0 | 1 | 0 | 0 | 0 | 1 | 0 | 2 | 1 | 1 | 1 | 0 | 2 | 3 | 1 | 2 | 1 | 1 | 2 | 1 | 0 | 2 | 0 | 0 | 1 | 1 | 1 | 1 | 0 | 1 | 0 | 0 | 0 | 0 | 0 | 2 | 0 | 1 | 0 | 4 | 1 | 1 | 0 | 0 | 1 | 4 | 4 | 0 | 1 | 0 | 0 | 0 | 2 | 8 | 33 | 65 | 0 | 0 | 33 | 0 | 16 |
| XDLL | 0 | 1 | 1 | 1 | 0 | 0 | 7 | 0 | 1 | 1 | 0 | 1 | 1 | 1 | 1 | 1 | 1 | 0 | 0 | 1 | 0 | 0 | 0 | 1 | 0 | 0 | 0 | 1 | 0 | 2 | 1 | 1 | 1 | 0 | 3 | 4 | 1 | 2 | 2 | 1 | 0 | 1 | 0 | 2 | 0 | 0 | 1 | 1 | 1 | 1 | 0 | 1 | 0 | 0 | 0 | 0 | 1 | 3 | 0 | 1 | 0 | 3 | 1 | 1 | 0 | 0 | 1 | 4 | 4 | 0 | 2 | 0 | 0 | 0 | 2 | 9 | 39 | 71 | 0 | 0 | 44 | 0 | 23 |
| AJUW | 0 | 2 | 1 | 1 | 1 | 1 | 5 | 2 | 4 | 4 | 3 | 1 | 2 | 1 | 2 | 1 | 1 | 1 | 1 | 1 | 0 | 0 | 0 | 1 | 0 | 0 | 0 | 2 | 0 | 2 | 1 | 1 | 2 | 0 | 4 | 2 | 1 | 3 | 1 | 1 | 2 | 1 | 0 | 1 | 0 | 0 | 0 | 0 | 1 | 0 | 0 | 3 | 1 | 0 | 0 | 1 | 1 | 2 | 1 | 1 | 0 | 7 | 1 | 1 | 1 | 0 | 3 | 4 | 3 | 0 | 0 | 1 | 1 | 1 | 1 | 3 | 45 | 75 | 6 | 0 | 57 | 2 | 29 |
| LBRP | 0 | 1 | 2 | 1 | 1 | 0 | 3 | 1 | 4 | 4 | 4 | 2 | 1 | 1 | 1 | 1 | 1 | 1 | 0 | 1 | 0 | 0 | 0 | 0 | 0 | 0 | 2 | 0 | 0 | 0 | 1 | 1 | 1 | 0 | 2 | 1 | 0 | 3 | 1 | 1 | 2 | 1 | 0 | 1 | 0 | 0 | 1 | 1 | 1 | 1 | 0 | 0 | 0 | 0 | 0 | 0 | 1 | 2 | 0 | 0 | 0 | 4 | 1 | 1 | 1 | 0 | 1 | 3 | 3 | 0 | 1 | 2 | 0 | 1 | 0 | 4 | 36 | 55 | 0 | 0 | 36 | 0 | 14 |
| AOUJ | 0 | 1 | 2 | 1 | 1 | 1 | 7 | 4 | 3 | 3 | 2 | 1 | 1 | 1 | 1 | 1 | 2 | 1 | 1 | 1 | 0 | 0 | 1 | 1 | 0 | 0 | 0 | 2 | 0 | 1 | 1 | 1 | 1 | 0 | 6 | 1 | 1 | 4 | 1 | 0 | 2 | 2 | 1 | 1 | 0 | 1 | 0 | 0 | 1 | 0 | 1 | 1 | 0 | 1 | 0 | 0 | 1 | 3 | 0 | 1 | 0 | 8 | 1 | 1 | 1 | 1 | 1 | 4 | 5 | 0 | 1 | 1 | 1 | 0 | 1 | 2 | 40 | 62 | 8 | 0 | 43 | 2 | 22 |
| UTRE | 0 | 1 | 0 | 0 | 0 | 0 | 5 | 0 | 1 | 1 | 1 | 0 | 0 | 1 | 0 | 0 | 0 | 1 | 1 | 1 | 0 | 0 | 0 | 0 | 0 | 0 | 0 | 1 | 0 | 0 | 1 | 0 | 2 | 0 | 1 | 0 | 0 | 0 | 0 | 0 | 0 | 0 | 0 | 0 | 0 | 0 | 0 | 0 | 0 | 0 | 0 | 0 | 0 | 0 | 0 | 0 | 0 | 0 | 0 | 0 | 0 | 1 | 0 | 0 | 0 | 0 | 0 | 1 | 0 | 0 | 0 | 0 | 0 | 0 | 0 | 0 | 9 | 18 | 1 | 0 | 10 | 0 | 4 |
| FXHG | 0 | 1 | 2 | 1 | 0 | 1 | 11 | 2 | 4 | 4 | 3 | 2 | 1 | 1 | 1 | 1 | 1 | 1 | 1 | 1 | 0 | 0 | 0 | 0 | 0 | 0 | 0 | 1 | 0 | 1 | 1 | 1 | 1 | 0 | 6 | 2 | 1 | 3 | 1 | 1 | 3 | 1 | 1 | 1 | 0 | 1 | 0 | 0 | 0 | 0 | 1 | 3 | 1 | 0 | 1 | 1 | 1 | 3 | 2 | 1 | 0 | 9 | 1 | 0 | 1 | 0 | 4 | 3 | 3 | 3 | 1 | 2 | 1 | 1 | 1 | 11 | 42 | 83 | 5 | 0 | 59 | 2 | 36 |
| RUIF | 0 | 1 | 1 | 1 | 1 | 1 | 6 | 3 | 4 | 4 | 3 | 2 | 1 | 1 | 1 | 1 | 1 | 1 | 1 | 1 | 0 | 0 | 0 | 1 | 0 | 0 | 0 | 1 | 0 | 1 | 1 | 1 | 1 | 0 | 5 | 1 | 1 | 4 | 0 | 1 | 0 | 1 | 0 | 1 | 0 | 0 | 0 | 0 | 0 | 0 | 1 | 1 | 0 | 0 | 0 | 0 | 1 | 2 | 0 | 1 | 0 | 4 | 1 | 1 | 0 | 0 | 0 | 5 | 4 | 0 | 1 | 1 | 0 | 0 | 1 | 13 | 29 | 61 | 0 | 0 | 37 | 0 | 27 |
| VIAU | 0 | 1 | 1 | 1 | 0 | 1 | 10 | 4 | 4 | 4 | 3 | 2 | 1 | 1 | 1 | 1 | 1 | 1 | 1 | 1 | 0 | 0 | 0 | 1 | 0 | 0 | 0 | 1 | 0 | 2 | 1 | 1 | 2 | 0 | 6 | 2 | 1 | 2 | 1 | 1 | 0 | 1 | 1 | 2 | 0 | 0 | 0 | 0 | 1 | 0 | 0 | 0 | 0 | 0 | 0 | 0 | 1 | 4 | 0 | 1 | 0 | 6 | 1 | 1 | 1 | 0 | 3 | 3 | 2 | 0 | 0 | 1 | 1 | 0 | 1 | 3 | 26 | 72 | 6 | 0 | 59 | 0 | 31 |
| XTON | 0 | 1 | 1 | 0 | 1 | 2 | 6 | 3 | 4 | 4 | 3 | 1 | 1 | 1 | 1 | 1 | 1 | 1 | 1 | 1 | 0 | 0 | 0 | 0 | 0 | 0 | 2 | 3 | 1 | 2 | 1 | 1 | 2 | 1 | 4 | 3 | 1 | 4 | 1 | 1 | 0 | 1 | 0 | 1 | 0 | 0 | 0 | 0 | 1 | 0 | 0 | 0 | 0 | 0 | 0 | 0 | 0 | 2 | 2 | 1 | 0 | 4 | 1 | 0 | 1 | 0 | 0 | 3 | 2 | 0 | 0 | 1 | 0 | 0 | 1 | 21 | 22 | 51 | 0 | 0 | 37 | 0 | 19 |
| XKWQ | 0 | 1 | 1 | 0 | 0 | 1 | 4 | 1 | 2 | 2 | 1 | 1 | 0 | 1 | 1 | 1 | 1 | 2 | 1 | 1 | 0 | 0 | 0 | 1 | 0 | 0 | 0 | 1 | 0 | 2 | 1 | 1 | 1 | 0 | 5 | 2 | 1 | 2 | 1 | 0 | 0 | 1 | 0 | 2 | 0 | 0 | 0 | 0 | 1 | 0 | 0 | 1 | 0 | 0 | 0 | 0 | 0 | 1 | 2 | 0 | 0 | 7 | 0 | 0 | 0 | 0 | 1 | 2 | 1 | 0 | 0 | 1 | 0 | 0 | 1 | 8 | 11 | 34 | 6 | 0 | 29 | 0 | 16 |
| VJDZ | 0 | 1 | 2 | 1 | 1 | 2 | 28 | 4 | 4 | 4 | 3 | 2 | 1 | 1 | 1 | 1 | 1 | 1 | 1 | 1 | 0 | 0 | 1 | 2 | 0 | 0 | 1 | 2 | 1 | 2 | 2 | 1 | 1 | 1 | 6 | 3 | 1 | 4 | 1 | 2 | 0 | 1 | 1 | 2 | 0 | 1 | 1 | 1 | 1 | 1 | 2 | 3 | 1 | 1 | 0 | 1 | 1 | 3 | 2 | 1 | 0 | 9 | 1 | 1 | 1 | 0 | 2 | 1 | 2 | 2 | 0 | 2 | 1 | 2 | 1 | 22 | 50 | 99 | 8 | 0 | 78 | 3 | 41 |
| PZIF | 0 | 1 | 2 | 1 | 0 | 1 | 24 | 4 | 4 | 4 | 3 | 1 | 1 | 1 | 1 | 1 | 1 | 1 | 1 | 1 | 0 | 0 | 1 | 1 | 0 | 0 | 1 | 2 | 1 | 2 | 2 | 1 | 1 | 1 | 10 | 4 | 1 | 4 | 1 | 2 | 3 | 1 | 0 | 2 | 0 | 0 | 1 | 1 | 1 | 1 | 4 | 1 | 1 | 1 | 0 | 1 | 1 | 4 | 1 | 1 | 0 | 8 | 1 | 1 | 1 | 1 | 5 | 4 | 2 | 0 | 0 | 1 | 0 | 0 | 1 | 12 | 39 | 83 | 4 | 0 | 63 | 1 | 36 |
| MWAN | 0 | 1 | 1 | 0 | 0 | 2 | 5 | 4 | 3 | 3 | 2 | 1 | 1 | 1 | 1 | 1 | 1 | 0 | 1 | 1 | 0 | 0 | 0 | 0 | 0 | 0 | 0 | 1 | 0 | 1 | 1 | 1 | 1 | 0 | 3 | 2 | 1 | 1 | 1 | 0 | 1 | 1 | 0 | 1 | 0 | 0 | 0 | 0 | 0 | 0 | 0 | 0 | 0 | 1 | 0 | 0 | 1 | 2 | 1 | 1 | 0 | 5 | 0 | 1 | 0 | 0 | 0 | 0 | 0 | 0 | 0 | 1 | 0 | 0 | 1 | 0 | 20 | 50 | 3 | 0 | 31 | 2 | 12 |
| OTQG | 0 | 1 | 1 | 0 | 0 | 2 | 3 | 1 | 3 | 2 | 2 | 1 | 0 | 1 | 1 | 1 | 1 | 0 | 0 | 1 | 0 | 0 | 0 | 0 | 0 | 0 | 0 | 2 | 0 | 1 | 1 | 1 | 2 | 0 | 5 | 2 | 1 | 1 | 1 | 0 | 2 | 1 | 0 | 2 | 0 | 0 | 0 | 0 | 0 | 0 | 0 | 0 | 1 | 1 | 0 | 1 | 0 | 1 | 1 | 1 | 0 | 4 | 0 | 1 | 1 | 1 | 0 | 0 | 0 | 0 | 0 | 1 | 1 | 1 | 1 | 0 | 20 | 55 | 5 | 0 | 28 | 1 | 18 |
| BZSH | 0 | 1 | 2 | 1 | 1 | 1 | 10 | 4 | 4 | 3 | 3 | 2 | 3 | 1 | 1 | 1 | 1 | 1 | 1 | 1 | 0 | 0 | 1 | 1 | 0 | 0 | 1 | 2 | 1 | 2 | 1 | 1 | 1 | 0 | 7 | 3 | 1 | 4 | 1 | 2 | 2 | 1 | 1 | 2 | 0 | 1 | 0 | 0 | 0 | 0 | 2 | 1 | 1 | 1 | 1 | 1 | 1 | 4 | 2 | 1 | 0 | 8 | 1 | 1 | 1 | 1 | 6 | 5 | 5 | 2 | 2 | 1 | 0 | 1 | 1 | 1 | 41 | 84 | 7 | 0 | 78 | 3 | 46 |
| ENAU | 0 | 0 | 0 | 0 | 0 | 1 | 1 | 0 | 4 | 3 | 2 | 1 | 1 | 1 | 1 | 1 | 1 | 1 | 1 | 1 | 0 | 0 | 0 | 0 | 0 | 0 | 3 | 2 | 0 | 1 | 1 | 1 | 2 | 1 | 2 | 2 | 1 | 3 | 1 | 0 | 2 | 1 | 0 | 1 | 0 | 0 | 0 | 0 | 0 | 0 | 0 | 0 | 0 | 0 | 0 | 0 | 0 | 1 | 0 | 1 | 0 | 3 | 1 | 0 | 0 | 0 | 1 | 0 | 1 | 0 | 0 | 1 | 0 | 1 | 1 | 1 | 16 | 47 | 4 | 0 | 13 | 0 | 8 |
| MXDS | 0 | 0 | 1 | 1 | 0 | 1 | 3 | 0 | 4 | 4 | 3 | 1 | 1 | 1 | 1 | 1 | 1 | 1 | 1 | 1 | 0 | 0 | 0 | 0 | 0 | 0 | 0 | 2 | 0 | 3 | 1 | 1 | 1 | 0 | 3 | 4 | 1 | 4 | 1 | 1 | 3 | 1 | 0 | 1 | 0 | 0 | 1 | 1 | 1 | 1 | 0 | 0 | 0 | 0 | 0 | 0 | 1 | 2 | 0 | 1 | 0 | 6 | 1 | 1 | 1 | 0 | 3 | 0 | 1 | 0 | 0 | 1 | 1 | 1 | 1 | 6 | 38 | 75 | 3 | 0 | 45 | 4 | 25 |
| DZPJ | 0 | 1 | 2 | 0 | 1 | 0 | 11 | 1 | 4 | 4 | 3 | 1 | 3 | 1 | 1 | 1 | 1 | 1 | 0 | 1 | 0 | 0 | 0 | 0 | 0 | 0 | 0 | 1 | 0 | 2 | 2 | 1 | 1 | 1 | 6 | 3 | 1 | 3 | 1 | 1 | 2 | 1 | 0 | 2 | 0 | 0 | 1 | 1 | 1 | 1 | 1 | 1 | 1 | 1 | 0 | 1 | 1 | 1 | 1 | 1 | 0 | 7 | 1 | 1 | 0 | 1 | 5 | 4 | 3 | 0 | 2 | 2 | 1 | 0 | 1 | 4 | 39 | 79 | 5 | 0 | 58 | 0 | 36 |
| JMUI | 0 | 1 | 1 | 0 | 1 | 1 | 3 | 1 | 4 | 4 | 3 | 1 | 1 | 1 | 1 | 1 | 1 | 1 | 1 | 1 | 0 | 0 | 0 | 0 | 0 | 0 | 0 | 1 | 0 | 2 | 1 | 1 | 1 | 0 | 2 | 2 | 0 | 1 | 1 | 0 | 1 | 1 | 0 | 1 | 0 | 0 | 1 | 1 | 1 | 1 | 0 | 1 | 0 | 2 | 0 | 0 | 0 | 0 | 1 | 1 | 0 | 5 | 1 | 1 | 0 | 1 | 0 | 5 | 4 | 0 | 1 | 0 | 0 | 1 | 1 | 10 | 26 | 43 | 2 | 0 | 31 | 0 | 16 |
| VFIV | 0 | 1 | 2 | 1 | 0 | 1 | 7 | 0 | 4 | 4 | 3 | 1 | 1 | 1 | 1 | 1 | 1 | 1 | 1 | 0 | 0 | 0 | 0 | 1 | 0 | 0 | 0 | 0 | 0 | 2 | 1 | 1 | 1 | 0 | 2 | 2 | 1 | 3 | 1 | 2 | 0 | 1 | 0 | 1 | 0 | 0 | 0 | 0 | 0 | 0 | 0 | 2 | 2 | 1 | 1 | 0 | 1 | 4 | 2 | 1 | 0 | 9 | 1 | 1 | 1 | 3 | 3 | 4 | 2 | 0 | 1 | 1 | 2 | 1 | 12 | 33 | 77 | 5 | 0 | 44 | 3 | 23 |

# Supplementary Table S3 (pg 3/4, lower left quadrant)

| sample code | ec2.7.1.19 | ec4.1.1.39 | ec4.1.2.13 | ec5.1.3.1 | ec5.3.1.6 | PF00016 | PF00101 | PF00194 | PF00484 | PF02788 | Rubisco_activase | ec1.1.1.157 | ec1.1.1.30 | ec1.1.1.37 | ec1.1.1.38 | ec1.1.1.40 | ec1.1.5.3 | ec1.2.1.12 | ec1.2.1.13 | ec1.2.1.9 | ec2.3.1.9 | ec2.3.3.1 | ec2.3.3.9 | ec2.7.2.3 | ec2.7.9.1 | ec3.1.3.11 | ec3.1.3.37 | ec4.1.1.3 | ec4.1.1.32 | ec4.1.1.49 |
|---|---|---|---|---|---|---|---|---|---|---|---|---|---|---|---|---|---|---|---|---|---|---|---|---|---|---|---|---|---|---|
| ISGT | 1 | 3 | 1 | 1 | 1 | 1 | 5 | 1 | 0 | 1 | 0 | 2 | 12 | 6 | 1 | 1 | 1 | 5 | 2 | 3 | 2 | 2 | 1 | 1 | 1 | 5 | 4 | 0 | 0 | 0 |
| RAWF | 1 | 2 | 3 | 2 | 2 | 1 | 2 | 1 | 2 | 1 | 0 | 3 | 15 | 7 | 3 | 3 | 1 | 2 | 2 | 6 | 2 | 3 | 1 | 2 | 2 | 4 | 3 | 1 | 0 | 1 |
| IJMT | 1 | 1 | 3 | 4 | 3 | 0 | 1 | 2 | 9 | 0 | 0 | 6 | 38 | 9 | 4 | 4 | 4 | 5 | 3 | 20 | 7 | 7 | 4 | 2 | 2 | 5 | 4 | 2 | 1 | 1 |
| SDPC | 1 | 1 | 3 | 2 | 2 | 1 | 4 | 0 | 5 | 2 | 0 | 2 | 17 | 4 | 2 | 2 | 2 | 3 | 1 | 6 | 1 | 3 | 1 | 1 | 2 | 4 | 4 | 1 | 0 | 1 |
| DVYE | 1 | 1 | 3 | 2 | 2 | 0 | 4 | 1 | 0 | 1 | 0 | 2 | 16 | 4 | 3 | 3 | 1 | 3 | 2 | 4 | 2 | 3 | 1 | 1 | 3 | 4 | 2 | 2 | 0 | 2 |
| BAZF | 1 | 2 | 2 | 2 | 2 | 1 | 1 | 0 | 0 | 1 | 0 | 3 | 22 | 6 | 4 | 4 | 1 | 2 | 2 | 5 | 3 | 3 | 1 | 1 | 2 | 3 | 2 | 1 | 0 | 1 |
| JTIG | 1 | 3 | 3 | 1 | 2 | 1 | 2 | 2 | 6 | 1 | 0 | 2 | 17 | 4 | 2 | 2 | 1 | 4 | 2 | 6 | 2 | 4 | 0 | 1 | 1 | 4 | 4 | 1 | 0 | 3 |
| GYBH | 1 | 2 | 2 | 1 | 2 | 1 | 1 | 1 | 0 | 1 | 0 | 2 | 13 | 4 | 3 | 3 | 1 | 3 | 3 | 6 | 2 | 2 | 0 | 1 | 2 | 4 | 3 | 1 | 0 | 1 |
| ETGN | 0 | 1 | 3 | 1 | 0 | 0 | 2 | 1 | 0 | 1 | 0 | 2 | 10 | 5 | 2 | 2 | 0 | 6 | 3 | 5 | 2 | 2 | 3 | 3 | 0 | 3 | 2 | 1 | 0 | 2 |
| QYXY | 1 | 2 | 3 | 1 | 1 | 0 | 2 | 2 | 4 | 1 | 0 | 2 | 27 | 6 | 4 | 4 | 1 | 2 | 2 | 7 | 2 | 4 | 1 | 2 | 1 | 6 | 5 | 1 | 0 | 2 |
| GXBM | 1 | 2 | 4 | 1 | 2 | 0 | 1 | 0 | 0 | 2 | 0 | 2 | 29 | 7 | 4 | 4 | 1 | 2 | 2 | 7 | 2 | 4 | 1 | 3 | 0 | 6 | 5 | 1 | 0 | 1 |
| NKXU | 1 | 2 | 3 | 2 | 2 | 1 | 2 | 1 | 7 | 1 | 0 | 2 | 40 | 4 | 4 | 4 | 2 | 6 | 3 | 7 | 2 | 4 | 1 | 1 | 0 | 4 | 3 | 1 | 0 | 1 |
| ZNUM | 1 | 1 | 4 | 1 | 1 | 0 | 1 | 1 | 3 | 0 | 0 | 2 | 25 | 6 | 4 | 3 | 1 | 4 | 4 | 6 | 2 | 4 | 1 | 1 | 0 | 5 | 3 | 1 | 0 | 1 |
| WXRI | 1 | 2 | 4 | 2 | 2 | 1 | 1 | 2 | 1 | 1 | 0 | 1 | 14 | 4 | 2 | 2 | 1 | 2 | 2 | 4 | 1 | 4 | 1 | 1 | 0 | 5 | 5 | 1 | 0 | 1 |
| WCLV | 1 | 1 | 4 | 2 | 2 | 0 | 1 | 1 | 0 | 0 | 0 | 1 | 17 | 4 | 4 | 4 | 1 | 2 | 2 | 6 | 1 | 4 | 1 | 1 | 0 | 5 | 3 | 1 | 0 | 1 |
| EEJO | 1 | 3 | 4 | 2 | 3 | 1 | 2 | 2 | 0 | 1 | 0 | 2 | 20 | 5 | 4 | 4 | 2 | 5 | 5 | 8 | 2 | 4 | 1 | 2 | 1 | 4 | 4 | 1 | 0 | 2 |
| AKCR | 1 | 5 | 3 | 3 | 2 | 1 | 4 | 0 | 0 | 1 | 0 | 2 | 28 | 6 | 4 | 3 | 1 | 3 | 3 | 8 | 3 | 4 | 1 | 2 | 1 | 4 | 4 | 1 | 0 | 2 |
| BILC | 0 | 0 | 2 | 2 | 1 | 0 | 1 | 0 | 0 | 0 | 0 | 2 | 14 | 4 | 2 | 2 | 1 | 2 | 2 | 4 | 2 | 3 | 1 | 2 | 0 | 2 | 2 | 0 | 0 | 2 |
| JQFK | 1 | 2 | 6 | 3 | 3 | 1 | 3 | 1 | 5 | 1 | 0 | 2 | 16 | 8 | 3 | 3 | 2 | 18 | 5 | 12 | 5 | 6 | 0 | 3 | 3 | 5 | 5 | 3 | 1 | 6 |
| MNCB | 1 | 6 | 2 | 2 | 0 | 1 | 6 | 0 | 5 | 1 | 0 | 2 | 16 | 3 | 1 | 1 | 0 | 4 | 2 | 2 | 1 | 3 | 1 | 5 | 3 | 5 | 2 | 0 | 0 | 3 |
| MFYC | 1 | 4 | 3 | 3 | 2 | 1 | 5 | 1 | 3 | 1 | 0 | 2 | 14 | 4 | 3 | 2 | 1 | 4 | 3 | 5 | 3 | 4 | 1 | 1 | 1 | 6 | 5 | 1 | 0 | 1 |
| HHXJ | 1 | 2 | 5 | 2 | 2 | 1 | 2 | 0 | 0 | 1 | 0 | 3 | 7 | 3 | 3 | 3 | 1 | 2 | 2 | 2 | 1 | 3 | 0 | 2 | 1 | 5 | 3 | 1 | 0 | 1 |
| HVNO | 1 | 2 | 4 | 2 | 4 | 1 | 2 | 0 | 0 | 1 | 0 | 2 | 17 | 5 | 3 | 3 | 1 | 4 | 3 | 6 | 2 | 4 | 1 | 2 | 2 | 7 | 5 | 2 | 0 | 2 |
| DUMA | 2 | 4 | 8 | 5 | 4 | 0 | 3 | 0 | 0 | 2 | 0 | 2 | 18 | 4 | 5 | 5 | 1 | 5 | 4 | 4 | 3 | 4 | 0 | 3 | 1 | 5 | 4 | 1 | 0 | 1 |
| FMVB | 1 | 4 | 5 | 2 | 3 | 2 | 2 | 1 | 0 | 2 | 0 | 2 | 11 | 3 | 5 | 5 | 0 | 2 | 2 | 4 | 2 | 4 | 0 | 2 | 1 | 6 | 4 | 1 | 0 | 1 |
| PUAN | 1 | 1 | 4 | 3 | 2 | 1 | 1 | 0 | 2 | 1 | 0 | 1 | 7 | 4 | 4 | 4 | 1 | 3 | 3 | 3 | 1 | 3 | 0 | 1 | 1 | 3 | 2 | 1 | 0 | 0 |
| RRSV | 2 | 2 | 5 | 3 | 1 | 1 | 3 | 2 | 1 | 1 | 0 | 1 | 4 | 4 | 2 | 2 | 1 | 4 | 4 | 2 | 1 | 3 | 0 | 2 | 2 | 3 | 2 | 1 | 0 | 0 |
| AYPS | 1 | 2 | 3 | 2 | 1 | 1 | 2 | 1 | 0 | 1 | 0 | 3 | 13 | 6 | 3 | 3 | 1 | 2 | 2 | 4 | 3 | 3 | 0 | 1 | 0 | 3 | 3 | 1 | 0 | 1 |
| TGNL | 1 | 5 | 7 | 3 | 2 | 1 | 5 | 0 | 0 | 1 | 0 | 2 | 12 | 4 | 2 | 2 | 0 | 8 | 7 | 3 | 1 | 4 | 0 | 2 | 1 | 4 | 4 | 1 | 0 | 1 |
| MMKU | 1 | 1 | 4 | 2 | 4 | 1 | 2 | 2 | 1 | 1 | 0 | 2 | 20 | 6 | 6 | 5 | 2 | 6 | 5 | 7 | 2 | 4 | 0 | 3 | 1 | 8 | 4 | 1 | 0 | 0 |
| ISIM | 1 | 1 | 4 | 1 | 1 | 1 | 4 | 2 | 0 | 1 | 0 | 2 | 14 | 3 | 3 | 3 | 1 | 4 | 2 | 4 | 2 | 3 | 0 | 1 | 1 | 5 | 3 | 2 | 0 | 1 |
| MCPK | 2 | 5 | 6 | 5 | 2 | 1 | 2 | 0 | 1 | 1 | 0 | 2 | 5 | 8 | 3 | 3 | 1 | 7 | 4 | 3 | 3 | 3 | 0 | 3 | 2 | 7 | 3 | 1 | 0 | 2 |
| QXSZ | 1 | 2 | 4 | 2 | 1 | 0 | 3 | 1 | 2 | 1 | 0 | 2 | 4 | 6 | 3 | 3 | 2 | 8 | 3 | 3 | 4 | 3 | 1 | 2 | 1 | 3 | 1 | 0 | 0 | 0 |
| BTFM | 1 | 2 | 3 | 2 | 1 | 1 | 2 | 4 | 0 | 1 | 0 | 1 | 10 | 5 | 3 | 2 | 1 | 5 | 2 | 4 | 1 | 4 | 0 | 2 | 0 | 5 | 3 | 1 | 0 | 0 |
| XOAL | 1 | 2 | 4 | 3 | 1 | 0 | 1 | 1 | 2 | 0 | 0 | 1 | 15 | 3 | 3 | 3 | 2 | 7 | 5 | 8 | 6 | 4 | 0 | 2 | 0 | 2 | 2 | 1 | 0 | 1 |
| TNAW | 2 | 5 | 4 | 4 | 2 | 2 | 7 | 7 | 2 | 2 | 0 | 1 | 14 | 5 | 3 | 2 | 1 | 13 | 5 | 3 | 2 | 2 | 0 | 4 | 1 | 3 | 2 | 1 | 0 | 1 |
| JMTE | 1 | 3 | 5 | 3 | 1 | 0 | 2 | 0 | 1 | 1 | 0 | 2 | 14 | 4 | 1 | 1 | 1 | 11 | 3 | 7 | 2 | 4 | 0 | 3 | 0 | 5 | 2 | 0 | 0 | 1 |
| EGNB | 2 | 5 | 5 | 3 | 2 | 2 | 4 | 1 | 0 | 1 | 0 | 1 | 2 | 5 | 1 | 1 | 0 | 6 | 2 | 2 | 2 | 3 | 0 | 3 | 0 | 3 | 2 | 0 | 0 | 0 |
| HYHN | 2 | 3 | 4 | 1 | 2 | 0 | 4 | 0 | 0 | 0 | 0 | 3 | 12 | 4 | 2 | 2 | 0 | 6 | 4 | 7 | 2 | 4 | 0 | 2 | 1 | 5 | 2 | 0 | 0 | 1 |
| XMCL | 1 | 3 | 2 | 1 | 1 | 0 | 6 | 0 | 0 | 0 | 0 | 2 | 7 | 5 | 1 | 1 | 1 | 4 | 3 | 3 | 2 | 4 | 0 | 1 | 2 | 3 | 3 | 0 | 0 | 0 |
| AZZW | 1 | 2 | 3 | 2 | 4 | 1 | 3 | 1 | 0 | 1 | 0 | 2 | 25 | 4 | 2 | 3 | 2 | 11 | 3 | 10 | 2 | 3 | 1 | 3 | 0 | 4 | 4 | 0 | 0 | 1 |
| NNHQ | 1 | 3 | 4 | 2 | 1 | 1 | 2 | 0 | 5 | 1 | 0 | 1 | 9 | 4 | 2 | 2 | 1 | 4 | 4 | 9 | 2 | 2 | 1 | 2 | 0 | 4 | 2 | 0 | 0 | 1 |
| KYIO | 1 | 1 | 2 | 1 | 2 | 1 | 0 | 3 | 2 | 1 | 0 | 2 | 13 | 6 | 2 | 2 | 1 | 3 | 3 | 3 | 3 | 2 | 1 | 1 | 0 | 5 | 5 | 1 | 1 | 2 |
| FQLP | 1 | 4 | 5 | 2 | 2 | 0 | 4 | 5 | 0 | 0 | 0 | 2 | 19 | 6 | 1 | 1 | 1 | 3 | 3 | 9 | 3 | 3 | 1 | 3 | 1 | 4 | 2 | 0 | 0 | 1 |
| FPCO | 1 | 3 | 5 | 2 | 3 | 0 | 6 | 2 | 0 | 0 | 0 | 2 | 33 | 6 | 4 | 4 | 2 | 4 | 4 | 11 | 3 | 3 | 1 | 4 | 0 | 4 | 3 | 0 | 0 | 1 |
| BFIK | 1 | 2 | 4 | 2 | 2 | 1 | 2 | 2 | 0 | 1 | 0 | 2 | 21 | 7 | 5 | 2 | 1 | 6 | 5 | 8 | 3 | 2 | 1 | 4 | 1 | 4 | 4 | 0 | 0 | 1 |
| MWXT | 0 | 1 | 2 | 1 | 2 | 1 | 2 | 1 | 2 | 1 | 0 | 0 | 4 | 4 | 4 | 1 | 0 | 8 | 4 | 5 | 0 | 2 | 0 | 2 | 1 | 3 | 1 | 0 | 0 | 2 |
| VQBJ | 2 | 1 | 4 | 2 | 3 | 0 | 2 | 3 | 0 | 0 | 0 | 2 | 12 | 7 | 3 | 3 | 1 | 3 | 3 | 10 | 3 | 2 | 1 | 2 | 1 | 4 | 3 | 0 | 0 | 2 |
| QPDY | 2 | 3 | 6 | 2 | 2 | 1 | 2 | 2 | 0 | 1 | 0 | 2 | 21 | 7 | 4 | 4 | 1 | 3 | 3 | 8 | 3 | 2 | 1 | 2 | 1 | 4 | 3 | 0 | 0 | 2 |
| DRGY | 1 | 1 | 3 | 1 | 2 | 1 | 1 | 5 | 0 | 1 | 0 | 2 | 12 | 6 | 2 | 2 | 2 | 6 | 5 | 8 | 3 | 2 | 1 | 1 | 1 | 3 | 2 | 0 | 0 | 2 |
| HAOX | 1 | 3 | 3 | 2 | 2 | 1 | 2 | 1 | 0 | 1 | 0 | 1 | 8 | 6 | 3 | 3 | 1 | 4 | 3 | 3 | 3 | 2 | 1 | 1 | 0 | 4 | 3 | 0 | 0 | 2 |
| WSJO | 1 | 2 | 4 | 3 | 3 | 0 | 2 | 1 | 0 | 1 | 0 | 2 | 23 | 6 | 3 | 3 | 2 | 4 | 4 | 8 | 3 | 2 | 1 | 2 | 0 | 4 | 2 | 0 | 0 | 1 |
| WDCW | 1 | 2 | 6 | 2 | 3 | 0 | 1 | 2 | 1 | 1 | 0 | 2 | 20 | 10 | 4 | 4 | 2 | 6 | 4 | 8 | 3 | 2 | 1 | 3 | 1 | 5 | 4 | 0 | 0 | 1 |
| YOXI | 1 | 2 | 5 | 2 | 4 | 0 | 5 | 3 | 0 | 0 | 0 | 3 | 23 | 7 | 3 | 3 | 1 | 4 | 4 | 8 | 4 | 2 | 1 | 2 | 0 | 4 | 4 | 0 | 0 | 2 |
| RPGL | 1 | 0 | 5 | 2 | 5 | 0 | 4 | 2 | 0 | 0 | 0 | 3 | 16 | 6 | 3 | 2 | 1 | 4 | 4 | 7 | 3 | 2 | 1 | 3 | 0 | 4 | 4 | 0 | 0 | 2 |
| HKZW | 1 | 3 | 5 | 2 | 3 | 1 | 4 | 2 | 0 | 1 | 0 | 2 | 13 | 7 | 3 | 3 | 1 | 9 | 5 | 7 | 4 | 2 | 3 | 2 | 0 | 4 | 2 | 0 | 0 | 3 |
| JOJQ | 1 | 4 | 5 | 2 | 2 | 1 | 4 | 3 | 4 | 1 | 0 | 2 | 21 | 6 | 3 | 3 | 1 | 4 | 4 | 9 | 3 | 2 | 1 | 2 | 1 | 4 | 2 | 0 | 0 | 1 |
| MFZO | 1 | 2 | 4 | 2 | 3 | 1 | 3 | 2 | 0 | 1 | 0 | 2 | 21 | 7 | 3 | 3 | 1 | 6 | 3 | 8 | 3 | 2 | 1 | 1 | 1 | 4 | 3 | 0 | 0 | 2 |
| FFGR | 1 | 1 | 4 | 2 | 2 | 1 | 0 | 2 | 0 | 1 | 0 | 2 | 14 | 7 | 3 | 1 | 1 | 3 | 3 | 6 | 3 | 2 | 1 | 1 | 1 | 4 | 2 | 0 | 0 | 3 |
| KMNX | 1 | 1 | 3 | 2 | 2 | 1 | 2 | 2 | 2 | 1 | 0 | 1 | 8 | 5 | 3 | 2 | 1 | 3 | 3 | 5 | 1 | 2 | 1 | 2 | 0 | 3 | 3 | 0 | 0 | 2 |
| SNOX | 1 | 2 | 4 | 2 | 3 | 1 | 1 | 3 | 0 | 1 | 0 | 2 | 9 | 5 | 3 | 3 | 1 | 5 | 4 | 7 | 2 | 2 | 1 | 2 | 0 | 4 | 2 | 0 | 0 | 2 |
| XRTZ | 1 | 2 | 4 | 2 | 3 | 0 | 2 | 1 | 0 | 0 | 0 | 2 | 15 | 5 | 3 | 3 | 1 | 4 | 4 | 6 | 3 | 3 | 1 | 2 | 1 | 4 | 2 | 0 | 0 | 3 |
| KEYW | 1 | 4 | 6 | 2 | 4 | 1 | 5 | 2 | 0 | 1 | 0 | 2 | 13 | 7 | 2 | 2 | 2 | 4 | 4 | 6 | 3 | 3 | 2 | 2 | 0 | 4 | 2 | 0 | 0 | 3 |
| DRFX | 1 | 5 | 2 | 1 | 1 | 1 | 4 | 0 | 0 | 1 | 0 | 2 | 5 | 3 | 3 | 1 | 0 | 10 | 4 | 4 | 4 | 2 | 1 | 2 | 5 | 1 | 0 | 0 | 0 | 2 |
| AEKF | 1 | 3 | 4 | 2 | 2 | 2 | 3 | 1 | 0 | 2 | 0 | 2 | 11 | 2 | 2 | 2 | 1 | 6 | 2 | 2 | 3 | 2 | 1 | 2 | 2 | 3 | 2 | 0 | 0 | 2 |
| YSQT | 1 | 2 | 4 | 2 | 3 | 1 | 2 | 1 | 0 | 1 | 0 | 2 | 14 | 5 | 2 | 2 | 2 | 6 | 4 | 6 | 4 | 2 | 1 | 2 | 1 | 4 | 3 | 0 | 0 | 2 |
| RPQV | 1 | 4 | 4 | 2 | 3 | 1 | 3 | 2 | 0 | 1 | 0 | 2 | 19 | 6 | 3 | 2 | 1 | 7 | 3 | 6 | 3 | 2 | 1 | 1 | 1 | 5 | 3 | 0 | 0 | 3 |
| HIDG | 1 | 4 | 2 | 2 | 2 | 1 | 3 | 1 | 0 | 1 | 0 | 2 | 9 | 3 | 1 | 1 | 1 | 6 | 3 | 2 | 1 | 2 | 2 | 3 | 1 | 1 | 0 | 0 | 0 | 2 |
| RQFE | 1 | 2 | 4 | 2 | 2 | 1 | 1 | 1 | 0 | 1 | 0 | 2 | 17 | 5 | 2 | 2 | 1 | 5 | 5 | 6 | 3 | 2 | 1 | 1 | 3 | 3 | 2 | 0 | 0 | 2 |
| GYRP | 1 | 2 | 3 | 1 | 3 | 1 | 3 | 1 | 0 | 1 | 0 | 2 | 12 | 4 | 2 | 2 | 1 | 6 | 3 | 7 | 3 | 2 | 1 | 2 | 1 | 4 | 3 | 0 | 0 | 3 |
| MNNM | 1 | 1 | 4 | 2 | 2 | 1 | 1 | 1 | 0 | 1 | 0 | 2 | 10 | 5 | 3 | 2 | 1 | 5 | 5 | 4 | 3 | 2 | 1 | 2 | 1 | 3 | 3 | 0 | 0 | 2 |
| ISHC | 2 | 2 | 3 | 4 | 3 | 2 | 2 | 1 | 1 | 2 | 0 | 2 | 11 | 7 | 3 | 3 | 1 | 8 | 5 | 6 | 3 | 1 | 1 | 4 | 4 | 5 | 2 | 0 | 0 | 3 |
| QWFV | 1 | 3 | 3 | 4 | 2 | 1 | 3 | 1 | 0 | 1 | 0 | 1 | 10 | 5 | 4 | 3 | 1 | 13 | 4 | 4 | 4 | 2 | 2 | 1 | 5 | 4 | 3 | 0 | 0 | 5 |
| DFDS | 2 | 3 | 5 | 2 | 3 | 2 | 2 | 1 | 0 | 2 | 0 | 2 | 8 | 5 | 3 | 3 | 1 | 7 | 2 | 6 | 3 | 2 | 1 | 2 | 1 | 4 | 2 | 0 | 0 | 2 |
| GGWH | 1 | 5 | 5 | 2 | 3 | 0 | 6 | 2 | 0 | 0 | 0 | 2 | 22 | 5 | 3 | 3 | 1 | 8 | 5 | 7 | 3 | 2 | 1 | 4 | 1 | 5 | 4 | 0 | 0 | 3 |
| WCQU | 1 | 3 | 4 | 2 | 2 | 1 | 4 | 3 | 0 | 1 | 0 | 2 | 18 | 6 | 3 | 2 | 1 | 7 | 4 | 6 | 3 | 2 | 1 | 2 | 1 | 4 | 2 | 0 | 0 | 3 |
| BHBK | 1 | 0 | 4 | 2 | 3 | 0 | 2 | 2 | 0 | 0 | 0 | 3 | 14 | 6 | 3 | 3 | 1 | 4 | 3 | 6 | 3 | 6 | 1 | 0 | 1 | 5 | 4 | 0 | 0 | 3 |
| RPRU | 1 | 1 | 4 | 2 | 3 | 0 | 2 | 3 | 0 | 1 | 0 | 2 | 15 | 7 | 3 | 3 | 1 | 7 | 4 | 6 | 3 | 2 | 1 | 3 | 1 | 4 | 2 | 0 | 0 | 2 |
| MCHJ | 1 | 3 | 4 | 1 | 3 | 1 | 5 | 1 | 1 | 1 | 0 | 2 | 15 | 6 | 3 | 3 | 1 | 3 | 3 | 6 | 3 | 2 | 1 | 2 | 1 | 4 | 3 | 0 | 0 | 3 |
| GBGT | 0 | 2 | 3 | 2 | 3 | 1 | 1 | 1 | 0 | 1 | 0 | 1 | 8 | 4 | 3 | 3 | 1 | 3 | 3 | 3 | 3 | 4 | 1 | 2 | 1 | 4 | 2 | 0 | 0 | 2 |
| MOYY | 0 | 2 | 2 | 2 | 2 | 1 | 1 | 1 | 0 | 1 | 0 | 2 | 8 | 3 | 2 | 2 | 1 | 3 | 0 | 3 | 3 | 2 | 1 | 0 | 1 | 1 | 0 | 0 | 0 | 1 |
| STKJ | 1 | 1 | 6 | 2 | 3 | 1 | 2 | 0 | 0 | 1 | 0 | 2 | 9 | 4 | 2 | 2 | 1 | 7 | 5 | 6 | 2 | 2 | 1 | 2 | 3 | 3 | 1 | 0 | 0 | 2 |
| WDGV | 1 | 4 | 5 | 2 | 4 | 1 | 5 | 2 | 1 | 1 | 0 | 2 | 17 | 6 | 3 | 3 | 2 | 5 | 5 | 6 | 3 | 2 | 1 | 6 | 3 | 5 | 3 | 0 | 0 | 3 |
| MXEZ | 1 | 3 | 4 | 2 | 1 | 1 | 2 | 0 | 1 | 1 | 0 | 2 | 12 | 5 | 1 | 1 | 1 | 3 | 2 | 6 | 2 | 4 | 0 | 2 | 0 | 4 | 2 | 0 | 0 | 1 |
| MJMQ | 2 | 4 | 4 | 0 | 0 | 1 | 4 | 0 | 13 | 1 | 1 | 1 | 2 | 2 | 0 | 0 | 0 | 5 | 4 | 2 | 0 | 1 | 2 | 3 | 1 | 3 | 2 | 0 | 0 | 1 |
| YTYU | 1 | 1 | 3 | 2 | 2 | 1 | 1 | 0 | 2 | 1 | 0 | 3 | 9 | 2 | 1 | 1 | 1 | 2 | 2 | 2 | 2 | 3 | 0 | 3 | 3 | 6 | 2 | 1 | 4 | 0 |
| DXNY | 1 | 1 | 4 | 1 | 2 | 1 | 1 | 2 | 0 | 1 | 0 | 2 | 21 | 5 | 4 | 4 | 1 | 3 | 3 | 6 | 2 | 4 | 2 | 2 | 1 | 5 | 4 | 1 | 0 | 2 |
| AGIO | 1 | 3 | 3 | 2 | 1 | 2 | 2 | 1 | 0 | 1 | 0 | 2 | 9 | 5 | 3 | 2 | 1 | 3 | 1 | 2 | 2 | 2 | 1 | 2 | 2 | 3 | 3 | 0 | 0 | 2 |
| ODXI | 1 | 1 | 4 | 3 | 2 | 1 | 1 | 1 | 2 | 1 | 0 | 2 | 15 | 5 | 3 | 2 | 1 | 4 | 1 | 4 | 2 | 3 | 1 | 2 | 2 | 3 | 2 | 0 | 0 | 1 |
| NTLE | 1 | 4 | 2 | 2 | 2 | 1 | 5 | 1 | 1 | 1 | 0 | 2 | 12 | 5 | 2 | 1 | 1 | 11 | 5 | 2 | 2 | 2 | 0 | 2 | 2 | 3 | 3 | 2 | 0 | 0 |
| HJVM | 1 | 2 | 4 | 2 | 2 | 1 | 1 | 1 | 0 | 1 | 0 | 2 | 11 | 6 | 3 | 2 | 1 | 5 | 3 | 6 | 4 | 2 | 1 | 1 | 3 | 4 | 1 | 0 | 0 | 2 |

| sample code | ec4.1.3.1 | ec5.3.1.1 | ec6.2.1.1 | ec6.4.1.3 | ec6.4.1.4 | oxoglutarate-malate transpor | ec1.1.1.101 | ec1.1.1.94 | ec2.3.1.23 | ec2.3.1.51 | ec2.7.1.107 | ec2.7.1.30 | ec2.7.7.41 | ec2.7.8.5 | ec3.1.1.5 | ec3.1.3.27 | ec3.1.3.4 | ec3.1.4.3 | PF03062 | acyl carrier protein | ec1.1.1.100 | ec1.14.19.1 | ec1.14.19.2 | ec1.14.19.3 | ec1.14.19.4 | ec1.14.99.33 | ec1.3.1.38 | ec1.3.1.9 |
|---|---|---|---|---|---|---|---|---|---|---|---|---|---|---|---|---|---|---|---|---|---|---|---|---|---|---|---|---|
| ISGT | 1 | 1 | 3 | 4 | 3 | 2 | 3 | 1 | 2 | 1 | 0 | 1 | 1 | 2 | 0 | 0 | 1 | 0 | 2 | 3 | 19 | 1 | 1 | 0 | 0 | 0 | 1 | 1 |
| RAWF | 1 | 1 | 3 | 6 | 5 | 2 | 2 | 3 | 1 | 7 | 0 | 1 | 2 | 3 | 2 | 0 | 2 | 0 | 1 | 2 | 24 | 1 | 2 | 1 | 0 | 1 | 1 | 2 |
| IJMT | 2 | 2 | 13 | 10 | 11 | 2 | 6 | 2 | 4 | 12 | 2 | 3 | 4 | 4 | 5 | 0 | 1 | 2 | 5 | 4 | 65 | 4 | 1 | 0 | 0 | 1 | 6 | 2 |
| SDPC | 1 | 1 | 2 | 3 | 3 | 4 | 4 | 1 | 2 | 5 | 1 | 1 | 2 | 3 | 1 | 0 | 1 | 0 | 1 | 2 | 29 | 1 | 2 | 0 | 0 | 1 | 1 | 1 |
| DVYE | 1 | 1 | 3 | 2 | 1 | 3 | 3 | 2 | 2 | 5 | 0 | 1 | 2 | 2 | 4 | 0 | 2 | 0 | 2 | 2 | 28 | 1 | 1 | 0 | 0 | 1 | 1 | 1 |
| BAZF | 1 | 1 | 6 | 2 | 3 | 2 | 5 | 3 | 1 | 4 | 1 | 1 | 2 | 4 | 4 | 0 | 2 | 0 | 2 | 2 | 29 | 1 | 2 | 0 | 0 | 1 | 2 | 2 |
| JTIG | 0 | 1 | 1 | 3 | 2 | 3 | 4 | 2 | 3 | 9 | 2 | 1 | 2 | 4 | 3 | 0 | 3 | 0 | 2 | 2 | 33 | 2 | 1 | 1 | 1 | 2 | 3 | 1 |
| GYBH | 0 | 1 | 1 | 5 | 5 | 2 | 5 | 2 | 3 | 9 | 1 | 1 | 2 | 6 | 3 | 0 | 3 | 0 | 3 | 2 | 28 | 1 | 1 | 1 | 1 | 2 | 0 | 1 |
| ETGN | 2 | 1 | 1 | 0 | 1 | 0 | 2 | 0 | 0 | 1 | 0 | 0 | 0 | 0 | 0 | 0 | 0 | 0 | 0 | 3 | 20 | 1 | 1 | 0 | 0 | 1 | 1 | 0 |
| QYXY | 1 | 2 | 6 | 7 | 6 | 3 | 7 | 3 | 5 | 9 | 2 | 1 | 2 | 3 | 5 | 0 | 1 | 0 | 2 | 2 | 45 | 1 | 1 | 1 | 1 | 0 | 4 | 2 |
| GXBM | 1 | 2 | 6 | 5 | 6 | 2 | 3 | 2 | 5 | 10 | 2 | 1 | 2 | 5 | 6 | 0 | 2 | 0 | 2 | 2 | 42 | 1 | 1 | 1 | 1 | 1 | 4 | 2 |
| NKXU | 1 | 2 | 7 | 4 | 5 | 2 | 3 | 2 | 3 | 6 | 2 | 1 | 2 | 2 | 4 | 0 | 3 | 0 | 3 | 2 | 68 | 2 | 1 | 2 | 0 | 2 | 3 | 2 |
| ZNUM | 1 | 2 | 5 | 6 | 7 | 2 | 3 | 2 | 4 | 9 | 2 | 1 | 2 | 4 | 3 | 0 | 5 | 0 | 3 | 3 | 52 | 2 | 2 | 1 | 0 | 2 | 1 | 2 |
| WXRI | 1 | 1 | 2 | 2 | 3 | 2 | 0 | 2 | 3 | 6 | 0 | 1 | 2 | 1 | 2 | 0 | 1 | 0 | 2 | 2 | 29 | 1 | 1 | 0 | 0 | 2 | 8 | 1 |
| WCLV | 1 | 1 | 5 | 3 | 4 | 2 | 1 | 2 | 3 | 8 | 1 | 1 | 2 | 1 | 1 | 0 | 2 | 0 | 3 | 2 | 30 | 1 | 1 | 1 | 0 | 2 | 1 | 1 |
| EEJO | 1 | 3 | 9 | 6 | 7 | 2 | 2 | 3 | 2 | 9 | 2 | 1 | 2 | 3 | 6 | 0 | 3 | 0 | 3 | 2 | 40 | 1 | 1 | 0 | 0 | 2 | 4 | 2 |
| AKCR | 1 | 2 | 5 | 4 | 5 | 2 | 2 | 3 | 2 | 7 | 1 | 1 | 2 | 3 | 4 | 0 | 3 | 0 | 2 | 4 | 49 | 2 | 2 | 0 | 0 | 2 | 4 | 1 |
| BILC | 1 | 2 | 4 | 5 | 5 | 2 | 2 | 2 | 2 | 5 | 0 | 1 | 1 | 3 | 2 | 0 | 2 | 0 | 3 | 2 | 18 | 0 | 2 | 0 | 0 | 1 | 2 | 2 |
| JQFK | 1 | 6 | 7 | 5 | 6 | 2 | 3 | 2 | 3 | 6 | 2 | 1 | 2 | 5 | 2 | 1 | 2 | 2 | 3 | 11 | 29 | 4 | 2 | 1 | 0 | 2 | 4 | 4 |
| MNCB | 1 | 1 | 3 | 3 | 4 | 2 | 2 | 1 | 2 | 5 | 0 | 1 | 2 | 4 | 3 | 0 | 2 | 0 | 1 | 1 | 28 | 1 | 2 | 0 | 0 | 1 | 1 | 1 |
| MFYC | 1 | 2 | 3 | 4 | 4 | 2 | 1 | 3 | 4 | 10 | 1 | 1 | 2 | 2 | 3 | 0 | 1 | 0 | 2 | 2 | 25 | 2 | 1 | 1 | 0 | 1 | 1 | 2 |
| HHXJ | 0 | 1 | 4 | 5 | 5 | 4 | 2 | 2 | 1 | 5 | 0 | 1 | 2 | 0 | 1 | 0 | 0 | 0 | 1 | 2 | 23 | 2 | 1 | 2 | 1 | 0 | 1 | 2 |
| HVNO | 1 | 3 | 4 | 4 | 3 | 2 | 1 | 2 | 2 | 6 | 1 | 1 | 1 | 2 | 1 | 0 | 1 | 0 | 2 | 2 | 33 | 3 | 2 | 2 | 1 | 0 | 1 | 1 |
| DUMA | 0 | 3 | 3 | 5 | 6 | 4 | 3 | 2 | 2 | 6 | 1 | 1 | 1 | 1 | 3 | 0 | 1 | 0 | 3 | 2 | 30 | 2 | 2 | 1 | 0 | 0 | 1 | 2 |
| FMVB | 0 | 2 | 6 | 5 | 6 | 3 | 1 | 3 | 1 | 5 | 1 | 1 | 2 | 3 | 2 | 0 | 0 | 0 | 2 | 2 | 24 | 1 | 1 | 1 | 0 | 1 | 1 | 1 |
| PUAN | 0 | 1 | 4 | 2 | 2 | 2 | 0 | 1 | 1 | 4 | 1 | 1 | 3 | 2 | 2 | 0 | 2 | 0 | 2 | 3 | 18 | 1 | 2 | 1 | 0 | 2 | 2 | 1 |
| RRSV | 0 | 1 | 2 | 1 | 2 | 2 | 0 | 1 | 2 | 7 | 1 | 1 | 2 | 1 | 3 | 0 | 2 | 0 | 1 | 4 | 12 | 1 | 2 | 0 | 0 | 1 | 2 | 1 |
| AYPS | 0 | 1 | 3 | 4 | 5 | 2 | 1 | 3 | 1 | 7 | 2 | 1 | 2 | 3 | 5 | 0 | 1 | 0 | 2 | 2 | 24 | 1 | 1 | 1 | 0 | 2 | 2 | 2 |
| TGNL | 0 | 2 | 3 | 1 | 3 | 2 | 2 | 3 | 4 | 8 | 2 | 1 | 2 | 3 | 4 | 0 | 0 | 0 | 3 | 2 | 25 | 1 | 1 | 2 | 0 | 3 | 2 | 1 |
| MMKU | 0 | 3 | 6 | 5 | 6 | 2 | 2 | 1 | 1 | 8 | 2 | 1 | 2 | 4 | 2 | 0 | 1 | 0 | 3 | 2 | 38 | 2 | 1 | 2 | 0 | 3 | 2 | 1 |
| ISIM | 0 | 2 | 1 | 4 | 3 | 2 | 0 | 2 | 2 | 8 | 1 | 1 | 1 | 2 | 3 | 0 | 0 | 0 | 2 | 2 | 35 | 2 | 2 | 3 | 0 | 3 | 2 | 2 |
| MCPK | 0 | 3 | 2 | 1 | 3 | 4 | 1 | 2 | 0 | 4 | 2 | 1 | 2 | 3 | 2 | 0 | 1 | 0 | 1 | 4 | 18 | 2 | 2 | 2 | 1 | 2 | 0 | 2 |
| QXSZ | 1 | 3 | 3 | 0 | 1 | 1 | 1 | 1 | 1 | 3 | 1 | 0 | 2 | 1 | 1 | 0 | 1 | 0 | 0 | 3 | 12 | 1 | 1 | 2 | 1 | 2 | 0 | 0 |
| BTFM | 0 | 2 | 2 | 1 | 4 | 2 | 1 | 1 | 3 | 9 | 2 | 1 | 2 | 3 | 5 | 0 | 0 | 0 | 3 | 2 | 23 | 3 | 2 | 1 | 0 | 4 | 0 | 1 |
| XOAL | 0 | 2 | 3 | 3 | 3 | 2 | 1 | 2 | 3 | 7 | 2 | 1 | 2 | 1 | 1 | 1 | 0 | 1 | 2 | 4 | 33 | 2 | 1 | 3 | 1 | 4 | 2 | 2 |
| TNAW | 0 | 4 | 2 | 3 | 3 | 3 | 0 | 1 | 1 | 8 | 1 | 0 | 2 | 3 | 2 | 0 | 0 | 0 | 2 | 3 | 32 | 3 | 1 | 2 | 0 | 4 | 2 | 1 |
| JMTE | 0 | 1 | 4 | 3 | 4 | 2 | 1 | 2 | 4 | 6 | 0 | 1 | 2 | 4 | 1 | 0 | 1 | 0 | 3 | 2 | 31 | 1 | 1 | 1 | 1 | 2 | 2 | 1 |
| EGNB | 0 | 6 | 1 | 1 | 1 | 2 | 0 | 4 | 1 | 2 | 0 | 1 | 2 | 0 | 1 | 0 | 0 | 0 | 3 | 2 | 11 | 1 | 1 | 1 | 0 | 0 | 1 | 2 |
| HYHN | 0 | 3 | 4 | 4 | 4 | 2 | 1 | 1 | 0 | 6 | 2 | 1 | 2 | 2 | 2 | 0 | 1 | 0 | 3 | 4 | 25 | 2 | 1 | 1 | 0 | 2 | 0 | 2 |
| XMCL | 0 | 1 | 4 | 2 | 2 | 1 | 1 | 0 | 1 | 2 | 1 | 1 | 3 | 3 | 3 | 0 | 1 | 1 | 1 | 2 | 18 | 1 | 1 | 2 | 0 | 1 | 0 | 0 |
| AZZW | 1 | 2 | 6 | 4 | 5 | 1 | 0 | 1 | 3 | 11 | 2 | 1 | 2 | 2 | 2 | 0 | 1 | 0 | 3 | 2 | 37 | 2 | 1 | 0 | 0 | 2 | 2 | 1 |
| NNHQ | 1 | 2 | 6 | 4 | 3 | 2 | 1 | 2 | 4 | 8 | 1 | 1 | 2 | 2 | 2 | 0 | 1 | 0 | 3 | 2 | 17 | 1 | 1 | 0 | 0 | 0 | 1 | 1 |
| KYIO | 1 | 2 | 1 | 2 | 1 | 2 | 0 | 2 | 1 | 9 | 0 | 0 | 1 | 1 | 1 | 0 | 0 | 0 | 1 | 2 | 23 | 3 | 1 | 1 | 0 | 2 | 2 | 1 |
| FQLP | 1 | 2 | 7 | 4 | 4 | 2 | 4 | 1 | 3 | 9 | 1 | 1 | 2 | 1 | 5 | 0 | 2 | 1 | 2 | 2 | 29 | 3 | 1 | 2 | 1 | 2 | 2 | 1 |
| FPCO | 1 | 2 | 8 | 5 | 5 | 2 | 5 | 1 | 5 | 10 | 2 | 1 | 2 | 3 | 6 | 0 | 3 | 1 | 5 | 3 | 52 | 3 | 2 | 2 | 1 | 2 | 2 | 2 |
| BFIK | 1 | 2 | 9 | 5 | 4 | 2 | 1 | 1 | 5 | 12 | 2 | 0 | 2 | 4 | 3 | 0 | 3 | 1 | 1 | 2 | 31 | 3 | 1 | 1 | 0 | 2 | 2 | 1 |
| MWXT | 0 | 4 | 2 | 1 | 1 | 2 | 0 | 0 | 0 | 2 | 0 | 1 | 1 | 0 | 0 | 0 | 0 | 1 | 0 | 1 | 9 | 1 | 1 | 0 | 1 | 1 | 1 | 1 |
| VQBJ | 1 | 1 | 4 | 4 | 3 | 2 | 2 | 1 | 3 | 8 | 2 | 1 | 2 | 1 | 3 | 0 | 1 | 1 | 3 | 1 | 24 | 1 | 1 | 1 | 2 | 1 | 1 | 1 |
| QPDY | 1 | 2 | 5 | 4 | 3 | 2 | 4 | 1 | 5 | 10 | 2 | 1 | 2 | 2 | 5 | 0 | 1 | 2 | 4 | 2 | 38 | 2 | 1 | 1 | 2 | 2 | 1 | 1 |
| DRGY | 1 | 2 | 1 | 2 | 1 | 1 | 2 | 1 | 3 | 5 | 1 | 1 | 2 | 2 | 5 | 0 | 0 | 1 | 3 | 2 | 19 | 1 | 1 | 1 | 1 | 2 | 1 | 1 |
| HAOX | 2 | 2 | 3 | 1 | 1 | 2 | 1 | 1 | 1 | 5 | 0 | 0 | 2 | 1 | 5 | 0 | 1 | 0 | 0 | 2 | 12 | 2 | 1 | 2 | 1 | 2 | 1 | 1 |
| WSJO | 2 | 2 | 5 | 5 | 5 | 2 | 3 | 1 | 5 | 10 | 1 | 1 | 2 | 3 | 3 | 0 | 1 | 1 | 3 | 3 | 34 | 1 | 1 | 0 | 1 | 2 | 2 | 1 |
| WDCW | 1 | 3 | 6 | 6 | 5 | 2 | 3 | 1 | 4 | 9 | 2 | 1 | 2 | 1 | 5 | 0 | 2 | 1 | 4 | 4 | 37 | 2 | 1 | 0 | 1 | 1 | 2 | 2 |
| YOXI | 1 | 2 | 7 | 5 | 4 | 2 | 2 | 1 | 4 | 9 | 2 | 1 | 2 | 4 | 8 | 0 | 1 | 1 | 3 | 3 | 35 | 2 | 1 | 0 | 1 | 2 | 1 | 2 |
| RPGL | 1 | 2 | 5 | 5 | 4 | 2 | 2 | 1 | 5 | 8 | 2 | 0 | 2 | 1 | 5 | 0 | 0 | 1 | 3 | 3 | 22 | 2 | 1 | 0 | 1 | 1 | 0 | 1 |
| HKZW | 3 | 2 | 8 | 5 | 4 | 2 | 1 | 1 | 1 | 7 | 2 | 2 | 2 | 3 | 4 | 0 | 2 | 1 | 3 | 2 | 18 | 2 | 1 | 1 | 1 | 2 | 2 | 1 |
| JOJQ | 1 | 2 | 4 | 5 | 4 | 2 | 1 | 1 | 3 | 10 | 2 | 1 | 3 | 2 | 8 | 0 | 2 | 1 | 4 | 2 | 30 | 1 | 1 | 0 | 1 | 2 | 4 | 1 |
| MFZO | 1 | 2 | 7 | 6 | 4 | 2 | 2 | 1 | 4 | 10 | 2 | 1 | 3 | 3 | 10 | 0 | 2 | 2 | 3 | 2 | 30 | 2 | 1 | 1 | 1 | 2 | 2 | 1 |
| FFGR | 1 | 2 | 4 | 4 | 3 | 2 | 3 | 1 | 1 | 5 | 2 | 1 | 2 | 2 | 4 | 0 | 1 | 1 | 2 | 3 | 22 | 2 | 1 | 1 | 1 | 0 | 1 | 1 |
| KMNX | 3 | 2 | 1 | 1 | 1 | 2 | 0 | 0 | 1 | 4 | 1 | 1 | 1 | 1 | 2 | 0 | 0 | 0 | 0 | 3 | 12 | 1 | 2 | 0 | 1 | 0 | 0 | 1 |
| SNOX | 1 | 3 | 6 | 5 | 4 | 2 | 2 | 1 | 1 | 6 | 2 | 1 | 2 | 2 | 3 | 0 | 1 | 0 | 3 | 2 | 19 | 2 | 1 | 1 | 1 | 1 | 1 | 1 |
| XRTZ | 1 | 2 | 7 | 5 | 4 | 2 | 3 | 1 | 1 | 6 | 2 | 1 | 1 | 2 | 4 | 0 | 1 | 0 | 2 | 2 | 24 | 2 | 1 | 0 | 0 | 2 | 1 | 1 |
| KEYW | 1 | 2 | 4 | 5 | 4 | 2 | 3 | 1 | 2 | 8 | 1 | 1 | 2 | 2 | 5 | 0 | 2 | 1 | 3 | 3 | 21 | 2 | 1 | 1 | 1 | 3 | 1 | 1 |
| DRFX | 1 | 2 | 6 | 3 | 2 | 4 | 1 | 1 | 1 | 1 | 1 | 1 | 1 | 1 | 0 | 0 | 2 | 0 | 0 | 2 | 11 | 1 | 1 | 0 | 0 | 1 | 0 | 1 |
| AEKF | 1 | 2 | 5 | 4 | 4 | 1 | 0 | 1 | 1 | 4 | 1 | 1 | 2 | 1 | 6 | 0 | 1 | 0 | 2 | 2 | 20 | 1 | 1 | 0 | 1 | 1 | 0 | 1 |
| YSQT | 1 | 2 | 6 | 5 | 4 | 2 | 0 | 0 | 2 | 5 | 2 | 1 | 2 | 1 | 5 | 0 | 1 | 1 | 4 | 2 | 25 | 1 | 1 | 1 | 1 | 1 | 1 | 1 |
| RPQV | 1 | 2 | 6 | 4 | 4 | 2 | 1 | 0 | 2 | 7 | 2 | 1 | 2 | 2 | 2 | 0 | 1 | 0 | 4 | 2 | 31 | 2 | 1 | 0 | 1 | 1 | 2 | 1 |
| HIDG | 2 | 2 | 3 | 2 | 4 | 0 | 2 | 0 | 1 | 2 | 0 | 0 | 0 | 1 | 0 | 0 | 0 | 0 | 2 | 2 | 20 | 2 | 2 | 0 | 1 | 0 | 0 | 1 |
| RQFE | 1 | 2 | 6 | 5 | 4 | 2 | 1 | 0 | 3 | 7 | 2 | 1 | 2 | 1 | 4 | 0 | 1 | 1 | 4 | 2 | 25 | 2 | 1 | 0 | 1 | 2 | 1 | 1 |
| GYRP | 0 | 1 | 4 | 2 | 2 | 2 | 0 | 1 | 1 | 4 | 1 | 1 | 2 | 1 | 1 | 0 | 1 | 0 | 1 | 2 | 18 | 1 | 0 | 1 | 1 | 0 | 2 | 1 |
| MNNM | 1 | 1 | 4 | 5 | 4 | 1 | 1 | 1 | 1 | 4 | 2 | 1 | 2 | 1 | 4 | 0 | 1 | 0 | 1 | 2 | 19 | 1 | 2 | 1 | 1 | 1 | 0 | 1 |
| ISHC | 2 | 2 | 5 | 3 | 3 | 1 | 1 | 1 | 1 | 4 | 2 | 1 | 2 | 2 | 3 | 0 | 1 | 0 | 2 | 2 | 19 | 1 | 1 | 1 | 1 | 1 | 0 | 1 |
| QWFV | 2 | 1 | 4 | 1 | 3 | 2 | 0 | 0 | 2 | 4 | 1 | 0 | 2 | 1 | 3 | 0 | 0 | 0 | 2 | 3 | 18 | 1 | 1 | 0 | 1 | 0 | 2 | 1 |
| DFDS | 1 | 1 | 6 | 5 | 3 | 2 | 1 | 0 | 1 | 6 | 3 | 1 | 2 | 1 | 4 | 0 | 0 | 1 | 1 | 2 | 13 | 1 | 1 | 1 | 0 | 0 | 0 | 1 |
| GGWH | 1 | 1 | 7 | 5 | 4 | 2 | 1 | 1 | 3 | 7 | 3 | 1 | 2 | 1 | 6 | 0 | 1 | 1 | 4 | 5 | 31 | 1 | 1 | 0 | 0 | 2 | 0 | 1 |
| WCQU | 1 | 1 | 7 | 5 | 4 | 3 | 1 | 0 | 3 | 6 | 2 | 1 | 2 | 1 | 4 | 0 | 1 | 0 | 4 | 3 | 29 | 2 | 1 | 1 | 1 | 2 | 0 | 1 |
| BHBK | 1 | 3 | 8 | 5 | 3 | 2 | 1 | 0 | 3 | 8 | 2 | 1 | 2 | 1 | 7 | 0 | 1 | 0 | 2 | 3 | 26 | 3 | 2 | 0 | 1 | 2 | 1 | 1 |
| RPRU | 1 | 2 | 7 | 5 | 4 | 1 | 2 | 0 | 3 | 6 | 1 | 1 | 2 | 2 | 3 | 0 | 1 | 1 | 6 | 4 | 28 | 1 | 2 | 1 | 0 | 0 | 0 | 1 |
| MCHJ | 1 | 2 | 7 | 5 | 5 | 2 | 2 | 1 | 1 | 4 | 2 | 1 | 3 | 1 | 4 | 0 | 1 | 1 | 4 | 3 | 27 | 2 | 1 | 1 | 1 | 2 | 1 | 1 |
| GBGT | 1 | 2 | 7 | 3 | 1 | 2 | 1 | 0 | 1 | 4 | 2 | 1 | 1 | 1 | 3 | 0 | 2 | 0 | 2 | 3 | 13 | 1 | 3 | 0 | 1 | 0 | 0 | 1 |
| MOYY | 1 | 2 | 8 | 5 | 4 | 1 | 1 | 1 | 2 | 3 | 2 | 1 | 2 | 1 | 5 | 0 | 2 | 0 | 2 | 1 | 16 | 1 | 1 | 1 | 1 | 0 | 0 | 1 |
| STKJ | 1 | 2 | 6 | 4 | 5 | 1 | 3 | 0 | 1 | 5 | 2 | 1 | 1 | 2 | 2 | 0 | 1 | 0 | 2 | 4 | 21 | 1 | 1 | 1 | 0 | 1 | 1 | 1 |
| WDGV | 1 | 3 | 9 | 5 | 4 | 2 | 1 | 1 | 3 | 7 | 2 | 1 | 2 | 0 | 7 | 0 | 2 | 0 | 4 | 3 | 30 | 1 | 2 | 1 | 2 | 2 | 2 | 1 |
| MXEZ | 0 | 1 | 3 | 3 | 4 | 2 | 1 | 2 | 4 | 6 | 0 | 0 | 2 | 4 | 1 | 0 | 1 | 0 | 3 | 2 | 28 | 1 | 1 | 1 | 1 | 4 | 2 | 1 |
| MJMQ | 1 | 3 | 1 | 1 | 0 | 0 | 0 | 2 | 0 | 0 | 0 | 0 | 1 | 0 | 4 | 0 | 0 | 0 | 0 | 1 | 7 | 0 | 0 | 0 | 0 | 2 | 1 | 0 |
| YTYU | 0 | 2 | 3 | 2 | 1 | 0 | 1 | 1 | 1 | 6 | 1 | 0 | 1 | 2 | 1 | 0 | 1 | 0 | 2 | 1 | 17 | 2 | 0 | 1 | 0 | 2 | 2 | 1 |
| DXNY | 2 | 1 | 6 | 6 | 5 | 2 | 2 | 3 | 4 | 8 | 2 | 1 | 2 | 4 | 2 | 0 | 2 | 0 | 2 | 2 | 37 | 2 | 1 | 1 | 0 | 1 | 1 | 1 |
| AGIO | 1 | 1 | 5 | 3 | 2 | 3 | 1 | 2 | 0 | 4 | 1 | 1 | 3 | 2 | 2 | 0 | 0 | 0 | 0 | 2 | 23 | 2 | 3 | 0 | 0 | 2 | 0 | 1 |
| ODXI | 1 | 1 | 6 | 5 | 4 | 3 | 2 | 2 | 1 | 4 | 0 | 1 | 2 | 4 | 5 | 0 | 0 | 0 | 3 | 1 | 33 | 3 | 2 | 0 | 0 | 2 | 1 | 1 |
| NTLE | 0 | 1 | 2 | 1 | 2 | 2 | 1 | 0 | 0 | 4 | 0 | 0 | 2 | 2 | 3 | 0 | 1 | 0 | 2 | 2 | 21 | 2 | 3 | 1 | 0 | 2 | 1 | 1 |
| HJVM | 1 | 2 | 6 | 5 | 5 | 2 | 3 | 0 | 1 | 4 | 2 | 2 | 1 | 3 | 3 | 0 | 2 | 0 | 2 | 4 | 16 | 1 | 1 | 1 | 0 | 1 | 1 | 0 |

| sample code | ec1.3.3.6 | ec1.3.99.3 | ec2.3.1.16 | ec2.3.1.179 | ec2.3.1.180 | ec2.3.1.181 | ec2.3.1.39 | ec3.1.2.14 | ec3.1.2.20 | ec3.1.2.22 | ec4.2.1.17 | ec4.2.1.60 | ec6.2.1.3 | ec6.4.1.2 | ec2.1.1.103 | 4.1.9 | ec2.7.1.32 | ec2.7.1.82 | ec2.7.7.14 | ec2.7.7.15 | ec2.7.8.1 | ec2.7.8.11 | ec2.7.8.2 | ec2.7.8.24 | ec3.1.3.75 | ec3.13.1.1 |
|---|---|---|---|---|---|---|---|---|---|---|---|---|---|---|---|---|---|---|---|---|---|---|---|---|---|---|
| ISGT | 5 | 8 | 2 | 2 | 0 | 2 | 1 | 0 | 1 | 1 | 7 | 0 | 7 | 3 | 1 | 2 | 2 | 2 | 2 | 2 | 1 | 1 | 1 | 0 | 1 | 1 |
| RAWF | 5 | 9 | 2 | 2 | 1 | 2 | 1 | 0 | 2 | 1 | 8 | 0 | 6 | 6 | 1 | 3 | 2 | 3 | 2 | 2 | 1 | 1 | 1 | 0 | 2 | 1 |
| IJMT | 10 | 21 | 7 | 4 | 1 | 2 | 2 | 0 | 2 | 3 | 20 | 0 | 19 | 9 | 1 | 7 | 2 | 2 | 4 | 4 | 4 | 2 | 3 | 0 | 1 | 1 |
| SDPC | 4 | 7 | 1 | 2 | 1 | 1 | 1 | 1 | 0 | 1 | 6 | 0 | 10 | 4 | 1 | 2 | 0 | 0 | 2 | 2 | 0 | 1 | 0 | 0 | 1 | 1 |
| DVYE | 3 | 7 | 2 | 3 | 1 | 2 | 1 | 0 | 0 | 1 | 6 | 0 | 8 | 3 | 1 | 4 | 1 | 1 | 2 | 2 | 0 | 1 | 0 | 0 | 1 | 1 |
| BAZF | 5 | 8 | 2 | 3 | 2 | 2 | 2 | 2 | 1 | 1 | 8 | 0 | 17 | 5 | 0 | 3 | 2 | 2 | 2 | 1 | 1 | 1 | 1 | 0 | 1 | 1 |
| JTIG | 3 | 5 | 2 | 1 | 1 | 2 | 1 | 1 | 2 | 2 | 6 | 0 | 10 | 4 | 1 | 4 | 2 | 2 | 2 | 1 | 1 | 1 | 1 | 0 | 1 | 1 |
| GYBH | 3 | 5 | 2 | 3 | 1 | 2 | 1 | 2 | 1 | 1 | 8 | 0 | 11 | 7 | 1 | 4 | 1 | 1 | 2 | 2 | 1 | 1 | 1 | 0 | 1 | 1 |
| ETGN | 2 | 0 | 3 | 2 | 0 | 2 | 0 | 0 | 0 | 1 | 3 | 0 | 4 | 0 | 1 | 2 | 0 | 0 | 1 | 1 | 0 | 1 | 0 | 0 | 0 | 0 |
| QYXY | 5 | 10 | 2 | 6 | 4 | 2 | 3 | 0 | 1 | 1 | 8 | 0 | 14 | 7 | 1 | 3 | 2 | 2 | 2 | 2 | 1 | 1 | 1 | 0 | 2 | 1 |
| GXBM | 5 | 8 | 2 | 6 | 2 | 2 | 2 | 2 | 1 | 1 | 8 | 0 | 13 | 6 | 1 | 3 | 2 | 2 | 2 | 2 | 1 | 1 | 1 | 0 | 1 | 1 |
| NKXU | 5 | 8 | 2 | 3 | 2 | 2 | 2 | 1 | 2 | 1 | 8 | 0 | 15 | 6 | 1 | 3 | 2 | 2 | 2 | 2 | 1 | 1 | 1 | 0 | 0 | 1 |
| ZNUM | 4 | 8 | 2 | 3 | 2 | 1 | 3 | 1 | 2 | 1 | 8 | 0 | 11 | 7 | 1 | 3 | 2 | 2 | 2 | 2 | 1 | 1 | 1 | 0 | 1 | 1 |
| WXRI | 5 | 5 | 1 | 2 | 2 | 2 | 1 | 2 | 0 | 1 | 2 | 0 | 6 | 4 | 1 | 1 | 0 | 1 | 2 | 2 | 1 | 1 | 1 | 0 | 0 | 1 |
| WCLV | 5 | 6 | 1 | 2 | 1 | 2 | 1 | 1 | 1 | 1 | 5 | 0 | 9 | 5 | 1 | 2 | 2 | 2 | 2 | 2 | 1 | 1 | 1 | 0 | 1 | 1 |
| EEJO | 5 | 9 | 2 | 3 | 1 | 2 | 1 | 2 | 1 | 1 | 9 | 0 | 14 | 8 | 2 | 3 | 1 | 1 | 2 | 2 | 1 | 1 | 1 | 0 | 2 | 1 |
| AKCR | 6 | 8 | 3 | 3 | 1 | 2 | 1 | 2 | 1 | 1 | 9 | 0 | 17 | 5 | 1 | 3 | 2 | 2 | 2 | 2 | 1 | 1 | 1 | 0 | 1 | 1 |
| BILC | 5 | 8 | 2 | 3 | 2 | 2 | 2 | 2 | 2 | 1 | 7 | 0 | 8 | 6 | 1 | 3 | 2 | 2 | 2 | 2 | 0 | 1 | 0 | 0 | 0 | 1 |
| JQFK | 4 | 8 | 4 | 6 | 3 | 2 | 4 | 2 | 0 | 3 | 13 | 1 | 12 | 7 | 2 | 3 | 2 | 2 | 2 | 2 | 1 | 1 | 1 | 0 | 1 | 1 |
| MNCB | 5 | 5 | 3 | 2 | 1 | 2 | 1 | 0 | 2 | 1 | 8 | 0 | 7 | 4 | 0 | 2 | 2 | 2 | 2 | 2 | 1 | 1 | 0 | 0 | 0 | 1 |
| MFYC | 5 | 7 | 2 | 3 | 2 | 2 | 1 | 2 | 2 | 1 | 6 | 0 | 5 | 5 | 1 | 3 | 2 | 1 | 2 | 2 | 1 | 1 | 1 | 0 | 0 | 1 |
| HHXJ | 2 | 7 | 1 | 2 | 1 | 2 | 1 | 0 | 0 | 1 | 8 | 0 | 7 | 4 | 0 | 2 | 1 | 1 | 2 | 2 | 1 | 1 | 1 | 0 | 0 | 1 |
| HVNO | 2 | 7 | 2 | 3 | 1 | 1 | 1 | 1 | 1 | 1 | 7 | 0 | 8 | 2 | 1 | 4 | 1 | 1 | 2 | 1 | 0 | 1 | 0 | 0 | 1 | 1 |
| DUMA | 3 | 9 | 3 | 3 | 1 | 1 | 2 | 1 | 0 | 1 | 7 | 0 | 7 | 5 | 0 | 2 | 1 | 1 | 1 | 1 | 0 | 1 | 0 | 0 | 1 | 1 |
| FMVB | 3 | 9 | 2 | 3 | 1 | 1 | 1 | 1 | 2 | 1 | 7 | 0 | 9 | 5 | 1 | 4 | 1 | 1 | 2 | 2 | 1 | 1 | 1 | 0 | 1 | 1 |
| PUAN | 3 | 4 | 1 | 3 | 1 | 2 | 1 | 1 | 0 | 1 | 3 | 0 | 9 | 4 | 0 | 2 | 1 | 1 | 1 | 1 | 1 | 1 | 1 | 0 | 1 | 1 |
| RRSV | 3 | 4 | 1 | 3 | 0 | 0 | 1 | 1 | 0 | 1 | 2 | 0 | 4 | 4 | 0 | 2 | 0 | 0 | 1 | 1 | 0 | 1 | 0 | 0 | 1 | 1 |
| AYPS | 2 | 9 | 2 | 2 | 1 | 2 | 2 | 2 | 0 | 1 | 9 | 0 | 7 | 6 | 0 | 3 | 1 | 1 | 1 | 1 | 0 | 1 | 0 | 0 | 0 | 1 |
| TGNL | 2 | 2 | 1 | 3 | 1 | 2 | 2 | 2 | 0 | 1 | 5 | 0 | 8 | 3 | 1 | 2 | 2 | 2 | 2 | 2 | 1 | 1 | 1 | 0 | 0 | 1 |
| MMKU | 3 | 8 | 2 | 5 | 1 | 2 | 3 | 2 | 0 | 1 | 8 | 0 | 11 | 8 | 1 | 3 | 2 | 2 | 2 | 2 | 1 | 1 | 1 | 0 | 0 | 1 |
| ISIM | 3 | 9 | 2 | 4 | 1 | 1 | 1 | 2 | 1 | 1 | 7 | 0 | 5 | 4 | 0 | 2 | 2 | 1 | 2 | 1 | 2 | 1 | 2 | 0 | 1 | 1 |
| MCPK | 1 | 8 | 3 | 4 | 1 | 3 | 2 | 1 | 1 | 1 | 7 | 0 | 8 | 3 | 0 | 2 | 1 | 1 | 1 | 1 | 1 | 1 | 1 | 0 | 0 | 1 |
| QXSZ | 1 | 7 | 4 | 3 | 0 | 1 | 1 | 0 | 0 | 1 | 3 | 0 | 4 | 2 | 0 | 1 | 0 | 0 | 0 | 0 | 0 | 1 | 0 | 0 | 0 | 1 |
| BTFM | 2 | 6 | 1 | 3 | 1 | 2 | 1 | 1 | 0 | 1 | 5 | 0 | 6 | 2 | 1 | 2 | 1 | 1 | 3 | 2 | 1 | 1 | 1 | 0 | 0 | 1 |
| XOAL | 1 | 8 | 5 | 4 | 2 | 3 | 3 | 3 | 3 | 1 | 13 | 0 | 9 | 4 | 0 | 2 | 0 | 1 | 1 | 1 | 1 | 1 | 1 | 0 | 0 | 1 |
| TNAW | 3 | 4 | 2 | 2 | 1 | 1 | 2 | 3 | 0 | 1 | 7 | 0 | 10 | 3 | 0 | 2 | 0 | 0 | 2 | 1 | 0 | 1 | 0 | 0 | 0 | 1 |
| JMTE | 2 | 10 | 2 | 2 | 1 | 1 | 3 | 3 | 1 | 1 | 8 | 0 | 11 | 3 | 0 | 4 | 0 | 0 | 1 | 1 | 1 | 1 | 1 | 0 | 0 | 1 |
| EGNB | 4 | 4 | 1 | 3 | 1 | 0 | 1 | 1 | 0 | 2 | 4 | 0 | 5 | 3 | 0 | 2 | 0 | 0 | 0 | 0 | 1 | 0 | 0 | 0 | 0 | 1 |
| HYHN | 0 | 8 | 2 | 2 | 1 | 0 | 1 | 2 | 1 | 1 | 4 | 0 | 8 | 5 | 0 | 3 | 0 | 1 | 1 | 0 | 1 | 1 | 0 | 0 | 0 | 1 |
| XMCL | 1 | 7 | 2 | 1 | 0 | 1 | 0 | 0 | 1 | 1 | 8 | 0 | 7 | 1 | 0 | 2 | 1 | 1 | 0 | 0 | 0 | 1 | 0 | 0 | 0 | 1 |
| AZZW | 2 | 6 | 2 | 3 | 1 | 2 | 3 | 1 | 0 | 1 | 9 | 0 | 9 | 4 | 1 | 3 | 2 | 2 | 2 | 2 | 2 | 1 | 2 | 0 | 0 | 1 |
| NNHQ | 4 | 5 | 2 | 2 | 1 | 0 | 1 | 1 | 0 | 1 | 7 | 0 | 7 | 4 | 0 | 2 | 1 | 1 | 2 | 2 | 1 | 1 | 0 | 0 | 0 | 1 |
| KYIO | 3 | 5 | 3 | 2 | 1 | 1 | 2 | 2 | 0 | 1 | 7 | 0 | 7 | 1 | 0 | 2 | 0 | 0 | 2 | 2 | 1 | 0 | 1 | 0 | 0 | 1 |
| FQLP | 4 | 5 | 3 | 3 | 2 | 1 | 1 | 3 | 0 | 1 | 10 | 0 | 11 | 5 | 1 | 2 | 1 | 1 | 2 | 2 | 1 | 1 | 1 | 0 | 1 | 1 |
| FPCO | 4 | 7 | 3 | 3 | 1 | 2 | 1 | 3 | 1 | 1 | 11 | 0 | 17 | 6 | 1 | 3 | 2 | 2 | 2 | 2 | 1 | 1 | 1 | 0 | 1 | 1 |
| BFIK | 4 | 7 | 3 | 2 | 1 | 2 | 1 | 1 | 0 | 2 | 11 | 0 | 17 | 5 | 1 | 2 | 2 | 2 | 2 | 2 | 1 | 1 | 1 | 0 | 1 | 1 |
| MWXT | 1 | 1 | 0 | 4 | 1 | 1 | 1 | 0 | 0 | 1 | 4 | 0 | 4 | 2 | 1 | 1 | 0 | 1 | 1 | 1 | 0 | 1 | 0 | 0 | 0 | 1 |
| VQBJ | 4 | 7 | 3 | 2 | 1 | 1 | 1 | 1 | 1 | 1 | 8 | 0 | 8 | 3 | 1 | 2 | 2 | 2 | 2 | 2 | 1 | 1 | 1 | 0 | 1 | 1 |
| QPDY | 4 | 6 | 3 | 3 | 1 | 2 | 1 | 1 | 1 | 2 | 8 | 0 | 14 | 4 | 1 | 2 | 2 | 2 | 2 | 2 | 1 | 1 | 1 | 0 | 1 | 1 |
| DRGY | 2 | 4 | 3 | 4 | 1 | 0 | 1 | 1 | 0 | 1 | 8 | 0 | 8 | 2 | 0 | 2 | 1 | 1 | 1 | 1 | 1 | 1 | 1 | 0 | 1 | 1 |
| HAOX | 4 | 2 | 2 | 2 | 1 | 1 | 1 | 1 | 0 | 0 | 7 | 0 | 4 | 3 | 0 | 2 | 0 | 0 | 2 | 2 | 0 | 1 | 0 | 0 | 0 | 1 |
| WSJO | 5 | 7 | 3 | 3 | 1 | 1 | 1 | 2 | 1 | 1 | 9 | 0 | 13 | 5 | 0 | 3 | 2 | 2 | 2 | 2 | 1 | 1 | 1 | 0 | 0 | 1 |
| WDCW | 5 | 8 | 3 | 3 | 2 | 1 | 1 | 1 | 0 | 1 | 8 | 0 | 13 | 5 | 1 | 4 | 2 | 2 | 2 | 2 | 1 | 1 | 1 | 0 | 1 | 1 |
| YOXI | 4 | 7 | 4 | 3 | 1 | 2 | 1 | 1 | 0 | 1 | 10 | 0 | 14 | 5 | 0 | 3 | 0 | 0 | 1 | 1 | 1 | 1 | 1 | 0 | 1 | 1 |
| RPGL | 2 | 7 | 3 | 3 | 1 | 2 | 1 | 1 | 0 | 1 | 10 | 0 | 11 | 4 | 0 | 3 | 0 | 0 | 1 | 1 | 0 | 1 | 0 | 0 | 0 | 1 |
| HKZW | 3 | 6 | 3 | 4 | 1 | 2 | 1 | 1 | 0 | 1 | 8 | 0 | 14 | 4 | 1 | 2 | 1 | 1 | 2 | 2 | 1 | 1 | 1 | 0 | 0 | 1 |
| JOJQ | 4 | 7 | 3 | 3 | 1 | 2 | 1 | 1 | 1 | 1 | 8 | 0 | 15 | 5 | 1 | 3 | 2 | 2 | 2 | 2 | 1 | 1 | 0 | 0 | 0 | 1 |
| MFZO | 4 | 7 | 3 | 3 | 1 | 2 | 1 | 1 | 1 | 1 | 8 | 0 | 13 | 5 | 1 | 4 | 2 | 2 | 2 | 2 | 1 | 1 | 1 | 0 | 0 | 1 |
| FFGR | 3 | 6 | 3 | 3 | 1 | 0 | 1 | 2 | 1 | 1 | 7 | 0 | 8 | 4 | 0 | 3 | 1 | 1 | 1 | 0 | 1 | 1 | 0 | 0 | 0 | 1 |
| KMNX | 4 | 4 | 1 | 3 | 1 | 0 | 1 | 0 | 0 | 1 | 6 | 0 | 8 | 2 | 0 | 2 | 0 | 0 | 0 | 0 | 0 | 1 | 0 | 0 | 0 | 1 |
| SNOX | 4 | 5 | 2 | 3 | 1 | 2 | 1 | 1 | 0 | 1 | 9 | 0 | 12 | 4 | 0 | 3 | 1 | 1 | 1 | 1 | 0 | 1 | 0 | 0 | 1 | 1 |
| XRTZ | 3 | 6 | 3 | 3 | 2 | 2 | 1 | 1 | 0 | 1 | 7 | 0 | 12 | 4 | 0 | 3 | 0 | 0 | 2 | 2 | 0 | 1 | 0 | 0 | 0 | 1 |
| KEYW | 4 | 7 | 3 | 3 | 1 | 2 | 1 | 2 | 0 | 1 | 8 | 0 | 11 | 4 | 0 | 3 | 1 | 1 | 0 | 0 | 1 | 1 | 1 | 0 | 0 | 1 |
| DRFX | 3 | 4 | 4 | 3 | 1 | 1 | 1 | 1 | 0 | 0 | 7 | 0 | 9 | 2 | 0 | 1 | 0 | 0 | 0 | 1 | 1 | 1 | 0 | 0 | 0 | 1 |
| AEKF | 4 | 6 | 3 | 3 | 2 | 1 | 1 | 1 | 0 | 1 | 6 | 0 | 9 | 2 | 0 | 3 | 1 | 1 | 1 | 0 | 0 | 1 | 0 | 0 | 0 | 1 |
| YSQT | 5 | 6 | 3 | 3 | 1 | 2 | 1 | 1 | 0 | 2 | 8 | 0 | 13 | 5 | 0 | 4 | 1 | 1 | 1 | 1 | 0 | 1 | 0 | 0 | 0 | 1 |
| RPQV | 4 | 6 | 3 | 3 | 1 | 2 | 1 | 1 | 0 | 1 | 10 | 0 | 14 | 3 | 0 | 3 | 2 | 2 | 1 | 1 | 0 | 1 | 0 | 0 | 0 | 1 |
| HIDG | 2 | 4 | 1 | 2 | 1 | 2 | 1 | 1 | 0 | 1 | 4 | 0 | 6 | 2 | 0 | 2 | 1 | 1 | 1 | 0 | 0 | 1 | 0 | 0 | 0 | 1 |
| RQFE | 4 | 7 | 3 | 3 | 1 | 2 | 1 | 1 | 0 | 1 | 9 | 0 | 13 | 5 | 0 | 4 | 1 | 1 | 1 | 1 | 0 | 1 | 0 | 0 | 0 | 1 |
| GYRP | 3 | 6 | 3 | 3 | 1 | 1 | 1 | 0 | 0 | 1 | 6 | 0 | 9 | 2 | 0 | 4 | 1 | 1 | 1 | 0 | 1 | 1 | 1 | 0 | 0 | 1 |
| MNNM | 4 | 7 | 3 | 3 | 1 | 0 | 1 | 1 | 0 | 1 | 7 | 0 | 9 | 4 | 0 | 3 | 1 | 1 | 0 | 0 | 0 | 1 | 0 | 0 | 0 | 1 |
| ISHC | 5 | 7 | 3 | 3 | 2 | 2 | 1 | 3 | 0 | 1 | 8 | 0 | 11 | 4 | 0 | 2 | 1 | 1 | 1 | 1 | 0 | 1 | 0 | 0 | 0 | 1 |
| QWFV | 5 | 5 | 4 | 2 | 2 | 1 | 2 | 1 | 0 | 1 | 8 | 0 | 8 | 3 | 0 | 2 | 0 | 1 | 1 | 1 | 0 | 1 | 0 | 0 | 0 | 1 |
| DFDS | 5 | 8 | 3 | 3 | 0 | 2 | 1 | 1 | 0 | 1 | 7 | 0 | 9 | 5 | 0 | 2 | 0 | 0 | 1 | 1 | 1 | 1 | 1 | 0 | 0 | 1 |
| GGWH | 4 | 8 | 3 | 5 | 2 | 2 | 1 | 2 | 0 | 1 | 7 | 0 | 15 | 4 | 0 | 3 | 1 | 1 | 2 | 1 | 1 | 1 | 1 | 0 | 0 | 1 |
| WCQU | 4 | 7 | 3 | 3 | 1 | 2 | 1 | 2 | 0 | 1 | 8 | 0 | 14 | 5 | 0 | 4 | 1 | 1 | 1 | 1 | 1 | 1 | 1 | 0 | 0 | 1 |
| BHBK | 4 | 8 | 3 | 4 | 1 | 2 | 1 | 2 | 1 | 1 | 10 | 0 | 12 | 5 | 0 | 2 | 1 | 1 | 1 | 1 | 1 | 1 | 1 | 0 | 0 | 1 |
| RPRU | 4 | 7 | 3 | 4 | 1 | 2 | 1 | 1 | 0 | 1 | 8 | 0 | 13 | 4 | 0 | 3 | 1 | 1 | 2 | 1 | 1 | 1 | 1 | 0 | 0 | 1 |
| MCHJ | 4 | 7 | 3 | 3 | 1 | 2 | 1 | 0 | 0 | 1 | 8 | 0 | 13 | 5 | 0 | 3 | 1 | 1 | 1 | 0 | 1 | 1 | 1 | 0 | 0 | 1 |
| GBGT | 3 | 4 | 3 | 3 | 2 | 2 | 1 | 1 | 0 | 1 | 7 | 0 | 10 | 2 | 0 | 3 | 1 | 1 | 0 | 0 | 1 | 0 | 0 | 0 | 0 | 1 |
| MOYY | 4 | 5 | 3 | 3 | 2 | 1 | 1 | 0 | 0 | 1 | 8 | 0 | 10 | 4 | 0 | 3 | 0 | 0 | 2 | 1 | 1 | 1 | 1 | 0 | 0 | 0 |
| STKJ | 4 | 4 | 1 | 4 | 1 | 1 | 1 | 0 | 0 | 1 | 7 | 0 | 11 | 2 | 0 | 3 | 1 | 1 | 1 | 1 | 1 | 1 | 0 | 0 | 0 | 1 |
| WDGV | 4 | 7 | 3 | 3 | 1 | 2 | 1 | 0 | 0 | 1 | 8 | 0 | 15 | 5 | 0 | 4 | 1 | 1 | 1 | 1 | 0 | 1 | 0 | 0 | 0 | 2 |
| MXEZ | 2 | 9 | 2 | 3 | 1 | 1 | 2 | 2 | 1 | 1 | 7 | 0 | 9 | 3 | 0 | 2 | 0 | 0 | 0 | 0 | 1 | 1 | 1 | 0 | 0 | 1 |
| MJMQ | 0 | 3 | 0 | 0 | 0 | 0 | 0 | 0 | 0 | 1 | 0 | 0 | 5 | 0 | 0 | 0 | 0 | 0 | 0 | 0 | 0 | 0 | 0 | 0 | 0 | 0 |
| YTYU | 3 | 3 | 2 | 2 | 0 | 1 | 1 | 0 | 1 | 0 | 3 | 0 | 6 | 1 | 0 | 2 | 1 | 1 | 1 | 2 | 0 | 1 | 0 | 0 | 0 | 1 |
| DXNY | 5 | 8 | 2 | 2 | 1 | 2 | 1 | 1 | 0 | 1 | 7 | 0 | 9 | 5 | 1 | 4 | 1 | 1 | 2 | 2 | 1 | 1 | 1 | 0 | 1 | 1 |
| AGIO | 4 | 6 | 2 | 3 | 1 | 2 | 1 | 1 | 0 | 1 | 5 | 0 | 7 | 4 | 1 | 4 | 0 | 1 | 2 | 2 | 0 | 1 | 0 | 0 | 0 | 1 |
| ODXI | 5 | 6 | 2 | 3 | 1 | 2 | 2 | 1 | 2 | 1 | 6 | 0 | 10 | 6 | 1 | 3 | 1 | 1 | 2 | 2 | 0 | 1 | 0 | 0 | 1 | 1 |
| NTLE | 4 | 4 | 1 | 2 | 1 | 1 | 2 | 1 | 0 | 1 | 5 | 0 | 10 | 4 | 0 | 3 | 0 | 1 | 3 | 2 | 0 | 1 | 0 | 0 | 1 | 1 |
| HJVM | 4 | 4 | 4 | 3 | 1 | 1 | 1 | 0 | 0 | 1 | 7 | 0 | 10 | 5 | 0 | 3 | 1 | 1 | 1 | 1 | 1 | 1 | 0 | 0 | 0 | 1 |

# Supplementary Table S3 (pg 4/4, lower right quadrant)

| sample code | PF04191 | PF11899 | ec2.4.1.241 | ec2.4.1.46 | ec2.3.1.158 | ec2.3.1.20 | ec3.1.1.3 | PF03982 | ec2.5.1.1 | ec2.5.1.10 | ec2.5.1.29 | ec2.5.1.32 | ec5.3.3.2 | ec1.1.1.267 | ec1.17.1.2 | ec1.17.7.1 | ec2.2.1.7 | ec2.7.1.148 | ec2.7.7.60 | ec4.6.1.12 | ec1.1.1.34 | ec1.1.1.88 | ec2.3.3.10 | ec2.7.1.36 | ec2.7.4.2 | ec4.1.1.33 | ec2.1.1.14 | PF00809 | PF01717 | PF02310 | PF02574 | PF02607 | PF02965 | PF08267 | ec1.1.1.65 | ec1.2.1.72 | ec1.4.3.5 | ec2.3.1.47 | ec2.6.1.52 | ec2.7.1.35 | ec3.1.3.74 | ec4.2.3.1 | PF01174 | ec2.6.1.62 | ec2.8.1.6 | ec6.2.1.14 | ec6.3.3.3 | ec6.3.4.10 | ec6.3.4.11 | ec6.3.4.15 | ec6.3.4.9 | ec1.4.3.19 | ec2.5.1.3 | ec2.7.1.49 | ec2.7.1.50 | ec2.7.4.16 | ec2.7.4.7 | ec2.7.6.2 | ec2.8.1.7 | ec3.5.99.2 | ec3.6.1.15 | ec3.6.1.28 | PF00899 | PF01946 | PF01964 | PF02110 | PF00636 | PF02170 | PF02171 | PF03368 | PF05183 | PF02301 | PF03962 | PF07106 | SPO11 | PF00078 | PF00270 | PF00271 | PF00493 | PF02889 | PF08241 | PF08423 | PF13489 |
|---|---|---|---|---|---|---|---|---|---|---|---|---|---|---|---|---|---|---|---|---|---|---|---|---|---|---|---|---|---|---|---|---|---|---|---|---|---|---|---|---|---|---|---|---|---|---|---|---|---|---|---|---|---|---|---|---|---|---|---|---|---|---|---|---|---|---|---|---|---|---|---|---|---|---|---|---|---|---|---|---|---|---|---|
| ISGT | 0 | 1 | 1 | 0 | 0 | 1 | 3 | 1 | 3 | 3 | 2 | 1 | 1 | 1 | 1 | 1 | 1 | 1 | 1 | 1 | 0 | 0 | 0 | 0 | 0 | 0 | 0 | 1 | 0 | 2 | 1 | 0 | 2 | 0 | 4 | 2 | 1 | 2 | 1 | 0 | 2 | 1 | 0 | 1 | 0 | 0 | 0 | 0 | 0 | 0 | 2 | 0 | 0 | 1 | 0 | 0 | 0 | 1 | 0 | 1 | 0 | 7 | 0 | 2 | 2 | 1 | 1 | 2 | 3 | 0 | 1 | 0 | 0 | 0 | 1 | 10 | 13 | 25 | 0 | 0 | 25 | 1 | 13 |
| RAWF | 0 | 1 | 2 | 0 | 0 | 1 | 11 | 1 | 3 | 3 | 2 | 2 | 1 | 1 | 1 | 1 | 1 | 1 | 1 | 1 | 0 | 0 | 1 | 1 | 0 | 0 | 1 | 3 | 1 | 2 | 2 | 1 | 2 | 1 | 7 | 2 | 1 | 2 | 1 | 1 | 0 | 1 | 0 | 2 | 0 | 1 | 0 | 0 | 1 | 0 | 2 | 2 | 0 | 1 | 0 | 0 | 1 | 3 | 1 | 1 | 0 | 8 | 1 | 1 | 1 | 1 | 2 | 4 | 3 | 0 | 0 | 1 | 0 | 0 | 1 | 15 | 33 | 65 | 3 | 0 | 56 | 2 | 29 |
| IJMT | 1 | 1 | 1 | 1 | 0 | 3 | 18 | 1 | 8 | 7 | 7 | 3 | 3 | 1 | 1 | 1 | 1 | 1 | 0 | 1 | 1 | 0 | 1 | 2 | 1 | 1 | 0 | 3 | 0 | 4 | 2 | 1 | 3 | 0 | 8 | 3 | 1 | 7 | 2 | 4 | 2 | 2 | 1 | 1 | 0 | 1 | 0 | 0 | 0 | 0 | 1 | 2 | 2 | 2 | 0 | 2 | 2 | 4 | 3 | 2 | 1 | 19 | 1 | 1 | 1 | 2 | 7 | 6 | 5 | 1 | 3 | 1 | 0 | 1 | 1 | 5 | 54 | 115 | 6 | 0 | 78 | 1 | 45 |
| SDPC | 0 | 1 | 1 | 1 | 0 | 0 | 5 | 1 | 4 | 4 | 3 | 2 | 1 | 1 | 1 | 1 | 1 | 1 | 1 | 1 | 0 | 0 | 0 | 1 | 0 | 0 | 0 | 2 | 0 | 1 | 2 | 1 | 1 | 0 | 5 | 1 | 0 | 3 | 1 | 1 | 0 | 1 | 1 | 1 | 0 | 0 | 1 | 1 | 1 | 1 | 0 | 0 | 0 | 1 | 0 | 0 | 2 | 2 | 0 | 1 | 0 | 6 | 1 | 1 | 1 | 1 | 0 | 3 | 3 | 0 | 0 | 1 | 1 | 2 | 1 | 7 | 25 | 50 | 4 | 0 | 41 | 1 | 22 |
| DVYE | 0 | 1 | 0 | 1 | 1 | 0 | 6 | 1 | 4 | 3 | 3 | 2 | 1 | 1 | 1 | 1 | 1 | 1 | 1 | 1 | 0 | 0 | 0 | 1 | 0 | 0 | 0 | 3 | 0 | 2 | 1 | 1 | 2 | 0 | 6 | 2 | 1 | 3 | 1 | 2 | 1 | 1 | 1 | 1 | 0 | 1 | 0 | 0 | 1 | 0 | 1 | 1 | 1 | 1 | 0 | 1 | 1 | 2 | 0 | 1 | 0 | 7 | 1 | 1 | 1 | 1 | 2 | 3 | 4 | 1 | 0 | 0 | 0 | 1 | 1 | 9 | 34 | 61 | 7 | 0 | 50 | 0 | 32 |
| BAZF | 0 | 1 | 2 | 1 | 0 | 1 | 12 | 2 | 4 | 3 | 3 | 1 | 1 | 1 | 1 | 1 | 1 | 1 | 1 | 1 | 0 | 0 | 0 | 1 | 0 | 0 | 2 | 1 | 1 | 2 | 2 | 1 | 1 | 0 | 8 | 2 | 1 | 3 | 1 | 1 | 1 | 1 | 0 | 2 | 0 | 0 | 0 | 0 | 1 | 0 | 1 | 1 | 0 | 1 | 0 | 0 | 1 | 2 | 0 | 1 | 0 | 8 | 1 | 2 | 1 | 1 | 1 | 6 | 6 | 0 | 0 | 2 | 1 | 0 | 1 | 5 | 33 | 75 | 6 | 0 | 62 | 2 | 33 |
| JTIG | 0 | 1 | 2 | 1 | 0 | 1 | 13 | 3 | 4 | 4 | 3 | 1 | 2 | 1 | 1 | 1 | 1 | 1 | 1 | 1 | 0 | 0 | 0 | 1 | 0 | 0 | 1 | 0 | 1 | 1 | 1 | 1 | 1 | 1 | 10 | 2 | 1 | 4 | 1 | 2 | 1 | 1 | 1 | 1 | 1 | 1 | 0 | 0 | 1 | 0 | 0 | 3 | 1 | 1 | 0 | 1 | 1 | 3 | 2 | 1 | 0 | 7 | 1 | 1 | 1 | 1 | 2 | 2 | 4 | 0 | 0 | 1 | 1 | 1 | 0 | 4 | 41 | 70 | 3 | 0 | 64 | 3 | 40 |
| GYBH | 0 | 1 | 2 | 1 | 0 | 1 | 15 | 3 | 5 | 5 | 4 | 2 | 1 | 1 | 1 | 1 | 1 | 1 | 1 | 1 | 0 | 0 | 1 | 1 | 0 | 0 | 1 | 2 | 1 | 0 | 1 | 0 | 0 | 1 | 9 | 3 | 1 | 4 | 2 | 2 | 1 | 1 | 1 | 1 | 0 | 1 | 1 | 1 | 1 | 1 | 0 | 3 | 1 | 1 | 0 | 1 | 1 | 4 | 1 | 1 | 0 | 6 | 1 | 1 | 1 | 1 | 2 | 3 | 3 | 1 | 1 | 1 | 1 | 1 | 0 | 9 | 52 | 81 | 8 | 0 | 44 | 3 | 31 |
| ETGN | 0 | 0 | 0 | 0 | 0 | 0 | 2 | 1 | 2 | 2 | 1 | 0 | 1 | 0 | 1 | 0 | 1 | 0 | 1 | 1 | 0 | 0 | 1 | 0 | 0 | 0 | 2 | 2 | 1 | 2 | 1 | 0 | 2 | 0 | 3 | 3 | 0 | 2 | 1 | 1 | 0 | 0 | 0 | 1 | 0 | 0 | 0 | 0 | 0 | 0 | 0 | 0 | 0 | 0 | 0 | 0 | 0 | 1 | 1 | 1 | 0 | 1 | 1 | 1 | 0 | 0 | 2 | 0 | 2 | 0 | 0 | 1 | 0 | 0 | 2 | 6 | 4 | 16 | 0 | 0 | 26 | 1 | 16 |
| QYXY | 0 | 1 | 2 | 1 | 1 | 1 | 10 | 4 | 4 | 4 | 3 | 1 | 1 | 1 | 1 | 1 | 1 | 1 | 1 | 1 | 0 | 0 | 0 | 1 | 0 | 0 | 1 | 2 | 1 | 2 | 3 | 1 | 1 | 1 | 5 | 2 | 1 | 3 | 1 | 1 | 0 | 1 | 0 | 1 | 0 | 0 | 1 | 1 | 1 | 1 | 3 | 0 | 0 | 1 | 1 | 0 | 1 | 3 | 1 | 1 | 0 | 7 | 0 | 1 | 0 | 1 | 3 | 3 | 3 | 1 | 1 | 2 | 1 | 0 | 1 | 15 | 44 | 83 | 6 | 0 | 64 | 2 | 43 |
| GXBM | 0 | 1 | 1 | 1 | 1 | 1 | 17 | 3 | 4 | 4 | 3 | 2 | 2 | 1 | 1 | 1 | 1 | 1 | 1 | 1 | 0 | 0 | 0 | 1 | 0 | 0 | 2 | 2 | 2 | 0 | 0 | 0 | 0 | 2 | 8 | 2 | 1 | 5 | 1 | 2 | 1 | 1 | 1 | 1 | 0 | 1 | 1 | 1 | 1 | 1 | 1 | 2 | 1 | 1 | 0 | 1 | 1 | 3 | 1 | 1 | 0 | 8 | 1 | 1 | 1 | 1 | 3 | 1 | 1 | 0 | 0 | 2 | 1 | 1 | 1 | 1 | 48 | 93 | 6 | 0 | 64 | 2 | 49 |
| NKXU | 0 | 1 | 2 | 1 | 1 | 1 | 16 | 4 | 4 | 4 | 3 | 2 | 2 | 1 | 1 | 1 | 1 | 1 | 1 | 1 | 0 | 0 | 1 | 1 | 0 | 0 | 1 | 2 | 1 | 1 | 2 | 1 | 2 | 1 | 8 | 3 | 1 | 3 | 2 | 2 | 1 | 1 | 0 | 1 | 0 | 1 | 1 | 1 | 1 | 1 | 4 | 3 | 1 | 1 | 0 | 1 | 1 | 4 | 2 | 1 | 0 | 7 | 1 | 1 | 1 | 1 | 7 | 2 | 1 | 1 | 0 | 2 | 1 | 1 | 2 | 3 | 57 | 98 | 7 | 0 | 76 | 3 | 44 |
| ZNUM | 0 | 1 | 2 | 1 | 1 | 1 | 15 | 3 | 4 | 4 | 3 | 1 | 1 | 1 | 1 | 1 | 1 | 1 | 1 | 1 | 0 | 0 | 1 | 1 | 0 | 0 | 1 | 1 | 1 | 2 | 2 | 1 | 2 | 0 | 12 | 4 | 1 | 4 | 1 | 2 | 0 | 1 | 1 | 1 | 0 | 1 | 1 | 1 | 1 | 1 | 1 | 3 | 1 | 1 | 0 | 1 | 1 | 2 | 1 | 1 | 0 | 7 | 1 | 1 | 1 | 1 | 3 | 1 | 1 | 0 | 0 | 1 | 1 | 0 | 2 | 3 | 42 | 83 | 3 | 0 | 68 | 4 | 51 |
| WXRI | 0 | 0 | 1 | 1 | 0 | 1 | 3 | 1 | 5 | 3 | 3 | 1 | 1 | 1 | 1 | 1 | 1 | 0 | 0 | 1 | 0 | 0 | 1 | 1 | 0 | 0 | 0 | 3 | 0 | 1 | 1 | 1 | 1 | 0 | 6 | 2 | 0 | 1 | 1 | 1 | 0 | 1 | 0 | 1 | 0 | 0 | 0 | 0 | 0 | 0 | 1 | 0 | 0 | 0 | 0 | 0 | 1 | 3 | 1 | 1 | 0 | 4 | 1 | 0 | 0 | 0 | 2 | 1 | 1 | 0 | 0 | 1 | 1 | 2 | 1 | 1 | 24 | 54 | 2 | 0 | 45 | 1 | 22 |
| WCLV | 0 | 0 | 1 | 1 | 1 | 1 | 12 | 2 | 4 | 4 | 3 | 2 | 1 | 1 | 1 | 1 | 1 | 1 | 1 | 1 | 0 | 0 | 1 | 1 | 0 | 0 | 1 | 3 | 1 | 1 | 1 | 1 | 1 | 1 | 4 | 2 | 0 | 3 | 1 | 2 | 0 | 1 | 1 | 1 | 0 | 1 | 1 | 1 | 1 | 1 | 1 | 2 | 1 | 1 | 0 | 1 | 1 | 3 | 1 | 1 | 0 | 7 | 1 | 1 | 0 | 1 | 4 | 1 | 1 | 1 | 0 | 2 | 1 | 2 | 1 | 1 | 49 | 85 | 7 | 0 | 51 | 1 | 33 |
| EEJO | 0 | 0 | 1 | 1 | 0 | 1 | 21 | 4 | 4 | 4 | 3 | 2 | 1 | 1 | 1 | 1 | 1 | 1 | 1 | 1 | 0 | 0 | 1 | 0 | 0 | 0 | 1 | 2 | 1 | 2 | 2 | 1 | 1 | 0 | 8 | 5 | 1 | 3 | 1 | 2 | 3 | 1 | 1 | 1 | 0 | 0 | 1 | 1 | 1 | 1 | 3 | 3 | 1 | 1 | 1 | 1 | 1 | 4 | 3 | 1 | 0 | 7 | 1 | 1 | 0 | 1 | 5 | 1 | 1 | 0 | 0 | 2 | 1 | 1 | 1 | 3 | 50 | 102 | 7 | 0 | 70 | 3 | 47 |
| AKCR | 0 | 1 | 1 | 1 | 0 | 1 | 13 | 5 | 4 | 4 | 4 | 2 | 2 | 1 | 1 | 1 | 1 | 1 | 1 | 1 | 0 | 0 | 1 | 1 | 0 | 0 | 1 | 2 | 1 | 1 | 2 | 1 | 1 | 1 | 9 | 3 | 1 | 3 | 1 | 1 | 2 | 1 | 1 | 2 | 0 | 0 | 0 | 0 | 1 | 0 | 2 | 3 | 1 | 1 | 0 | 1 | 1 | 4 | 3 | 1 | 0 | 7 | 1 | 1 | 0 | 1 | 3 | 1 | 1 | 1 | 0 | 0 | 0 | 0 | 1 | 4 | 44 | 89 | 8 | 0 | 72 | 3 | 46 |
| BILC | 0 | 0 | 0 | 1 | 0 | 1 | 5 | 1 | 4 | 3 | 3 | 1 | 3 | 1 | 1 | 1 | 1 | 1 | 1 | 1 | 0 | 0 | 1 | 1 | 0 | 0 | 2 | 2 | 1 | 2 | 2 | 1 | 1 | 1 | 3 | 2 | 1 | 3 | 1 | 1 | 1 | 1 | 0 | 1 | 0 | 0 | 0 | 0 | 1 | 0 | 1 | 1 | 1 | 1 | 0 | 1 | 1 | 2 | 4 | 1 | 0 | 8 | 1 | 1 | 0 | 1 | 0 | 0 | 0 | 0 | 0 | 2 | 1 | 1 | 1 | 0 | 36 | 71 | 4 | 0 | 27 | 2 | 12 |
| JQFK | 0 | 1 | 2 | 1 | 0 | 1 | 16 | 2 | 5 | 5 | 4 | 2 | 3 | 1 | 1 | 1 | 1 | 1 | 1 | 1 | 1 | 0 | 0 | 1 | 0 | 0 | 1 | 2 | 1 | 3 | 3 | 1 | 1 | 0 | 5 | 6 | 2 | 5 | 1 | 2 | 1 | 1 | 1 | 2 | 0 | 1 | 1 | 1 | 1 | 1 | 2 | 3 | 1 | 0 | 0 | 1 | 1 | 6 | 0 | 1 | 0 | 8 | 1 | 1 | 1 | 0 | 4 | 0 | 0 | 0 | 0 | 1 | 1 | 1 | 1 | 6 | 62 | 110 | 8 | 0 | 66 | 3 | 43 |
| MNCB | 0 | 0 | 0 | 1 | 1 | 1 | 1 | 2 | 4 | 4 | 3 | 3 | 2 | 1 | 1 | 0 | 1 | 1 | 1 | 1 | 0 | 0 | 1 | 0 | 0 | 0 | 0 | 2 | 0 | 1 | 1 | 1 | 1 | 0 | 6 | 3 | 2 | 3 | 2 | 2 | 1 | 1 | 0 | 1 | 0 | 0 | 1 | 1 | 1 | 1 | 1 | 0 | 0 | 0 | 0 | 0 | 1 | 3 | 0 | 1 | 0 | 4 | 1 | 0 | 0 | 0 | 2 | 4 | 3 | 0 | 1 | 0 | 0 | 1 | 1 | 3 | 49 | 81 | 2 | 0 | 46 | 0 | 24 |
| MFYC | 0 | 1 | 1 | 1 | 1 | 1 | 5 | 3 | 4 | 4 | 3 | 1 | 1 | 1 | 1 | 1 | 1 | 1 | 0 | 1 | 0 | 0 | 1 | 1 | 0 | 0 | 0 | 1 | 0 | 2 | 1 | 1 | 1 | 0 | 3 | 3 | 1 | 3 | 1 | 1 | 2 | 1 | 0 | 2 | 0 | 0 | 0 | 0 | 0 | 0 | 0 | 0 | 0 | 1 | 0 | 0 | 0 | 2 | 0 | 1 | 0 | 6 | 1 | 1 | 1 | 1 | 2 | 0 | 0 | 1 | 0 | 2 | 0 | 0 | 1 | 1 | 34 | 76 | 5 | 0 | 50 | 0 | 28 |
| HHXJ | 0 | 1 | 1 | 1 | 0 | 1 | 6 | 1 | 4 | 4 | 4 | 1 | 1 | 1 | 1 | 1 | 1 | 1 | 1 | 1 | 0 | 0 | 0 | 0 | 0 | 0 | 0 | 1 | 0 | 3 | 2 | 1 | 1 | 0 | 3 | 2 | 0 | 2 | 2 | 0 | 2 | 2 | 0 | 1 | 0 | 0 | 0 | 0 | 0 | 0 | 0 | 1 | 1 | 1 | 0 | 1 | 1 | 3 | 1 | 1 | 0 | 4 | 1 | 1 | 0 | 1 | 1 | 1 | 1 | 0 | 0 | 0 | 0 | 0 | 0 | 3 | 26 | 62 | 3 | 0 | 34 | 0 | 24 |
| HVNO | 0 | 1 | 1 | 3 | 1 | 1 | 8 | 1 | 2 | 2 | 2 | 2 | 1 | 1 | 1 | 1 | 0 | 0 | 0 | 1 | 0 | 0 | 0 | 1 | 0 | 0 | 1 | 1 | 0 | 2 | 2 | 2 | 1 | 0 | 4 | 3 | 1 | 4 | 2 | 2 | 1 | 2 | 0 | 2 | 0 | 0 | 1 | 1 | 1 | 1 | 1 | 1 | 1 | 1 | 0 | 0 | 1 | 3 | 0 | 0 | 0 | 5 | 1 | 0 | 1 | 0 | 2 | 2 | 2 | 1 | 0 | 1 | 1 | 0 | 0 | 1 | 32 | 85 | 2 | 0 | 67 | 1 | 43 |
| DUMA | 0 | 1 | 2 | 2 | 1 | 1 | 8 | 3 | 4 | 4 | 4 | 3 | 1 | 1 | 1 | 1 | 1 | 1 | 1 | 1 | 0 | 0 | 0 | 0 | 0 | 0 | 2 | 3 | 0 | 2 | 1 | 1 | 1 | 0 | 4 | 4 | 0 | 3 | 2 | 2 | 1 | 2 | 1 | 1 | 0 | 0 | 1 | 1 | 1 | 1 | 0 | 1 | 1 | 1 | 0 | 1 | 1 | 3 | 2 | 1 | 0 | 8 | 1 | 0 | 1 | 1 | 2 | 3 | 3 | 1 | 2 | 2 | 1 | 1 | 1 | 5 | 60 | 116 | 6 | 0 | 71 | 2 | 47 |
| FMVB | 0 | 1 | 2 | 1 | 1 | 1 | 6 | 0 | 4 | 4 | 3 | 2 | 1 | 1 | 1 | 1 | 1 | 1 | 1 | 1 | 0 | 0 | 0 | 1 | 0 | 0 | 0 | 1 | 0 | 2 | 2 | 1 | 2 | 0 | 7 | 2 | 0 | 4 | 1 | 1 | 1 | 1 | 0 | 2 | 0 | 0 | 1 | 1 | 1 | 1 | 0 | 2 | 0 | 1 | 0 | 0 | 0 | 3 | 0 | 1 | 0 | 7 | 1 | 1 | 0 | 1 | 2 | 3 | 3 | 0 | 1 | 1 | 0 | 0 | 1 | 4 | 37 | 81 | 7 | 0 | 56 | 0 | 38 |
| PUAN | 0 | 1 | 1 | 1 | 0 | 1 | 8 | 1 | 4 | 4 | 4 | 2 | 2 | 1 | 1 | 1 | 1 | 1 | 1 | 1 | 0 | 0 | 0 | 1 | 0 | 0 | 1 | 2 | 1 | 1 | 2 | 1 | 1 | 0 | 2 | 3 | 1 | 2 | 2 | 2 | 1 | 1 | 0 | 1 | 0 | 1 | 1 | 1 | 1 | 1 | 0 | 1 | 1 | 1 | 0 | 1 | 1 | 3 | 0 | 1 | 0 | 6 | 1 | 1 | 1 | 1 | 2 | 1 | 1 | 1 | 0 | 1 | 0 | 1 | 1 | 6 | 31 | 78 | 8 | 0 | 46 | 1 | 33 |
| RRSV | 0 | 1 | 1 | 1 | 0 | 1 | 6 | 1 | 3 | 3 | 3 | 1 | 0 | 1 | 2 | 1 | 1 | 1 | 1 | 1 | 0 | 0 | 0 | 1 | 0 | 0 | 1 | 1 | 1 | 1 | 1 | 1 | 1 | 1 | 3 | 4 | 0 | 2 | 1 | 2 | 0 | 1 | 0 | 1 | 0 | 0 | 1 | 1 | 1 | 1 | 0 | 1 | 1 | 0 | 0 | 1 | 0 | 2 | 0 | 1 | 0 | 2 | 1 | 1 | 1 | 0 | 1 | 2 | 2 | 0 | 0 | 0 | 0 | 0 | 1 | 5 | 26 | 48 | 3 | 0 | 26 | 0 | 23 |
| AYPS | 0 | 1 | 1 | 1 | 1 | 0 | 18 | 2 | 4 | 3 | 3 | 1 | 1 | 1 | 1 | 1 | 1 | 1 | 1 | 1 | 0 | 0 | 1 | 1 | 0 | 0 | 0 | 3 | 0 | 1 | 1 | 1 | 1 | 0 | 5 | 2 | 0 | 3 | 2 | 1 | 2 | 1 | 1 | 1 | 0 | 0 | 1 | 1 | 1 | 1 | 1 | 1 | 0 | 0 | 0 | 0 | 1 | 3 | 0 | 1 | 0 | 8 | 1 | 1 | 1 | 0 | 0 | 0 | 0 | 0 | 0 | 3 | 1 | 1 | 1 | 1 | 43 | 69 | 7 | 0 | 45 | 2 | 26 |
| TGNL | 0 | 1 | 1 | 1 | 1 | 1 | 6 | 3 | 4 | 4 | 3 | 2 | 2 | 1 | 1 | 1 | 1 | 1 | 1 | 1 | 0 | 0 | 0 | 1 | 0 | 1 | 1 | 2 | 1 | 1 | 2 | 1 | 1 | 1 | 6 | 6 | 0 | 2 | 1 | 1 | 0 | 1 | 1 | 1 | 0 | 1 | 1 | 1 | 1 | 1 | 0 | 2 | 1 | 1 | 1 | 1 | 1 | 3 | 2 | 1 | 0 | 7 | 1 | 0 | 0 | 1 | 3 | 1 | 1 | 0 | 1 | 2 | 1 | 2 | 0 | 2 | 39 | 77 | 9 | 0 | 55 | 5 | 32 |
| MMKU | 0 | 1 | 1 | 1 | 1 | 2 | 15 | 2 | 4 | 4 | 3 | 3 | 1 | 1 | 1 | 1 | 1 | 1 | 1 | 1 | 1 | 0 | 1 | 1 | 0 | 0 | 0 | 3 | 0 | 2 | 2 | 1 | 1 | 0 | 3 | 5 | 1 | 3 | 1 | 1 | 4 | 1 | 0 | 1 | 0 | 0 | 1 | 1 | 1 | 1 | 1 | 3 | 1 | 1 | 0 | 1 | 1 | 3 | 1 | 1 | 0 | 11 | 1 | 1 | 1 | 1 | 1 | 0 | 0 | 0 | 0 | 2 | 1 | 1 | 1 | 10 | 49 | 97 | 5 | 0 | 75 | 3 | 41 |
| ISIM | 0 | 0 | 1 | 1 | 1 | 1 | 5 | 1 | 4 | 4 | 3 | 2 | 2 | 1 | 1 | 1 | 1 | 1 | 1 | 1 | 1 | 0 | 0 | 1 | 0 | 0 | 0 | 1 | 0 | 2 | 1 | 1 | 1 | 0 | 6 | 1 | 0 | 2 | 1 | 1 | 3 | 1 | 0 | 1 | 0 | 0 | 1 | 1 | 1 | 1 | 2 | 0 | 0 | 0 | 0 | 0 | 1 | 3 | 0 | 1 | 0 | 8 | 1 | 0 | 0 | 0 | 1 | 0 | 0 | 0 | 0 | 1 | 0 | 1 | 1 | 1 | 33 | 70 | 3 | 0 | 74 | 1 | 41 |
| MCPK | 0 | 1 | 2 | 1 | 1 | 0 | 5 | 2 | 5 | 5 | 4 | 1 | 2 | 1 | 1 | 2 | 1 | 1 | 1 | 1 | 0 | 0 | 0 | 1 | 0 | 0 | 0 | 2 | 0 | 1 | 1 | 1 | 1 | 0 | 2 | 4 | 0 | 2 | 1 | 0 | 2 | 1 | 0 | 2 | 0 | 0 | 0 | 0 | 0 | 0 | 0 | 0 | 0 | 0 | 0 | 0 | 1 | 3 | 0 | 2 | 0 | 7 | 1 | 0 | 0 | 0 | 0 | 0 | 0 | 0 | 0 | 1 | 1 | 1 | 1 | 1 | 51 | 94 | 8 | 0 | 61 | 3 | 39 |
| QXSZ | 0 | 1 | 1 | 0 | 0 | 0 | 4 | 2 | 2 | 3 | 3 | 2 | 1 | 1 | 1 | 1 | 1 | 1 | 1 | 1 | 0 | 0 | 0 | 1 | 0 | 0 | 0 | 2 | 0 | 1 | 1 | 1 | 1 | 0 | 2 | 2 | 0 | 1 | 3 | 2 | 0 | 2 | 0 | 1 | 0 | 0 | 0 | 0 | 0 | 0 | 0 | 0 | 0 | 0 | 0 | 0 | 1 | 3 | 0 | 1 | 1 | 8 | 1 | 0 | 0 | 0 | 1 | 1 | 1 | 1 | 0 | 2 | 1 | 1 | 0 | 5 | 27 | 63 | 7 | 0 | 45 | 3 | 29 |
| BTFM | 0 | 0 | 1 | 1 | 0 | 1 | 8 | 1 | 4 | 4 | 3 | 1 | 1 | 1 | 1 | 1 | 1 | 1 | 1 | 1 | 0 | 0 | 0 | 0 | 0 | 0 | 0 | 3 | 0 | 1 | 2 | 1 | 1 | 0 | 8 | 2 | 0 | 3 | 1 | 1 | 2 | 1 | 0 | 1 | 0 | 0 | 0 | 0 | 0 | 0 | 0 | 1 | 0 | 0 | 0 | 0 | 1 | 3 | 0 | 1 | 0 | 5 | 1 | 1 | 0 | 0 | 2 | 5 | 5 | 1 | 0 | 1 | 0 | 1 | 1 | 5 | 39 | 73 | 6 | 0 | 64 | 1 | 34 |
| XOAL | 0 | 1 | 1 | 1 | 1 | 0 | 6 | 2 | 4 | 4 | 3 | 3 | 1 | 1 | 1 | 1 | 1 | 1 | 1 | 1 | 0 | 0 | 3 | 0 | 0 | 1 | 0 | 5 | 0 | 1 | 2 | 1 | 1 | 0 | 7 | 5 | 0 | 3 | 1 | 1 | 0 | 2 | 1 | 1 | 0 | 1 | 1 | 1 | 1 | 1 | 0 | 2 | 0 | 0 | 0 | 0 | 1 | 3 | 0 | 2 | 0 | 12 | 1 | 1 | 1 | 0 | 1 | 2 | 2 | 1 | 0 | 1 | 1 | 1 | 1 | 3 | 50 | 102 | 7 | 0 | 90 | 2 | 58 |
| TNAW | 0 | 1 | 2 | 1 | 1 | 1 | 7 | 2 | 4 | 4 | 2 | 2 | 1 | 1 | 1 | 1 | 1 | 1 | 1 | 2 | 0 | 0 | 1 | 0 | 0 | 0 | 1 | 1 | 1 | 2 | 1 | 1 | 1 | 0 | 5 | 5 | 0 | 3 | 1 | 1 | 0 | 1 | 0 | 1 | 0 | 0 | 1 | 1 | 1 | 1 | 1 | 1 | 1 | 1 | 0 | 1 | 1 | 3 | 1 | 1 | 1 | 7 | 1 | 1 | 1 | 1 | 2 | 2 | 2 | 0 | 2 | 2 | 1 | 0 | 1 | 2 | 29 | 82 | 6 | 0 | 82 | 2 | 50 |
| JMTE | 0 | 1 | 1 | 1 | 1 | 1 | 12 | 1 | 4 | 4 | 3 | 3 | 1 | 1 | 1 | 1 | 1 | 1 | 1 | 1 | 0 | 0 | 0 | 1 | 0 | 1 | 3 | 3 | 1 | 1 | 2 | 1 | 2 | 1 | 3 | 3 | 0 | 4 | 3 | 2 | 3 | 2 | 0 | 1 | 0 | 1 | 0 | 0 | 1 | 0 | 1 | 1 | 1 | 1 | 0 | 1 | 1 | 3 | 2 | 1 | 1 | 9 | 1 | 1 | 1 | 1 | 1 | 0 | 1 | 0 | 1 | 3 | 1 | 1 | 1 | 2 | 55 | 103 | 8 | 0 | 69 | 4 | 39 |
| EGNB | 0 | 1 | 0 | 1 | 0 | 1 | 3 | 0 | 4 | 4 | 3 | 1 | 1 | 1 | 2 | 1 | 1 | 1 | 1 | 1 | 1 | 0 | 1 | 0 | 0 | 0 | 0 | 1 | 0 | 1 | 1 | 1 | 1 | 0 | 2 | 1 | 0 | 2 | 1 | 1 | 1 | 1 | 0 | 1 | 0 | 0 | 0 | 0 | 0 | 0 | 0 | 1 | 1 | 0 | 0 | 0 | 0 | 2 | 0 | 1 | 0 | 5 | 1 | 1 | 1 | 0 | 0 | 2 | 1 | 0 | 0 | 0 | 0 | 0 | 1 | 7 | 31 | 66 | 2 | 0 | 37 | 0 | 23 |
| HYHN | 0 | 1 | 0 | 0 | 1 | 1 | 8 | 0 | 4 | 3 | 3 | 1 | 1 | 1 | 1 | 1 | 1 | 1 | 0 | 1 | 0 | 0 | 0 | 2 | 0 | 0 | 0 | 1 | 0 | 1 | 0 | 1 | 1 | 0 | 4 | 4 | 0 | 3 | 2 | 0 | 1 | 1 | 0 | 0 | 0 | 0 | 1 | 1 | 1 | 1 | 0 | 0 | 0 | 0 | 0 | 0 | 0 | 3 | 0 | 1 | 0 | 7 | 0 | 0 | 0 | 0 | 0 | 0 | 0 | 0 | 0 | 2 | 0 | 1 | 1 | 0 | 21 | 68 | 1 | 0 | 38 | 1 | 28 |
| XMCL | 0 | 1 | 1 | 1 | 0 | 1 | 3 | 0 | 4 | 4 | 3 | 2 | 1 | 0 | 1 | 1 | 1 | 2 | 0 | 1 | 0 | 0 | 0 | 1 | 0 | 0 | 0 | 1 | 0 | 1 | 2 | 0 | 1 | 0 | 6 | 3 | 0 | 2 | 1 | 1 | 0 | 3 | 0 | 0 | 0 | 0 | 0 | 0 | 0 | 0 | 1 | 1 | 0 | 1 | 1 | 0 | 1 | 3 | 0 | 1 | 0 | 10 | 0 | 0 | 0 | 1 | 0 | 0 | 0 | 0 | 0 | 1 | 1 | 0 | 2 | 0 | 38 | 76 | 6 | 0 | 63 | 2 | 42 |
| AZZW | 0 | 1 | 1 | 1 | 1 | 2 | 14 | 1 | 4 | 4 | 3 | 1 | 1 | 1 | 1 | 1 | 1 | 1 | 1 | 1 | 1 | 0 | 1 | 2 | 1 | 1 | 0 | 2 | 0 | 2 | 2 | 1 | 1 | 0 | 7 | 3 | 1 | 3 | 1 | 1 | 2 | 1 | 1 | 1 | 0 | 0 | 1 | 1 | 1 | 1 | 1 | 3 | 1 | 1 | 0 | 1 | 1 | 3 | 2 | 1 | 0 | 6 | 1 | 1 | 1 | 1 | 2 | 3 | 3 | 1 | 3 | 2 | 0 | 0 | 2 | 2 | 47 | 84 | 2 | 0 | 66 | 1 | 39 |
| NNHQ | 0 | 1 | 2 | 1 | 1 | 2 | 9 | 0 | 4 | 4 | 3 | 1 | 1 | 1 | 1 | 1 | 1 | 1 | 1 | 1 | 1 | 0 | 0 | 2 | 2 | 0 | 0 | 2 | 0 | 2 | 3 | 1 | 2 | 0 | 7 | 4 | 0 | 2 | 1 | 1 | 1 | 1 | 0 | 1 | 0 | 1 | 1 | 1 | 1 | 1 | 1 | 0 | 0 | 0 | 0 | 0 | 1 | 2 | 0 | 1 | 0 | 4 | 1 | 0 | 0 | 0 | 2 | 1 | 1 | 1 | 2 | 1 | 0 | 1 | 1 | 0 | 20 | 45 | 0 | 0 | 31 | 1 | 20 |
| KYIO | 0 | 0 | 0 | 0 | 0 | 1 | 4 | 0 | 3 | 3 | 3 | 1 | 1 | 1 | 1 | 1 | 1 | 0 | 1 | 1 | 1 | 0 | 1 | 0 | 0 | 0 | 0 | 0 | 0 | 2 | 1 | 0 | 0 | 0 | 5 | 3 | 0 | 3 | 1 | 1 | 1 | 1 | 0 | 1 | 0 | 0 | 1 | 1 | 1 | 1 | 0 | 0 | 0 | 0 | 0 | 0 | 1 | 4 | 0 | 1 | 0 | 2 | 1 | 1 | 0 | 0 | 1 | 5 | 4 | 0 | 0 | 2 | 0 | 1 | 1 | 0 | 19 | 29 | 0 | 0 | 33 | 1 | 19 |
| FQLP | 0 | 1 | 1 | 1 | 1 | 1 | 10 | 3 | 3 | 3 | 2 | 1 | 1 | 1 | 1 | 1 | 1 | 1 | 1 | 1 | 1 | 0 | 1 | 1 | 0 | 1 | 0 | 1 | 0 | 2 | 2 | 1 | 1 | 0 | 6 | 3 | 1 | 2 | 1 | 1 | 1 | 1 | 0 | 1 | 0 | 0 | 1 | 1 | 1 | 1 | 0 | 1 | 0 | 2 | 0 | 0 | 1 | 1 | 2 | 1 | 0 | 3 | 0 | 0 | 0 | 0 | 4 | 4 | 3 | 1 | 0 | 0 | 0 | 0 | 0 | 7 | 32 | 58 | 1 | 0 | 48 | 0 | 23 |
| FPCO | 0 | 1 | 2 | 1 | 1 | 1 | 19 | 4 | 4 | 4 | 3 | 2 | 1 | 1 | 1 | 1 | 1 | 1 | 1 | 1 | 1 | 0 | 1 | 3 | 1 | 1 | 1 | 3 | 1 | 2 | 2 | 1 | 1 | 1 | 13 | 4 | 1 | 4 | 1 | 2 | 2 | 1 | 2 | 2 | 0 | 1 | 1 | 1 | 1 | 1 | 2 | 2 | 1 | 1 | 1 | 1 | 1 | 4 | 1 | 1 | 0 | 9 | 1 | 1 | 1 | 1 | 3 | 4 | 3 | 1 | 1 | 2 | 1 | 0 | 1 | 5 | 56 | 107 | 6 | 0 | 73 | 3 | 51 |
| BFIK | 0 | 1 | 1 | 1 | 1 | 1 | 12 | 1 | 4 | 4 | 4 | 2 | 2 | 1 | 1 | 1 | 1 | 1 | 1 | 1 | 1 | 0 | 1 | 2 | 1 | 1 | 0 | 3 | 0 | 2 | 2 | 1 | 2 | 0 | 10 | 4 | 1 | 2 | 1 | 2 | 4 | 1 | 1 | 1 | 0 | 1 | 1 | 1 | 1 | 1 | 2 | 1 | 1 | 1 | 0 | 1 | 1 | 3 | 2 | 1 | 0 | 10 | 1 | 1 | 1 | 1 | 2 | 4 | 3 | 0 | 0 | 1 | 1 | 1 | 1 | 2 | 46 | 87 | 7 | 0 | 55 | 2 | 34 |
| MWXT | 0 | 1 | 1 | 1 | 0 | 0 | 2 | 0 | 4 | 3 | 3 | 2 | 1 | 1 | 1 | 1 | 2 | 1 | 1 | 1 | 1 | 0 | 1 | 0 | 0 | 1 | 0 | 1 | 0 | 2 | 1 | 1 | 1 | 0 | 2 | 4 | 0 | 1 | 1 | 1 | 1 | 1 | 0 | 1 | 0 | 0 | 0 | 0 | 0 | 0 | 0 | 1 | 1 | 0 | 0 | 1 | 1 | 2 | 0 | 0 | 0 | 5 | 0 | 0 | 1 | 0 | 0 | 0 | 1 | 0 | 1 | 0 | 0 | 0 | 0 | 3 | 16 | 22 | 0 | 0 | 30 | 0 | 11 |
| VQBJ | 0 | 1 | 1 | 1 | 0 | 1 | 7 | 0 | 3 | 3 | 2 | 2 | 2 | 1 | 1 | 1 | 1 | 1 | 1 | 1 | 1 | 0 | 1 | 1 | 0 | 1 | 1 | 3 | 1 | 2 | 2 | 1 | 2 | 0 | 6 | 3 | 1 | 2 | 1 | 2 | 0 | 1 | 0 | 1 | 0 | 0 | 1 | 1 | 1 | 1 | 0 | 1 | 1 | 0 | 0 | 1 | 1 | 4 | 0 | 1 | 0 | 8 | 1 | 1 | 1 | 0 | 4 | 3 | 2 | 0 | 0 | 1 | 1 | 1 | 1 | 20 | 40 | 71 | 6 | 0 | 54 | 1 | 26 |
| QPDY | 0 | 1 | 2 | 1 | 1 | 1 | 18 | 4 | 4 | 4 | 3 | 2 | 1 | 1 | 1 | 1 | 1 | 1 | 1 | 1 | 1 | 0 | 1 | 2 | 1 | 1 | 0 | 3 | 0 | 2 | 2 | 1 | 2 | 0 | 6 | 3 | 2 | 4 | 1 | 1 | 0 | 1 | 1 | 2 | 0 | 1 | 1 | 1 | 1 | 1 | 3 | 2 | 1 | 0 | 0 | 1 | 1 | 4 | 0 | 1 | 0 | 10 | 1 | 1 | 1 | 0 | 6 | 4 | 2 | 1 | 2 | 2 | 1 | 0 | 2 | 6 | 57 | 95 | 5 | 0 | 73 | 3 | 36 |
| DRGY | 0 | 1 | 1 | 1 | 0 | 1 | 1 | 1 | 3 | 3 | 2 | 1 | 1 | 1 | 1 | 1 | 1 | 1 | 1 | 1 | 1 | 0 | 1 | 1 | 0 | 1 | 0 | 1 | 0 | 1 | 1 | 1 | 1 | 0 | 5 | 4 | 1 | 2 | 1 | 1 | 1 | 1 | 0 | 1 | 0 | 0 | 0 | 0 | 0 | 0 | 0 | 0 | 0 | 0 | 0 | 0 | 0 | 2 | 0 | 1 | 0 | 5 | 1 | 1 | 0 | 0 | 1 | 2 | 2 | 0 | 0 | 2 | 1 | 0 | 1 | 2 | 24 | 37 | 5 | 0 | 47 | 2 | 22 |
| HAOX | 0 | 1 | 1 | 1 | 1 | 0 | 4 | 0 | 3 | 3 | 2 | 1 | 2 | 1 | 1 | 1 | 1 | 1 | 1 | 1 | 1 | 0 | 1 | 1 | 0 | 1 | 0 | 1 | 0 | 1 | 2 | 1 | 1 | 0 | 5 | 3 | 0 | 2 | 1 | 1 | 1 | 1 | 1 | 2 | 0 | 0 | 0 | 0 | 0 | 0 | 1 | 1 | 1 | 1 | 0 | 1 | 0 | 2 | 0 | 1 | 0 | 4 | 1 | 1 | 1 | 1 | 1 | 2 | 2 | 0 | 0 | 0 | 0 | 0 | 1 | 0 | 26 | 36 | 1 | 0 | 30 | 1 | 13 |
| WSJO | 0 | 1 | 1 | 1 | 1 | 1 | 11 | 1 | 4 | 4 | 3 | 2 | 1 | 1 | 1 | 1 | 1 | 1 | 1 | 1 | 1 | 0 | 0 | 2 | 0 | 1 | 1 | 2 | 1 | 3 | 2 | 1 | 1 | 1 | 8 | 4 | 1 | 4 | 1 | 1 | 0 | 1 | 0 | 1 | 0 | 0 | 1 | 1 | 1 | 1 | 0 | 0 | 1 | 1 | 1 | 1 | 1 | 4 | 0 | 1 | 0 | 9 | 1 | 1 | 1 | 1 | 3 | 4 | 3 | 1 | 0 | 2 | 1 | 1 | 2 | 0 | 46 | 83 | 2 | 0 | 69 | 3 | 33 |
| WDCW | 0 | 1 | 2 | 1 | 1 | 1 | 14 | 2 | 5 | 4 | 3 | 2 | 2 | 1 | 1 | 1 | 1 | 1 | 1 | 1 | 1 | 0 | 0 | 1 | 1 | 1 | 1 | 4 | 1 | 2 | 2 | 1 | 2 | 0 | 10 | 4 | 1 | 4 | 1 | 2 | 0 | 1 | 1 | 1 | 0 | 0 | 1 | 1 | 1 | 1 | 0 | 1 | 1 | 0 | 0 | 1 | 1 | 2 | 2 | 1 | 0 | 9 | 0 | 1 | 1 | 0 | 7 | 3 | 2 | 1 | 1 | 2 | 1 | 2 | 0 | 2 | 43 | 87 | 8 | 0 | 69 | 2 | 30 |
| YOXI | 0 | 1 | 1 | 2 | 1 | 1 | 16 | 1 | 3 | 3 | 3 | 2 | 2 | 1 | 1 | 1 | 1 | 1 | 1 | 1 | 1 | 0 | 1 | 2 | 1 | 1 | 1 | 4 | 1 | 2 | 2 | 1 | 2 | 1 | 9 | 4 | 1 | 3 | 1 | 2 | 0 | 1 | 1 | 1 | 0 | 1 | 1 | 1 | 1 | 1 | 0 | 2 | 1 | 1 | 0 | 1 | 1 | 2 | 1 | 1 | 0 | 7 | 0 | 1 | 1 | 1 | 4 | 5 | 4 | 2 | 0 | 1 | 1 | 0 | 1 | 4 | 50 | 93 | 7 | 0 | 65 | 4 | 37 |
| RPGL | 0 | 1 | 1 | 2 | 1 | 1 | 11 | 2 | 3 | 2 | 3 | 2 | 1 | 1 | 1 | 0 | 1 | 1 | 1 | 1 | 0 | 0 | 0 | 1 | 0 | 1 | 0 | 2 | 0 | 1 | 2 | 1 | 1 | 0 | 6 | 4 | 1 | 4 | 0 | 1 | 0 | 1 | 1 | 1 | 0 | 0 | 1 | 1 | 1 | 1 | 0 | 1 | 1 | 1 | 0 | 1 | 1 | 2 | 1 | 1 | 0 | 6 | 1 | 1 | 1 | 0 | 2 | 4 | 4 | 1 | 1 | 3 | 0 | 0 | 0 | 4 | 40 | 76 | 8 | 0 | 45 | 4 | 20 |
| HKZW | 0 | 1 | 2 | 2 | 1 | 1 | 12 | 2 | 3 | 3 | 2 | 3 | 1 | 1 | 1 | 1 | 1 | 1 | 1 | 1 | 1 | 0 | 1 | 2 | 1 | 1 | 2 | 2 | 0 | 2 | 2 | 1 | 1 | 1 | 4 | 5 | 1 | 3 | 1 | 2 | 1 | 1 | 1 | 1 | 0 | 1 | 1 | 1 | 1 | 1 | 1 | 2 | 1 | 1 | 0 | 1 | 1 | 3 | 1 | 1 | 0 | 9 | 1 | 1 | 1 | 1 | 4 | 2 | 2 | 1 | 1 | 2 | 1 | 0 | 1 | 1 | 51 | 93 | 7 | 0 | 67 | 2 | 34 |
| JOJQ | 0 | 1 | 2 | 2 | 1 | 1 | 12 | 2 | 4 | 3 | 3 | 2 | 1 | 1 | 1 | 1 | 1 | 1 | 1 | 1 | 1 | 0 | 0 | 2 | 0 | 1 | 1 | 2 | 1 | 2 | 2 | 1 | 1 | 1 | 7 | 4 | 0 | 4 | 1 | 2 | 1 | 1 | 1 | 1 | 0 | 1 | 1 | 1 | 1 | 1 | 3 | 1 | 1 | 1 | 1 | 1 | 1 | 3 | 1 | 1 | 0 | 9 | 1 | 1 | 1 | 1 | 5 | 4 | 2 | 1 | 1 | 2 | 0 | 0 | 2 | 3 | 51 | 95 | 2 | 0 | 72 | 5 | 38 |
| MFZO | 0 | 1 | 2 | 2 | 1 | 1 | 18 | 1 | 4 | 4 | 3 | 2 | 1 | 1 | 1 | 1 | 1 | 1 | 1 | 1 | 1 | 0 | 1 | 2 | 1 | 1 | 0 | 3 | 0 | 2 | 2 | 1 | 2 | 0 | 9 | 3 | 1 | 4 | 1 | 2 | 2 | 1 | 0 | 1 | 0 | 1 | 1 | 1 | 1 | 1 | 1 | 1 | 1 | 1 | 1 | 1 | 1 | 2 | 1 | 1 | 0 | 8 | 1 | 1 | 1 | 1 | 6 | 3 | 2 | 1 | 0 | 0 | 1 | 0 | 1 | 7 | 46 | 94 | 8 | 0 | 72 | 3 | 40 |
| FFGR | 0 | 1 | 1 | 1 | 1 | 0 | 8 | 2 | 4 | 3 | 3 | 1 | 1 | 1 | 1 | 1 | 1 | 1 | 1 | 1 | 2 | 0 | 1 | 1 | 1 | 1 | 1 | 3 | 1 | 2 | 2 | 1 | 1 | 1 | 4 | 3 | 1 | 3 | 1 | 2 | 1 | 1 | 0 | 1 | 0 | 0 | 1 | 1 | 1 | 1 | 1 | 0 | 1 | 0 | 0 | 1 | 2 | 2 | 0 | 1 | 0 | 6 | 1 | 1 | 1 | 0 | 1 | 3 | 3 | 0 | 0 | 0 | 0 | 1 | 0 | 0 | 30 | 49 | 3 | 0 | 48 | 1 | 14 |
| KMNX | 0 | 0 | 0 | 1 | 0 | 0 | 2 | 1 | 2 | 1 | 3 | 0 | 1 | 1 | 1 | 1 | 1 | 1 | 1 | 1 | 0 | 0 | 0 | 0 | 0 | 0 | 0 | 2 | 0 | 1 | 1 | 1 | 2 | 0 | 5 | 3 | 1 | 2 | 1 | 1 | 1 | 1 | 0 | 1 | 0 | 0 | 0 | 0 | 0 | 0 | 0 | 0 | 0 | 0 | 0 | 0 | 1 | 2 | 0 | 1 | 0 | 3 | 0 | 1 | 1 | 0 | 3 | 2 | 2 | 0 | 0 | 0 | 0 | 0 | 0 | 0 | 10 | 32 | 1 | 0 | 22 | 1 | 6 |
| SNOX | 0 | 1 | 2 | 1 | 1 | 1 | 9 | 0 | 4 | 4 | 3 | 1 | 1 | 1 | 1 | 1 | 1 | 1 | 1 | 1 | 1 | 0 | 1 | 2 | 0 | 1 | 0 | 3 | 0 | 2 | 2 | 1 | 1 | 0 | 6 | 4 | 1 | 3 | 1 | 2 | 0 | 1 | 1 | 2 | 0 | 0 | 1 | 1 | 1 | 1 | 2 | 1 | 1 | 1 | 0 | 1 | 1 | 2 | 0 | 1 | 0 | 8 | 1 | 1 | 1 | 1 | 3 | 4 | 3 | 0 | 0 | 0 | 1 | 1 | 1 | 1 | 41 | 76 | 6 | 0 | 51 | 2 | 25 |
| XRTZ | 0 | 1 | 1 | 1 | 0 | 1 | 12 | 1 | 3 | 3 | 2 | 1 | 1 | 1 | 1 | 1 | 1 | 1 | 1 | 1 | 1 | 0 | 1 | 2 | 1 | 1 | 1 | 0 | 1 | 2 | 2 | 1 | 1 | 1 | 4 | 4 | 1 | 4 | 1 | 1 | 0 | 1 | 0 | 1 | 0 | 0 | 1 | 1 | 1 | 1 | 2 | 1 | 1 | 1 | 1 | 1 | 1 | 2 | 0 | 1 | 0 | 7 | 1 | 1 | 1 | 0 | 2 | 3 | 3 | 0 | 0 | 1 | 1 | 2 | 0 | 0 | 39 | 76 | 5 | 0 | 61 | 2 | 29 |
| KEYW | 0 | 1 | 1 | 1 | 0 | 1 | 4 | 0 | 4 | 4 | 3 | 2 | 1 | 1 | 1 | 1 | 1 | 1 | 1 | 1 | 1 | 0 | 1 | 2 | 1 | 1 | 0 | 3 | 0 | 2 | 2 | 1 | 2 | 0 | 7 | 4 | 1 | 3 | 1 | 1 | 1 | 1 | 0 | 1 | 0 | 0 | 0 | 0 | 0 | 0 | 0 | 1 | 1 | 1 | 0 | 1 | 1 | 3 | 0 | 1 | 0 | 6 | 1 | 1 | 1 | 1 | 1 | 3 | 3 | 0 | 1 | 1 | 0 | 1 | 0 | 1 | 38 | 71 | 6 | 0 | 59 | 2 | 22 |
| DRFX | 0 | 1 | 0 | 0 | 0 | 0 | 2 | 0 | 2 | 1 | 2 | 1 | 1 | 1 | 0 | 0 | 0 | 1 | 1 | 1 | 0 | 0 | 1 | 0 | 0 | 1 | 0 | 3 | 0 | 2 | 1 | 1 | 0 | 0 | 4 | 4 | 1 | 1 | 1 | 2 | 0 | 0 | 0 | 1 | 0 | 0 | 0 | 0 | 0 | 0 | 0 | 1 | 1 | 1 | 0 | 1 | 1 | 2 | 0 | 0 | 0 | 3 | 1 | 1 | 0 | 1 | 2 | 3 | 2 | 0 | 0 | 0 | 0 | 0 | 0 | 3 | 15 | 34 | 4 | 0 | 38 | 1 | 11 |
| AEKF | 0 | 1 | 0 | 1 | 0 | 1 | 7 | 1 | 4 | 4 | 3 | 1 | 1 | 1 | 1 | 1 | 1 | 1 | 1 | 1 | 1 | 0 | 1 | 0 | 0 | 1 | 3 | 3 | 1 | 1 | 2 | 1 | 1 | 0 | 4 | 2 | 1 | 3 | 1 | 1 | 0 | 1 | 0 | 1 | 0 | 0 | 1 | 1 | 1 | 1 | 0 | 0 | 0 | 0 | 0 | 0 | 0 | 3 | 0 | 1 | 0 | 5 | 0 | 1 | 0 | 0 | 0 | 2 | 2 | 0 | 0 | 1 | 1 | 1 | 0 | 0 | 14 | 23 | 3 | 0 | 37 | 1 | 14 |
| YSQT | 0 | 1 | 1 | 1 | 1 | 1 | 12 | 1 | 4 | 4 | 3 | 1 | 1 | 1 | 1 | 1 | 1 | 1 | 1 | 1 | 2 | 0 | 1 | 1 | 2 | 1 | 0 | 2 | 0 | 2 | 2 | 1 | 2 | 0 | 6 | 4 | 1 | 4 | 1 | 2 | 0 | 1 | 1 | 2 | 0 | 1 | 0 | 0 | 0 | 0 | 1 | 1 | 1 | 0 | 0 | 1 | 1 | 3 | 1 | 1 | 0 | 8 | 1 | 1 | 0 | 0 | 2 | 3 | 3 | 0 | 2 | 1 | 1 | 2 | 0 | 0 | 41 | 76 | 7 | 0 | 69 | 1 | 24 |
| RPQV | 0 | 1 | 1 | 1 | 1 | 1 | 12 | 1 | 4 | 4 | 4 | 2 | 2 | 1 | 1 | 1 | 2 | 1 | 1 | 1 | 1 | 0 | 1 | 1 | 0 | 1 | 0 | 3 | 0 | 2 | 2 | 1 | 2 | 0 | 6 | 2 | 1 | 3 | 1 | 2 | 0 | 1 | 0 | 2 | 0 | 0 | 1 | 1 | 1 | 1 | 1 | 1 | 1 | 0 | 0 | 1 | 1 | 3 | 0 | 1 | 0 | 6 | 1 | 1 | 1 | 0 | 1 | 2 | 3 | 0 | 0 | 1 | 1 | 2 | 0 | 2 | 41 | 71 | 8 | 0 | 72 | 1 | 28 |
| HIDG | 0 | 1 | 0 | 0 | 0 | 1 | 2 | 1 | 3 | 3 | 2 | 1 | 1 | 1 | 1 | 1 | 1 | 1 | 0 | 1 | 1 | 0 | 0 | 0 | 0 | 1 | 0 | 1 | 0 | 2 | 1 | 2 | 1 | 0 | 0 | 1 | 1 | 2 | 1 | 1 | 1 | 1 | 0 | 1 | 0 | 0 | 0 | 0 | 1 | 0 | 0 | 0 | 0 | 0 | 0 | 0 | 1 | 3 | 0 | 1 | 0 | 2 | 1 | 2 | 0 | 0 | 1 | 0 | 2 | 0 | 0 | 1 | 0 | 0 | 0 | 17 | 8 | 26 | 1 | 0 | 31 | 3 | 14 |
| RQFE | 0 | 1 | 1 | 1 | 1 | 1 | 12 | 1 | 4 | 3 | 3 | 2 | 0 | 1 | 1 | 1 | 1 | 1 | 1 | 1 | 1 | 0 | 1 | 2 | 1 | 2 | 0 | 2 | 0 | 2 | 2 | 1 | 1 | 0 | 6 | 5 | 1 | 5 | 1 | 2 | 0 | 1 | 1 | 2 | 0 | 1 | 1 | 1 | 1 | 1 | 2 | 2 | 1 | 0 | 0 | 1 | 1 | 4 | 0 | 1 | 0 | 7 | 1 | 1 | 1 | 0 | 3 | 3 | 3 | 1 | 0 | 2 | 1 | 0 | 0 | 13 | 45 | 83 | 6 | 0 | 61 | 3 | 29 |
| GYRP | 0 | 1 | 1 | 2 | 0 | 0 | 4 | 1 | 4 | 3 | 4 | 2 | 1 | 1 | 1 | 1 | 1 | 1 | 0 | 1 | 2 | 0 | 1 | 1 | 1 | 1 | 0 | 2 | 0 | 1 | 1 | 1 | 0 | 0 | 4 | 3 | 1 | 4 | 1 | 1 | 1 | 1 | 0 | 2 | 0 | 0 | 1 | 1 | 1 | 1 | 0 | 1 | 1 | 1 | 0 | 1 | 1 | 2 | 0 | 0 | 0 | 8 | 1 | 0 | 2 | 1 | 1 | 4 | 2 | 0 | 0 | 1 | 1 | 0 | 0 | 8 | 36 | 73 | 8 | 0 | 61 | 1 | 19 |
| MNNM | 0 | 1 | 1 | 1 | 0 | 1 | 6 | 1 | 4 | 4 | 3 | 2 | 1 | 1 | 1 | 1 | 1 | 1 | 0 | 1 | 0 | 0 | 1 | 0 | 0 | 1 | 1 | 1 | 1 | 2 | 2 | 1 | 1 | 0 | 6 | 5 | 1 | 4 | 1 | 2 | 1 | 1 | 0 | 2 | 0 | 0 | 1 | 1 | 1 | 1 | 0 | 0 | 1 | 0 | 0 | 1 | 0 | 4 | 0 | 1 | 0 | 7 | 1 | 1 | 0 | 0 | 0 | 1 | 3 | 0 | 0 | 0 | 1 | 1 | 0 | 1 | 19 | 50 | 6 | 0 | 47 | 1 | 22 |
| ISHC | 0 | 2 | 1 | 1 | 0 | 1 | 8 | 0 | 4 | 4 | 3 | 1 | 1 | 1 | 1 | 1 | 1 | 1 | 1 | 1 | 0 | 0 | 0 | 1 | 0 | 1 | 0 | 3 | 0 | 2 | 2 | 1 | 2 | 0 | 6 | 4 | 1 | 3 | 1 | 1 | 1 | 1 | 0 | 1 | 0 | 0 | 0 | 0 | 1 | 0 | 1 | 1 | 1 | 1 | 0 | 1 | 0 | 3 | 0 | 1 | 0 | 3 | 1 | 1 | 1 | 1 | 1 | 2 | 2 | 0 | 0 | 1 | 0 | 1 | 0 | 0 | 26 | 48 | 2 | 0 | 62 | 1 | 26 |
| QWFV | 0 | 1 | 2 | 1 | 0 | 0 | 2 | 0 | 5 | 5 | 4 | 1 | 1 | 1 | 1 | 1 | 1 | 1 | 1 | 1 | 1 | 0 | 1 | 1 | 0 | 1 | 0 | 2 | 0 | 1 | 2 | 1 | 1 | 0 | 6 | 3 | 1 | 4 | 1 | 2 | 0 | 1 | 1 | 2 | 0 | 0 | 0 | 0 | 1 | 0 | 0 | 1 | 1 | 1 | 0 | 1 | 1 | 2 | 0 | 1 | 0 | 5 | 1 | 1 | 1 | 1 | 2 | 3 | 4 | 0 | 0 | 1 | 1 | 0 | 0 | 0 | 26 | 64 | 5 | 0 | 51 | 2 | 18 |
| DFDS | 0 | 1 | 1 | 1 | 0 | 1 | 6 | 0 | 3 | 2 | 2 | 1 | 1 | 1 | 1 | 1 | 1 | 1 | 0 | 1 | 0 | 0 | 0 | 1 | 0 | 0 | 0 | 1 | 0 | 2 | 2 | 1 | 1 | 0 | 6 | 2 | 1 | 3 | 1 | 2 | 1 | 1 | 0 | 2 | 0 | 0 | 0 | 0 | 1 | 0 | 0 | 1 | 1 | 1 | 0 | 1 | 1 | 3 | 0 | 1 | 0 | 6 | 0 | 0 | 0 | 1 | 1 | 2 | 2 | 0 | 0 | 0 | 1 | 1 | 0 | 0 | 24 | 49 | 4 | 0 | 49 | 1 | 24 |
| GGWH | 0 | 1 | 2 | 1 | 1 | 1 | 12 | 1 | 4 | 3 | 3 | 1 | 1 | 1 | 1 | 1 | 1 | 1 | 1 | 1 | 1 | 0 | 1 | 2 | 1 | 1 | 0 | 2 | 0 | 2 | 2 | 1 | 1 | 0 | 7 | 5 | 1 | 4 | 1 | 1 | 1 | 1 | 0 | 3 | 0 | 0 | 0 | 0 | 0 | 0 | 1 | 1 | 1 | 2 | 0 | 1 | 1 | 3 | 0 | 1 | 0 | 7 | 1 | 1 | 0 | 1 | 1 | 2 | 3 | 0 | 3 | 1 | 1 | 1 | 0 | 2 | 40 | 79 | 6 | 0 | 66 | 1 | 24 |
| WCQU | 0 | 1 | 1 | 1 | 0 | 1 | 15 | 1 | 4 | 4 | 3 | 2 | 1 | 1 | 1 | 1 | 1 | 1 | 1 | 1 | 1 | 0 | 1 | 2 | 0 | 1 | 1 | 2 | 1 | 2 | 3 | 1 | 1 | 1 | 7 | 4 | 1 | 4 | 1 | 2 | 0 | 1 | 1 | 2 | 0 | 0 | 1 | 1 | 1 | 1 | 0 | 1 | 1 | 0 | 0 | 1 | 1 | 4 | 0 | 1 | 0 | 7 | 1 | 1 | 1 | 0 | 2 | 4 | 4 | 0 | 2 | 0 | 1 | 1 | 1 | 2 | 34 | 78 | 7 | 0 | 80 | 3 | 30 |
| BHBK | 0 | 1 | 1 | 2 | 1 | 1 | 7 | 2 | 4 | 4 | 3 | 1 | 1 | 1 | 1 | 1 | 1 | 1 | 1 | 1 | 1 | 0 | 1 | 2 | 1 | 1 | 4 | 3 | 2 | 1 | 2 | 1 | 1 | 1 | 6 | 3 | 1 | 3 | 1 | 2 | 1 | 1 | 0 | 2 | 0 | 0 | 0 | 0 | 0 | 0 | 2 | 1 | 1 | 1 | 0 | 1 | 1 | 4 | 0 | 2 | 0 | 6 | 1 | 1 | 0 | 1 | 2 | 3 | 2 | 0 | 0 | 1 | 1 | 1 | 0 | 3 | 44 | 79 | 6 | 0 | 67 | 2 | 29 |
| RPRU | 0 | 1 | 2 | 1 | 0 | 1 | 14 | 2 | 3 | 3 | 3 | 1 | 1 | 1 | 1 | 1 | 1 | 1 | 1 | 1 | 0 | 0 | 1 | 2 | 1 | 1 | 0 | 3 | 0 | 2 | 2 | 1 | 1 | 0 | 6 | 4 | 2 | 3 | 1 | 2 | 0 | 1 | 1 | 3 | 0 | 1 | 1 | 1 | 1 | 1 | 2 | 1 | 1 | 0 | 0 | 1 | 1 | 4 | 0 | 1 | 0 | 8 | 1 | 1 | 1 | 0 | 4 | 3 | 3 | 1 | 3 | 1 | 1 | 2 | 0 | 5 | 49 | 94 | 7 | 0 | 74 | 1 | 32 |
| MCHJ | 0 | 1 | 2 | 1 | 0 | 1 | 10 | 1 | 4 | 4 | 3 | 3 | 1 | 1 | 1 | 1 | 1 | 1 | 0 | 1 | 1 | 0 | 1 | 2 | 0 | 1 | 1 | 2 | 1 | 2 | 1 | 1 | 1 | 0 | 6 | 3 | 1 | 3 | 1 | 2 | 1 | 1 | 0 | 1 | 0 | 0 | 1 | 1 | 1 | 1 | 1 | 1 | 1 | 1 | 0 | 1 | 1 | 3 | 0 | 1 | 0 | 8 | 1 | 1 | 1 | 1 | 2 | 3 | 3 | 0 | 0 | 1 | 1 | 0 | 0 | 5 | 36 | 76 | 7 | 2 | 68 | 2 | 34 |
| GBGT | 0 | 1 | 1 | 1 | 0 | 1 | 7 | 1 | 4 | 4 | 3 | 1 | 1 | 1 | 1 | 1 | 1 | 1 | 0 | 1 | 1 | 0 | 1 | 1 | 0 | 1 | 0 | 1 | 0 | 2 | 3 | 1 | 2 | 0 | 6 | 3 | 1 | 3 | 1 | 0 | 1 | 1 | 0 | 2 | 0 | 0 | 0 | 0 | 1 | 0 | 0 | 1 | 0 | 1 | 0 | 0 | 1 | 4 | 0 | 0 | 0 | 5 | 1 | 2 | 1 | 1 | 1 | 2 | 2 | 0 | 1 | 1 | 1 | 0 | 0 | 0 | 30 | 60 | 4 | 0 | 51 | 2 | 23 |
| MOYY | 0 | 0 | 1 | 1 | 0 | 0 | 6 | 1 | 2 | 2 | 2 | 1 | 1 | 0 | 1 | 0 | 0 | 1 | 0 | 1 | 0 | 0 | 1 | 0 | 1 | 1 | 0 | 2 | 0 | 2 | 2 | 1 | 1 | 0 | 6 | 0 | 1 | 4 | 1 | 1 | 1 | 1 | 0 | 2 | 0 | 0 | 1 | 1 | 1 | 1 | 2 | 1 | 1 | 1 | 0 | 1 | 1 | 2 | 0 | 1 | 0 | 8 | 0 | 1 | 1 | 1 | 1 | 3 | 3 | 0 | 0 | 0 | 1 | 0 | 0 | 3 | 26 | 48 | 6 | 0 | 52 | 1 | 14 |
| STKJ | 0 | 1 | 0 | 2 | 1 | 1 | 6 | 1 | 0 | 0 | 0 | 1 | 1 | 1 | 1 | 0 | 2 | 1 | 0 | 1 | 0 | 0 | 1 | 2 | 0 | 1 | 0 | 1 | 0 | 2 | 2 | 1 | 1 | 0 | 3 | 4 | 1 | 3 | 1 | 1 | 0 | 1 | 0 | 2 | 0 | 0 | 0 | 0 | 0 | 0 | 0 | 0 | 0 | 1 | 0 | 0 | 0 | 3 | 0 | 1 | 0 | 6 | 1 | 1 | 1 | 1 | 1 | 3 | 2 | 0 | 1 | 0 | 1 | 1 | 0 | 2 | 27 | 62 | 5 | 0 | 59 | 1 | 24 |
| WDGV | 0 | 1 | 1 | 1 | 1 | 1 | 19 | 1 | 3 | 3 | 2 | 1 | 1 | 1 | 1 | 1 | 1 | 1 | 1 | 1 | 1 | 0 | 1 | 1 | 1 | 1 | 1 | 3 | 0 | 2 | 2 | 1 | 2 | 0 | 6 | 5 | 1 | 4 | 1 | 2 | 0 | 2 | 0 | 2 | 0 | 0 | 1 | 1 | 1 | 1 | 2 | 1 | 1 | 1 | 0 | 1 | 1 | 3 | 1 | 1 | 0 | 7 | 1 | 1 | 1 | 1 | 3 | 4 | 3 | 1 | 2 | 1 | 1 | 2 | 0 | 1 | 42 | 79 | 7 | 0 | 78 | 2 | 33 |
| MXEZ | 0 | 1 | 0 | 1 | 1 | 1 | 11 | 2 | 3 | 3 | 2 | 2 | 1 | 1 | 1 | 1 | 1 | 1 | 1 | 1 | 0 | 0 | 0 | 1 | 0 | 1 | 0 | 2 | 0 | 1 | 1 | 1 | 1 | 0 | 4 | 2 | 0 | 3 | 1 | 2 | 3 | 1 | 0 | 1 | 0 | 0 | 0 | 0 | 1 | 0 | 0 | 1 | 1 | 1 | 0 | 1 | 1 | 3 | 2 | 1 | 1 | 9 | 1 | 1 | 1 | 1 | 1 | 0 | 0 | 0 | 1 | 2 | 1 | 1 | 1 | 1 | 46 | 95 | 2 | 0 | 70 | 1 | 37 |
| MJMQ | 0 | 1 | 0 | 0 | 0 | 0 | 2 | 0 | 1 | 1 | 1 | 0 | 0 | 0 | 0 | 1 | 0 | 0 | 0 | 0 | 0 | 0 | 1 | 0 | 0 | 0 | 0 | 1 | 0 | 1 | 1 | 1 | 1 | 0 | 2 | 4 | 0 | 0 | 1 | 0 | 0 | 1 | 0 | 0 | 0 | 0 | 0 | 0 | 0 | 0 | 0 | 0 | 0 | 0 | 0 | 0 | 0 | 1 | 0 | 0 | 0 | 4 | 0 | 0 | 0 | 0 | 0 | 0 | 0 | 0 | 0 | 0 | 0 | 0 | 0 | 2 | 18 | 34 | 1 | 2 | 23 | 1 | 9 |
| YTYU | 1 | 0 | 0 | 0 | 0 | 0 | 7 | 1 | 5 | 5 | 4 | 2 | 1 | 1 | 1 | 1 | 1 | 0 | 0 | 1 | 1 | 0 | 1 | 0 | 0 | 0 | 0 | 1 | 0 | 2 | 1 | 1 | 1 | 0 | 3 | 2 | 1 | 2 | 1 | 1 | 1 | 1 | 0 | 2 | 1 | 0 | 0 | 0 | 0 | 0 | 0 | 2 | 0 | 0 | 0 | 0 | 1 | 3 | 0 | 1 | 2 | 6 | 1 | 1 | 1 | 0 | 2 | 3 | 2 | 0 | 0 | 1 | 0 | 0 | 0 | 10 | 28 | 58 | 1 | 2 | 48 | 1 | 28 |
| DXNY | 0 | 1 | 1 | 1 | 1 | 1 | 6 | 1 | 4 | 4 | 3 | 1 | 1 | 1 | 1 | 1 | 1 | 1 | 1 | 1 | 0 | 0 | 1 | 1 | 0 | 0 | 1 | 2 | 1 | 2 | 2 | 1 | 1 | 1 | 7 | 3 | 1 | 5 | 1 | 2 | 1 | 1 | 1 | 1 | 0 | 0 | 1 | 1 | 1 | 1 | 1 | 2 | 1 | 1 | 0 | 1 | 1 | 2 | 1 | 1 | 0 | 7 | 1 | 1 | 1 | 1 | 3 | 6 | 5 | 0 | 2 | 0 | 1 | 1 | 1 | 8 | 36 | 75 | 6 | 0 | 56 | 3 | 36 |
| AGIO | 0 | 1 | 1 | 1 | 0 | 1 | 4 | 2 | 4 | 4 | 3 | 2 | 1 | 1 | 1 | 1 | 1 | 1 | 1 | 1 | 0 | 0 | 0 | 1 | 0 | 0 | 0 | 1 | 0 | 1 | 1 | 1 | 1 | 0 | 1 | 1 | 1 | 4 | 1 | 0 | 1 | 1 | 1 | 1 | 0 | 0 | 0 | 0 | 0 | 0 | 1 | 0 | 1 | 1 | 0 | 1 | 0 | 3 | 0 | 1 | 0 | 4 | 1 | 1 | 1 | 1 | 0 | 3 | 3 | 0 | 0 | 1 | 1 | 1 | 1 | 5 | 38 | 60 | 2 | 0 | 38 | 1 | 25 |
| ODXI | 0 | 1 | 1 | 1 | 1 | 1 | 14 | 3 | 4 | 4 | 3 | 2 | 1 | 1 | 1 | 1 | 1 | 1 | 1 | 1 | 0 | 0 | 1 | 1 | 0 | 0 | 1 | 2 | 1 | 1 | 1 | 1 | 1 | 0 | 5 | 1 | 1 | 4 | 1 | 2 | 3 | 1 | 0 | 1 | 0 | 1 | 1 | 1 | 1 | 1 | 1 | 0 | 1 | 1 | 0 | 1 | 1 | 3 | 0 | 1 | 0 | 8 | 1 | 1 | 1 | 1 | 2 | 3 | 3 | 0 | 2 | 1 | 1 | 1 | 2 | 10 | 51 | 83 | 6 | 0 | 58 | 3 | 35 |
| NTLE | 0 | 1 | 2 | 0 | 0 | 1 | 7 | 3 | 3 | 3 | 2 | 1 | 1 | 1 | 1 | 1 | 1 | 0 | 1 | 1 | 0 | 0 | 1 | 1 | 0 | 0 | 2 | 1 | 1 | 1 | 2 | 1 | 1 | 1 | 6 | 2 | 1 | 3 | 1 | 1 | 2 | 1 | 0 | 1 | 0 | 0 | 1 | 1 | 1 | 1 | 0 | 1 | 1 | 1 | 0 | 1 | 1 | 2 | 2 | 1 | 0 | 7 | 0 | 6 | 1 | 1 | 1 | 2 | 2 | 0 | 0 | 2 | 1 | 1 | 1 | 14 | 38 | 71 | 7 | 0 | 52 | 3 | 20 |
| HJVM | 0 | 1 | 1 | 1 | 0 | 1 | 8 | 1 | 2 | 2 | 2 | 1 | 1 | 1 | 1 | 1 | 2 | 1 | 0 | 1 | 1 | 0 | 1 | 1 | 0 | 1 | 0 | 1 | 0 | 3 | 2 | 1 | 1 | 0 | 5 | 3 | 1 | 3 | 1 | 1 | 1 | 1 | 0 | 2 | 0 | 0 | 0 | 0 | 1 | 0 | 0 | 1 | 0 | 1 | 0 | 0 | 1 | 4 | 0 | 1 | 0 | 8 | 1 | 1 | 1 | 1 | 3 | 3 | 2 | 0 | 2 | 1 | 1 | 1 | 0 | 2 | 34 | 62 | 6 | 0 | 67 | 1 | 25 |

Supplementary Table S4: strains analyzed for DGTS by mass spectrometry

| 1kP_Sample | species | strain name | media | DUF3419 positive? | DGTS by MS? |
|---|---|---|---|---|---|
| IRZA | Proteomonas sulcata | CCMP 704 | f/2-Si | + | + |
| NMAK | Pavlova lutheri | UTEX LB 1293 | F/2 media | + | + |
| OBUY | Porphyridium cruentum | UTEX 161 | F/2 media | - | - |
| RHVC | Dunaliella salina | SAG 42.88 | ASP-12 + soil extra | + | + |
| QRTH | Chlorococcum microstigmaticum | SAG 11.43 | Waris-H | + | + |
| PRIQ | Chlorococcum oleofaciens | SAG 30.93 | Waris-H | + | + |
| ZLQE | Stephanosphaera pluvialis | SAG 78-1a | Waris-H | + | + |
| VALZ | Chlamydomonas noctigama | SAG 36.72 | Waris-H | + | + |
| JRGZ | Chlamydomonas moewusii | SAG 1.98 | Waris-H | + | + |
| IHOI | Chloromonas oogama | SAG 9.79 | Waris-H | + | + |
| BCYF | Chlamydomonas cribrum | SAG 13.72 | Waris-H | + | + |
| IRYH | Heterochlamydomonas inaequalis | SAG 4.75 | Waris-H | + | + |
| LBRP | Chloromonas reticulata | SAG 26.86 | Waris-H | + | + |
| VJDZ | Botryococcus sudeticus | UTEX 2629 | 3N-BBM-V media | + | + |
| PZIF | Scenedesmus dimorphus | UTEX 746 | 3N-BBM-V media | + | + |
| MWAN | Chlorella minutissima | UTEX 2219 | F/2 media | + | + |
| ETGN | Botryococcus braunii | UTEX 2441 | 3N-BBM-V media | - | + |
| EEJO | Neochloris oleoabundans | UTEX 1185 | 3N-BBM-V media | - | - |
| JQFK | Picochlorum atomus | UTEX LB 1998 | F/2 media | + | + |
| HVNO | Tetraselmis chui | UTEX LB 232 | F/2 media | + | + |
| PUAN | Pedinomonas tuberculata | CCAP 1965/2 | Waris-H | + | + |
| AYPS | unidentified species | CCMP 1205 | ASP-12 | + | + |
| TGNL | Picocystis salinarum | CCMP 1897 | ASP-12 | + | - |
| HYHN | Prasinoderma coloniale | CCMP 1413 | ASP-12 | + | + |
| KYIO | Mesostigma viride | NIES 476 | Waris-H | - | - |
| MXEZ | Pycnococcus provasolii | CCMP 1203 | ASP-12 | + | + |
